# Perceptions, views and practices regarding antibiotic prescribing and stewardship among hospital physicians in Jakarta, Indonesia: a questionnaire-based survey

**DOI:** 10.1136/bmjopen-2021-054768

**Published:** 2022-05-18

**Authors:** Ralalicia Limato, Erni Juwita Nelwan, Manzilina Mudia, Monik Alamanda, Elfrida Rinawaty Manurung, Ifael Yerosias Mauleti, Maria Mayasari, Iman Firmansyah, Roswin Djaafar, Huong Thi Lan Vu, H Rogier van Doorn, Alex Broom, Raph L Hamers

**Affiliations:** 1 Eijkman-Oxford Clinical Research Unit, Jakarta, Indonesia; 2 Nuffield Department of Medicine, Centre for Tropical Medicine and Global Health, University of Oxford, Oxford, UK; 3 Department of Internal Medicine, Division of Infectious Diseases, Cipto Mangunkusumo National Hospital, Jakarta, Indonesia; 4 Faculty of Medicine, Universitas Indonesia, Jakarta, Indonesia; 5 Royal Taruma Hospital, Jakarta, Indonesia; 6 Fatmawati General Hospital, Jakarta, Indonesia; 7 St. Carolus Hospital, Jakarta, Indonesia; 8 Prof. Dr. Sulianti Saroso Infectious Disease Hospital, Jakarta, Indonesia; 9 Metropolitan Medical Centre Hospital, Jakarta, Indonesia; 10 Oxford University Clinical Research Unit, Hanoi, Viet Nam; 11 Sydney Centre for Healthy Societies, School of Social and Political Sciences, The University of Sydney, Sydney, New South Wales, Australia

**Keywords:** public health, public health, social medicine

## Abstract

**Objectives:**

Antibiotic overuse is one of the main drivers of antimicrobial resistance (AMR), especially in low-income and middle-income countries. This study aimed to understand the perceptions and views towards AMR, antibiotic prescribing practice and antimicrobial stewardship (AMS) among hospital physicians in Jakarta, Indonesia.

**Design:**

Cross-sectional, self-administered questionnaire-based survey, with descriptive statistics, exploratory factor analysis (EFA) to identify distinct underlying constructs in the dataset, and multivariable linear regression of factor scores to analyse physician subgroups.

**Setting:**

Six public and private acute-care hospitals in Jakarta in 2019.

**Participants:**

1007 of 1896 (53.1% response rate) antibiotic prescribing physicians.

**Results:**

Physicians acknowledged the significance of AMR and contributing factors, rational antibiotic prescribing, and purpose and usefulness of AMS. However, this conflicted with reported suboptimal local hospital practices, such as room cleaning, hand hygiene and staff education, and views regarding antibiotic decision making. These included insufficiently applying AMS principles and utilising microbiology, lack of confidence in prescribing decisions and defensive prescribing due to pervasive diagnostic uncertainty, fear of patient deterioration or because patients insisted. EFA identified six latent factors (overall Crohnbach’s α=0.85): awareness of AMS activities; awareness of AMS purpose; views regarding rational antibiotic prescribing; confidence in antibiotic prescribing decisions; perception of AMR as a significant problem; and immediate actions to contain AMR. Factor scores differed across hospitals, departments, work experience and medical hierarchy.

**Conclusions:**

AMS implementation in Indonesian hospitals is challenged by institutional, contextual and diagnostic vulnerabilities, resulting in externalising AMR instead of recognising it as a local problem. Appropriate recognition of the contextual determinants of antibiotic prescribing decision making will be critical to change physicians’ attitudes and develop context-specific AMS interventions.

**Strengths and limitations of this study**

- The self-developed questionnaire in this study identified a relevant set of attributes through a factor analysis optimisation process, with adequate content, face and construct validity and internal reliability. This study adds important value in the absence of adequately validated instruments regarding antimicrobial resistance and stewardship, with particular applicability for low-income and middle-income countries.
- This study had a large, varied respondent sample and high response rate among physicians at six public and private hospitals in Jakarta, Indonesia, and identified differences between physicians across hospitals, departments, work experience and medical hierarchy, which can guide priority-setting and tailoring of stewardship interventions.
- However, non-participation and the convenient hospital sample could have introduced selection bias, and the data are not necessarily representative for Jakarta or Indonesia.
- Factor analysis is based on using a ‘heuristic’, which leaves room to more than one interpretation of the same data and cannot identify causality.

## Introduction

The global rise in drug-resistant infections is one of the leading threats to public health globally, with increasing rates of morbidity, mortality and escalating healthcare costs.^1^ Misuse and overuse of antimicrobial drugs in human healthcare is one of the main drivers^2 3^ and also represents a key solution, that is, judicious use of remaining antibiotics. Globally, use of antibiotics remains largely unrestrained and poorly governed, with large, unregulated healthcare systems representing an increasingly challenging area for achieving the goal of optimisation. Substantial variations in contributing factors to inappropriate antibiotic prescribing exist across contexts, for example, diagnostic uncertainty, pressure from pharmaceutical industry or patients,^4 5^ with the structure and funding of health systems inflecting enactment of optimisation strategies, including antimicrobial stewardship (AMS).^6^

Patients infected with drug-resistant bacteria causing common bacterial infections in hospitalised populations are more likely to receive inappropriate antibiotic therapy resulting in higher mortality rates and opportunities for spreading to other patients.^7 8^ Hospital AMS programmes aim to control antimicrobial use, and have been associated with reducing hospital-acquired infections, unnecessary healthcare costs and potentially drug-resistant infections.^9–11^ However, AMS programmes in turn may jar with local constraints and practices and have been shown to have limited traction when attempts to implement occur without adequate understanding of context.^12^

The global push to enact effective AMS requires detailed, context-specific data on physicians, given their central role in the complex process of antibiotic prescribing in hospitals, which can inform on how antimicrobial resistance (AMR) is conceived, how current prescribing is rationalised, and how broad AMS principles may be experienced across contexts and nations.^13 14^ Few studies to date have been conducted on this topic in low-income and middle-income countries (LMICs), with insufficient evaluation of the psychometric properties of their measurement instruments to examine their suitability to the specific context.^4 5^

Indonesia, a diverse lower-middle-income country in Southeast Asia with the world’s fourth largest population (275 million), is particularly vulnerable for AMR, ranked among the greatest risers (29th of 76 countries) in antibiotic consumption, estimated at 2.5-fold between 2000 and 2015.^2^ The implementation of the National Action Plan for AMR has been hindered due to, among other factors, a limited evidence base of AMR epidemiology, antibiotic utilisation and rational prescribing practices.^15^ Weakly enforced antibiotic policies promote inappropriate prescribing and over-the-counter access without a prescription, and AMS is generally in an early stage of implementation.^15 16^ Although nationwide representative AMR data are not available, anecdotal evidence suggested high rates of AMR among common Gram-negative bacteria.

To identify context-specific opportunities for AMS interventions, we conducted a questionnaire-based survey among antibiotic-prescribing physicians in hospitals in Jakarta, Indonesia, to evaluate their perceptions of AMR, antibiotic prescribing practice, and views on AMS, and explore differences between physician subgroups. The study also aimed to evaluate the construct validity and psychometric properties of the questionnaire, and explore differences between physician subgroups.

## Methods

### Study design and setting

We conducted a cross-sectional survey between March and August 2019 among all antimicrobial prescribing physicians at six acute-care hospitals in Jakarta, Indonesia, as part of a mixed-method study to identify targets for quality improvement in antibiotic prescribing practices (EXPLAIN study).^17^ The participant hospitals were purposively sampled to achieve a representation of the diversity of hospital types in Jakarta, in terms of geographic location, size (small to large), health sector (public and private) and healthcare level (tertiary and secondary). In 2018, a total of 189 hospitals were operational in Jakarta province, of which 59 were public and 130 private, with a total of 15 444 and 15 425 inpatient beds, respectively.^18^ The hospital sample was then pragmatically composed based on existing collaborations, site willingness and available study resources, and included two tertiary-care government hospitals and four secondary hospitals, three of which were private hospitals, with between 134 and 853 inpatient beds, situated across all five districts of Jakarta. At the time of the survey, all six hospitals had an AMS programme, although at various stages of AMS implementation, initiated between 2009 and 2018.

### Study respondents

All qualified physicians prescribing antibiotics on a regular basis working were eligible for the survey and were approached to participate. The survey intended to include physicians across all clinical departments, work experience and professional hierarchy, to ensure representation of different antibiotic practices and views, perceptions and experiences related to AMR and AMS.^19^ We included interns/internship doctors (magang/dokter internsip; recent graduates working under supervision of a specialist), general practitioners (GPs) (dokter umum; physicians who practice general medicine treating common, non-specialised medical conditions), residents (residen; physicians who are in specialist training), specialist/consultant physicians (dokter spesialis/konsultan; specialised in a particular area of medicine) and other physicians.

### Patient and public involvement statement

Patients or the public were not involved in the design, conduct or reporting of the research.

### Survey questionnaire

We developed a two-page anonymous, self-administered, paper-based questionnaire, which was easy to complete and based on a conceptual framework that included attributes related to prescribers’ perceptions, views and practices. Good practice recommendations for questionnaire design and existing questionnaires in the literature were reviewed and discussed with several experts. The Clinician Pre/Post Perception Survey of the Greater New York Hospital Association United Hospital Fund^20^ constituted the initial set of items, supplemented with relevant items from other existing questionnaires.^21–27^ From a preliminary pool, we selected 69 items, of which eight items were worded in the negative to address the acquiescence effect. The instrument was translated from English to Indonesian, and back-translated by an independent translator. The questionnaire was pretested by a convenience panel of 18 physicians (2 GPs, 15 residents and 1 consultant). According to their feedback, we made adjustments to clarify ambiguous items, and remove redundant items. The final version consisted of 40 items and took about 10 min to complete.

The final questionnaire included an explanation of study purpose and completion instructions; 40 short statements (items) to which participants were asked to indicate the extent to which each reflected their own opinion on a 5-point Likert scale, divided into three sections: scope of the AMR problem and key contributors; antibiotic prescribing practices; AMS and respondent sociodemographics (online supplemental appendix).

10.1136/bmjopen-2021-054768.supp1 Supplementary data

### Respondent recruitment

The hospital management provided the total number of prescribing physicians for each department. The questionnaires were delivered to the head of each unit who then distributed the survey to all eligible staff. The study coordinator kept a record of numbers of physicians approached and participated. On survey completion, respondents could enter a raffle to win one of three gift cards in each hospital (US$14 each); there were no other incentives for participation.

### Statistical analysis

The percentage of respondents selecting each answer choice was calculated using the total number of responses as the denominator. For an exploratory factor analysis (EFA), a common lower bound for sample size is 10 cases per variable, suggesting a minimum sample size of 400; to allow for meaningful subgroup comparisons and minimise selection bias, we targeted a>50% response rate and a sample size of >1000 across the six hospitals.

We performed EFA to identify underlying distinct constructs, using factor, pcf command in Stata with orthogonal (varimax) rotation. For this analysis, the eight items worded in the negative were reverse-coded, and missing data for categorical variables were treated as a separate category. The Kaiser-Meyer-Olkin (KMO) was calculated to ensure EFA requirements were met. Each item was assigned to a certain factor based on the highest absolute factor loading of the rotated solution. To determine the optimal factor solution, we used the Kaiser criterion, scree plot of eigenvalues, and Horn’s parallel analysis (*paran* package in Stata). Parallel analysis adjusts the original eigenvalues for sampling error induced collinearity among the variables to arrive at adjusted eigenvalues. Analogous to the Kaiser criterion, only factors with adjusted eigenvalues >1 were retained. The scree plot of eigenvalues and the scree plot of parallel analysis were visually inspected and factors were retained above the inflection. To interpret the factor solutions, we examined the factor loadings, and assigned each item to a certain factor based on the highest absolute factor loading (minimum loading 0.30), and then produced an umbrella label for each factor that best characterised the joint meaning of all the variables associated with it. If a certain item did not fit with the factor structure (eg, the results were not interpretable or a factor comprised less than three items), the analysis was rerun without the pertinent item(s). After the optimal factor solution had been achieved, we used the regression scoring method to compute the factor scores based on the factor loadings, and Cronbach’s α coefficient to test internal reliability. Using the factor scores as the dependent variable, we developed six (one for each factor score) multivariable linear regression models to assess associations with key physician characteristics (‘subgroups’) as the following five independent variables, that is, hospital sector and care level, grouped departments, work experience and medical hierarchy. Given the hierarchical structure of the data (respondents clustered within hospitals) we applied multilevel (two-level) modelling to control for possible clustering within hospitals. We also adjusted for possible confounders. Values of p<0.05 were considered statistically significant. All analyses were done with Stata/IC v16.1 (StataCorp).

## Results

### Respondent characteristics

All 1896 antibiotic prescribing physicians at the six hospitals were approached, and 1007 (53.1%) participated in the survey. Table 1 summarises the participants’ key characteristics. Online supplemental table S1 summarises the response rates.

10.1136/bmjopen-2021-054768.supp2 Supplementary data

**Table 1:** Characteristics of respondents

Total	1007
Sex*	
Female	477 (47.4)
Male	524 (52.0)
Professional hierarchy	
Intern/internship doctor	10 (1.0)
General practitioner	113 (11.2)
Resident	500 (49.7)
Specialist/consultant	358 (35.6)
Other	18 (1.8)
Professional experience (years)†	
<1	194 (19.3)
1–5	459 (45.6)
6–10	136 (13.5)
11–15	74 (7.4)
16–20	52 (5.2)
>20	81 (8.0)
Grouped departments‡§	
Surgery (including subspecialties)	371 (36.8)
Medicine (including subspecialties)	232 (23.0)
Acute specialties	156 (15.5)
Other departments	244 (24.2)
Information sources used to guide prescribing¶	
Guidelines	
International	619 (61.5)
National	628 (62.4)
Hospital	656 (65.1)
Department/division	405 (40.2
Consultation with senior colleague(s)	472 (46.9)
Consultation with microbiologist/infectious disease physician	523 (51.9)
Textbooks	410 (40.7)
Medical journals	389 (38.6)
Pharmaceutical company representative	34 (3.4)
Internet	115 (11.4)
Other	13 (1.3)
No of AMR/AMS trainings attended in the past year**	
0	396 (39.3)
1	342 (34.0)
≥2	168 (16.7)
Median (range)	1 (0, 10)

Data are reported as n (%) unless indicated otherwise.

*Data missing for: 6 (0.60%).

†11 (1.1%).

‡4 (0.40%).

§Surgery and surgical subspecialties includes obstetrics/gynaecology (146), surgery (122), orthopaedics (57), ENT (32), urology (14); medicine and medical subspecialties includes medicine (128), neurology (63), pulmonology (15), dermatology (14), cardiology (12); acute specialties includes anaesthesiology (72), emergency (57), ICU (27); other departments includes paediatrics (54), ophthalmology (39), multiple units (33), rehabilitation (32), psychiatry (30), dentist (27), other (29) and unspecified (4).

¶2 (0.20%).

**101 (10.0%).

AMR, antimicrobial resistance; AMS, antibiotic stewardship.

### Description of respondent responses

**Figure 1:**
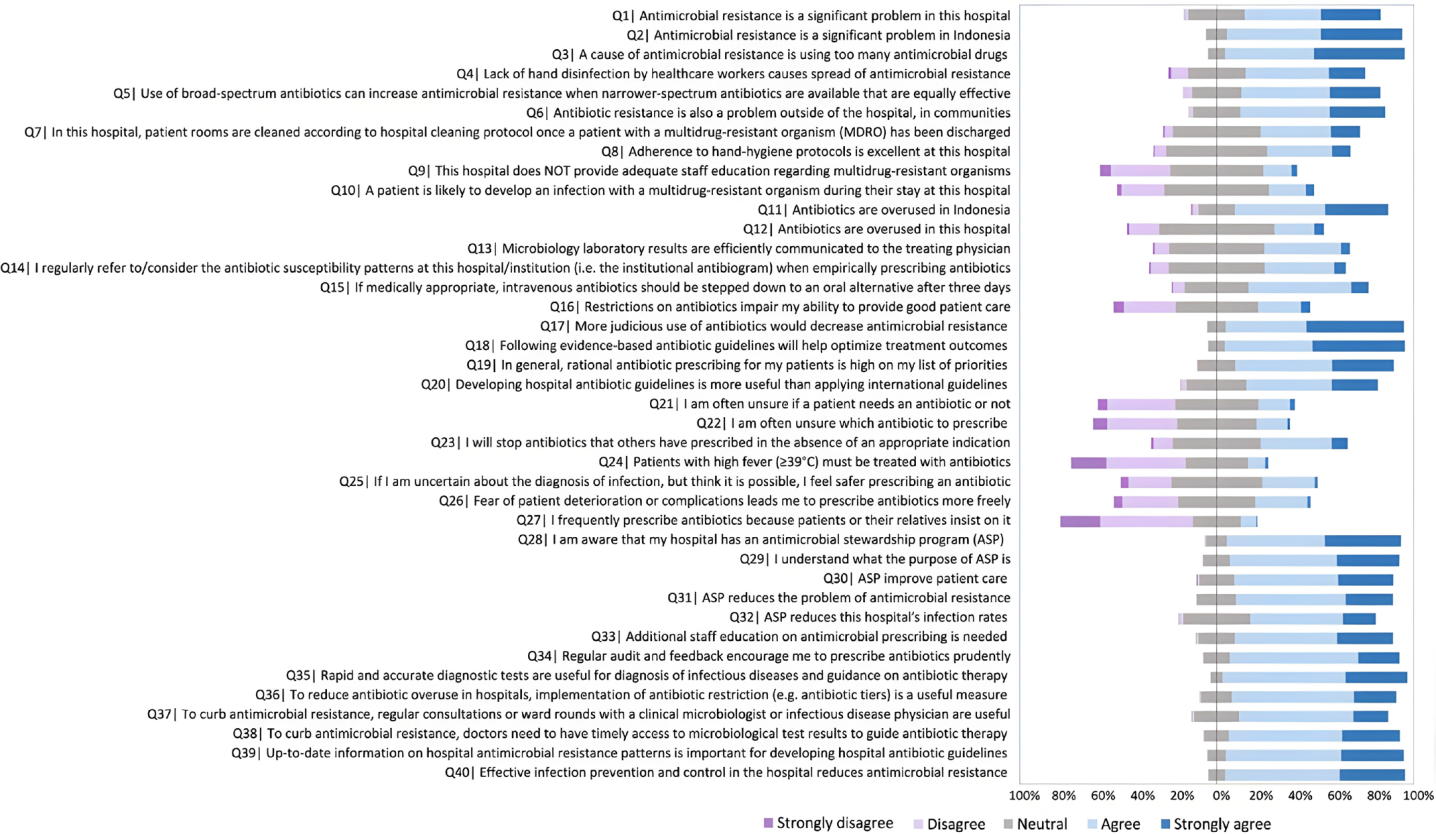
Five-point Likert scale responses for the 40-item questionnaire. Data (n, %) are summarised in online supplemental table S2. ASP, antibiotic stewardship programme

Figure 1 and online supplemental table S2 summarise the responses to all 40 items.

#### Scope of the AMR problem and key contributors

Most respondents agreed that AMR is an important problem in Indonesia (93.8%; 944/1006) (Q2); in communities outside of the hospital (83.6%; 838/1003) (Q6); and at their hospital (80.4%; 808/1005) (Q1), with 30.9% (311/1005) agreeing that patients are likely to develop an infection with a multidrug-resistant infection (Q10). Most acknowledged as key contributing factors: overuse of antimicrobial drugs (95.1%; 954/1003) (Q3), lack of hand hygiene (71.1%; 715/1005) (Q4), use of broad-spectrum antibiotics (80.5%; 808/1004) (Q5). Current infection and prevention control (IPC) practices at their hospital were regarded as suboptimal: 64.8% (651/1004) thought that patient rooms are cleaned according to hospital protocol after discharge of a patient with a multidrug-resistant organism (Q7); 56.9% (570/1002) thought adherence to hand-hygiene protocols to be excellent (Q8); and 22.5% (226/1005) felt that their hospital does not provide adequate staff education regarding multidrug-resistant organisms (Q9).

#### Antibiotic prescribing practices

Whereas most respondents (85.7%; 861/1005) agreed that antibiotics are overused in Indonesia (Q11), only 35.5% (357/1005) acknowledged this to be case at their hospital (Q12). Most agreed that more judicious antimicrobial prescribing practices would decrease AMR (94.8%; 953/1005) (Q17) and that following evidence-based antibiotic guidelines will help optimise treatment outcomes (95.3%; 958/1006) (Q18). Most gave high priority to rational antibiotic prescribing to their patients (88.8%; 892/1005) (Q19), and considered developing hospital antibiotic guidelines more useful than applying international guidelines (78.4%; 787/1004) (Q20). Nearly a quarter of respondents indicated to be often unsure if a patient needs an antibiotic or not (23.6%; 237/1006) (Q21) or which antibiotic to prescribe (21.3%; 215/1006) (Q22). A small but considerable fraction expressed lack of confidence in prescribing decisions, that is, 12.2% (123/1005) prescribed in patients with just a high fever (≥39°C) (Q24), 36.6% (368/1005) when they felt uncertain about the diagnosis of infection (Q25), 35.0% (352/1006) prescribed more freely because of fear of clinical failure (Q26), and 9.8% (98/1005) frequently prescribed antibiotics because patients or their relatives insist (Q27). Just more than half of the respondents reported that microbiology laboratory results are efficiently communicated to the treating physician (57.3%; 576/1005) (Q13), considered the hospital antibiogram when empirically prescribing antibiotics (54.5%; 548/1005) (Q14), and would stop antibiotics that others have prescribed in the absence of an appropriate indication (57.0%; 571/1002) (Q23). Most (72.7%; 731/1006) agreed that, if medically appropriate, intravenous antibiotics should be stepped down to an oral alternative after 3 days (Q15). Notably, 33.6% (338/1006) felt that restrictions on antibiotics impaired their ability to provide good patient care (Q16).

#### Antibiotic stewardship

When asked about AMS in general, most respondents were aware that their hospital had an AMS programme (93.1%; 937/1006) (Q28), they reported they understood its purpose (92.1%; 927/1006) (Q29), and they agreed that AMS can improve patient care (88.6%; 891/1006) (Q30), reduce AMR (88.3%; 887/1005) (Q31) and hospital-acquired infections (76.7%; 770/1004) (Q32). When asked about the usefulness of specific AMS activities, most respondents acknowledged that additional education on antibiotic prescribing was needed (88.4%; 888/1005) (Q33), regular audit and feedback encouraged them to prescribe antibiotics prudently (92.2%; 928/1006) (Q34), rapid and accurate diagnostic tests are useful for diagnosis of infectious diseases and guidance on antibiotic therapy (96.5%; 971/1006) (Q35), implementation of antibiotic restriction (eg, antibiotic tiers) can reduce antibiotic overuse in hospitals (90.4%; 910/1007) (Q36), regular consultations or ward rounds with a clinical microbiologist or infectious disease physician can curb AMR (85.4%; 859/1006) (Q37), timely access to microbiological test results is needed to guide antibiotic therapy (92.4%; 930/1006) (Q38), and IPC in the hospital can reduce AMR (95.3%; 959/1006) (Q40).

### Exploratory factor analysis

The KMO was 0.8773 overall and >0.5 for all items, suggesting the data were suitable for EFA. Analysis of the scree plot (online supplemental figure S1) indicated a case for four factors, whereas the parallel analysis (online supplemental figure S2) indicated a case for seven factors. The four-factor solution yielded strong factors but explained only 39.9% of the variance and lacked a theoretical basis for one factor. The seven-factor solution contained one factor with only three items that was difficult to interpret; two of these items (Q9 and Q10) did not load well with any factor in various alternative factor solutions and were removed. Therefore, a six-factor model with a clear theoretical basis based on 38 items was deemed most suitable, explaining 47.4% of the variance, with KMO 0.8802 overall and >0.5 for each item (table 2). The six latent factors are (table 3, see online supplemental table S3 for details): (1) Awareness of AMS activities; (2) Awareness of AMS purpose; (3) Views regarding rational antibiotic prescribing; (4) Confidence in antibiotic prescribing decisions; (5) Perception of AMR as a significant problem and (6) Immediate actions to contain AMR. Internal reliability was excellent for the overall 38-item scale (α=0.85) and factor 1 (α=0.8734) and 2 (α=0.8334), good for factor 3, 4 and 5 (α=0.70 each) and acceptable for factor 6 (α=0.57).

**Table 2:** Summary of the exploratory factor analysis of the six-factor solution (n=973)

Item #	Original item	Rotated factor loadings						Uniqueness
		Factor 1	Factor 2	Factor 3	Factor 4	Factor 5	Factor 6	
**Q01**	Antimicrobial resistance is a significant problem in this hospital	0.2159	0.0410	0.1389	0.0057	0.5701	0.0416	0.6056
**Q02**	Antimicrobial resistance is a significant problem in Indonesia	0.2433	0.1073	0.3295	0.0588	0.5742	0.0385	0.4861
**Q03**	A cause of antimicrobial resistance is using too many antimicrobial drugs	0.1641	0.2006	0.3993	0.0695	0.5361	−0.0462	0.4790
**Q04**	Lack of hand disinfection by healthcare workers causes spread of antimicrobial resistance	0.0640	0.0902	−0.2533	0.0152	0.5725	0.1179	0.5817
**Q05**	Use of broad-spectrum antibiotics can increase antimicrobial resistance when narrower-spectrum antibiotics are available that are equally effective	0.2150	0.0536	0.1482	0.0005	0.5480	0.1093	0.6167
**Q06**	Antibiotic resistance is also a problem outside of the hospital, in communities	0.2088	0.0318	0.2463	0.0632	0.4766	0.0432	0.6617
**Q07**	In this hospital, patient rooms are cleaned according to hospital cleaning protocol once a patient with a multidrug-resistant organism has been discharged	0.1038	0.0753	0.0868	0.0210	0.0378	0.6058	0.6071
**Q08**	Adherence to hand-hygiene protocols is excellent at this hospital	−0.0098	0.0364	0.0606	0.1099	−0.0830	0.6368	0.5705
**Q11**	Antibiotics are overused in Indonesia	0.1924	0.1831	0.5138	−0.0809	0.2489	−0.2318	0.5432
**Q12**	Antibiotics are overused in this hospital	0.0548	0.0417	0.2094	−0.4550	0.1635	−0.2381	0.6610
**Q13**	Microbiology laboratory results are efficiently communicated to the treating physician	0.1689	0.1174	−0.0915	0.0532	−0.0963	0.5116	0.6754
**Q14**	I regularly refer to/consider the antibiotic susceptibility patterns at this hospital/institution (ie, the institutional antibiogram) when empirically prescribing antibiotics	0.0288	0.1070	−0.0292	−0.0237	0.2056	0.6115	0.5701
**Q15**	If medically appropriate, intravenous antibiotics should be stepped down to an oral alternative after 3 days	−0.0873	0.2085	0.1141	−0.0850	0.2311	0.3617	0.7444
**Q16**	Restrictions on antibiotics impair my ability to provide good patient care	0.0460	0.1839	0.0527	0.4031	0.0843	−0.0868	0.7842
**Q17**	More judicious use of antibiotics would decrease antimicrobial resistance	0.3010	0.0747	0.7362	0.0820	0.0892	0.0648	0.3429
**Q18**	Following evidence-based antibiotic guidelines will help optimise treatment outcomes	0.2274	0.1934	0.6565	0.1206	0.1256	0.1851	0.4153
**Q19**	In general, rational antibiotic prescribing for my patients is high on my list of priorities	0.1845	0.1804	0.5246	0.1574	0.1184	0.3228	0.5151
**Q20**	Developing hospital antibiotic guidelines is more useful than applying international guidelines	0.1803	0.0075	0.3499	−0.1335	0.2631	0.0672	0.7534
**Q21**	I am often unsure if a patient needs an antibiotic or not	0.0489	0.0260	0.0497	0.5640	−0.3364	0.1021	0.5527
**Q22**	I am often unsure which antibiotic to prescribe	−0.0084	0.0457	0.1256	0.5670	−0.1938	0.0781	0.6170
**Q23**	I will stop antibiotics that others have prescribed in the absence of an appropriate indication	0.0016	−0.0620	0.1517	−0.1137	0.2090	0.3892	0.7650
**Q24**	Patients with high fever (≥39°C) must be treated with antibiotics	0.0077	0.1331	0.1695	0.4794	0.2095	−0.2351	0.6245
**Q25**	If I am uncertain about the diagnosis of infection, but think it is possible, I feel safer prescribing an antibiotic	0.0217	−0.0415	−0.0877	0.6741	0.1927	−0.0086	0.4985
**Q26**	Fear of patient deterioration or complications leads me to prescribe antibiotics more freely	0.0113	0.0039	−0.1459	0.7092	0.0883	0.0246	0.4672
**Q27**	I frequently prescribe antibiotics because patients or their relatives insist on it	0.1069	0.0474	0.2869	0.6318	0.0518	−0.0100	0.5021
**Q28**	I am aware that my hospital has an antimicrobial stewardship programme (ASP)	0.2434	0.6224	0.2918	0.0866	0.0418	0.0110	0.4588
**Q29**	I understand what the purpose of ASP is	0.2217	0.6957	0.2635	0.1188	0.0092	0.0199	0.3828
**Q30**	ASP improve patient care	0.2364	0.7744	0.0873	0.0932	0.0852	0.1241	0.3055
**Q31**	ASP reduces the problem of antimicrobial resistance	0.2775	0.7532	0.0949	0.0172	0.0906	0.1753	0.3074
**Q32**	ASP reduces this hospital’s infection rates	0.2045	0.6670	−0.1122	0.0569	0.1283	0.1799	0.4486
**Q33**	Additional staff education on antimicrobial prescribing is needed	0.5202	0.2816	0.0917	−0.0079	0.2203	−0.0284	0.5923
**Q34**	Regular audit and feedback encourage me to prescribe antibiotics prudently	0.6151	0.3581	0.0075	0.0064	0.1402	0.1117	0.4612
**Q35**	Rapid and accurate diagnostic tests are useful for diagnosis of infectious diseases and guidance on antibiotic therapy	0.6714	0.2576	0.1522	−0.0362	0.0386	0.0379	0.4555
**Q36**	To reduce antibiotic overuse in hospitals, implementation of antibiotic restriction (eg, antibiotic tiers) is a useful measure	0.6428	0.2670	0.1088	−0.0335	0.2153	0.0013	0.4562
**Q37**	To curb antimicrobial resistance, regular consultations or ward rounds with a clinical microbiologist or infectious disease physician are useful	0.7046	0.0787	0.0061	0.0190	0.1881	0.0781	0.4555
**Q38**	To curb antimicrobial resistance, doctors need to have timely access to microbiological test results to guide antibiotic therapy	0.7197	0.0835	0.2786	0.0985	0.0670	0.0820	0.3765
**Q39**	Up-to-date information on hospital antimicrobial resistance patterns is important for developing hospital antibiotic guidelines	0.7374	0.0854	0.3223	0.1088	0.1739	0.0348	0.3018
**Q40**	Effective infection prevention and control in the hospital reduces antimicrobial resistance	0.7067	0.1690	0.2876	0.0950	0.1798	0.0533	0.3452
	Eigenvalues	4.39	3.19	2.82	2.78	2.65	2.17	
	% of variance explained	11.56	8.40	7.43	7.32	6.98	5.72	Overall 47.4%

Table shows the results of the exploratory factor analysis (principal axis factoring) with orthogonal varimax rotation of the six-factor solution using the factor, pcf command in Stata.

Rotated factor loadings: a measure of how much each item contributes to the factor. Loadings close to −1 or 1 indicate that the factor strongly affects the item and loadings close to 0 indicate that the factor has a weak effect on the item.

Item #9 and 10 were excluded from the analysis as explained in the Results section.

Uniqueness: shows the proportion of the item’s variance that is not explained by the factors

**Table 3:** The latent factors of antibiotic prescribing

Factor	Factor label	No of items	Original item #	Loadings range	Reliability (Cronbach’s α)
**1**	Awareness of AMS activities	8	33–40	0.5202, 0.7374	0.8734
**2**	Awareness of AMS purposes	5	28–32	0.6224, 0.7744	0.8334
**3**	Views regarding rational antibiotic prescribing	5	11, 17–20	0.3499, 0.7362	0.6961
**4**	Confidence in antibiotic prescribing decisions	8	12, 16, 21, 22, 24–27	0.4031, 0.7092	0.6997
**5**	Perception of AMR as a significant problem	6	1–6	0.4766, 0.5742	0.6967
**6**	Immediate actions to contain AMR	6	7, 8, 13–15, 23	0.3617, 0.6368	0.5695

Item #9 and 10 were excluded from the analysis, as explained in the Results section.

The full table is included in online supplemental table S2.

AMR, antimicrobial resistance; AMS, antibiotic stewardship.

### Physician subgroup analysis

Table 4 summarises the results of the subgroup analysis.

**Table 4:** Physician subgroup analysis

	Factor 1			Factor 2			Factor 3			Factor 4			Factor 5			Factor 6		
	Coeff	95% CI	P value	Coeff	95% CI	P value	Coeff	95% CI	P value	Coeff	95% CI	P value	Coeff	95% CI	P value	Coeff	95% CI	P value
**Hospital**																		
05	Ref			Ref			Ref			Ref			Ref			Ref		
01	0.194	−0.089 to 0.477	0.179	−**0.422**	−**0.701 to** −**0.142**	**0.003**	0.244	−0.039 to 0.527	0.091	−0.167	−0.451 to 0.117	0.248	−**0.430**	−**0.716 to** −**0.145**	**0.003**	−**0.504**	−**0.778 to** −**0.23**	**0.000**
02	0.064	−0.13 to 0.258	0.518	−**0.352**	−**0.543 to** −**0.161**	**0.000**	0.134	−0.06 to 0.328	0.174	−0.180	−0.375 to 0.014	0.069	−0.095	−0.29 to 0.101	0.342	−0.087	−0.274 to 0.101	0.366
03	0.164	−0.116 to 0.444	0.252	−0.066	−0.343 to 0.211	0.641	**0.387**	**0.107 to 0.668**	**0.007**	0.131	−0.151 to 0.412	0.363	−0.044	−0.327 to 0.238	0.758	0.092	−0.18 to 0.364	0.507
04	0.117	−0.218 to 0.453	0.494	0.116	−0.215 to 0.448	0.492	0.096	−0.239 to 0.432	0.573	0.249	−0.088 to 0.586	0.148	−0.081	−0.419 to 0.257	0.638	**0.361**	**0.035 to 0.686**	**0.030**
06	0.196	−0.153 to 0.544	0.271	0.106	−0.239 to 0.45	0.548	**0.419**	**0.07 to 0.768**	**0.019**	0.013	−0.338 to 0.363	0.944	−**0.432**	−**0.783 to** −**0.08**	**0.016**	**0.479**	**0.141 to 0.817**	**0.005**
**Departments**																		
Surgical	Ref			Ref			Ref			Ref			Ref			Ref		
Medical	**0.239**	**0.073 to 0.405**	**0.005**	0.150	−0.014 to 0.314	0.073	−0.120	−0.286 to 0.046	0.156	**0.307**	**0.140 to 0.473**	**0.000**	**0.221**	**0.054 to 0.389**	**0.009**	−**0.398**	−**0.559 to** −**0.237**	**0.000**
Acute	−**0.275**	−**0.488 to** −**0.062**	**0.011**	−**0.214**	−**0.425 to** −**0.004**	**0.046**	−**0.273**	−**0.486 to** −**0.06**	**0.012**	0.169	−0.045 to 0.382	0.122	0.124	−0.091 to 0.338	0.258	**0.242**	**0.036 to 0.449**	**0.021**
Other	−0.081	−0.253 to 0.092	0.358	−0.027	−0.197 to 0.144	0.760	−0.086	−0.259 to 0.086	0.326	0.046	−0.127 to 0.219	0.603	0.119	−0.055 to 0.292	0.181	−**0.302**	−**0.469 to** −**0.135**	**0.000**
Medical	Ref			Ref			Ref			Ref			Ref			Ref		
Acute	−**0.514**	−**0.745 to** −**0.283**	**0.000**	−**0.364**	−**0.593 to** −**0.136**	**0.002**	−0.153	−0.384 to 0.078	0.194	−0.138	−0.370 to 0.094	0.243	−0.098	−0.330 to 0.135	0.411	**0.640**	**0.417 to 0.864**	**0.000**
Other	−**0.320**	−**0.507 to** −**0.133**	**0.001**	−0.177	−0.361 to 0.0083	0.061	0.034	−0.154 to 0.221	0.724	−**0.261**	−**0.449 to 0.073**	**0.006**	−0.103	−0.291 to 0.086	0.285	0.096	−0.085 to 0.277	0.298
**Medical hierarchy**																		
Consultant	Ref			Ref			Ref			Ref			Ref			Ref		
Intern	−**0.744**	−**1.374 to** −**0.115**	**0.020**	**0.637**	**0.015 to 1.259**	**0.045**	0.443	−0.187 to 1.074	0.168	0.067	−0.565 to 0.699	0.836	0.495	−0.14 to 1.129	0.126	0.003	−0.607 to 0.613	0.993
GP	0.168	−0.096 to 0.432	0.212	0.153	−0.108 to 0.413	0.251	−**0.397**	−**0.661 to** −**0.133**	**0.003**	−0.169	−0.434 to 0.095	0.210	−0.011	−0.276 to 0.255	0.938	−0.024	−0.279 to 0.232	0.857
Resident	−0.091	−0.287 to 0.105	0.363	**0.297**	**0.104 to 0.490**	**0.003**	0.004	−0.192 to 0.199	0.971	−**0.271**	−**0.468 to** −**0.075**	**0.007**	0.058	−0.139 to 0.255	0.563	−**0.202**	−**0.391 to** −**0.012**	**0.037**
Other	−0.013	−0.491 to 0.466	0.958	0.148	−0.325 to 0.620	0.541	0.252	−0.227 to 0.731	0.303	−0.172	−0.653 to 0.308	0.482	−0.013	−0.495 to 0.469	0.959	0.304	−0.159 to 0.768	0.198
Intern	Ref			Ref			Ref			Ref			Ref			Ref		
GP	**0.912**	**0.245 to 1.579**	**0.007**	−0.485	−1.144 to 0.175	0.150	−0.840	−1.508 to −0.172	0.014	−0.236	−0.906 to 0.434	0.489	−0.505	−1.18 to 0.167	0.141	−0.0263	−0.673 to 0.620	0.936
Resident	**0.653**	**0.0458 to 1.261**	**0.035**	−0.340	−0.941 to −0.260	0.267	−0.440	−1.048 to 0.169	0.157	−0.338	−0.948 to 0.272	0.278	−0.436	−1.049 to 0.176	0.162	−0.205	−0.793 to 0.384	0.496
Other	0.732	−0.457 to 1.509	0.065	−0.490	−1.258 to 0.279	0.212	−0.191	−0.970 to 0.587	0.630	−0.239	−1.020 to 0.542	0.548	−0.507	−1.291 to 0.276	0.204	0.302	−0.452 to 1.055	0.433
Resident	Ref			Ref			Ref			Ref			Ref			Ref		
GP	0.259	−0.037 to 0.555	0.086	−0.145	−0.437 to 0.148	0.333	−0.400	−0.696 to −0.104	0.008	0.102	−0.196 to 0.399	0.503	−0.069	−0.367 to 0.229	0.651	0.178	−0.108 to 0.465	0.223
Other	0.0781	−0.417 to 0.574	0.757	−0.150	−0.639 to 0.340	0.550	0.248	−0.248 to 0.744	0.326	0.099	−0.399 to 0.596	0.697	−0.710	−0.570 to 0.428	0.780	**0.506**	−**0.163 to 1.29**	**0.039**
**Years in current profession**																		
0–5	Ref			Ref			Ref			Ref			Ref			Ref		
6–10	0.089	−0.122 to 0.299	0.410	0.225	**0.017 to 0.434**	**0.034**	−0.089	−0.300 to 0.122	0.410	0.094	−0.118 to 0.305	0.385	**0.290**	**0.077 to 0.502**	**0.008**	0.053	−0.151 to 0.258	0.608
11–15	0.087	−0.185 to 0.358	0.532	−0.015	−0.283 to 0.254	0.915	−0.066	−0.338 to 0.207	0.637	0.271	−0.002 to 0.544	0.052	0.156	−0.118 to 0.43	0.265	−0.095	−0.358 to 0.169	0.481
>15	0.117	−0.118 to 0.353	0.328	0.194	−0.039 to 0.427	0.102	−0.044	−0.279 to 0.192	0.715	−0.057	−0.293 to 0.179	0.637	**0.333**	**0.096 to 0.57**	**0.006**	**0.366**	**0.138 to 0.594**	**0.002**
Public sector*	−0.064	−0.4 to 0.273	0.710	0.264	−0.207 to 0.736	0.272	−0.238	−0.575 to 0.100	0.167	0.256	−0.835 to 0.595	0.140	0.192	−0.148 to 0.533	0.268	0.362	0.273 to 0.996	0.264
Tertiary level*	−0.093	−0.42 to 0.234	0.577	−0.289	−0.767 to 0.190	0.237	−0.047	−0.375 to 0.281	0.779	−0.317	−0.647 to 0.012	0.059	0.052	−0.279 to 0.383	0.758	−0.407	−1.065 to 0.25	0.225

The table summarises the results of the multivariable mixed-effect linear regression models to assess the associations between hospital (#1–6), department, medical hierarchy, work experience, health sector, healthcare level as the independent variables of interest and each of the factor scores (#1–6) as the dependent variable. Each model adjusted for the possible interdependence of observations clustered within hospitals, as well as for the confounders sex and AMS training. Values in bold indicate statistical significance.

*This model did not include hospital as an independent variable due to collinearity.

AMS, antimicrobial stewardship; GP, general practitioner.

#### Hospitals

Statistically significant differences were identified between hospitals for awareness of AMS purposes (factor 2), views regarding rational antibiotic prescribing (factor 3), perception of AMR as a significant problem (factor 5) and immediate actions to contain AMR (factor 6), but not for awareness of AMS activities (factor 1) and confidence in antibiotic prescribing decisions (factor 4). None of the factor scores differed between prescribers in public vs private, or secondary vs tertiary hospitals.

#### Professional hierarchy

For awareness of AMS activities (factor 1), consultants, GPs and residents scored higher than interns. For awareness of AMS purposes (factor 2), consultants scored lower than interns and residents. For views regarding rational antibiotic prescribing (factor 3), consultants scored higher than GPs. For confidence in antibiotic prescribing decisions (factor 4), consultants scored lower than residents, whereas for immediate actions to contain AMR (factor 6), consultants scored higher than residents. No differences were identified for perception of AMR as a significant problem (factor 5).

#### Departments

For awareness of AMS activities (factor 1) and purpose (factor 2), physicians in surgery and medicine scored higher than the acute specialties, whereas for awareness of AMS activities (factor 1), medicine scored higher than surgery. For views regarding rational antibiotic prescribing (factor 3), surgery scored higher than acute specialties. For confidence in antibiotic prescribing decisions (factor 4), medicine scored lower than surgery and other specialties. For perception of AMR as a significant problem (factor 5), surgery scored lower than medicine. For immediate actions to contain AMR (factor 6), surgery scored higher than medicine and other specialties and the acute specialties scored higher than surgery and medicine.

#### Work experience

Physicians with little (0–5 years) work experience scored lower than more experienced colleagues, for awareness of AMS purpose (factor 2); for perception of AMR as a significant problem (factor 5); and for immediate actions to contain AMR (factor 6). No differences were identified for factors 1, 3 and 4.

## Discussion

This survey, among over 1000 physicians in Indonesian hospitals, assessed their perceptions and views regarding the AMR problem and its key contributors, antibiotic prescribing practices and AMS. Starting from those three broad themes, we used EFA to identify distinct underlying constructs in the dataset, that is, (1) awareness of AMS activities; (2) awareness of AMS purposes; (3) views regarding rational antibiotic prescribing; (4) confidence in antibiotic prescribing decisions; (5) perception of AMR as a significant problem and (6) immediate actions to contain AMR. The survey findings thus outline a series of dynamics around AMR and AMS in the Indonesian context. Spanning issues around awareness (education)^28^ (factors 1, 2, 5), visibility (diagnostics)^29^ (factor 3, 4 and 6), and institutional form (governance)^30^ (factors 3 and 6), the survey results tease out many of the core issues illustrated in other settings,^4 5^ but in turn, illustrate that several specific factors play a role, that is, uncertainty, risk and lack of sense of responsibility.

In this respect, an important finding was that only few physicians recognised that antibiotic overuse was an issue at their own hospital, that many physicians were hesitant to stop antibiotics without an appropriate indication that others had prescribed, and that many felt that antibiotic restrictions impaired their ability to provide good patient care. Lack of confidence in prescribing decisions and defensive prescribing were common due to diagnostic uncertainty, fear of patient deterioration or complications, or because patients or their relatives insisted. These views and perceptions concur with the observed overuse of broad-spectrum antibiotics, the underuse of diagnostic stewardship to guide antibiotic decisions and poor guideline compliance of antibiotic prescribing in the same hospitals, described in our recent paper, which identified several priority areas for stewardship.^17^

Based on the survey data, we considered that an important obstacle that hinders effective AMS implementation in this context could be linked to perceived ‘externality of AMR’ as a problem.^31^ That is, physicians acknowledge its significance but do not take ownership or responsibility, thus reflecting a production of AMR as an externality, for example, a result of irrational use elsewhere in communities or other hospitals. The lack of systematic surveillance of AMR and antibiotic use and the underutilisation of bacterial cultures, recognised by many of the respondents, also reinforces the perception of AMR as a ‘problem of elsewhere’. This feeds a lack of engaging with AMS, as physicians do not recognise it as a value-add for an already stretched institutional context, and in turn provides the context for continued defensive prescribing ‘to be on the safe side’. Moreover, defensive prescribing practices may somewhat offset (in the short term) problems around room cleaning, hand hygiene and staff education. In this way, AMR as an externality and the vulnerabilities of the institution, offer an environment conducive to the ongoing overuse of antimicrobials.^32 33^ The higher incidence of hospital-acquired infections in LMICs than in high-income countries could further promote defensive prescribing as a way to compensate for substandard IPC practices.^34^ All in all, this supports the notion that physicians tend to prioritise managing immediate clinical risks, reputation and concordance with peer practice, vis-à-vis the long-term population consequences of AMR.^35^

The identified differences between hospitals regarding awareness of AMS purposes, views regarding rational antibiotic prescribing, perception of AMR as a significant problem and immediate actions to contain AMR could not be attributed or related to the pertinent hospital sector (public vs private) or healthcare level (secondary vs tertiary), but they were found to be associated with several differences between the physician subgroups. First of all, work experience and medical hierarchy were found to influence the awareness of AMS purpose, AMR as a significant problem, and immediate actions to contain AMR. Interestingly, compared with junior physicians, specialists/consultants expressed lower confidence to make antibiotic decisions in uncertain situations while showing higher confidence in actions to contain AMR. Possible explanations include that specialists/consultants have a better recognition of the ‘unknowns’ (eg, lack of data on bacterial susceptibility patterns) and that they bear final patient responsibility, introducing the fear of losing a patient or legal consequences,^36^ whereas taking actions to curb AMR can be a remedy to compensate their fear. Conversely, residents’ higher confidence in antibiotic prescribing may also relate to their contemporary medical training, which promotes the use of evidence-based prescribing guidelines, as opposed to late-career physicians.^37^ GPs had low scores on views regarding rational antibiotic prescribing compared with consultants which could reflect the GPs’ limited responsibility in the antibiotic decision-making hierarchy, possibly leading to a lack of positive attitude towards guidelines and preference for complying with them.^38 39^

Furthermore, the acute specialties (including emergency, Intensive Care Unit (ICU) and anaesthesiology) had lower awareness of AMS activities and purposes, compared with surgery or medicine, but scored higher for immediate actions to contain AMR. Compared with surgeons, physicians in medicine had a greater awareness of AMS activities and recognition of AMR as a significant problem, but they had lower confidence in antibiotic prescribing decisions and immediate actions to contain AMR. These observations are in line with a UK study that found that emergency physicians experienced pressure for immediate action out of fear of losing a patient and a lack of ownership of antibiotic decision making due to the patient transitioning to inpatient care, that medical doctors adopted a more policy-informed, interdisciplinary approach and that senior surgeons left complex antibiotic decisions to junior staff, resulting in potential defensive and inappropriate antibiotic use.^19^ Variations in the social norms, values and behaviours between specialties should inform what is the best approach to antibiotic decision making.

Our study had several strengths and weaknesses. First, we were able to identify a relevant set of attributes through a factor analysis optimisation process, with adequate content, face and construct validity and internal reliability. In the absence of adequately validated instruments regarding AMR and AMS,^40^ this study adds important value to the field, with particular relevance and applicability for LMICs. Nonetheless, further questionnaire validation steps (such as criterion-related validity) are necessary to achieve a fully valid and reliable instrument. Second, the study had a large, varied respondent sample and high response rate. However, non-participation and the purposive and limited hospital sample could have introduced selection bias, and the data are not necessarily representative for Jakarta or Indonesia at large. The authenticity of the answers was maximised by protecting the respondents’ identities, although reliance on self-report has potential for social desirability bias. Third, this study focused on hospital settings. It is, however, acknowledged that the problem of AMR extends beyond hospital settings to the wider community through the primary healthcare system as well as informal providers, which warrants further study in the Indonesian context.^41^ Fourth, factor analysis is based on using a ‘heuristic’, which leaves room to more than one interpretation of the same data and cannot identify causality.

## Conclusion

AMS implementation in Indonesian hospitals is likely highly dependent on institutional, contextual and diagnostic vulnerabilities. These may result in the problem of AMR being externalised, instead of recognised as a local hospital problem. Current AMS strategies may be insufficiently successful in promoting prudent antibiotic use, due to lack of systematic engagement with and feedback to prescribers, aimed at building confidence in antibiotic decision making and ownership of the AMR problem. Appropriate recognition of the contextual and social determinants of antibiotic prescribing decision making, including hospital factors, dynamics in medical hierarchy and experience, among others, will be critical to design context-specific AMS interventions that are adopted by healthcare professionals and successfully influence behaviours.^14^

## Supplementary Material

Reviewer comments

Author's manuscript

## Data Availability

Data are available on reasonable request. The survey instrument is included in the data supplement. The data are deidentified survey participant data. Data are available on request, with written permission from the investigators. Requests can be submitted to the corresponding author.

## References

[1] O’Neill J. Tackling drug-resistant infections globally: final report and recommendations, 2016.

[2] Klein EY, Van Boeckel TP, Martinez EM, et al. Global increase and geographic convergence in antibiotic consumption between 2000 and 2015. Proc Natl Acad Sci U S A 2018;115:E3463–70. 10.1073/pnas.1717295115 29581252 PMC5899442

[3] Goossens H, Ferech M, Vander Stichele R, et al. Outpatient antibiotic use in Europe and association with resistance: a cross-national database study. Lancet 2005;365:579–87. 10.1016/S0140-6736(05)17907-0 15708101

[4] Chaw PS, Höpner J, Mikolajczyk R. The knowledge, attitude and practice of health practitioners towards antibiotic prescribing and resistance in developing countries-A systematic review. J Clin Pharm Ther 2018;43:606–13. 10.1111/jcpt.12730 29959783

[5] Md Rezal RS, Hassali MA, Alrasheedy AA, et al. Physicians' knowledge, perceptions and behaviour towards antibiotic prescribing: a systematic review of the literature. Expert Rev Anti Infect Ther 2015;13:665–80. 10.1586/14787210.2015.1025057 25813839

[6] Do NTT, Vu HTL, Nguyen CTK, et al. Community-Based antibiotic access and use in six low-income and middle-income countries: a mixed-method approach. Lancet Glob Health 2021;9:e610-e619. 10.1016/S2214-109X(21)00024-3 33713630 PMC8050200

[7] Ayobami O, Brinkwirth S, Eckmanns T, et al. Antibiotic resistance in hospital-acquired ESKAPE-E infections in low- and lower-middle-income countries: a systematic review and meta-analysis. Emerg Microbes Infect 2022;11:443–51. 10.1080/22221751.2022.2030196 35034585 PMC8820817

[8] Kim SH, Song J-H, Chung DR, et al. Changing trends in antimicrobial resistance and serotypes of Streptococcus pneumoniae isolates in Asian countries: an Asian network for surveillance of resistant pathogens (ANSORP) study. Antimicrob Agents Chemother 2012;56:1418–26. 10.1128/AAC.05658-11 22232285 PMC3294909

[9] Schuts EC, Hulscher MEJL, Mouton JW, et al. Current evidence on hospital antimicrobial stewardship objectives: a systematic review and meta-analysis. Lancet Infect Dis 2016;16:847–56. 10.1016/S1473-3099(16)00065-7 26947617

[10] Honda H, Ohmagari N, Tokuda Y, et al. Antimicrobial stewardship in inpatient settings in the Asia Pacific region: a systematic review and meta-analysis. Clin Infect Dis 2017;64:S119–26. 10.1093/cid/cix017 28475777

[11] Laundy M, Gilchrist M, Whitney L. Antimicrobial stewardship. Oxford, UK: Oxford University Press, 2016.

[12] Rolfe R, Kwobah C, Muro F, et al. Barriers to implementing antimicrobial stewardship programs in three low- and middle-income country tertiary care settings: findings from a multi-site qualitative study. Antimicrob Resist Infect Control 2021;10:60. 10.1186/s13756-021-00929-4 33766135 PMC7993456

[13] Teixeira Rodrigues A, Roque F, Falcão A, et al. Understanding physician antibiotic prescribing behaviour: a systematic review of qualitative studies. Int J Antimicrob Agents 2013;41:203–12. 10.1016/j.ijantimicag.2012.09.003 23127482

[14] Aveling E-L, Martin G, Armstrong N, et al. Quality improvement through clinical communities: eight lessons for practice. J Health Organ Manag 2012;26:158–74. 10.1108/14777261211230754 22856174

[15] Parathon H, Kuntaman K, Widiastoety TH, et al. Progress towards antimicrobial resistance containment and control in Indonesia. BMJ 2017;358:j3808–5. 10.1136/bmj.j3808 28874346 PMC5598291

[16] Herawati F, Ananta SC, Parwitha IAA, et al. Interview-Based cross-sectional needs assessment to advance the implementation of an effective antibiotic stewardship program in Indonesian hospitals. Health Policy Open 2020;1:100002. 10.1016/j.hpopen.2019.100002 PMC10297807 37383308

[17] Limato R, Nelwan EJ, Mudia M, et al. A multicentre point prevalence survey of patterns and quality of antibiotic prescribing in Indonesian hospitals. JAC Antimicrob Resist 2021;3:dlab047. 10.1093/jacamr/dlab047 33937773 PMC8072040

[18] Badan Pusat Statistik Provinsi DKI Jakarta (BPS-Statistics of DKI Jakarta Province). Jumlah Rumah Sakit DAN Tempat Tidur Yang Tersedia Menurut Kabupaten/Kota Administrasi DAN status Rumah Sakit di Provinsi DKI Jakarta 2018-2020. Badan pus. STAT. Provinsi DKI Jakarta, 2021. Available: https://jakarta.bps.go.id/indicator/30/539/1/jumlah-rumah-sakit-dan-tempat-tidur-yang-tersedia-menurut-kabupaten-kota-administrasi-dan-status-rumah-sakit-di-provinsi-dki-jakarta.html [Accessed 20 Dec 2021].

[19] Charani E, Ahmad R, Rawson TM, et al. The differences in antibiotic decision-making between acute surgical and acute medical teams: an ethnographic study of culture and team dynamics. Clin Infect Dis 2019;69:12–20. 10.1093/cid/ciy844 30445453 PMC6579961

[20] Greater New York Association United Hospital Fund. Antimicrobial stewardship toolkit: best practices from the GNYHA/UHF antimicrobial stewardship collaborative, 2011. Available: https://uhfnyc.org/media/filer_public/4a/49/4a497bf9-d0a1-44c0-8849-874af1bb6019/antimicrobial_stewardship.pdf

[21] Chaw PS, Schlinkmann KM, Raupach-Rosin H, et al. Knowledge, attitude and practice of Gambian health practitioners towards antibiotic prescribing and microbiological testing: a cross-sectional survey. Trans R Soc Trop Med Hyg 2017;111:117–24. 10.1093/trstmh/trx027 28633334

[22] Asante KP, Boamah EA, Abdulai MA, et al. Knowledge of antibiotic resistance and antibiotic prescription practices among prescribers in the Brong Ahafo region of Ghana; a cross-sectional study. BMC Health Serv Res 2017;17:422. 10.1186/s12913-017-2365-2 28633631 PMC5477684

[23] Labi A-K, Obeng-Nkrumah N, Bjerrum S, et al. Physicians' knowledge, attitudes, and perceptions concerning antibiotic resistance: a survey in a Ghanaian tertiary care hospital. BMC Health Serv Res 2018;18:126. 10.1186/s12913-018-2899-y 29458432 PMC5819203

[24] Venugopalan V, Trustman N, Manning N, et al. Administration of a survey to evaluate the attitudes of house staff physicians towards antimicrobial resistance and the antimicrobial stewardship programme at a community teaching hospital. J Glob Antimicrob Resist 2016;4:21–7. 10.1016/j.jgar.2016.01.004 27436388

[25] García C, Llamocca LP, García K, et al. Knowledge, attitudes and practice survey about antimicrobial resistance and prescribing among physicians in a hospital setting in Lima, Peru. BMC Clin Pharmacol 2011;11:18. 10.1186/1472-6904-11-18 22085536 PMC3231801

[26] Alumran A, Hou X-Y, Hurst C. Validity and reliability of instruments designed to measure factors influencing the overuse of antibiotics. J Infect Public Health 2012;5:221–32. 10.1016/j.jiph.2012.03.003 22632596

[27] Alothman A, Algwizani A, Alsulaiman M, et al. Knowledge and attitude of physicians toward prescribing antibiotics and the risk of resistance in two reference hospitals. Infect Dis 2016;9:IDRT.S40047. 10.4137/IDRT.S40047 PMC4941866 27429557

[28] Charoenboon N, Haenssgen MJ, Warapikuptanun P, et al. Translating antimicrobial resistance: a case study of context and consequences of antibiotic-related communication in three Northern Thai villages. Palgrave Commun 2019;5:23. 10.1057/s41599-019-0226-9

[29] Chandler CI, Hutchinson E, Hutchison C. Addressing antimicrobial resistance through social theory: an Anthropologically oriented report. London School of Hygiene & Tropical Medicine, 2016.

[30] Broom A, Doron A. Antimicrobial resistance, politics, and practice in India. Qual Health Res 2020;30:1684–96. 10.1177/1049732320919088 32458726

[31] Broom A, Broom J, Kirby E, et al. Antibiotic optimisation in 'the bush': Local know-how and core-periphery relations. Health Place 2017;48:56–62. 10.1016/j.healthplace.2017.09.003 28941843

[32] Broom A, Kirby E, Gibson AF, et al. Myth, manners, and medical ritual: defensive medicine and the Fetish of antibiotics. Qual Health Res 2017;27:1994–2005. 10.1177/1049732317721478 28737082

[33] Will CM. Editorial: beyond behavior? institutions, interactions and inequalities in the response to antimicrobial resistance. Sociol Health Illn 2018;40:E1–9. 10.1111/1467-9566.12735 29574948

[34] Chandler CIR. Current accounts of antimicrobial resistance: stabilisation, individualisation and antibiotics as infrastructure. Palgrave Commun 2019;5:53. 10.1057/s41599-019-0263-4 PMC6542671 31157116

[35] Broom A, Broom J, Kirby E. Cultures of resistance? A Bourdieusian analysis of doctors' antibiotic prescribing. Soc Sci Med 2014;110:81–8. 10.1016/j.socscimed.2014.03.030 24727665

[36] Broom A, Broom J, Kirby E, et al. The social dynamics of antibiotic use in an Australian hospital. J Sociol 2016;52:824–39. 10.1177/1440783315594486

[37] Fernandez-Lazaro CI, Brown KA, Langford BJ, et al. Late-career physicians prescribe longer courses of antibiotics. Clin Infect Dis 2019;69:1467–75. 10.1093/cid/ciy1130 30615108

[38] R Hansen C, Bradley CP, Sahm LJ. Factors influencing successful prescribing by intern doctors: a qualitative systematic review. Pharmacy 2016;4:24. 10.3390/pharmacy4030024 PMC5419364 28970397

[39] Roumie CL, Halasa NB, Edwards KM, et al. Differences in antibiotic prescribing among physicians, residents, and nonphysician clinicians. Am J Med 2005;118:641–8. 10.1016/j.amjmed.2005.02.013 15922696

[40] DeVon HA, Block ME, Moyle-Wright P, et al. A psychometric toolbox for testing validity and reliability. J Nurs Scholarsh 2007;39:155–64. 10.1111/j.1547-5069.2007.00161.x 17535316

[41] Frost I, Laxminarayan R, McKenna N, et al. Antimicrobial resistance and primary health care. World Heal Organ 2018:3–6.

