# Supplementary material for: Perceptions, views and practices regarding antibiotic prescribing and stewardship among hospital physicians in Jakarta, Indonesia: a questionnaire-based survey
Source: BMJ Open. 2022 May 18;12(5):e054768. doi: 10.1136/bmjopen-2021-054768 (PMC9121411; doi:10.1136/bmjopen-2021-054768)
Supplement: Supplementary data [file bmjopen-2021-054768supp001.pdf]

Appendix: Survey questionnaire

We kindly request you to indicate your agreement with the following 40 statements using a five-point scale: ① Strongly Disagree ② Disagree ③ Neutral or Don't Know ④ Agree ⑤ Strongly Agree						
**ANTIMICROBIAL RESISTANCE: SCOPE OF THE PROBLEM AND KEY CONTRIBUTORS**						
**1.**	Antimicrobial resistance is a significant problem in this hospital	①	②	③	④	⑤
**2.**	Antimicrobial resistance is a significant problem in Indonesia	①	②	③	④	⑤
**3.**	A cause of antimicrobial resistance is using too many antimicrobial drugs	①	②	③	④	⑤
**4.**	Lack of hand disinfection by healthcare workers causes spread of antimicrobial resistance	①	②	③	④	⑤
**5.**	Use of broad-spectrum antibiotics can increase antimicrobial resistance when narrower-spectrum antibiotics are available that are equally effective	①	②	③	④	⑤
**6.**	Antibiotic resistance is also a problem outside of the hospital, in communities	①	②	③	④	⑤
**7.**	In this hospital, patient rooms are cleaned according to hospital cleaning protocol once a patient with a multidrug-resistant organism (MDRO) has been discharged	①	②	③	④	⑤
**8.**	Adherence to hand-hygiene protocols is excellent at this hospital	①	②	③	④	⑤
**9.**	This hospital does NOT provide adequate staff education regarding multidrug-resistant organisms	①	②	③	④	⑤
**10.**	A patient is likely to develop an infection with a multidrug-resistant organism during their stay at this hospital	①	②	③	④	⑤
**ANTIBIOTIC PRESCRIBING PRACTICES**						
**11.**	Antibiotics are overused in Indonesia	①	②	③	④	⑤
**12.**	Antibiotics are overused in this hospital	①	②	③	④	⑤
**13.**	Microbiology laboratory results are efficiently communicated to the treating physician	①	②	③	④	⑤
**14.**	I regularly refer to/consider the antibiotic susceptibility patterns at this hospital/institution (i.e. the institutional antibiogram) when empirically prescribing antibiotics	①	②	③	④	⑤
**15.**	If medically appropriate, intravenous antibiotics should be stepped down to an oral alternative after three days	①	②	③	④	⑤
**16.**	Restrictions on antibiotics impair my ability to provide good patient care	①	②	③	④	⑤
**17.**	More judicious use of antibiotics would decrease antimicrobial resistance	①	②	③	④	⑤
**18.**	Following evidence-based antibiotic guidelines will help optimize treatment outcomes	①	②	③	④	⑤
**19.**	In general, rational antibiotic prescribing for my patients is high on my list of priorities	①	②	③	④	⑤
**20.**	Developing hospital antibiotic guidelines is more useful than applying international guidelines	①	②	③	④	⑤
**21.**	I am often unsure if a patient needs an antibiotic or not	①	②	③	④	⑤

**22.**	I am often unsure which antibiotic to prescribe	①	②	③	④	⑤
**23.**	I will stop antibiotics that others have prescribed in the absence of an appropriate indication	①	②	③	④	⑤
**24.**	Patients with high fever (≥39°C) must be treated with antibiotics	①	②	③	④	⑤
**25.**	If I am uncertain about the diagnosis of infection, but think it is possible, I feel safer prescribing an antibiotic	①	②	③	④	⑤
**26.**	Fear of patient deterioration or complications leads me to prescribe antibiotics more freely	①	②	③	④	⑤
**27.**	I frequently prescribe antibiotics because patients or their relatives insist on it	①	②	③	④	⑤
**ANTIMICROBIAL STEWARDSHIP PROGRAM (ASP)**<br>***A formal program formal that monitors and manages the appropiate use of antibiotics***						
**28.**	I am aware that my hospital has an antimicrobial stewardship program (ASP)	①	②	③	④	⑤
**29.**	I understand what the purpose of ASP is	①	②	③	④	⑤
**30.**	ASP improve patient care	①	②	③	④	⑤
**31.**	ASP reduces the problem of antimicrobial resistance	①	②	③	④	⑤
**32.**	ASP reduces this hospital’s infection rates	①	②	③	④	⑤
**33.**	Additional staff education on antimicrobial prescribing is needed	①	②	③	④	⑤
**34.**	Regular audit and feedback encourage me to prescribe antibiotics prudently	①	②	③	④	⑤
**35.**	Rapid and accurate diagnostic tests are useful for diagnosis of infectious diseases and guidance on antibiotic therapy	①	②	③	④	⑤
**36.**	To reduce antibiotic overuse in hospitals, implementation of antibiotic restriction (e.g., antibiotic tiers) is a useful measure	①	②	③	④	⑤
**37.**	To curb antimicrobial resistance, regular consultations or ward rounds with a clinical microbiologist or infectious disease physician are useful	①	②	③	④	⑤
**38.**	To curb antimicrobial resistance, doctors need to have timely access to microbiological test results to guide antibiotic therapy	①	②	③	④	⑤
**39.**	Up-to-date information on hospital antimicrobial resistance patterns is important for developing hospital antibiotic guidelines	①	②	③	④	⑤
**40.**	Effective infection prevention and control in the hospital reduces antimicrobial resistance	①	②	③	④	⑤

BACKGROUND INFORMATION

**41.**	What is your primary work unit in this hospital? **Tick ONE**			
	○ Many different unit/not specific<br>○ Emergency department<br>○ Surgery<br>○ Anaesthesiology<br>○ Intensive Care Unit (ALL)	○ OBGYN (Obstetrics/ Gynaecology)<br>○ Internal Medicine<br>○ Neurology<br>○ Paediatrics<br>○ Psychiatrics<br>○ ENT (Ear Nose Throat)	○ Eye<br>○ Dermato-venerology<br>○ Pulmonology<br>○ Cardiology<br>○ Orthopaedics<br>○ Radiology	○ Rehabilitation<br>○ Pharmacy<br>○ Microbiology<br>○ Laboratory<br>○ Other ____________

**42.**	How long have you worked in this hospital? **Tick ONE**		
	○ Less than 1 year<br>○ 1 – 5 years	○ 6 – 10 years<br>○ 11 – 15 years	○ 16 - 20 years<br>○ More than 20 years
**43.**	What is your position in this hospital? **Tick ONE**		
	○ Internship doctor<br>○ General Practitioner	○ Resident<br>○ Specialist	○ Other<br>___________________
**44.**	How long have you worked in your current specialty or profession? **Tick ONE**		
	○ Less than 1 year<br>○ 1 – 5 years	○ 6 – 10 years<br>○ 11 – 15 years	○ 16 - 20 years<br>○ More than 20 years
**45.**	Which of the following resources do you use to guide your antibiotic prescribing? **Tick ALL that apply**		
	○ Consultation with senior colleague(s)<br>○ Consultation with specialist in microbiology/ infectious disease<br>○ Textbooks	Guideline<br>☐ Internasional<br>☐ Nasional<br>☐ Hospital<br>☐ Departement/ division	○ Medical journal<br>○ Phamaceutical representative ______________________________<br>○ Internet _______________________<br>○ Other _________________________
**46.**	During the past year, how many times have you received training/teaching or attended seminars/courses on antimicrobial prescribing, resistance and/or stewardship? _____ times		
**47.**	What is your sex: ○ Male ○ Female		
**48.**	I would like to take part in the raffle: ○ yes ○ no If yes, my email is:__________________		

Thank you for completing this survey!
