# Supplementary material for: Perceptions, views and practices regarding antibiotic prescribing and stewardship among hospital physicians in Jakarta, Indonesia: a questionnaire-based survey
Source: BMJ Open. 2022 May 18;12(5):e054768. doi: 10.1136/bmjopen-2021-054768 (PMC9121411; doi:10.1136/bmjopen-2021-054768)
Supplement: Supplementary data [file bmjopen-2021-054768supp002.pdf]

Table S1 Survey response rate, overall and by hospital, department and professional hierarchy

	Total physicians invited	Total physicians responded	Response rate (%)	Respondents per physician type				
				Specialist/ Consultant	Resident	General practitioner	Intern/Internship doctor	Other
**Total**	**1896**	**1007**	**53.1%**	**358 (35.6%)**	**500 (49.7%)**	**113 (11.2%)**	**10 (1.0%)**	**18 (1.8%)**
**Hospital** [a]								
01	101 (5.3%)	70 (7.0%)	69.3%	39 (10.9%)	0 (0.0%)	27 (23.9%)	0 (0.0%)	4 (22.2%)
02	255 (13.4%)	155 (15.4%)	60.8%	70 (19.6%)	52 (10.4%)	26 (23.0%)	0 (0.0%)	5 (27.8%)
03	143 (7.5%)	77 (7.7%)	53.9%	41 (11.5%)	0 (0.0%)	35 (31.0%)	0 (0.0%)	1 (5.6%)
04	55 (2.9%)	44 (4.4%)	80.0%	29 (8.1%)	0 (0.0%)	11 (9.7%)	0 (0.0%)	1 (5.6%)
05	1240 (65.4%)	622 (61.8%)	50.2%	152 (42.5%)	448 (89.6%)	4 (3.5%)	10 (100.0%)	5 (27.8%)
06	102 (5.4%)	39 (3.9%)	38.2%	27 (7.5%)	0 (0.0%)	10 (8.9%)	0 (0.0%)	2 (11.1%)
**Sector** [b]								
Private	346	186 (18.5%)	53.8%	107 (29.9%)	0 (0.0%)	0 (0.0%)	72 (63.7%)	7 (38.9%)
Public	1550	821 (81.5%)	53.0%	251 (70.1%)	500 (100.0%)	41 (36.3%)	10 (100.0%)	11 (61.1%)
**Level of care** [c]								
Secondary	1499	777 (77.2%)	51.8%	136 (38.0%)	0 (0.0%)	83 (73.5%)	0 (0.0%)	8 (44.4%)
Tertiary	397	230 (22.8%)	57.9%	222 (62.0%)	500 (100.0%)	30 (26.6%)	10 (100.0%)	10 (55.6%)
**Department** [d]								
Surgery and surgical subspecialties	***628***	***371 (36.8)***	***59.1%***	***91 (24.5%)***	***226 (60.9%)***	***1 (0.27%)***	***4 (1.1%)***	***0 (0.0%)***
Obstetrics/gynaecology	229	146 (14.5%)	63.8%	51 (14.3%)	91 (18.2%)	1 (0.88%)	1 (10.0%)	0 (0.0%)
Surgery	210	122 (12.1%)	58.1%	2 (11.1%)	74 (14.8%)	0 (0.0%)	3 (30.0%)	0 (0.0%)
Orthopaedics	72	57 (5.7%)	44.4%	25 (7.0%)	32 (6.4%)	0 (0.0%)	0 (0.0%)	0 (0.0%)
Ear-nose-throat	74	32 (3.2%)	77.0%	12 (3.4%)	19 (3.8%)	0 (0.0%)	0 (0.0%)	0 (0.0%)
Urology	43	14 (1.4%)	32.6%	1 (1.1%)	10 (2.0%)	0 (0.0%)	0 (0.0%)	0 (0.0%)
Medicine and medical subspecialties	***456***	***232 (23.0%)***	***50.9%***	***115 (49.6%)***	***109 (47.0%)***	***0 (0.0%)***	***3 (1.3%)***	***5 (2.2%)***
Internal medicine	261	128 (12.7%)	49.0%	59 (16.5%)	67 (13.4%)	0 (0.0%)	0 (0.0%)	2 (11.1%)
Neurology	74	63 (6.3%)	85.1%	17 (4.8%)	40 (8.0%)	0 (0.0%)	3 (30.0%)	3 (16.7%)
Pulmonology	21	15 (1.5%)	71.4%	15 (4.2%)	0 (0.0%)	0 (0.0%)	0 (0.0%)	0 (0.0%)
Dermato-venerology	77	14 (1.4%)	18.2%	12 (0.0%)	2 (3.4%)	0 (0.0%)	0 (0.0%)	0 (0.0%)
Cardiology	23	12 (1.2%)	52.2%	12 (3.4%)	0 (0.0%)	0 (0.0%)	0 (0.0%)	0 (0.0%)
Acute specialties [e]	***96***	***156 (15.5)***	***162.5%***	***28 (17.9%)***	***67 (42.9%)***	***68 (43.6%)***	***2 (1.3%)***	***1 (0.64%)***
Anaesthesiology	96	72 (7.2%)	75.0%	4 (1.1%)	66 (13.2%)	0 (0.0%)	2 (20.0%)	0 (0.0%)

	Total physicians invited	Total physicians responded	Response rate (%)	Respondents per physician type				
				Specialist/ Consultant	Resident	General practitioner	Intern/Internship doctor	Other
Emergency dept [e]	-	57 (5.7%)	-	0 (0.0%)	0 (0.0%)	57 (50.4%)	0 (0.0%)	0 (0.0%)
Intensive Care Unit [e]	-	27 (2.7%)	-	14 (3.9%)	1 (0.2%)	11 (9.7%)	0 (0.0%)	1 (5.6%)
Other departments	***539***	***244 (24.2)***	***45.2%***	***90 (36.9%)***	***98 (40.2%)***	***24 (9.8%)***	***1 (0.41%)***	***10 (4.1%)***
Paediatrics	202	54 (5.4%)	26.7%	28 (7.8%)	25 (5.0%)	0 (0.0%)	0 (0.0%)	0 (0.0%)
Ophthalmology	102	39 (3.9%)	38.2%	22 (6.2%)	17 (3.4%)	0 (0.0%)	0 (0.0%)	0 (0.0%)
Multiple/unspecified units [e]	-	33 (3.3%)	-	5 (1.4%)	5 (1.0%)	21 (18.6%)	1 (10.0%)	0 (0.0%)
Rehabilitation	70	32 (3.2%)	45.7%	4 (1.1%)	28 (5.6%)	0 (0.0%)	0 (0.0%)	0 (0.0%)
Psychiatry	54	30 (3.0%)	55.6%	11 (3.1%)	19 (3.8%)	0 (0.0%)	0 (0.0%)	0 (0.0%)
Dentist	49	27 (2.7%)	55.1%	11 (3.1%)	1 (0.20%)	3 (2.7%)	0 (0.0%)	10 (55.6%)
Other	39	25 (2.5%)	64.1%	4 (1.1%)	1 (0.20%)	20 (17.7%)	0 (0.0%)	0 (0.0%)
Microbiology	4	3 (0.30%)	75.0%	2 (0.56%)	1 (0.20%)	0 (0.0%)	0 (0.0%)	0 (0.0%)
Laboratory	19	1 (0.10%)	5.3%	1 (0.28%)	0 (0.0%)	0 (0.0%)	0 (0.0%)	0 (0.0%)
Missing	-	4 (0.40%)	-	2 (0.56%)	1 (0.20%)	0 (0.0%)	0 (0.0%)	0 (0.0%)

Data are reported as n (%).
Doctor type was missing for 8 respondents, across [a] hospitals (02 (2), 03 (3), and 05 (3)); [b] sector (public 8, private 0); [c] level of care (secondary 3, tertiary 5); and [d] departments (obstetrics/gynaecology 2; ENT 1; paediatrics 1; multiple units 1; dental/oral surgery 2; missing dept 1).
[e] Invitations to participate in the survey were sent out according to primary work units; the discrepancies reported for the acute and other departments result from the fact that n=177 respondents who worked across multiple units (mostly GPs and internship doctors) indicated a different primary department on their questionnaire.

Table S2. Five-point Likert scale responses for the 40-item questionnaire

Item #	No of respondents	Strongly disagree (1)	Disagree (2)	Neutral (3)	Agree (4)	Strongly agree (5)
Q1	1005	3 (0.30)	26 (2.6)	168 (16.7)	452 (45.0)	356 (35.4)
Q2	1006	0 (0.0)	7 (0.70)	55 (5.5)	505 (50.2)	439 (43.6)
Q3	1003	0 (0.0)	6 (0.60)	43 (4.3)	472 (47.1)	482 (48.1)
Q4	1005	19 (1.9)	98 (9.8)	173 (17.2)	497 (49.5)	218 (21.7)
Q5	1004	2 (0.20)	49 (4.9)	145 (14.4)	514 (51.2)	294 (29.3)
Q6	1003	0 (0.0)	29 (2.9)	136 (13.6)	516 (51.5)	322 (32.1)
Q7	1004	12 (1.2)	53 (5.3)	288 (28.7)	460 (45.8)	191 (19.0)
Q8	1002	6 (0.60)	81 (8.1)	345 (34.4)	444 (44.3)	126 (12.6)
Q9	1005	71 (7.1)	397 (39.5)	311 (31.0)	188 (18.7)	38 (3.8)
Q10	1005	32 (3.2)	299 (29.8)	363 (36.1)	256 (25.5)	55 (5.5)
Q11	1005	8 (0.80)	34 (3.4)	102 (10.2)	506 (50.4)	355 (35.3)
Q12	1005	18 (1.8)	213 (21.2)	417 (41.5)	286 (28.5)	71 (7.1)
Q13	1005	11 (1.1)	97 (9.7)	321 (31.9)	515 (51.2)	61 (6.1)
Q14	1005	12 (1.2)	121 (12.0)	324 (32.2)	468 (46.6)	80 (8.0)
Q15	1006	7 (0.70)	73 (7.3)	195 (19.4)	623 (61.9)	108 (10.7)
Q16	1006	67 (6.7)	334 (33.2)	267 (26.5)	276 (27.4)	62 (6.2)
Q17	1005	2 (0.20)	2 (0.20)	48 (4.8)	431 (42.9)	522 (51.9)
Q18	1006	0 (0.0)	6 (0.60)	42 (4.2)	467 (46.4)	491 (48.8)
Q19	1005	1 (0.10)	7 (0.70)	105 (10.5)	545 (54.3)	347 (34.5)
Q20	1004	3 (0.30)	34 (3.4)	180 (17.9)	511 (50.9)	276 (27.5)
Q21	1006	60 (6.0)	440 (43.7)	269 (26.7)	204 (20.3)	33 (3.3)
Q22	1006	89 (8.9)	448 (44.5)	254 (25.3)	196 (19.5)	19 (1.9)
Q23	1002	19 (1.9)	124 (12.4)	288 (28.7)	464 (46.3)	107 (10.7)
Q24	1005	213 (21.2)	480 (47.8)	189 (18.8)	104 (10.4)	19 (1.9)
Q25	1005	51 (5.1)	285 (28.4)	301 (30.0)	348 (34.6)	20 (2.0)
Q26	1006	55 (5.5)	354 (35.2)	245 (24.4)	330 (32.8)	22 (2.2)
Q27	1005	231 (22.3)	538 (53.5)	138 (13.7)	90 (9.0)	8 (0.80)
Q28	1006	0 (0.0)	15 (1.5)	54 (5.4)	526 (52.3)	411 (40.9)
Q29	1006	2 (0.20)	6 (0.60)	71 (7.1)	585 (58.2)	342 (34.0)
Q30	1006	7 (0.70)	11 (1.1)	97 (9.6)	582 (57.9)	309 (30.7)
Q31	1005	2 (0.20)	7 (0.70)	109 (10.89)	620 (61.7)	267 (26.6)

Item #	No of respondents	Strongly disagree (1)	Disagree (2)	Neutral (3)	Agree (4)	Strongly agree (5)
Q32	1004	3 (0.30)	25 (2.5)	206 (20.5)	568 (56.6)	202 (20.1)
Q33	1005	2 (0.20)	14 (1.4)	101 (10.1)	574 (57.1)	314 (31.2)
Q34	1006	1 (0.10)	7 (0.70)	70 (7.0)	702 (69.8)	226 (22.5)
Q35	1006	0 (0.0)	4 (0.40)	31 (3.1)	646 (64.2)	325 (32.3)
Q36	1007	1 (0.10)	13 (1.3)	83 (8.2)	675 (67.0)	235 (23.3)
Q37	1006	2 (0.20)	17 (1.7)	128 (12.7)	657 (65.3)	202 (20.1)
Q38	1006	0 (0.0)	10 (0.99)	66 (6.6)	616 (61.2)	314 (31.2)
Q39	1006	0 (0.0)	4 (0.40)	49 (4.9)	617 (61.3)	336 (33.4)
Q40	1006	0 (0.0)	4 (0.40)	43 (4.3)	612 (60.8)	347 (34.5)

Data are also shown in Figure 1.

Table S3 The latent factors of antibiotic prescribing (full table)

Factor	Factor label	Survey questions included in the factor loading	Loadings	Reliability (Cronbach's α)
**1**	Awareness of AMS activities	Q33: Additional staff education on antimicrobial prescribing is needed	0.5202	0.8734
		Q34: Regular audit and feedback encourage me to prescribe antibiotics prudently	0.6151	
		Q35: Rapid and accurate diagnostic tests are useful for diagnosis of infectious diseases and guidance on antibiotic therapy	0.6714	
		Q36: To reduce antibiotic overuse in hospitals, implementation of antibiotic restriction (e.g. antibiotic tiers) is a useful measure	0.6428	
		Q37: To curb antimicrobial resistance, regular consultations or ward rounds with a clinical microbiologist or infectious disease physician are useful	0.7046	
		Q38: To curb antimicrobial resistance, doctors need to have timely access to microbiological test results to guide antibiotic therapy	0.7197	
		Q39: Up-to-date information on hospital antimicrobial resistance patterns is important for developing hospital antibiotic guidelines	0.7374	
		Q40: Effective infection prevention and control in the hospital reduces antimicrobial resistance	0.7067	
**2**	Awareness of AMS purposes	Q28: I am aware that my hospital has an antimicrobial stewardship program (ASP)	0.6224	0.8334
		Q29: I understand what the purpose of ASP is	0.6957	
		Q30: ASP improve patient care	0.7744	
		Q31: ASP reduces the problem of antimicrobial resistance	0.7532	
		Q32: ASP reduces this hospital's infection rates	0.6670	
**3**	Views regarding rational antibiotic prescribing	Q11: Antibiotics are overused in Indonesia	0.5138	0.6961
		Q17: More judicious use of antibiotics would decrease antimicrobial resistance	0.7362	
		Q18: Following evidence-based antibiotic guidelines will help optimize treatment outcomes	0.6565	
		Q19: In general, rational antibiotic prescribing for my patients is high on my list of priorities	0.5246	
		Q20: Developing hospital antibiotic guidelines is more useful than applying international guidelines	0.3499	
**4**	Confidence in antibiotic prescribing decisions	Q12: Antibiotics are overused in this hospital	-0.4550	0.6997
		Q16: Restrictions on antibiotics impair my ability to provide good patient care	0.4031	
		Q21: I am often unsure if a patient needs an antibiotic or not	0.5640	
		Q22: I am often unsure which antibiotic to prescribe	0.5670	
		Q24: Patients with high fever (≥39°C) must be treated with antibiotics	0.4794	
		Q25: If I am uncertain about the diagnosis of infection, but think it is possible, I feel	0.6741	

Factor	Factor label	Survey questions included in the factor loading	Loadings	Reliability (Cronbach's α)
		safer prescribing an antibiotic		
		Q26: Fear of patient deterioration or complications leads me to prescribe antibiotics more freely	0.7092	
		Q27: I frequently prescribe antibiotics because patients or their relatives insist on it	0.6318	
**5**	Perception of AMR as a significant problem	Q1: Antimicrobial resistance is a significant problem in this hospital	0.5701	0.6967
		Q2: Antimicrobial resistance is a significant problem in Indonesia	0.5742	
		Q3: A cause of antimicrobial resistance is using too many antimicrobial drugs	0.5361	
		Q4: Lack of hand disinfection by healthcare workers causes spread of antimicrobial resistance	0.5725	
		Q5: Use of broad-spectrum antibiotics can increase antimicrobial resistance when narrower-spectrum antibiotics are available that are equally effective	0.5480	
		Q6: Antibiotic resistance is also a problem outside of the hospital, in communities	0.4766	
**6**	Immediate actions to contain AMR	Q7: In this hospital, patient rooms are cleaned according to hospital cleaning protocol once a patient with a multidrug-resistant organism (MDRO) has been discharged	0.6058	0.5695
		Q8: Adherence to hand-hygiene protocols is excellent at this hospital	0.6368	
		Q13: Microbiology laboratory results are efficiently communicated to the treating physician	0.5116	
		Q14: I regularly refer to/consider the antibiotic susceptibility patterns at this hospital/institution (i.e. the institutional antibiogram) when empirically prescribing antibiotics	0.6115	
		Q15: If medically appropriate, intravenous antibiotics should be stepped down to an oral alternative after three days	0.3617	
		Q23: I will stop antibiotics that others have prescribed in the absence of an appropriate indication	0.3892	

Abbreviations: AMS, antibiotic stewardship; ASP, antibiotic stewardship programme; IPC, infection prevention and control
Data are summarised in Table 3
Item #9 and 10 were excluded from the analysis, as explained in Results.

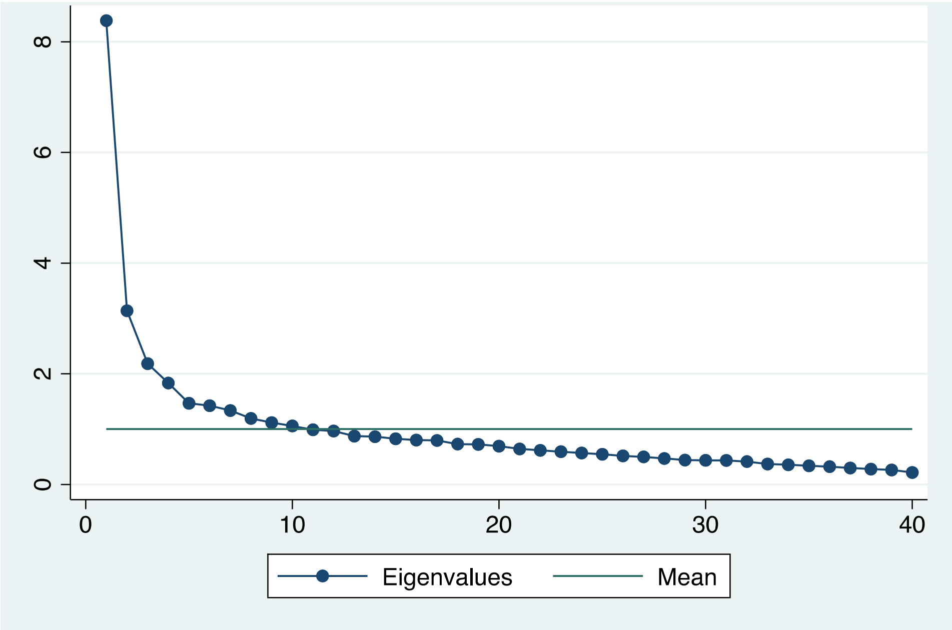

Figure S1 Scree plot showing eigenvalues for the 40 factors

Figure S2 Parallel analysis showing adjusted eigenvalues for the 40 factors.

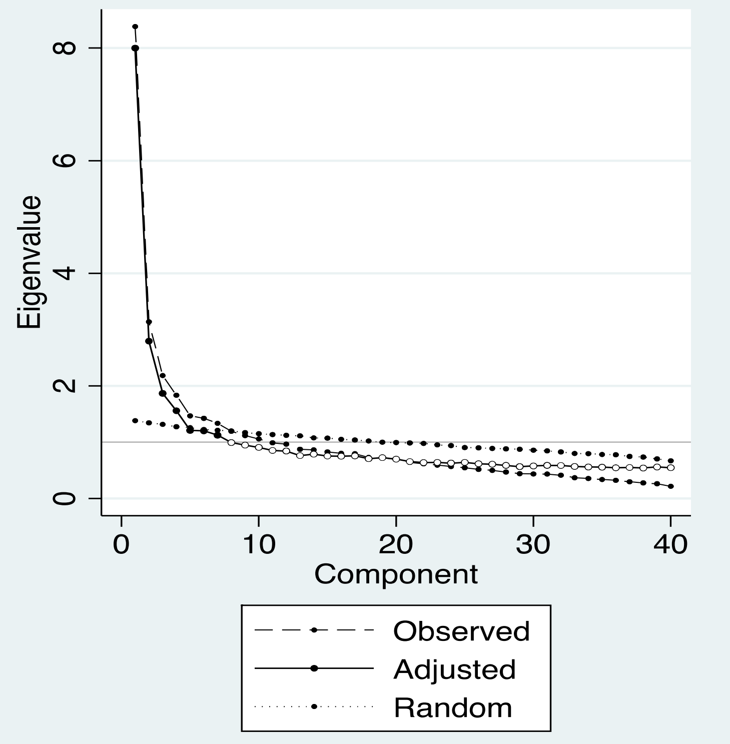

Parallel analysis adjusted the original eigenvalues for sampling error-induced collinearity among the variables to arrive at the adjusted eigenvalues.
