# Supplementary material for: Perceptions, views and practices regarding antibiotic prescribing and stewardship among hospital physicians in Jakarta, Indonesia: a questionnaire-based survey
Source: BMJ Open. 2022 May 18;12(5):e054768. doi: 10.1136/bmjopen-2021-054768 (PMC9121411; doi:10.1136/bmjopen-2021-054768)
Supplement: Author's manuscript [file bmjopen-2021-054768.draft_revisions.pdf]

BMJ Open

BMJ Open is committed to open peer review. As part of this commitment we make the peer review history of every article we publish publicly available.

When an article is published we post the peer reviewers' comments and the authors' responses online. We also post the versions of the paper that were used during peer review. These are the versions that the peer review comments apply to.

The versions of the paper that follow are the versions that were submitted during the peer review process. They are not the versions of record or the final published versions. They should not be cited or distributed as the published version of this manuscript.

BMJ Open is an open access journal and the full, final, typeset and author-corrected version of record of the manuscript is available on our site with no access controls, subscription charges or pay-per-view fees (http://bmjopen.bmj.com).

If you have any questions on BMJ Open's open peer review process please email info.bmjopen@bmj.com

BMJ Open

Perceptions, views and practices regarding antibiotic prescribing and stewardship among hospital physicians in Jakarta, Indonesia

Journal:	*BMJ Open*
Manuscript ID	bmjopen-2021-054768
Article Type:	Original research
Date Submitted by the Author:	22-Jun-2021
Complete List of Authors:	Limato, Ralalicia; Eijkman-Oxford Clinical Research Unit; Centre for Tropical Medicine and Global Health, Nuffield Department of Medicine, University of Oxford<br>Nelwan, Erni Juwita ; Cipto Mangunkusumo National General Hospital, Department of Internal Medicine, Division of Infectious Diseases; Faculty of Medicine, Universitas Indonesia<br>Mudia, Manzilina; Eijkman-Oxford Clinical Research Unit<br>Alamanda, Monik; Eijkman-Oxford Clinical Research Unit<br>Manurung, Elfrida Rinawaty; Royal Taruma Hospital<br>Mauleti, Ifael Yerosias; Fatmawati General Hospital<br>Mayasari, Maria; St. Carolus Hospital<br>Firmansyah, Iman; Prof. Dr. Sulianti Saroso Infectious Disease Hospital<br>Djaafar , Roswin ; Metropolitan Medical Centre Hospital<br>Vu, Huong; Oxford University Clinical Research Unit Viet Nam, Hanoi Unit<br>van Doorn, H. Rogier; Oxford University Clinical Research Unit; Nuffield Department of Clinical Medicine<br>Broom, Alex; The University of Sydney, School of Social and Political Sciences, Faculty of Arts and Social Sciences<br>Hamers, Raph Leonardus; Eijkman-Oxford Clinical Research Unit; Centre for Tropical Medicine and Global Health, Nuffield Department of Medicine, University of Oxford
Keywords:	Public health < INFECTIOUS DISEASES, PUBLIC HEALTH, SOCIAL MEDICINE

SCHOLARONE™
Manuscripts

I, the Submitting Author has the right to grant and does grant on behalf of all authors of the Work (as defined in the below author licence), an exclusive licence and/or a non-exclusive licence for contributions from authors who are: i) UK Crown employees; ii) where BMJ has agreed a CC-BY licence shall apply, and/or iii) in accordance with the terms applicable for US Federal Government officers or employees acting as part of their official duties; on a worldwide, perpetual, irrevocable, royalty-free basis to BMJ Publishing Group Ltd ("BMJ") its licensees and where the relevant Journal is co-owned by BMJ to the co-owners of the Journal, to publish the Work in this journal and any other BMJ products and to exploit all rights, as set out in our licence.

The Submitting Author accepts and understands that any supply made under these terms is made by BMJ to the Submitting Author unless you are acting as an employee on behalf of your employer or a postgraduate student of an affiliated institution which is paying any applicable article publishing charge ("APC") for Open Access articles. Where the Submitting Author wishes to make the Work available on an Open Access basis (and intends to pay the relevant APC), the terms of reuse of such Open Access shall be governed by a Creative Commons licence – details of these licences and which Creative Commons licence will apply to this Work are set out in our licence referred to above.

Other than as permitted in any relevant BMJ Author's Self Archiving Policies, I confirm this Work has not been accepted for publication elsewhere, is not being considered for publication elsewhere and does not duplicate material already published. I confirm all authors consent to publication of this Work and authorise the granting of this licence.

For peer review only

Perceptions, views and practices regarding antibiotic prescribing and stewardship among hospital physicians in Jakarta, Indonesia

Authors

Ralalicia Limato[1,2], Erni J. Nelwan[3,4,5,6], Manzilina Mudia[1], Monik Alamanda[1], Elfrida R. Manurung[7], Ifael Y. Mauleti[8], Maria Mayasari[9], Iman Firmansyah[10], Roswin Djafar[6], Huong Vu Thi Lan[11], H. Rogier van Doorn[2,11], Alex Broom[12], Raph L. Hamers[1,2,4*]

Affiliations

1. Eijkman-Oxford Clinical Research Unit, Jakarta, Indonesia
2. Centre for Tropical Medicine and Global Health, Nuffield Department of Medicine, University of Oxford, Oxford, UK
3. Department of Internal Medicine, Division of Infectious Diseases, Cipto Mangunkusumo National General Hospital, Jakarta, Indonesia
4. Faculty of Medicine, Universitas Indonesia, Jakarta, Indonesia
5. Infectious Disease and Immunology Research Cluster, Indonesian Medical Education and Research Institute, Jakarta, Indonesia
6. Metropolitan Medical Centre Hospital, Jakarta, Indonesia
7. Royal Taruma Hospital, Jakarta, Indonesia
8. Fatmawati General Hospital, Jakarta, Indonesia
9. St. Carolus Hospital, Jakarta, Indonesia
10. Prof. Dr. Sulianti Saroso Infectious Disease Hospital, Jakarta, Indonesia
11. Oxford University Clinical Research Unit, Hanoi, Vietnam
12. School of Social and Political Sciences, The University of Sydney, Sydney, Australia

***Corresponding author**

Raph L. Hamers MD PhD

Email : raph.hamers@ndm.ox.ac.uk

Phone : +62 21 31900971

Fax : -

Inserts: Tables 4; Figures 1

Appendix: survey questionnaire

Data supplement: Tables 3; Figures 2

Abstract

Objectives: Antibiotic overuse is one of the main drivers of antimicrobial resistance (AMR), especially in low and middle-income countries. This study aimed to gain an understanding of perceptions, views, and practices regarding AMR, antibiotic prescribing, and stewardship (AMS) among hospital physicians in Jakarta, Indonesia.

Design: cross-sectional, self-administered questionnaire-based survey, with descriptive statistics, exploratory factor analysis (EFA) to identify distinct underlying constructs in the dataset, and multivariable linear regression of factor scores to analyse physician subgroups.

Setting: Six public and private general hospitals in Jakarta in 2019.

Participants: 1007 of 1896 (53.1% response rate) antibiotic prescribing physicians.

Results: EFA identified six latent factors (overall Crohnbach's α=0.85): awareness of AMS activities; awareness of AMS purpose; views regarding rational antibiotic prescribing; confidence in antibiotic prescribing decisions; perception of AMR as a significant problem; and immediate actions to contain AMR. Physicians acknowledged the significance of AMR and contributing factors, rational antibiotic prescribing, and purpose and usefulness of AMS. However, this conflicted with reported suboptimal local hospital practices, such as room cleaning, hand hygiene and staff education, and views regarding antibiotic decision-making. These included insufficiently applying AMS principles and utilising microbiology, lack of confidence in prescribing decisions, and defensive prescribing due to pervasive diagnostic uncertainty, fear of patient deterioration or because patients insisted. Physicians' factor scores differed across hospitals, departments, work experience and medical hierarchy.

Conclusions: AMS implementation in Indonesian hospitals is challenged by institutional, contextual and diagnostic vulnerabilities, resulting in externalising AMR instead of recognising it as a local problem. Appropriate recognition of the contextual determinants of antibiotic prescribing decision-making will be critical to change physicians' attitudes and develop context-specific AMS interventions.

Keywords: perceptions; survey; antibiotic resistance; antibiotic prescribing; antibiotic stewardship; Indonesia

For peer review only

Strengths and limitations of this study

- The self-developed questionnaire in this study identified a relevant set of attributes through a factor analysis optimization process, with adequate content, face and construct validity and internal reliability. This study adds important value in the absence of adequately validated instruments regarding antimicrobial resistance and stewardship, with particular applicability for LMIC.
- This study had a large, varied respondent sample and high response rate among physicians at six public and private hospitals in Jakarta, Indonesia, and identified differences between physicians across hospitals, departments, work experience and medical hierarchy, which can guide priority-setting and tailoring of stewardship interventions.
- However, non-participation and the convenient hospital sample could have introduced selection bias, and the data are not necessarily representative for Jakarta or Indonesia.
- Factor analysis is based on using a "heuristic", which leaves room to more than one interpretation of the same data and cannot identify causality.

For peer review only

Introduction

The global rise in drug-resistant infections is one of the leading threats to public health globally, with increasing rates of morbidity, mortality and escalating healthcare costs[1]. Misuse and overuse of antimicrobial drugs in human health care is one of the main drivers[2,3] and also represents a key solution, i.e. judicious use of remaining antibiotics. Globally, use of antibiotics remains largely unrestrained and poorly governed, with large, unregulated healthcare systems representing an increasingly challenging area for achieving the goal of optimization. Substantial variations in contributing factors to inappropriate antibiotic prescribing exist across contexts, e.g. diagnostic uncertainty, pressure from pharmaceutical industry or patients[4,5], with the structure and funding of health systems inflecting enactment of optimization strategies, including antimicrobial stewardship (AMS)[6].

AMS programs aim to control antimicrobial use, and have been associated with reducing hospital-acquired infections, unnecessary healthcare costs, and potentially drug-resistant infections[7–9]. However, AMS programmes in turn may jar with local constraints and practices and have been shown to have limited traction when attempts to implement occur without adequate understanding of context[10].

The global push to enact effective AMS requires detailed, context-specific data on physicians, given their central role in the complex process of antibiotic prescribing in hospitals, which can inform on how AMR is conceived, how current prescribing is rationalised, and how broad AMS principles may be experienced across contexts and nations[11,12]. Few studies to date have been conducted on this topic in low and middle-income countries (LMIC), with insufficient evaluation of the psychometric properties of their measurement instruments to examine their suitability to the specific context[4,5]. Indonesia, a diverse middle-income country in Southeast Asia with the world's fourth largest population (275 million), is particularly vulnerable for AMR, driven by dense urban populations combined with rising antibiotic consumption[13], a decentralised health system[14], and weakly enforced antibiotic policies[15], hence promoting inappropriate prescribing and over-the-counter access without a prescription. Despite progress in government policies, AMS is generally in an early stage of implementation[15,16].

To identify context-specific opportunities for AMS interventions, we conducted a questionnaire-based survey among antibiotic-prescribing physicians in hospitals in Jakarta, Indonesia, to evaluate their perceptions of AMR, accounts of antibiotic prescribing, and views on AMS. We performed exploratory factor analysis (EFA) to evaluate the construct

For peer review only

validity and psychometric properties of the questionnaire, identify distinct underlying constructs in the data, and explore differences between physician subgroups.

Methods

Study design and setting

We conducted a cross-sectional survey between March and August 2019 among all antimicrobial prescribing physicians at six public and private general hospitals in Jakarta, Indonesia, as part of a mixed-method study to identify targets for quality improvement in antibiotic prescribing practices (EXPLAIN study[17]). The hospitals included two tertiary-care government hospitals and four secondary hospitals, three of which were private hospitals. At the time of the survey, all six hospitals had an AMS programme, albeit at an early stage of implementation.

All qualified physicians prescribing antibiotics on a regular basis working across all clinical departments were eligible to participate, including interns/internship doctors *(magang/dokter internsip),* general practitioners (*dokter umum*) (GPs), residents (*residen*), specialist/consultant physicians (*dokter spesialis/konsultan*), and others.

Ethical considerations

The research ethics committee of the Faculty of Medicine University of Indonesia (1364/UN2.F1/ETIK/2018) and the Oxford Tropical Research Ethics Committee (559-18) approved the study, with additional permission from hospital management. As the survey was anonymous, participant consent was inferred when the doctor completed and returned the questionnaire, as explained in the survey introduction.

Patient and public involvement statement

Patients or the public were not involved in the design, conduct, or reporting of the research.

Survey questionnaire

We developed a two-page anonymous, self-administered, paper-based questionnaire, which was easy to complete and based on a conceptual framework that included attributes related to prescribers' perceptions, views and practices. Behavioural theories, good practice recommendations for questionnaire design and existing questionnaires in the literature were reviewed and discussed with several experts. The Clinician Pre/Post Perception Survey of the Greater New York Hospital Association United Hospital Fund[18] constituted the initial set of items, supplemented with relevant items from other existing questionnaires[19–25]. From a preliminary pool, we selected 69 items, of which 8 items were worded in the negative to

For peer review only

address the acquiescence effect. The instrument was translated from English to Indonesian, and back-translated by an independent translator. The questionnaire was pre-tested by a convenience panel of 18 physicians (2 GPs, 15 residents, and 1 consultant), and adjustments were made in accordance with their feedback, reducing the number of items to 40. The final version took about 10 minutes to complete.

The final questionnaire included an explanation of study purpose and completion instructions; 40 short statements (items) to which participants were asked to indicate the extent to which each reflected their own opinion on a 5-point Likert scale, divided into 3 sections: scope of the AMR problem and key contributors; antibiotic prescribing practices; AMS; and respondent socio-demographics (**Appendix**).

Respondent recruitment

The hospital management provided the total number of prescribing physicians for each department. The questionnaires were delivered to the head of each unit who then distributed the survey to all eligible staff. The study coordinator kept a record of numbers of physicians approached and participated. Upon survey completion, respondents could enter a raffle to win one of three gift cards in each hospital (US$14 each); there were no other incentives for participation.

Ethical approval

The research ethics committee of the Faculty of Medicine University of Indonesia (1364/UN2.F1/ETIK/2018) and the Oxford Tropical Research Ethics Committee (559-18) approved the study, with additional permission from hospital management. As the survey was anonymous, consent was inferred when the participant completed and returned the questionnaire.

Statistical analysis

The percentage of respondents selecting each answer choice was calculated using the total number of responses as the denominator. For an EFA, a common lower bound for sample size is 10 cases per variable, suggesting a minimum sample size of 400; to allow for meaningful subgroup comparisons and minimize selection bias, we targeted a >50% response rate and a sample size of >1000 across the six hospitals. We performed EFA to identify underlying distinct constructs, using factor, pcf command in Stata with orthogonal (varimax) rotation. For this analysis, the eight items worded in the negative were reverse-coded, and missing data for categorical variables were treated as a separate category. The Kaiser-Meyer-Olkin (KMO) was calculated to ensure EFA requirements were met. Each item was assigned to a certain factor based on the highest absolute factor loading of the rotated

For peer review only

solution, and we then assigned an umbrella term to each factor. After the optimal factor solution had been achieved, we calculated factor scores using the regression scoring method, and Cronbach's α coefficient to test internal reliability. Using the factor scores (dependent variable), multivariable mixed-effects linear regression was used to analyze physician subgroups (independent variables i.e., hospital sector and care level, grouped departments, work experience and medical hierarchy), while adjusting for possible clustering within hospitals as well as relevant confounders. P-values<0.05 were considered statistically significant. All analyses were done with Stata/IC Version 16.1 (StataCorp, College Station, TX, US).

For peer review only

Results

Respondent characteristics

All 1896 antibiotic prescribing physicians at the six hospitals were approached, and 1007 (53.1%) participated in the survey. **Table 1** summarizes the participants' key characteristics. **Table S1** summarizes the response rates.

Exploratory factor analysis

The KMO was 0.8773 overall and >0.5 for all items, suggesting the data were suitable for EFA. Analysis of the scree plot (**Figure S1**) indicated a case for four factors, whereas the parallel analysis (**Figure S2**) indicated a case for seven factors. The four-factor solution yielded strong factors but explained only 39.9% of the variance and lacked a theoretical basis for one factor. The seven-factor solution contained one factor with only three items that was difficult to interpret; two of these items (Q9 and Q10) did not load well with any factor in various alternative factor solutions and were removed. Therefore, a six-factor model with a clear theoretical basis based on 38 items was deemed most suitable, explaining 47.4% of the variance, with KMO 0.8802 overall and >0.5 for each item (**Table 2**). The six latent factors are (**Table 3**, see **Table S2** for details): 1) Awareness of AMS activities; 2) Awareness of AMS purpose; 3) Views regarding rational antibiotic prescribing; 4) Confidence in antibiotic prescribing decisions; 5) Perception of AMR as a significant problem; and 6) Immediate actions to contain AMR. Internal reliability was excellent for the overall 38-item scale (α=0.85) and factor 1 (α=0.8734) and 2 (α=0.8334), good for factor 3, 4 and 5 (α=0.70 each), and acceptable for factor 6 (α=0.57).

Description of respondent responses

Figure 1 and **Table S3** summarise the responses to all 40 items.

Scope of the AMR problem and key contributors Most respondents agreed that AMR is an important problem in Indonesia (93.8%; 944/1006) [Q2]; in communities outside of the hospital (83.6%; 838/1003) [Q6]; and at their hospital (80.4%; 908/1005) [Q1], with 30.9% (311/1005) agreeing that patients are likely to develop an infection with a multidrug-resistant infection [Q10]. Most acknowledged as key contributing factors: overuse of antimicrobial drugs (95.1%; 954/1003) [Q3], lack of hand hygiene (71.1%; 715/1005) [Q4], use of broad-spectrum antibiotics (80.5%; 808/1004) [Q5]. Current infection and prevention control (IPC) practices at their hospital were regarded as suboptimal: 64.8% (651/1004) thought that patient rooms are cleaned according to hospital protocol after discharge of a patient with a multidrug-resistant organism [Q7]; 56.9% (570/1002) thought adherence to hand-hygiene

For peer review only

protocols to be excellent [Q8]; and 22.5% (226/1005) felt that their hospital does not provide adequate staff education regarding multidrug-resistant organisms [Q9].

Antibiotic prescribing practices Whereas most respondents (85.7%; 861/1005) agreed that antibiotics are overused in Indonesia (Q11), only 35.5% (357/1005) acknowledged this to be case at their hospital [Q12]. Most agreed that more judicious antimicrobial prescribing practices would decrease AMR (94.8%; 953/1005) [Q17] and that following evidence-based antibiotic guidelines will help optimize treatment outcomes (95.3%; 958/1006) [Q18]. Most gave high priority to rational antibiotic prescribing to their patients (88.8%; 892/1005) [Q19], and considered developing hospital antibiotic guidelines more useful than applying international guidelines (78.4%; 787/1004) [Q20]. Nearly a quarter of respondents indicated to be often unsure if a patient needs an antibiotic or not (23.6%; 237/1006) [Q21] or which antibiotic to prescribe (21.3%; 215/1006) [Q22]. A small but considerable fraction expressed lack of confidence in prescribing decisions, i.e. 12.2% (123/1005) prescribed in patients with just a high fever (≥39°C) [Q24], 36.6% (368/1005) when they felt uncertain about the diagnosis of infection [Q25], 35.0% (352/1006) prescribed more freely because of fear of clinical failure [Q26], and 9.8% (98/1005) frequently prescribed antibiotics because patients or their relatives insist [Q27]. Just more than half of the respondents reported that microbiology laboratory results are efficiently communicated to the treating physician (57.3%; 576/1005) [Q13], considered the hospital antibiogram when empirically prescribing antibiotics (54.5%; 548/1005) [Q14], and would stop antibiotics that others have prescribed in the absence of an appropriate indication (57.0%; 571/1002) [Q23]. Most (72.7%; 731/1006) agreed that, if medically appropriate, intravenous antibiotics should be stepped down to an oral alternative after three days [Q15]. Notably, 33.6% (338/1006) felt that restrictions on antibiotics impaired their ability to provide good patient care [Q16].

Antibiotic stewardship When asked about AMS in general, most respondents were aware that their hospital had an AMS programme (93.1%; 937/1006) [Q28], they reported they understood its purpose (92.1%; 927/1006) [Q29], and they agreed that AMS can improve patient care (88.6%; 891/1006) [Q30], reduce AMR (88.3%; 887/1005) [Q31] and hospital-acquired infections (76.7%; 770/1004) [Q32]. When asked about the usefulness of specific AMS activities, most respondents acknowledged that additional education on antibiotic prescribing was needed (88.4%; 888/1005) [Q33], regular audit and feedback encouraged them to prescribe antibiotics prudently (92.2%; 928/1006) [Q34], rapid and accurate diagnostic tests are useful for diagnosis of infectious diseases and guidance on antibiotic therapy (96.5%; 971/1006) [Q35], implementation of antibiotic restriction (e.g. antibiotic tiers) can reduce antibiotic overuse in hospitals (90.4%; 910/1007) [Q36], regular consultations or ward rounds with a clinical microbiologist or infectious disease physician can curb AMR

For peer review only

(85.4%; 859/1006) [Q37], timely access to microbiological test results is needed to guide antibiotic therapy (92.4%; 930/1006) [Q38], and IPC in the hospital can reduce AMR (95.3%; 959/1006) [Q40].

Physician subgroup analysis

Table 4 summarizes the results of the subgroup analysis.

Hospitals Statistically significant differences were identified between hospitals for awareness of AMS purposes (factor 2), views regarding rational antibiotic prescribing (factor 3), perception of AMR as a significant problem (factor 5) and immediate actions to contain AMR (factor 6), but not for awareness of AMS activities (factor 1) and confidence in antibiotic prescribing decisions (factor 4). None of the factor scores differed between prescribers in public versus private, or secondary versus tertiary hospitals.

Professional hierarchy For awareness of AMS activities (factor 1), consultants, GPs and residents scored higher than interns. For awareness of AMS purposes (factor 2), consultants scored lower than interns and residents. For views regarding rational antibiotic prescribing (factor 3), consultants scored higher than GPs. For confidence in antibiotic prescribing decisions (factor 4), consultants scored lower than residents, whereas for immediate actions to contain AMR (factor 6), consultants scored higher than residents. No differences were identified for perception of AMR as a significant problem (factor 5).

Departments For awareness of AMS activities (factor 1) and purpose (factor 2), physicians in surgery and medicine scored higher than the acute specialties, whereas for awareness of AMS activities (factor 1), medicine scored higher than surgery. For views regarding rational antibiotic prescribing (factor 3), surgery scored higher than acute specialties. For confidence in antibiotic prescribing decisions (factor 4), medicine scored lower than surgery and other specialties. For perception of AMR as a significant problem (factor 5), surgery scored lower than medicine. For immediate actions to contain AMR (factor 6), surgery scored higher than medicine and other specialties, and the acute specialties scored higher than surgery and medicine.

Work experience. Physicians with little (0-5 years) work experience scored lower than more experienced colleagues, for awareness of AMS purpose (factor 2); for perception of AMR as a significant problem (factor 5); and for immediate actions to contain AMR (factor 6). No differences were identified for factors 1, 3 and 4.

For peer review only

Discussion

This survey assessed the perceptions, views and practices regarding AMR, antibiotic prescribing and AMS among over 1000 physicians in Indonesian hospitals. Through an exploratory factor analysis we identified six distinct constructs in the dataset, i.e., 1) awareness of AMS activities; 2) awareness of AMS purposes; 3) views regarding rational antibiotic prescribing; 4) confidence in antibiotic prescribing decisions; 5) perception of AMR as a significant problem; and 6) immediate actions to contain AMR. The survey findings outline a series of dynamics around AMR and AMS in the Indonesian context. Spanning issues around *visibility* (diagnostics)[26], *awareness* (education)[27] and *institutional form* (governance)[28], the survey results tease out many of the core issues illustrated in other settings[4,5], but in turn, illustrate a specific mix of variables at play, i.e. uncertainty, risk, and lack of sense of responsibility. For instance, only about one-third of physicians recognised that antibiotic overuse was an issue at their own hospital, many physicians were hesitant to stop antibiotics that others had prescribed in the absence of an appropriate indication, and felt that antibiotic restrictions impaired their ability to provide good patient care. Lack of confidence in prescribing decisions and defensive prescribing were common due to diagnostic uncertainty, fear of patient deterioration or complications, or because patients or their relatives insisted. The study findings expand on our recently published paper on the patterns and quality indicators of antibiotic prescribing in the same hospitals, which identified several priority areas for stewardship[17].

The most significant factor in guiding the future agenda in Indonesia around effective AMS implementation, is the perceived "externality of AMR" as a problem[29]. That is, physicians acknowledge its significance but do not take ownership or responsibility, thus reflecting a production of AMR as an externality, e.g. a result of irrational use elsewhere in communities or other hospitals. The lack of systematic surveillance of AMR and antibiotic use and the underutilisation of bacterial cultures, recognised by many of the respondents, also reproduces the perception of AMR as a "problem of elsewhere". This feeds a lack of engaging with AMS, as it is not recognised as a value-add for an already stretched institutional context, and in turn provides the context for continued defensive prescribing "to be on the safe side". Moreover, defensive prescribing practices somewhat offset (in the short term) problems around room cleaning, hand hygiene and staff education. In this way, AMR as an externality and the vulnerabilities of the institution, offer an environment conducive to the ongoing over-use of antimicrobials[30,31]. The higher incidence of hospital-acquired infections in LMIC than in high-income countries could further promote defensive prescribing as a way to compensate for substandard IPC practices[32]. All in all, this supports the notion that physicians tend to prioritise managing immediate clinical risks, reputation and concordance with peer practice, vis-à-vis the long-term population consequences of AMR[33].

For peer review only - http://bmjopen.bmj.com/site/about/guidelines.xhtml

Work experience and medical hierarchy were found to influence the awareness of AMS purpose, AMR as a significant problem, and immediate actions to contain AMR. Interestingly, compared with junior physicians, specialists/consultants expressed lower confidence to make antibiotic decisions in uncertain situations while showing higher confidence in actions to contain AMR. Possible explanations include that specialists/consultants have a better recognition of the "unknowns" (e.g. lack of data on bacterial susceptibility patterns) and that they bear final patient responsibility, introducing the fear of losing a patient or legal consequences[34], whereas taking actions to curb AMR can be a remedy to compensate their fear. Conversely, residents' higher confidence in antibiotic prescribing may also relate to their contemporary medical training, which includes AMS, as opposed to late-career physicians[35]. GPs had low scores on views regarding rational antibiotic prescribing compared with consultants which could reflect the GPs' limited responsibility in the antibiotic decision-making hierarchy, possibly leading to a lack of positive attitude towards guidelines and preference for complying with them[36,37].

The acute specialties (including emergency, ICU and anaesthesiology) had lower awareness of AMS activities and purposes, compared with surgery or medicine, but scored higher for immediate actions to contain AMR. Compared with surgeons, physicians in medicine had a greater awareness of AMS activities and recognition of AMR as a significant problem, but they had lower confidence in antibiotic prescribing decisions and immediate actions to contain AMR. These observations are in line with a UK study that found that emergency physicians experienced pressure for immediate action out of fear of losing a patient and a lack of ownership of antibiotic decision-making due to the patient transitioning to inpatient care, that medical doctors adopted a more policy-informed, interdisciplinary approach, and that senior surgeons left complex antibiotic decisions to junior staff, resulting in potential defensive and inappropriate antibiotic use[38]. Variations in the social norms, values and behaviours between specialties should inform what is the best approach to antibiotic decision-making.

Our study had several strengths and weaknesses. First, based on our self-developed questionnaire, we were able to identify a relevant set of attributes through a factor analysis optimization process, with adequate content, face and construct validity and internal reliability. In the absence of adequately validated instruments regarding AMR and AMS[39], this study adds important value to the field, with particular relevance and applicability for LMIC. Nonetheless, further questionnaire validation steps (such as criterion-related validity) are necessary to achieve a fully valid and reliable instrument. Second, the study had a large, varied respondent sample and high response rate. However, non-participation and the

For peer review only

convenient hospital sample could have introduced selection bias, and the data are not necessarily representative for Jakarta or Indonesia at large. The authenticity of the answers was maximised by protecting the respondents' identities, although reliance on self-report has potential for social desirability bias. Third, factor analysis is based on using a "heuristic", which leaves room to more than one interpretation of the same data and cannot identify causality.

Conclusion

AMS implementation in Indonesian hospitals is likely highly dependent on institutional, contextual and diagnostic vulnerabilities. These may result in the problem of AMR being externalised, instead of recognised as a local hospital problem. Current AMS strategies may be insufficiently successful in promoting prudent antibiotic use, due to lack of systematic engagement with and feedback to prescribers, aimed at building confidence in antibiotic decision-making and ownership of the AMR problem. Appropriate recognition of the contextual and social determinants of antibiotic prescribing decision-making, including hospital factors, dynamics in medical hierarchy and experience, among others, will be critical to design context-specific AMS interventions that are adopted by healthcare professionals and successfully influence behaviours[12].

For peer review only

Acknowledgements

The authors are grateful to the management, research/medical committees and clinicians of the participating hospitals for their support to the study.

EXPLAIN study group

Ralalicia Limato, Erni J. Nelwan, Manzilina Mudia, Monik Alamanda, Helio Guterres, Enty Enty, Elfrida R. Manurung, Ifael Y. Mauleti, Maria Mayasari, Iman Firmansyah, May Hizrani, Roswin Djafar, Raph L. Hamers, Anis Karuniawati, Prof Taralan Tambunan, Prof Amin Soebandrio, Decy Subekti, Iqbal Elyazar, Mutia Rahardjani, Fitria Wulandari, Prof Reinout van Crevel, H. Rogier van Doorn, Vu Thi Lan Huong, Nga Do Ti Thuy, Sonia Lewycka, Prof Alex Broom

Funding

This work was funded by the Wellcome Trust, UK (106680/Z/14/Z). RL is supported by an OUCRU Prize Studentship and a Nuffield Dept of Medicine Tropical Network Fund DPhil Bursary.

Conflicts of interest

The authors declare no competing interests.

Author contributions

EJN and RLH conceived the idea for the study and are the principal investigators. RLH and RL obtained the funding. RL, EJN, HVTL and RLH designed the study protocol and developed the study instrument. MM, ERM, IYM, MM, IF, and RD collected and verified the data, overseen by RL. RL, MM, MA created and curated the database. RLH and MA performed the analysis and had full access to all study data. RL, MA, and RLH drafted the paper, with critical inputs from EJN, HVTL, HRvD and AB. All authors critically revised the manuscript and gave approval for the final version to be published.

Data Availability Statement

De-identified data are available upon reasonable request via the corresponding author, after written permission has been obtained from the lead investigators.

For peer review only

References

1 O'Neill J. Tackling Drug-Resistant Infections Globally: Final Report and Recommendations. 2016.

2 Klein EY, Van Boeckel TP, Martinez EM, *et al.* Global increase and geographic convergence in antibiotic consumption between 2000 and 2015. *Proc Natl Acad Sci U S A* 2018; **115**: E3463–70.

3 Goossens H, Ferech M, Vander Stichele R, Elseviers M. Outpatient antibiotic use in Europe and association with resistance: a cross-national database study. *Lancet* 2005; **365**: 579–87.

4 Chaw PS, Höpner J, Mikolajczyk R. The knowledge, attitude and practice of health practitioners towards antibiotic prescribing and resistance in developing countries—A systematic review. *J Clin Pharm Ther* 2018; **43**: 606–13.

5 Md Rezal RS, Hassali MA, Alrasheedy AA, Saleem F, Md Yusof FA, Godman B. Physicians' knowledge, perceptions and behaviour towards antibiotic prescribing: A systematic review of the literature. *Expert Rev Anti Infect Ther* 2015; **13**: 665–80.

6 Do NTT, Vu HTL, Nguyen CTK, *et al.* Community-based antibiotic access and use in six low-income and middle-income countries: a mixed-method approach. *Lancet Glob Heal* 2021; published online April. DOI:10.1016/S2214-109X(21)00024-3.

7 Laundy M, Gilchrist M, Whitney L. Antimicrobial Stewardship. Oxford, UK: Oxford University Press, 2016 DOI:10.1093/med/9780198758792.001.0001.

8 Schuts EC, Hulscher MEJL, Mouton JW, *et al.* Current evidence on hospital antimicrobial stewardship objectives: A systematic review and meta-analysis. *Lancet Infect Dis* 2016; **16**: 847–56.

9 Honda H, Ohmagari N, Tokuda Y, Mattar C, Warren DK. Antimicrobial stewardship in inpatient settings in the Asia pacific region: A systematic review and meta-analysis. *Clin Infect Dis* 2017; **64**: S119–26.

10 Rolfe R, Kwobah C, Muro F, *et al.* Barriers to implementing antimicrobial stewardship programs in three low- and middle-income country tertiary care settings: findings from a multi-site qualitative study. *Antimicrob Resist Infect Control* 2021; **10**: 60.

11 Teixeira Rodrigues A, Roque F, Falcão A, Figueiras A, Herdeiro MT. Understanding physician antibiotic prescribing behaviour: a systematic review of qualitative studies. *Int J Antimicrob Agents* 2013; **41**: 203–12.

12 Aveling E-L, Martin G, Armstrong N, Banerjee J, Dixon-Woods M. Quality improvement through clinical communities: eight lessons for practice. *J Health Organ Manag* 2012; **26**: 158–74.

13 Sriram A, Kalanxhi E, Kapoor G, *et al.* Center for Disease Dynamics, Economics & Policy, Washington DC. State of the world's antibiotics 2021: A global analysis of

For peer review only

antimicrobial resistance and its drivers. 2021.
14 Agustina R, Dartanto T, Sitompul R, *et al.* Universal health coverage in Indonesia: concept, progress, and challenges. *Lancet* 2019; **393**: 75–102.
15 Parathon H. Progress towards antimicrobial resistance containment and control in Indonesia. *Bmj* 2017; **358**.
16 Herawati F, Ananta SC, Parwitha IAA, *et al.* Interview-based cross-sectional needs assessment to advance the implementation of an effective antibiotic stewardship program in Indonesian hospitals. *Heal Policy OPEN* 2020; **1**: 100002.
17 Limato R, Nelwan EJ, Mudia M, *et al.* A multicentre point prevalence survey of patterns and quality of antibiotic prescribing in Indonesian hospitals. *JAC-Antimicrobial Resist* 2021; **3**. DOI:10.1093/jacamr/dlab047.
18 Greater New York Hospital Association United Hospital Fund. Antimicrobial Stewardship Toolkit. Clinician Pre-/Post-Perception Survey (Appendix B). 2011.
19 Chaw PS, Schlinkmann KM, Raupach-Rosin H, *et al.* Knowledge, attitude and practice of Gambian health practitioners towards antibiotic prescribing and microbiological testing: a cross-sectional survey. *Trans R Soc Trop Med Hyg* 2017; **111**: 117–24.
20 Asante KP, Boamah EA, Abdulai MA, *et al.* Knowledge of antibiotic resistance and antibiotic prescription practices among prescribers in the Brong Ahafo Region of Ghana; a cross-sectional study. *BMC Health Serv Res* 2017; **17**: 422.
21 Labi A-K, Obeng-Nkrumah N, Bjerrum S, *et al.* Physicians' knowledge, attitudes, and perceptions concerning antibiotic resistance: a survey in a Ghanaian tertiary care hospital. *BMC Health Serv Res* 2018; **18**: 126.
22 Venugopalan V, Trustman N, Manning N, Hashem N, Berkowitz L, Hidayat L. Administration of a survey to evaluate the attitudes of house staff physicians towards antimicrobial resistance and the antimicrobial stewardship programme at a community teaching hospital. *J Glob Antimicrob Resist* 2016; **4**: 21–7.
23 García C, Llamocca LP, García K, *et al.* Knowledge, attitudes and practice survey about antimicrobial resistance and prescribing among physicians in a hospital setting in Lima, Peru. *BMC Clin Pharmacol* 2011; **11**: 18.
24 Alumran A, Hou X-Y, Hurst C. Validity and reliability of instruments designed to measure factors influencing the overuse of antibiotics. *J Infect Public Health* 2012; **5**: 221–32.
25 Alothman A, Algwizani A, Alsulaiman M, Alalwan A, Binsalih S, Bosaeed M. Knowledge and Attitude of Physicians toward Prescribing Antibiotics and the Risk of Resistance in Two Reference Hospitals. *Infect Dis Res Treat* 2016; **9**: IDRT.S40047.
26 Chandler CI., Hutchinson E, Hutchison C. Addressing Antimicrobial Resistance Through Social Theory: An Anthropologically Oriented Report. London School of

For peer review only

Hygiene & Tropical Medicine. 2016.

27 Charoenboon N, Haenssgen MJ, Warapikuptanun P, Xayavong T, Khine Zaw Y. Translating antimicrobial resistance: a case study of context and consequences of antibiotic-related communication in three northern Thai villages. *Palgrave Commun* 2019; **5**: 23.

28 Broom A, Doron A. Antimicrobial Resistance, Politics, and Practice in India. *Qual Health Res* 2020; **30**: 1684–96.

29 Broom A, Broom J, Kirby E, Gibson A, Davis M. Antibiotic optimisation in 'the bush': Local know-how and core-periphery relations. *Health Place* 2017; **48**: 56–62.

30 Broom A, Kirby E, Gibson AF, Post JJ, Broom J. Myth, Manners, and Medical Ritual: Defensive Medicine and the Fetish of Antibiotics. *Qual Health Res* 2017; **27**: 1994–2005.

31 Will CM. Editorial: Beyond behavior? Institutions, interactions and inequalities in the response to antimicrobial resistance. Sociol. Health Illn. 2018; **40**: E1–9.

32 Chandler CIR. Current accounts of antimicrobial resistance: stabilisation, individualisation and antibiotics as infrastructure. *Palgrave Commun* 2019; **5**: 53.

33 Broom A, Broom J, Kirby E. Cultures of resistance? A Bourdieusian analysis of doctors' antibiotic prescribing. *Soc Sci Med* 2014; **110**: 81–8.

34 Broom A, Broom J, Kirby E, Adams J. The social dynamics of antibiotic use in an Australian hospital. *J Sociol* 2015; **52**: 824–39.

35 Fernandez-Lazaro CI, Brown KA, Langford BJ, Daneman N, Garber G, Schwartz KL. Late-career Physicians Prescribe Longer Courses of Antibiotics. *Clin Infect Dis* 2019; **69**: 1467–75.

36 R Hansen C, Bradley CP, Sahm LJ. Factors Influencing Successful Prescribing by Intern Doctors: A Qualitative Systematic Review. *Pharm (Basel, Switzerland)* 2016; **4**: 24.

37 Roumie CL, Halasa NB, Edwards KM, Zhu Y, Dittus RS, Griffin MR. Differences in antibiotic prescribing among physicians, residents, and nonphysician clinicians. *Am J Med* 2005; **118**: 641–8.

38 Charani E, Ahmad R, Rawson TM, Castro-Sanchèz E, Tarrant C, Holmes AH. The Differences in Antibiotic Decision-making Between Acute Surgical and Acute Medical Teams: An Ethnographic Study of Culture and Team Dynamics. *Clin Infect Dis* 2019; **69**: 12–20.

39 DeVon HA, Block ME, Moyle-Wright P, *et al.* A psychometric toolbox for testing validity and reliability. *J Nurs Scholarsh an Off Publ Sigma Theta Tau Int Honor Soc Nurs* 2007; **39**: 155–64.

For peer review only

Appendix: Survey questionnaire

We kindly request you to indicate your agreement with the following 40 statements using a five-point scale:
① Strongly Disagree ② Disagree ③ Neutral or Don't Know ④ Agree ⑤ Strongly Agree

**ANTIMICROBIAL RESISTANCE: SCOPE OF THE PROBLEM AND KEY CONTRIBUTORS**						
**1.**	Antimicrobial resistance is a significant problem in this hospital	①	②	③	④	⑤
**2.**	Antimicrobial resistance is a significant problem in Indonesia	①	②	③	④	⑤
**3.**	A cause of antimicrobial resistance is using too many antimicrobial drugs	①	②	③	④	⑤
**4.**	Lack of hand disinfection by healthcare workers causes spread of antimicrobial resistance	①	②	③	④	⑤
**5.**	Use of broad-spectrum antibiotics can increase antimicrobial resistance when narrower-spectrum antibiotics are available that are equally effective	①	②	③	④	⑤
**6.**	Antibiotic resistance is also a problem outside of the hospital, in communities	①	②	③	④	⑤
**7.**	In this hospital, patient rooms are cleaned according to hospital cleaning protocol once a patient with a multidrug-resistant organism (MDRO) has been discharged	①	②	③	④	⑤
**8.**	Adherence to hand-hygiene protocols is excellent at this hospital	①	②	③	④	⑤
**9.**	This hospital does NOT provide adequate staff education regarding multidrug-resistant organisms	①	②	③	④	⑤
**10.**	A patient is likely to develop an infection with a multidrug-resistant organism during their stay at this hospital	①	②	③	④	⑤
**ANTIBIOTIC PRESCRIBING PRACTICES**						
**11.**	Antibiotics are overused in Indonesia	①	②	③	④	⑤
**12.**	Antibiotics are overused in this hospital	①	②	③	④	⑤
**13.**	Microbiology laboratory results are efficiently communicated to the treating physician	①	②	③	④	⑤
**14.**	I regularly refer to/consider the antibiotic susceptibility patterns at this hospital/institution (i.e. the institutional antibiogram) when empirically prescribing antibiotics	①	②	③	④	⑤
**15.**	If medically appropriate, intravenous antibiotics should be stepped down to an oral alternative after three days	①	②	③	④	⑤
**16.**	Restrictions on antibiotics impair my ability to provide good patient care	①	②	③	④	⑤
**17.**	More judicious use of antibiotics would decrease antimicrobial resistance	①	②	③	④	⑤
**18.**	Following evidence-based antibiotic guidelines will help optimize treatment outcomes	①	②	③	④	⑤
**19.**	In general, rational antibiotic prescribing for my patients is high on my list of priorities	①	②	③	④	⑤
**20.**	Developing hospital antibiotic guidelines is more useful than applying international guidelines	①	②	③	④	⑤

For peer review only

**21.**	I am often unsure if a patient needs an antibiotic or not	①	②	③	④	⑤
**22.**	I am often unsure which antibiotic to prescribe	①	②	③	④	⑤
**23.**	I will stop antibiotics that others have prescribed in the absence of an appropriate indication	①	②	③	④	⑤
**24.**	Patients with high fever (≥39°C) must be treated with antibiotics	①	②	③	④	⑤
**25.**	If I am uncertain about the diagnosis of infection, but think it is possible, I feel safer prescribing an antibiotic	①	②	③	④	⑤
**26.**	Fear of patient deterioration or complications leads me to prescribe antibiotics more freely	①	②	③	④	⑤
**27.**	I frequently prescribe antibiotics because patients or their relatives insist on it	①	②	③	④	⑤
**ANTIMICROBIAL STEWARDSHIP PROGRAM (ASP)** ***A formal program formal that monitors and manages the appropiate use of antibiotics***						
**28.**	I am aware that my hospital has an antimicrobial stewardship program (ASP)	①	②	③	④	⑤
**29.**	I understand what the purpose of ASP is	①	②	③	④	⑤
**30.**	ASP improve patient care	①	②	③	④	⑤
**31.**	ASP reduces the problem of antimicrobial resistance	①	②	③	④	⑤
**32.**	ASP reduces this hospital's infection rates	①	②	③	④	⑤
**33.**	Additional staff education on antimicrobial prescribing is needed	①	②	③	④	⑤
**34.**	Regular audit and feedback encourage me to prescribe antibiotics prudently	①	②	③	④	⑤
**35.**	Rapid and accurate diagnostic tests are useful for diagnosis of infectious diseases and guidance on antibiotic therapy	①	②	③	④	⑤
**36.**	To reduce antibiotic overuse in hospitals, implementation of antibiotic restriction (e.g., antibiotic tiers) is a useful measure	①	②	③	④	⑤
**37.**	To curb antimicrobial resistance, regular consultations or ward rounds with a clinical microbiologist or infectious disease physician are useful	①	②	③	④	⑤
**38.**	To curb antimicrobial resistance, doctors need to have timely access to microbiological test results to guide antibiotic therapy	①	②	③	④	⑤
**39.**	Up-to-date information on hospital antimicrobial resistance patterns is important for developing hospital antibiotic guidelines	①	②	③	④	⑤
**40.**	Effective infection prevention and control in the hospital reduces antimicrobial resistance	①	②	③	④	⑤

BACKGROUND INFORMATION

41. What is your primary work unit in this hospital? **Tick ONE**

○ Many different unit/not specific	○ OBGYN (Obstetrics/ Gynaecology)	○ Eye	○ Rehabilitation
○ Emergency department	○ Internal Medicine	○ Dermato-venerology	○ Pharmacy
○ Surgery	○ Neurology	○ Pulmonology	○ Microbiology
○ Anaesthesiology	○ Paediatrics	○ Cardiology	○ Laboratory
		○ Orthopaedics	○ Other ________

For peer review only

	○ Intensive Care Unit (ALL)	○ Psychiatrics<br>○ ENT (Ear Nose Throat)	○ Radiology
**42.**	How long have you worked in this hospital? **Tick ONE**		
	○ Less than 1 year<br>○ 1 – 5 years	○ 6 – 10 years<br>○ 11 – 15 years	○ 16 - 20 years<br>○ More than 20 years
**43.**	What is your position in this hospital? **Tick ONE**		
	○ Internship doctor<br>○ General Practitioner	○ Resident<br>○ Specialist	○ Other<br>___________________
**44.**	How long have you worked in your current specialty or profession? **Tick ONE**		
	○ Less than 1 year<br>○ 1 – 5 years	○ 6 – 10 years<br>○ 11 – 15 years	○ 16 - 20 years<br>○ More than 20 years
**45.**	Which of the following resources do you use to guide your antibiotic prescribing? **Tick ALL that apply**		
	○ Consultation with senior colleague(s)<br>○ Consultation with specialist in microbiology/ infectious disease<br>○ Textbooks	Guideline<br>☐ Internasional<br>☐ Nasional<br>☐ Hospital<br>☐ Departement/ division	○ Medical journal<br>○ Phamaceutical representative<br>______________________________<br>○ Internet ______________________<br>○ Other _________________________
**46.**	During the past year, how many times have you received training/teaching or attended seminars/courses on antimicrobial prescribing, resistance and/or stewardship? _____ times		
**47.**	What is your sex: ○ Male ○ Female		
**48.**	I would like to take part in the raffle: ○ yes ○ no If yes, my email is:___________________		

Thank you for completing this survey!

For peer review only

Table 1 Characteristics of respondents

**Total**	**1007**
Sex [a]	
Female	477 (47.4)
Male	524 (52.0)
Professional hierarchy	
Intern/Internship doctor	10 (1.0)
General practitioner	113 (11.2)
Resident	500 (49.7)
Specialist/consultant	358 (35.6)
Other	18 (1.8)
Professional experience (years) [b]	
<1	194 (19.3)
1-5	459 (45.6)
6-10	136 (13.5)
11-15	74 (7.4)
16-20	52 (5.2)
>20	81 (8.0)
Grouped departments [c,f]	
Surgery (including subspecialties)	371 (36.8)
Medicine (including subspecialties)	232 (23.0)
Acute specialties	156 (15.5)
Other departments	244 (24.2)
Information sources used to guide prescribing [d]	
Guidelines	
International	619 (61.5)
National	628 (62.4)
Hospital	656 (65.1)
Department/Division	405 (40.2)
Consultation with senior colleague(s)	472 (46.9)
Consultation with microbiologist/infectious disease physician	523 (51.9)
Textbooks	410 (40.7)
Medical journals	389 (38.6)
Pharmaceutical company representative	34 (3.4)
Internet	115 (11.4)
Other	13 (1.3)
Number of AMR/AMS trainings attended in the past year [e]	
0	396 (39.3)
1	342 (34.0)
≥2	168 (16.7)
Median (range)	1 (0, 10)

Abbreviations: AMR, antimicrobial resistance; AMS, antibiotic stewardship;
Data are reported as n (%) unless indicated otherwise.
Data missing for: [a] 6 (0.60%); [b] 11 (1.1%); [c] 4 (0.40%); [d] 2 (0.20%); [e] 101 (10.0%).
[f] Surgery and surgical subspecialties includes obstetrics/gynaecology (146), surgery (122), orthopaedics (57), ENT (32), urology (14); medicine and medical subspecialties includes medicine (128), neurology (63), pulmonology (15), dermatology (14), cardiology (12); acute specialties includes anaesthesiology (72), emergency (57), ICU (27); other departments includes paediatrics (54), ophthalmology (39), multiple units (33), rehabilitation (32), psychiatry (30), dentist (27), other (29) and unspecified (4).

For peer review only

Table 2 Summary of the exploratory factor analysis of the six-factor solution (n=973)

Item #	Original item	Rotated factor loadings						Uniqueness
		Factor 1	Factor 2	Factor 3	Factor 4	Factor 5	Factor 6	
**Q01**	Antimicrobial resistance is a significant problem in this hospital	0.2159	0.04100	0.1389	0.0057	0.5701	0.0416	0.6056
**Q02**	Antimicrobial resistance is a significant problem in Indonesia	0.2433	0.10730	0.3295	0.0588	0.5742	0.0385	0.4861
**Q03**	A cause of antimicrobial resistance is using too many antimicrobial drugs	0.1641	0.20060	0.3993	0.0695	0.5361	-0.0462	0.4790
**Q04**	Lack of hand disinfection by healthcare workers causes spread of antimicrobial resistance	0.0640	0.09020	-0.2533	0.0152	0.5725	0.1179	0.5817
**Q05**	Use of broad-spectrum antibiotics can increase antimicrobial resistance when narrower-spectrum antibiotics are available that are equally effective	0.2150	0.05360	0.1482	0.0005	0.5480	0.1093	0.6167
**Q06**	Antibiotic resistance is also a problem outside of the hospital, in communities	0.2088	0.03180	0.2463	0.0632	0.4766	0.0432	0.6617
**Q07**	In this hospital, patient rooms are cleaned according to hospital cleaning protocol once a patient with a multidrug-resistant organism (MDRO) has been discharged	0.1038	0.07530	0.0868	0.0210	0.0378	0.6058	0.6071
**Q08**	Adherence to hand-hygiene protocols is excellent at this hospital	-0.0098	0.03640	0.0606	0.1099	-0.0830	0.6368	0.5705
**Q11**	Antibiotics are overused in Indonesia	0.1924	0.18310	0.5138	-0.0809	0.2489	-0.2318	0.5432
**Q12**	Antibiotics are overused in this hospital	0.0548	0.04170	0.2094	-0.4550	0.1635	-0.2381	0.6610
**Q13**	Microbiology laboratory results are efficiently communicated to the treating physician	0.1689	0.11740	-0.0915	0.0532	-0.0963	0.5116	0.6754
**Q14**	I regularly refer to/consider the antibiotic susceptibility patterns at this hospital/institution (i.e. the institutional antibiogram) when empirically prescribing antibiotics	0.0288	0.10700	-0.0292	-0.0237	0.2056	0.6115	0.5701
**Q15**	If medically appropriate, intravenous antibiotics should be stepped down to an oral alternative after three days	-0.0873	0.20850	0.1141	-0.0850	0.2311	0.3617	0.7444
**Q16**	Restrictions on antibiotics impair my ability to provide good patient care	0.0460	0.18390	0.0527	0.4031	0.0843	-0.0868	0.7842
**Q17**	More judicious use of antibiotics would decrease antimicrobial resistance	0.3010	0.07470	0.7362	0.0820	0.0892	0.0648	0.3429
**Q18**	Following evidence-based antibiotic guidelines will help optimize treatment outcomes	0.2274	0.19340	0.6565	0.1206	0.1256	0.1851	0.4153

For peer review only

Item #	Original item	Rotated factor loadings						Uniqueness
**Q19**	In general, rational antibiotic prescribing for my patients is high on my list of priorities	0.1845	0.18040	0.5246	0.1574	0.1184	0.3228	0.5151
**Q20**	Developing hospital antibiotic guidelines is more useful than applying international guidelines	0.1803	0.00750	0.3499	-0.1335	0.2631	0.0672	0.7534
**Q21**	I am often unsure if a patient needs an antibiotic or not	0.0489	0.02600	0.0497	0.5640	-0.3364	0.1021	0.5527
**Q22**	I am often unsure which antibiotic to prescribe	-0.0084	0.04570	0.1256	0.5670	-0.1938	0.0781	0.6170
**Q23**	I will stop antibiotics that others have prescribed in the absence of an appropriate indication	0.0016	-0.06200	0.1517	-0.1137	0.2090	0.3892	0.7650
**Q24**	Patients with high fever (≥39°C) must be treated with antibiotics	0.0077	0.13310	0.1695	0.4794	0.2095	-0.2351	0.6245
**Q25**	If I am uncertain about the diagnosis of infection, but think it is possible, I feel safer prescribing an antibiotic	0.0217	-0.04150	-0.0877	0.6741	0.1927	-0.0086	0.4985
**Q26**	Fear of patient deterioration or complications leads me to prescribe antibiotics more freely	0.0113	0.00390	-0.1459	0.7092	0.0883	0.0246	0.4672
**Q27**	I frequently prescribe antibiotics because patients or their relatives insist on it	0.1069	0.04740	0.2869	0.6318	0.0518	-0.0100	0.5021
**Q28**	I am aware that my hospital has an antimicrobial stewardship program (ASP)	0.2434	0.62240	0.2918	0.0866	0.0418	0.0110	0.4588
**Q29**	I understand what the purpose of ASP is	0.2217	0.69570	0.2635	0.1188	0.0092	0.0199	0.3828
**Q30**	ASP improve patient care	0.2364	0.77440	0.0873	0.0932	0.0852	0.1241	0.3055
**Q31**	ASP reduces the problem of antimicrobial resistance	0.2775	0.75320	0.0949	0.0172	0.0906	0.1753	0.3074
**Q32**	ASP reduces this hospital's infection rates	0.2045	0.66700	-0.1122	0.0569	0.1283	0.1799	0.4486
**Q33**	Additional staff education on antimicrobial prescribing is needed	0.5202	0.28160	0.0917	-0.0079	0.2203	-0.0284	0.5923
**Q34**	Regular audit and feedback encourage me to prescribe antibiotics prudently	0.6151	0.35810	0.0075	0.0064	0.1402	0.1117	0.4612
**Q35**	Rapid and accurate diagnostic tests are useful for diagnosis of infectious diseases and guidance on antibiotic therapy	0.6714	0.25760	0.1522	-0.0362	0.0386	0.0379	0.4555
**Q36**	To reduce antibiotic overuse in hospitals, implementation of antibiotic restriction (e.g. antibiotic tiers) is a useful measure	0.6428	0.26700	0.1088	-0.0335	0.2153	0.0013	0.4562
**Q37**	To curb antimicrobial resistance, regular consultations or ward rounds with a clinical microbiologist or infectious disease physician are useful	0.7046	0.07870	0.0061	0.0190	0.1881	0.0781	0.4555
**Q38**	To curb antimicrobial resistance, doctors need to have timely access to microbiological test results to guide antibiotic therapy	0.7197	0.08350	0.2786	0.0985	0.0670	0.0820	0.3765

For peer review only

Item #	Original item	Rotated factor loadings						Uniqueness
**Q39**	Up-to-date information on hospital antimicrobial resistance patterns is important for developing hospital antibiotic guidelines	0.7374	0.08540	0.3223	0.1088	0.1739	0.0348	0.3018
**Q40**	Effective infection prevention and control in the hospital reduces antimicrobial resistance	0.7067	0.16900	0.2876	0.0950	0.1798	0.0533	0.3452
	**Eigenvalues**	**4.39**	**3.19**	**2.82**	**2.78**	**2.65**	**2.17**	
	**% of variance explained**	**11.56%**	**8.40%**	**7.43%**	**7.32%**	**6.98%**	**5.72%**	**Overall 47.4%**

Table shows the results of the exploratory factor analysis (principal axis factoring) with orthogonal varimax rotation of the six-factor solution using the factor, pcf command in Stata.
Rotated factor loadings: a measure of how much each item contributes to the factor. Loadings close to -1 or 1 indicate that the factor strongly affects the item and loadings close to 0 indicate that the factor has a weak effect on the item.
Uniqueness: shows the proportion of the item's variance that is not explained by the factors.
Item #9 and 10 were excluded from the analysis as explained in Results.

For peer review only

Table 3 The latent factors of antibiotic prescribing

Factor	Factor label	Number of items	Original item #	Loadings range	Reliability (Cronbach's α)
**1**	Awareness of AMS activities	8	33-40	0.5202, 0.7374	0.8734
**2**	Awareness of AMS purposes	5	28-32	0.6224, 0.7744	0.8334
**3**	Views regarding rational antibiotic prescribing	5	11, 17-20	0.3499, 0.7362	0.6961
**4**	Confidence in antibiotic prescribing decisions	8	12, 16, 21, 22, 24-27	0.4031, 0.7092	0.6997
**5**	Perception of AMR as a significant problem	6	1-6	0.4766, 0.5742	0.6967
**6**	Immediate actions to contain AMR	6	7, 8, 13-15, 23	0.3617, 0.6368	0.5695

Abbreviations: AMR, antimicrobial resistance; AMS, antibiotic stewardship.
Item #9 and 10 were excluded from the analysis, as explained in Results.
The full table is included in Table S2.

For peer review only

Table 4 Physician subgroup analysis

	Factor 1			Factor 2			Factor 3			Factor 4			Factor 5			Factor 6		
	Coeff	95% CI	P	Coeff	95% CI	P	Coeff	95% CI	P	Coeff	95% CI	P	Coeff	95% CI	P	Coeff	95% CI	P
**Hospital**																		
**05**	Ref			Ref			Ref			Ref			Ref			Ref		
**01**	0.194	-0.089, 0.477	0.179	**-0.422**	**-0.701, -0.142**	**0.003**	0.244	-0.039, 0.527	0.091	-0.167	-0.451, 0.117	0.248	**-0.430**	**-0.716, -0.145**	**0.003**	**-0.504**	**-0.778, -0.23**	**0.000**
**02**	0.064	-0.13, 0.258	0.518	**-0.352**	**-0.543, -0.161**	**0.000**	0.134	-0.06, 0.328	0.174	-0.180	-0.375, 0.014	0.069	-0.095	-0.29, 0.101	0.342	-0.087	-0.274, 0.101	0.366
**03**	0.164	-0.116, 0.444	0.252	-0.066	-0.343, 0.211	0.641	**0.387**	**0.107, 0.668**	**0.007**	0.131	-0.151, 0.412	0.363	-0.044	-0.327, 0.238	0.758	0.092	-0.18, 0.364	0.507
**04**	0.117	-0.218, 0.453	0.494	0.116	-0.215, 0.448	0.492	0.096	-0.239, 0.432	0.573	0.249	-0.088, 0.586	0.148	-0.081	-0.419, 0.257	0.638	**0.361**	**0.035, 0.686**	**0.030**
**06**	0.196	-0.153, 0.544	0.271	0.106	-0.239, 0.45	0.548	**0.419**	**0.07, 0.768**	**0.019**	0.013	-0.338, 0.363	0.944	**-0.432**	**-0.783, -0.08**	**0.016**	**0.479**	**0.141, 0.817**	**0.005**
**Departments**																		
**Surgical**	Ref			Ref			Ref			Ref			Ref			Ref		
**Medical**	**0.239**	**0.073, 0.405**	**0.005**	0.150	-0.014, 0.314	0.073	-0.120	-0.286, 0.046	0.156	**0.307**	**0.140, 0.473**	**0.000**	**0.221**	**0.054, 0.389**	**0.009**	**-0.398**	**-0.559, -0.237**	**0.000**
**Acute**	**-0.275**	**-0.488, -0.062**	**0.011**	**-0.214**	**-0.425, -0.004**	**0.046**	**-0.273**	**-0.486, -0.06**	**0.012**	0.169	-0.045, 0.382	0.122	0.124	-0.091, 0.338	0.258	**0.242**	**0.036, 0.449**	**0.021**
**Other**	-0.081	-0.253, 0.092	0.358	-0.027	-0.197, 0.144	0.760	-0.086	-0.259, 0.086	0.326	0.046	-0.127, 0.219	0.603	0.119	-0.055, 0.292	0.181	**-0.302**	**-0.469, -0.135**	**0.000**
**Medical**	Ref			Ref			Ref			Ref			Ref			Ref		
**Acute**	**-0.514**	**-0.745, -0.283**	**0.000**	**-0.364**	**-0.593, -0.136**	**0.002**	-0.153	-0.384, 0.078	0.194	-0.138	-0.370, 0.094	0.243	-0.098	-0.330, 0.135	0.411	**0.640**	**0.417, 0.864**	**0.000**
**Other**	**-0.320**	**-0.507, -0.133**	**0.001**	-0.177	-0.361, 0.0083	0.061	0.034	-0.154, 0.221	0.724	**-0.261**	**-0.449, 0.073**	**0.006**	-0.103	-0.291, 0.086	0.285	0.096	-0.085, 0.277	0.298
**Medical Hierarchy**																		
**Consultant**	Ref			Ref			Ref			Ref			Ref			Ref		
**Intern**	**-0.744**	**-1.374, -0.115**	**0.020**	**0.637**	**0.015, 1.259**	**0.045**	0.443	-0.187, 1.074	0.168	0.067	-0.565, 0.699	0.836	0.495	-0.14, 1.129	0.126	0.003	-0.607, 0.613	0.993
**GP**	0.168	-0.096, 0.432	0.212	0.153	-0.108, 0.413	0.251	**-0.397**	**-0.661, -0.133**	**0.003**	-0.169	-0.434, 0.095	0.210	-0.011	-0.276, 0.255	0.938	-0.024	-0.279, 0.232	0.857
**Resident**	-0.091	-0.287, 0.105	0.363	**0.297**	**0.104, 0.490**	**0.003**	0.004	-0.192, 0.199	0.971	**-0.271**	**-0.468, -0.075**	**0.007**	0.058	-0.139, 0.255	0.563	**-0.202**	**-0.391, -0.012**	**0.037**
**Other**	-0.013	-0.491, 0.466	0.958	0.148	-0.325, 0.620	0.541	0.252	-0.227, 0.731	0.303	-0.172	-0.653, 0.308	0.482	-0.013	-0.495, 0.469	0.959	0.304	-0.159, 0.768	0.198
**Intern**	Ref			Ref			Ref			Ref			Ref			Ref		
**GP**	**0.912**	**0.245, 1.579**	**0.007**	-0.485	-1.144, 0.175	0.150	-0.840	-1.508, -0.172	0.014	-0.236	-0.906, 0.434	0.489	-0.505	-1.18, 0.167	0.141	-0.0263	-0.673, 0.620	0.936
**Resident**	**0.653**	**0.0458, 1.261**	**0.035**	-0.340	-0.941, -0.260	0.267	-0.440	-1.048, 0.169	0.157	-0.338	-0.948, 0.272	0.278	-0.436	-1.049, 0.176	0.162	-0.205	-0.793, 0.384	0.496
**Other**	0.732	-0.457, 1.509	0.065	-0.490	-1.258, 0.279	0.212	-0.191	-0.970, 0.587	0.630	-0.239	-1.020, 0.542	0.548	-0.507	-1.291, 0.276	0.204	0.302	-0.452, 1.055	0.433
**Resident**	Ref			Ref			Ref			Ref			Ref			Ref		
**GP**	0.259	-0.037, 0.555	0.086	-0.145	-0.437, 0.148	0.333	-0.400	-0.696, -0.104	0.008	0.102	-0.196, 0.399	0.503	-0.069	-0.367, 0.229	0.651	0.178	-0.108, 0.465	0.223
**Other**	0.0781	-0.417, 0.574	0.757	-0.150	-0.639, 0.340	0.550	0.248	-0.248, 0.744	0.326	0.099	-0.399, 0.596	0.697	-0.710	-0.570, 0.428	0.780	**0.506**	**-0.163, 1.29**	**0.039**
**Years in current profession**																		
**0-5**	Ref			Ref			Ref			Ref			Ref			Ref		
**6-10**	0.089	-0.122, 0.299	0.410	0.225	**0.017, 0.434**	**0.034**	-0.089	-0.300, 0.122	0.410	0.094	-0.118, 0.305	0.385	**0.290**	**0.077, 0.502**	**0.008**	0.053	-0.151, 0.258	0.608
**11-15**	0.087	-0.185, 0.358	0.532	-0.015	-0.283, 0.254	0.915	-0.066	-0.338, 0.207	0.637	0.271	-0.002, 0.544	0.052	0.156	-0.118, 0.43	0.265	-0.095	-0.358, 0.169	0.481

For peer review only - http://bmjopen.bmj.com/site/about/guidelines.xhtml

	Factor 1			Factor 2			Factor 3			Factor 4			Factor 5			Factor 6		
	**Coeff**	**95% CI**	**P**	**Coeff**	**95% CI**	**P**	**Coeff**	**95% CI**	**P**	**Coeff**	**95% CI**	**P**	**Coeff**	**95% CI**	**P**	**Coeff**	**95% CI**	**P**
**>15**	0.117	-0.118, 0.353	0.328	0.194	-0.039, 0.427	0.102	-0.044	-0.279, 0.192	0.715	-0.057	-0.293, 0.179	0.637	**0.333**	**0.096, 0.57**	**0.006**	**0.366**	**0.138, 0.594**	**0.002**
**Public sector***	-0.064	-0.4, 0.273	0.710	0.264	-0.207, 0.736	0.272	-0.238	-0.575, 0.100	0.167	0.256	-0.835, 0.595	0.140	0.192	-0.148, 0.533	0.268	0.362	0.273, 0.996	0.264
**Tertiary level***	-0.093	-0.42, 0.234	0.577	-0.289	-0.767, 0.190	0.237	-0.047	-0.375, 0.281	0.779	-0.317	-0.647, 0.012	0.059	0.052	-0.279, 0.383	0.758	-0.407	-1.065, 0.25	0.225

Abbreviations: GP, general practitioner.
The table summarizes the results of the multivariable mixed-effect linear regression models to assess the associations between independent variables and each of the factor scores (dependent variable), while adjusting for the possible interdependence of observations clustered within hospitals as well as confounders (sex, AMS training).
* This model did not include hospital as an independent variable due to collinearity.

For peer review only

Figure 1. Five-point Likert scale responses for the 40-item questionnaire

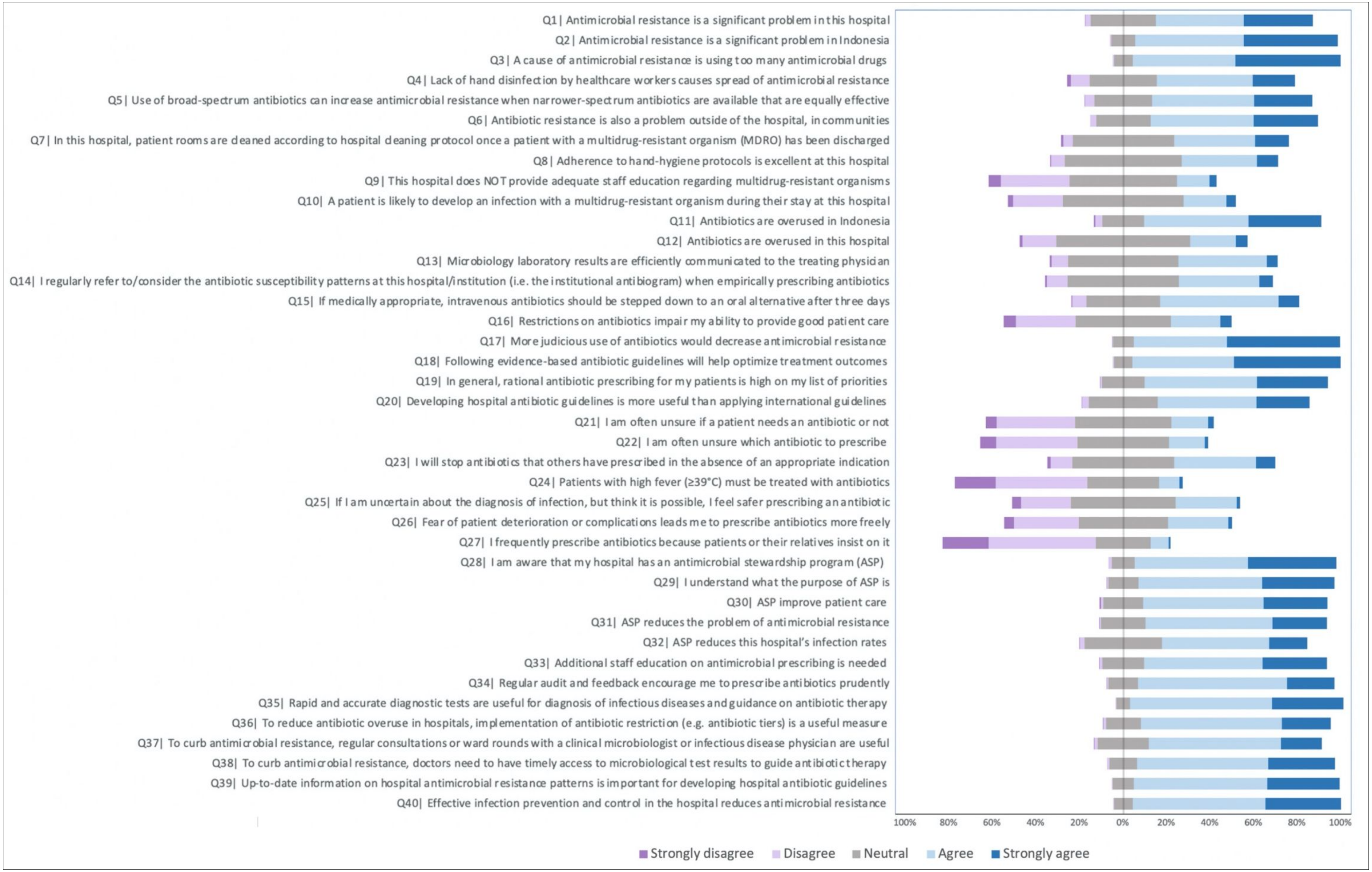

Acronyms: ASP, antibiotic stewardship program. Data (n, %) are summarized in Table S3.

Table S1 Survey response rate, overall and by hospital, department and professional hierarchy

	Total physicians invited	Total physicians responded	Response rate (%)	Respondents per physician type				
				Specialist/ Consultant	Resident	General practitioner	Intern/Internship doctor	Other
**Total**	**1896**	**1007**	**53.1%**	**358 (35.6%)**	**500 (49.7%)**	**113 (11.2%)**	**10 (1.0%)**	**18 (1.8%)**
**Hospital [a]**								
01	101 (5.3%)	70 (7.0%)	69.3%	39 (10.9%)	0 (0.0%)	27 (23.9%)	0 (0.0%)	4 (22.2%)
02	255 (13.4%)	155 (15.4%)	60.8%	70 (19.6%)	52 (10.4%)	26 (23.0%)	0 (0.0%)	5 (27.8%)
03	143 (7.5%)	77 (7.7%)	53.9%	41 (11.5%)	0 (0.0%)	35 (31.0%)	0 (0.0%)	1 (5.6%)
04	55 (2.9%)	44 (4.4%)	80.0%	29 (8.1%)	0 (0.0%)	11 (9.7%)	0 (0.0%)	1 (5.6%)
05	1240 (65.4%)	622 (61.8%)	50.2%	152 (42.5%)	448 (89.6%)	4 (3.5%)	10 (100.0%)	5 (27.8%)
06	102 (5.4%)	39 (3.9%)	38.2%	27 (7.5%)	0 (0.0%)	10 (8.9%)	0 (0.0%)	2 (11.1%)
**Sector [b]**								
Private	346	186 (18.5%)	53.8%	107 (29.9%)	0 (0.0%)	0 (0.0%)	72 (63.7%)	7 (38.9%)
Public	1550	821 (81.5%)	53.0%	251 (70.1%)	500 (100.0%)	41 (36.3%)	10 (100.0%)	11 (61.1%)
**Level of care [c]**								
Secondary	1499	777 (77.2%)	51.8%	136 (38.0%)	0 (0.0%)	83 (73.5%)	0 (0.0%)	8 (44.4%)
Tertiary	397	230 (22.8%)	57.9%	222 (62.0%)	500 (100.0%)	30 (26.6%)	10 (100.0%)	10 (55.6%)
**Department [d]**								
Surgery and surgical subspecialties	**628**	***371 (36.8)***	**59.1%**	***91 (24.5%)***	***226 (60.9%)***	***1 (0.27%)***	***4 (1.1%)***	***0 (0.0%)***
Obstetrics/gynaecology	229	146 (14.5%)	63.8%	51 (14.3%)	91 (18.2%)	1 (0.88%)	1 (10.0%)	0 (0.0%)
Surgery	210	122 (12.1%)	58.1%	2 (11.1%)	74 (14.8%)	0 (0.0%)	3 (30.0%)	0 (0.0%)
Orthopaedics	72	57 (5.7%)	44.4%	25 (7.0%)	32 (6.4%)	0 (0.0%)	0 (0.0%)	0 (0.0%)
Ear-nose-throat	74	32 (3.2%)	77.0%	12 (3.4%)	19 (3.8%)	0 (0.0%)	0 (0.0%)	0 (0.0%)
Urology	43	14 (1.4%)	32.6%	1 (1.1%)	10 (2.0%)	0 (0.0%)	0 (0.0%)	0 (0.0%)
Medicine and medical subspecialties	**456**	***232 (23.0%)***	**50.9%**	***115 (49.6%)***	***109 (47.0%)***	***0 (0.0%)***	***3 (1.3%)***	***5 (2.2%)***
Internal medicine	261	128 (12.7%)	49.0%	59 (16.5%)	67 (13.4%)	0 (0.0%)	0 (0.0%)	2 (11.1%)
Neurology	74	63 (6.3%)	85.1%	17 (4.8%)	40 (8.0%)	0 (0.0%)	3 (30.0%)	3 (16.7%)
Pulmonology	21	15 (1.5%)	71.4%	15 (4.2%)	0 (0.0%)	0 (0.0%)	0 (0.0%)	0 (0.0%)
Dermato-venerology	77	14 (1.4%)	18.2%	12 (0.0%)	2 (3.4%)	0 (0.0%)	0 (0.0%)	0 (0.0%)
Cardiology	23	12 (1.2%)	52.2%	12 (3.4%)	0 (0.0%)	0 (0.0%)	0 (0.0%)	0 (0.0%)
Acute specialties [e]	***96***	***156 (15.5)***	***162.5%***	***28 (17.9%)***	***67 (42.9%)***	***68 (43.6%)***	***2 (1.3%)***	***1 (0.64%)***
Anaesthesiology	96	72 (7.2%)	75.0%	4 (1.1%)	66 (13.2%)	0 (0.0%)	2 (20.0%)	0 (0.0%)

For peer review only

	Total physicians invited	Total physicians responded	Response rate (%)	Respondents per physician type				
				Specialist/ Consultant	Resident	General practitioner	Intern/Internship doctor	Other
Emergency dept [e]	-	57 (5.7%)	-	0 (0.0%)	0 (0.0%)	57 (50.4%)	0 (0.0%)	0 (0.0%)
Intensive Care Unit [e]	-	27 (2.7%)	-	14 (3.9%)	1 (0.2%)	11 (9.7%)	0 (0.0%)	1 (5.6%)
Other departments	**539**	***244 (24.2)***	**45.2%**	***90 (36.9%)***	***98 (40.2%)***	***24 (9.8%)***	***1 (0.41%)***	***10 (4.1%)***
Paediatrics	202	54 (5.4%)	26.7%	28 (7.8%)	25 (5.0%)	0 (0.0%)	0 (0.0%)	0 (0.0%)
Ophthalmology	102	39 (3.9%)	38.2%	22 (6.2%)	17 (3.4%)	0 (0.0%)	0 (0.0%)	0 (0.0%)
Multiple/unspecified units [e]	-	33 (3.3%)	-	5 (1.4%)	5 (1.0%)	21 (18.6%)	1 (10.0%)	0 (0.0%)
Rehabilitation	70	32 (3.2%)	45.7%	4 (1.1%)	28 (5.6%)	0 (0.0%)	0 (0.0%)	0 (0.0%)
Psychiatry	54	30 (3.0%)	55.6%	11 (3.1%)	19 (3.8%)	0 (0.0%)	0 (0.0%)	0 (0.0%)
Dentist	49	27 (2.7%)	55.1%	11 (3.1%)	1 (0.20%)	3 (2.7%)	0 (0.0%)	10 (55.6%)
Other	39	25 (2.5%)	64.1%	4 (1.1%)	1 (0.20%)	20 (17.7%)	0 (0.0%)	0 (0.0%)
Microbiology	4	3 (0.30%)	75.0%	2 (0.56%)	1 (0.20%)	0 (0.0%)	0 (0.0%)	0 (0.0%)
Laboratory	19	1 (0.10%)	5.3%	1 (0.28%)	0 (0.0%)	0 (0.0%)	0 (0.0%)	0 (0.0%)
Missing	-	4 (0.40%)	-	2 (0.56%)	1 (0.20%)	0 (0.0%)	0 (0.0%)	0 (0.0%)

Data are reported as n (%).
Doctor type was missing for 8 respondents, across [a] hospitals (02 (2), 03 (3), and 05 (3)); [b] sector (public 8, private 0); [c] level of care (secondary 3, tertiary 5); and [d] departments (obstetrics/gynaecology 2; ENT 1; paediatrics 1; multiple units 1; dental/oral surgery 2; missing dept 1).
[e] Invitations to participate in the survey were sent out according to primary work units; the discrepancies reported for the acute and other departments result from the fact that n=177 respondents who worked across multiple units (mostly GPs and internship doctors) indicated a different primary department on their questionnaire.

For peer review only

Table S2 The latent factors of antibiotic prescribing (full table)

Factor	Factor label	Survey questions included in the factor loading	Loadings	Reliability (Cronbach's α)
**1**	Awareness of AMS activities	Q33: Additional staff education on antimicrobial prescribing is needed	0.5202	0.8734
		Q34: Regular audit and feedback encourage me to prescribe antibiotics prudently	0.6151	
		Q35: Rapid and accurate diagnostic tests are useful for diagnosis of infectious diseases and guidance on antibiotic therapy	0.6714	
		Q36: To reduce antibiotic overuse in hospitals, implementation of antibiotic restriction (e.g. antibiotic tiers) is a useful measure	0.6428	
		Q37: To curb antimicrobial resistance, regular consultations or ward rounds with a clinical microbiologist or infectious disease physician are useful	0.7046	
		Q38: To curb antimicrobial resistance, doctors need to have timely access to microbiological test results to guide antibiotic therapy	0.7197	
		Q39: Up-to-date information on hospital antimicrobial resistance patterns is important for developing hospital antibiotic guidelines	0.7374	
		Q40: Effective infection prevention and control in the hospital reduces antimicrobial resistance	0.7067	
**2**	Awareness of AMS purposes	Q28: I am aware that my hospital has an antimicrobial stewardship program (ASP)	0.6224	0.8334
		Q29: I understand what the purpose of ASP is	0.6957	
		Q30: ASP improve patient care	0.7744	
		Q31: ASP reduces the problem of antimicrobial resistance	0.7532	
		Q32: ASP reduces this hospital's infection rates	0.6670	
**3**	Views regarding rational antibiotic prescribing	Q11: Antibiotics are overused in Indonesia	0.5138	0.6961
		Q17: More judicious use of antibiotics would decrease antimicrobial resistance	0.7362	
		Q18: Following evidence-based antibiotic guidelines will help optimize treatment outcomes	0.6565	
		Q19: In general, rational antibiotic prescribing for my patients is high on my list of priorities	0.5246	
		Q20: Developing hospital antibiotic guidelines is more useful than applying international guidelines	0.3499	
**4**	Confidence in antibiotic prescribing decisions	Q12: Antibiotics are overused in this hospital	-0.4550	0.6997
		Q16: Restrictions on antibiotics impair my ability to provide good patient care	0.4031	
		Q21: I am often unsure if a patient needs an antibiotic or not	0.5640	
		Q22: I am often unsure which antibiotic to prescribe	0.5670	
		Q24: Patients with high fever (≥39°C) must be treated with antibiotics	0.4794	

For peer review only

Factor	Factor label	Survey questions included in the factor loading	Loadings	Reliability (Cronbach's α)
		Q25: If I am uncertain about the diagnosis of infection, but think it is possible, I feel safer prescribing an antibiotic	0.6741	
		Q26: Fear of patient deterioration or complications leads me to prescribe antibiotics more freely	0.7092	
		Q27: I frequently prescribe antibiotics because patients or their relatives insist on it	0.6318	
**5**	Perception of AMR as a significant problem	Q1: Antimicrobial resistance is a significant problem in this hospital	0.5701	0.6967
		Q2: Antimicrobial resistance is a significant problem in Indonesia	0.5742	
		Q3: A cause of antimicrobial resistance is using too many antimicrobial drugs	0.5361	
		Q4: Lack of hand disinfection by healthcare workers causes spread of antimicrobial resistance	0.5725	
		Q5: Use of broad-spectrum antibiotics can increase antimicrobial resistance when narrower-spectrum antibiotics are available that are equally effective	0.5480	
		Q6: Antibiotic resistance is also a problem outside of the hospital, in communities	0.4766	
**6**	Immediate actions to contain AMR	Q7: In this hospital, patient rooms are cleaned according to hospital cleaning protocol once a patient with a multidrug-resistant organism (MDRO) has been discharged	0.6058	0.5695
		Q8: Adherence to hand-hygiene protocols is excellent at this hospital	0.6368	
		Q13: Microbiology laboratory results are efficiently communicated to the treating physician	0.5116	
		Q14: I regularly refer to/consider the antibiotic susceptibility patterns at this hospital/institution (i.e. the institutional antibiogram) when empirically prescribing antibiotics	0.6115	
		Q15: If medically appropriate, intravenous antibiotics should be stepped down to an oral alternative after three days	0.3617	
		Q23: I will stop antibiotics that others have prescribed in the absence of an appropriate indication	0.3892	

Abbreviations: AMS, antibiotic stewardship; ASP, antibiotic stewardship programme; IPC, infection prevention and control
Data are summarised in Table 3
Item #9 and 10 were excluded from the analysis, as explained in Results.

For peer review only

Table S3. Five-point Likert scale responses for the 40-item questionnaire

Item #	No of respondents	Strongly disagree (1)	Disagree (2)	Neutral (3)	Agree (4)	Strongly agree (5)
Q1	1005	3 (0.30)	26 (2.6)	168 (16.7)	452 (45.0)	356 (35.4)
Q2	1006	0 (0.0)	7 (0.70)	55 (5.5)	505 (50.2)	439 (43.6)
Q3	1003	0 (0.0)	6 (0.60)	43 (4.3)	472 (47.1)	482 (48.1)
Q4	1005	19 (1.9)	98 (9.8)	173 (17.2)	497 (49.5)	218 (21.7)
Q5	1004	2 (0.20)	49 (4.9)	145 (14.4)	514 (51.2)	294 (29.3)
Q6	1003	0 (0.0)	29 (2.9)	136 (13.6)	516 (51.5)	322 (32.1)
Q7	1004	12 (1.2)	53 (5.3)	288 (28.7)	460 (45.8)	191 (19.0)
Q8	1002	6 (0.60)	81 (8.1)	345 (34.4)	444 (44.3)	126 (12.6)
Q9	1005	71 (7.1)	397 (39.5)	311 (31.0)	188 (18.7)	38 (3.8)
Q10	1005	32 (3.2)	299 (29.8)	363 (36.1)	256 (25.5)	55 (5.5)
Q11	1005	8 (0.80)	34 (3.4)	102 (10.2)	506 (50.4)	355 (35.3)
Q12	1005	18 (1.8)	213 (21.2)	448 (44.5)	286 (28.5)	71 (7.1)
Q13	1005	11 (1.1)	97 (9.7)	321 (31.9)	515 (51.2)	61 (6.1)
Q14	1005	12 (1.2)	121 (12.0)	324 (32.2)	468 (46.6)	80 (8.0)
Q15	1006	7 (0.70)	73 (7.3)	195 (19.4)	623 (61.9)	108 (10.7)
Q16	1006	67 (6.7)	334 (33.2)	267 (26.5)	276 (27.4)	62 (6.2)
Q17	1005	2 (0.20)	2 (0.20)	48 (4.8)	431 (42.9)	522 (51.9)
Q18	1006	0 (0.0)	6 (0.60)	42 (4.2)	467 (46.4)	491 (48.8)
Q19	1005	1 (0.10)	7 (0.70)	105 (10.5)	545 (54.3)	347 (34.5)
Q20	1004	3 (0.30)	34 (3.4)	180 (17.9)	511 (50.9)	276 (27.5)
Q21	1006	60 (6.0)	440 (43.7)	269 (26.7)	204 (20.3)	33 (3.3)
Q22	1006	89 (8.9)	448 (44.5)	254 (25.3)	196 (19.5)	19 (1.9)
Q23	1002	19 (1.9)	124 (12.4)	288 (28.7)	464 (46.3)	107 (10.7)
Q24	1005	213 (21.2)	480 (47.8)	189 (18.8)	104 (10.4)	19 (1.9)
Q25	1005	51 (5.1)	285 (28.4)	301 (30.0)	348 (34.6)	20 (2.0)
Q26	1006	55 (5.5)	354 (35.2)	245 (24.4)	330 (32.8)	22 (2.2)
Q27	1005	231 (22.3)	538 (53.5)	138 (13.7)	90 (9.0)	8 (0.80)
Q28	1006	0 (0.0)	15 (1.5)	54 (5.4)	526 (52.3)	411 (40.9)
Q29	1006	2 (0.20)	6 (0.60)	71 (7.1)	585 (58.2)	342 (34.0)
Q30	1006	7 (0.70)	11 (1.1)	97 (9.6)	582 (57.9)	309 (30.7)
Q31	1005	2 (0.20)	7 (0.70)	109 (10.89)	620 (61.7)	267 (26.6)

For peer review only

Item #	No of respondents	Strongly disagree (1)	Disagree (2)	Neutral (3)	Agree (4)	Strongly agree (5)
Q32	1004	3 (0.30)	25 (2.5)	206 (20.5)	568 (56.6)	202 (20.1)
Q33	1005	2 (0.20)	14 (1.4)	101 (10.1)	574 (57.1)	314 (31.2)
Q34	1006	1 (0.10)	7 (0.70)	70 (7.0)	702 (69.8)	226 (22.5)
Q35	1006	0 (0.0)	4 (0.40)	31 (3.1)	646 (64.2)	325 (32.3)
Q36	1007	1 (0.10)	13 (1.3)	83 (8.2)	675 (67.0)	235 (23.3)
Q37	1006	2 (0.20)	17 (1.7)	128 (12.7)	657 (65.3)	202 (20.1)
Q38	1006	0 (0.0)	10 (0.99)	66 (6.6)	616 (61.2)	314 (31.2)
Q39	1006	0 (0.0)	4 (0.40)	49 (4.9)	617 (61.3)	336 (33.4)
Q40	1006	0 (0.0)	4 (0.40)	43 (4.3)	612 (60.8)	347 (34.5)

Data are also shown in Figure 1.

For peer review only

Figure S1 Scree plot showing eigenvalues for the 40 factors

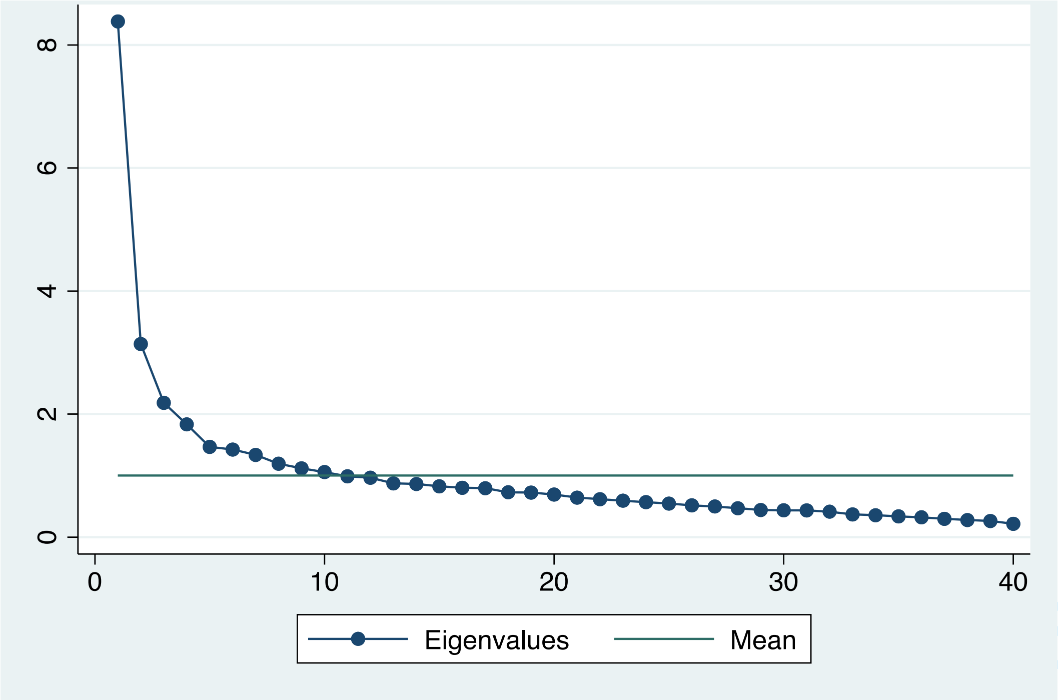

Figure S2 Parallel analysis showing adjusted eigenvalues for the 40 factors.

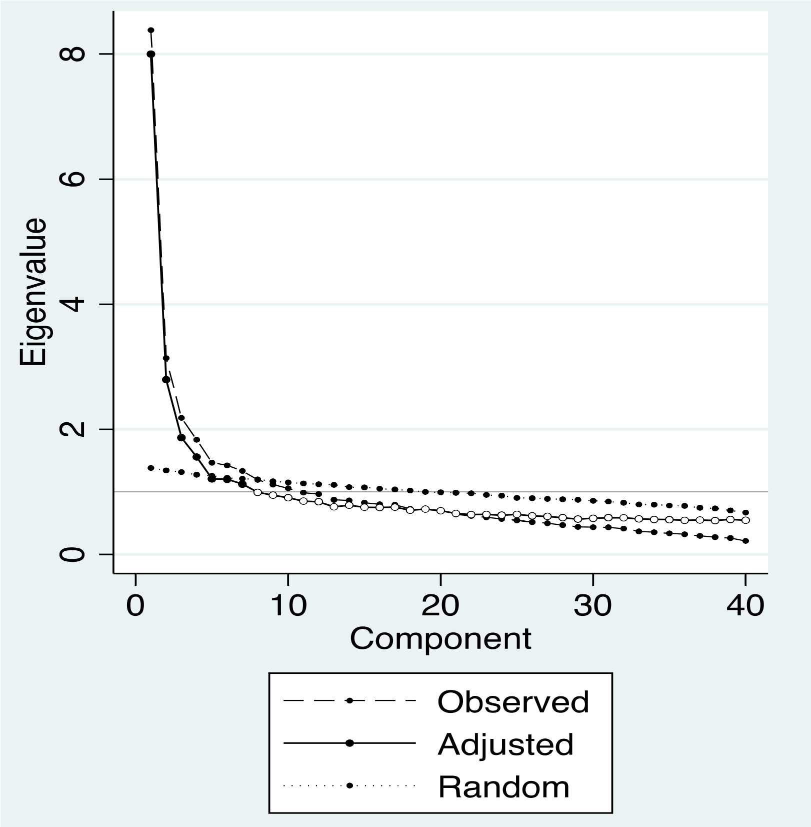

Parallel analysis adjusted the original eigenvalues for sampling error-induced collinearity among the variables to arrive at the adjusted eigenvalues.

STROBE Statement—

Checklist of items that should be included in reports of ***cross-sectional studies***

	Item No	Recommendation	Page No
**Title and abstract**	1	(*a*) Indicate the study's design with a commonly used term in the title or the abstract	1
		(*b*) Provide in the abstract an informative and balanced summary of what was done and what was found	2
**Introduction**			
Background/rationale	2	Explain the scientific background and rationale for the investigation being reported	4
Objectives	3	State specific objectives, including any prespecified hypotheses	4
**Methods**			
Study design	4	Present key elements of study design early in the paper	5
Setting	5	Describe the setting, locations, and relevant dates, including periods of recruitment, exposure, follow-up, and data collection	5
Participants	6	(*a*) Give the eligibility criteria, and the sources and methods of selection of participants	5
Variables	7	Clearly define all outcomes, exposures, predictors, potential confounders, and effect modifiers. Give diagnostic criteria, if applicable	6
Data sources/ measurement	8*	For each variable of interest, give sources of data and details of methods of assessment (measurement). Describe comparability of assessment methods if there is more than one group	6
Bias	9	Describe any efforts to address potential sources of bias	6
Study size	10	Explain how the study size was arrived at	7
Quantitative variables	11	Explain how quantitative variables were handled in the analyses. If applicable, describe which groupings were chosen and why	7
Statistical methods	12	(*a*) Describe all statistical methods, including those used to control for confounding	7
		(*b*) Describe any methods used to examine subgroups and interactions	7
		(*c*) Explain how missing data were addressed	7
		(*d*) If applicable, describe analytical methods taking account of sampling strategy	7
		(*e*) Describe any sensitivity analyses	NA
**Results**			
Participants	13*	(a) Report numbers of individuals at each stage of study—eg numbers potentially eligible, examined for eligibility, confirmed eligible, included in the study, completing follow-up, and analysed	8
		(b) Give reasons for non-participation at each stage	8
		(c) Consider use of a flow diagram	NA

For peer review only

Descriptive data	14*	(a) Give characteristics of study participants (eg demographic, clinical, social) and information on exposures and potential confounders	8
		(b) Indicate number of participants with missing data for each variable of interest	8-10
Outcome data	15*	Report numbers of outcome events or summary measures	8-11
Main results	16	(*a*) Give unadjusted estimates and, if applicable, confounder-adjusted estimates and their precision (eg, 95% confidence interval). Make clear which confounders were adjusted for and why they were included	8-11
		(*b*) Report category boundaries when continuous variables were categorized	8-11
		(*c*) If relevant, consider translating estimates of relative risk into absolute risk for a meaningful time period	NA
Other analyses	17	Report other analyses done—eg analyses of subgroups and interactions, and sensitivity analyses	10
**Discussion**			
Key results	18	Summarise key results with reference to study objectives	11-12
Limitations	19	Discuss limitations of the study, taking into account sources of potential bias or imprecision. Discuss both direction and magnitude of any potential bias	12
Interpretation	20	Give a cautious overall interpretation of results considering objectives, limitations, multiplicity of analyses, results from similar studies, and other relevant evidence	13
Generalisability	21	Discuss the generalisability (external validity) of the study results	12
**Other information**			
Funding	22	Give the source of funding and the role of the funders for the present study and, if applicable, for the original study on which the present article is based	14

*Give information separately for exposed and unexposed groups.

Note: An Explanation and Elaboration article discusses each checklist item and gives methodological background and published examples of transparent reporting. The STROBE checklist is best used in conjunction with this article (freely available on the Web sites of PLoS Medicine at http://www.plosmedicine.org/, Annals of Internal Medicine at http://www.annals.org/, and Epidemiology at http://www.epidem.com/). Information on the STROBE Initiative is available at www.strobe-statement.org.

For peer review only - http://bmjopen.bmj.com/site/about/guidelines.xhtml

BMJ Open

Perceptions, views and practices regarding antibiotic prescribing and stewardship among hospital physicians in Jakarta, Indonesia: a questionnaire-based survey

Journal:	*BMJ Open*
Manuscript ID	bmjopen-2021-054768.R1
Article Type:	Original research
Date Submitted by the Author:	30-Dec-2021
Complete List of Authors:	Limato, Ralalicia; Oxford University, Eijkman-Oxford Clinical Research Unit; Oxford University, Nuffield Department of Medicine, Centre for Tropical Medicine and Global Health<br>Nelwan, Erni Juwita ; RSCM, Department of Internal Medicine, Division of Infectious Diseases; Universitas Indonesia, Faculty of Medicine<br>Mudia, Manzilina; Oxford University, Eijkman-Oxford Clinical Research Unit<br>Alamanda, Monik; Oxford University, Eijkman-Oxford Clinical Research Unit<br>Manurung, Elfrida Rinawaty; Royal Taruma Hospital<br>Mauleti, Ifael Yerosias; Fatmawati General Hospital<br>Mayasari, Maria; St. Carolus Hospital<br>Firmansyah, Iman; Prof. Dr. Sulianti Saroso Infectious Disease Hospital<br>Djaafar , Roswin ; Metropolitan Medical Centre Hospital<br>Vu, Huong; Oxford University Clinical Research Unit, Hanoi Unit<br>van Doorn, H. Rogier; Oxford University Clinical Research Unit; Oxford University, Nuffield Department of Medicine, Centre for Tropical Medicine and Global Health<br>Broom, Alex; The University of Sydney, Sydney Centre for Healthy Societies, School of Social and Political Sciences<br>Hamers, Raph Leonardus; Oxford University, Eijkman-Oxford Clinical Research Unit; Oxford University, Nuffield Department of Medicine, Centre for Tropical Medicine and Global Health
**Primary Subject Heading**:	Public health
Secondary Subject Heading:	Sociology, Public health, Global health
Keywords:	Public health < INFECTIOUS DISEASES, PUBLIC HEALTH, SOCIAL MEDICINE

SCHOLARONE™
Manuscripts

I, the Submitting Author has the right to grant and does grant on behalf of all authors of the Work (as defined in the below author licence), an exclusive licence and/or a non-exclusive licence for contributions from authors who are: i) UK Crown employees; ii) where BMJ has agreed a CC-BY licence shall apply, and/or iii) in accordance with the terms applicable for US Federal Government officers or employees acting as part of their official duties; on a worldwide, perpetual, irrevocable, royalty-free basis to BMJ Publishing Group Ltd ("BMJ") its licensees and where the relevant Journal is co-owned by BMJ to the co-owners of the Journal, to publish the Work in this journal and any other BMJ products and to exploit all rights, as set out in our licence.

The Submitting Author accepts and understands that any supply made under these terms is made by BMJ to the Submitting Author unless you are acting as an employee on behalf of your employer or a postgraduate student of an affiliated institution which is paying any applicable article publishing charge ("APC") for Open Access articles. Where the Submitting Author wishes to make the Work available on an Open Access basis (and intends to pay the relevant APC), the terms of reuse of such Open Access shall be governed by a Creative Commons licence – details of these licences and which Creative Commons licence will apply to this Work are set out in our licence referred to above.

Other than as permitted in any relevant BMJ Author's Self Archiving Policies, I confirm this Work has not been accepted for publication elsewhere, is not being considered for publication elsewhere and does not duplicate material already published. I confirm all authors consent to publication of this Work and authorise the granting of this licence.

For peer review only

Perceptions, views and practices regarding antibiotic prescribing and stewardship among hospital physicians in Jakarta, Indonesia: a questionnaire-based survey

Authors

Ralalicia Limato[1,2], Erni J Nelwan[3,4,5,6], Manzilina Mudia[1], Monik Alamanda[1], Elfrida R Manurung[7], Ifael Y Mauleti[8], Maria Mayasari[9], Iman Firmansyah[10], Roswin Djaafar[6], Huong Vu Thi Lan[11], H Rogier van Doorn[2,11], Alex Broom[12], Raph L Hamers[1,2,4*]

Affiliations

1. Eijkman-Oxford Clinical Research Unit, Jakarta, Indonesia
2. Centre for Tropical Medicine and Global Health, Nuffield Department of Medicine, University of Oxford, Oxford, UK
3. Department of Internal Medicine, Division of Infectious Diseases, Cipto Mangunkusumo National General Hospital, Jakarta, Indonesia
4. Faculty of Medicine, Universitas Indonesia, Jakarta, Indonesia
5. Infectious Disease and Immunology Research Cluster, Indonesian Medical Education and Research Institute, Jakarta, Indonesia
6. Metropolitan Medical Centre Hospital, Jakarta, Indonesia
7. Royal Taruma Hospital, Jakarta, Indonesia
8. Fatmawati General Hospital, Jakarta, Indonesia
9. St. Carolus Hospital, Jakarta, Indonesia
10. Prof. Dr. Sulianti Saroso Infectious Disease Hospital, Jakarta, Indonesia
11. Oxford University Clinical Research Unit, Hanoi, Vietnam
12. Sydney Centre for Healthy Societies, School of Social and Political Sciences, The University of Sydney, Sydney, Australia

***Corresponding author**

Raph L. Hamers MD PhD

Email : raph.hamers@ndm.ox.ac.uk

Phone : +62 21 31900971

Fax : -

Inserts: Tables 4; Figures 1

Appendix: survey questionnaire

Data supplement: Tables 3; Figures 2

For peer review only

Abstract

Objectives: Antibiotic overuse is one of the main drivers of antimicrobial resistance (AMR), especially in low and middle-income countries. This study aimed to understand the perceptions of antimicrobial resistance (AMR), antibiotic prescribing practice, and views on antimicrobial stewardship (AMS) among hospital physicians in Jakarta, Indonesia.

Design: cross-sectional, self-administered questionnaire-based survey, with descriptive statistics, exploratory factor analysis (EFA) to identify distinct underlying constructs in the dataset, and multivariable linear regression of factor scores to analyse physician subgroups.

Setting: Six public and private general hospitals in Jakarta in 2019.

Participants: 1007 of 1896 (53.1% response rate) antibiotic prescribing physicians.

Results: EFA identified six latent factors (overall Crohnbach's α=0.85): awareness of AMS activities; awareness of AMS purpose; views regarding rational antibiotic prescribing; confidence in antibiotic prescribing decisions; perception of AMR as a significant problem; and immediate actions to contain AMR. Physicians acknowledged the significance of AMR and contributing factors, rational antibiotic prescribing, and purpose and usefulness of AMS. However, this conflicted with reported suboptimal local hospital practices, such as room cleaning, hand hygiene and staff education, and views regarding antibiotic decision-making. These included insufficiently applying AMS principles and utilising microbiology, lack of confidence in prescribing decisions, and defensive prescribing due to pervasive diagnostic uncertainty, fear of patient deterioration or because patients insisted. Physicians' factor scores differed across hospitals, departments, work experience and medical hierarchy.

Conclusions: AMS implementation in Indonesian hospitals is challenged by institutional, contextual and diagnostic vulnerabilities, resulting in externalising AMR instead of recognising it as a local problem. Appropriate recognition of the contextual determinants of antibiotic prescribing decision-making will be critical to change physicians' attitudes and develop context-specific AMS interventions.

Keywords: perceptions; survey; antibiotic resistance; antibiotic prescribing; antibiotic stewardship; Indonesia

Strengths and limitations of this study

- The self-developed questionnaire in this study identified a relevant set of attributes through a factor analysis optimization process, with adequate content, face and construct validity and internal reliability. This study adds important value in the absence of adequately validated instruments regarding antimicrobial resistance and stewardship, with particular applicability for LMIC.
- This study had a large, varied respondent sample and high response rate among physicians at six public and private hospitals in Jakarta, Indonesia, and identified differences between physicians across hospitals, departments, work experience and medical hierarchy, which can guide priority-setting and tailoring of stewardship interventions.
- However, non-participation and the convenient hospital sample could have introduced selection bias, and the data are not necessarily representative for Jakarta or Indonesia.
- Factor analysis is based on using a "heuristic", which leaves room to more than one interpretation of the same data and cannot identify causality.

For peer review only

Introduction

The global rise in drug-resistant infections is one of the leading threats to public health globally, with increasing rates of morbidity, mortality and escalating healthcare costs[1]. Misuse and overuse of antimicrobial drugs in human health care is one of the main drivers[2,3] and also represents a key solution, i.e. judicious use of remaining antibiotics. Globally, use of antibiotics remains largely unrestrained and poorly governed, with large, unregulated healthcare systems representing an increasingly challenging area for achieving the goal of optimization. Substantial variations in contributing factors to inappropriate antibiotic prescribing exist across contexts, e.g. diagnostic uncertainty, pressure from pharmaceutical industry or patients[4,5], with the structure and funding of health systems inflecting enactment of optimization strategies, including antimicrobial stewardship (AMS)[6].

AMS programs aim to control antimicrobial use, and have been associated with reducing hospital-acquired infections, unnecessary healthcare costs, and potentially drug-resistant infections[7–9]. However, AMS programmes in turn may jar with local constraints and practices and have been shown to have limited traction when attempts to implement occur without adequate understanding of context[10].

The global push to enact effective AMS requires detailed, context-specific data on physicians, given their central role in the complex process of antibiotic prescribing in hospitals, which can inform on how AMR is conceived, how current prescribing is rationalised, and how broad AMS principles may be experienced across contexts and nations[11,12]. Few studies to date have been conducted on this topic in low and middle-income countries (LMIC), with insufficient evaluation of the psychometric properties of their measurement instruments to examine their suitability to the specific context[4,5].

Indonesia, a diverse middle-income country in Southeast Asia with the world's fourth largest population (275 million), is particularly vulnerable for AMR, ranked among the greatest risers (29th of 76 countries) in antibiotic consumption, estimated at 2.5-fold between 2000 and 2015[2]. The implementation of the National Action Plan for AMR (NAP) has been hindered due to, among other factors, a limited evidence base of AMR epidemiology, antibiotic utilization and rational prescribing practices[13]. Weakly enforced antibiotic policies promote inappropriate prescribing and over-the-counter access without a prescription, and AMS is generally in an early stage of implementation[13,14]. Although nationwide representative AMR data are not available, anecdotal evidence suggested high rates of AMR among common Gram-negative bacteria.

For peer review only

To identify context-specific opportunities for AMS interventions, we conducted a questionnaire-based survey among antibiotic-prescribing physicians in hospitals in Jakarta, Indonesia, to evaluate their perceptions of AMR, antibiotic prescribing practice, and views on AMS, and explore differences between physician subgroups. The study also aimed to evaluate the construct validity and psychometric properties of the questionnaire, and explore differences between physician subgroups.

Methods

Study design and setting

We conducted a cross-sectional survey between March and August 2019 among all antimicrobial prescribing physicians at six general hospitals in Jakarta, Indonesia, as part of a mixed-method study to identify targets for quality improvement in antibiotic prescribing practices (EXPLAIN study[15]). The hospitals were purposively sampled to achieve representation of various types, respondents and geographic locations within Jakarta, and size, and included two tertiary-care government hospitals and four secondary hospitals, three of which were private hospitals, with between 134 and 853 inpatient beds. At the time of the survey, all six hospitals had an AMS programme, albeit at various stages of AMS implementation, initiated between 2009 and 2018.

Study respondents

Study respondents were purposively sampled to cover all departments, work experience and professional hierarchy, to ensure representation of different antibiotic practice and views, perceptions, experiences related to AMR and AMS[16]. All qualified physicians prescribing antibiotics on a regular basis working across all clinical departments were eligible and approached to participate, including interns/internship doctors *(magang/dokter internsip;* recent graduates working under supervision of a specialist), general practitioners (GPs) (*dokter umum*; physicians who practice general medicine treating common, non-specialized medical conditions), residents (*residen*; physicians who are in specialist training), specialist/consultant physicians (*dokter spesialis/konsultan*; specialized in a particular area of medicine), and others.

Patient and public involvement statement

Patients or the public were not involved in the design, conduct, or reporting of the research.

Survey questionnaire

We developed a two-page anonymous, self-administered, paper-based questionnaire, which was easy to complete and based on a conceptual framework that included attributes related

to prescribers' perceptions, views and practices. Good practice recommendations for questionnaire design and existing questionnaires in the literature were reviewed and discussed with several experts. The Clinician Pre/Post Perception Survey of the Greater New York Hospital Association United Hospital Fund[17] constituted the initial set of items, supplemented with relevant items from other existing questionnaires[18–24]. From a preliminary pool, we selected 69 items, of which 8 items were worded in the negative to address the acquiescence effect. The instrument was translated from English to Indonesian, and back-translated by an independent translator. The questionnaire was pre-tested by a convenience panel of 18 physicians (2 GPs, 15 residents, and 1 consultant). According to their feedback, we made adjustments to clarify ambiguous items, and remove redundant items. The final version consisted of 40 items and took about 10 minutes to complete.

The final questionnaire included an explanation of study purpose and completion instructions; 40 short statements (items) to which participants were asked to indicate the extent to which each reflected their own opinion on a 5-point Likert scale, divided into 3 sections: scope of the AMR problem and key contributors; antibiotic prescribing practices; AMS; and respondent socio-demographics (**Appendix**).

Respondent recruitment

The hospital management provided the total number of prescribing physicians for each department. The questionnaires were delivered to the head of each unit who then distributed the survey to all eligible staff. The study coordinator kept a record of numbers of physicians approached and participated. Upon survey completion, respondents could enter a raffle to win one of three gift cards in each hospital (US$14 each); there were no other incentives for participation.

Ethical approval

The research ethics committee of the Faculty of Medicine University of Indonesia (1364/UN2.F1/ETIK/2018) and the Oxford Tropical Research Ethics Committee (559-18) approved the study, with additional permission from hospital management. As the survey was anonymous, consent was inferred when the participant completed and returned the questionnaire.

Statistical analysis

The percentage of respondents selecting each answer choice was calculated using the total number of responses as the denominator. For an EFA, a common lower bound for sample size is 10 cases per variable, suggesting a minimum sample size of 400; to allow for meaningful subgroup comparisons and minimize selection bias, we targeted a >50%

For peer review only

response rate and a sample size of >1000 across the six hospitals. We performed EFA to identify underlying distinct constructs, using factor, pcf command in Stata with orthogonal (varimax) rotation. For this analysis, the eight items worded in the negative were reverse-coded, and missing data for categorical variables were treated as a separate category. The Kaiser-Meyer-Olkin (KMO) was calculated to ensure EFA requirements were met. Each item was assigned to a certain factor based on the highest absolute factor loading of the rotated solution, and we then assigned an umbrella term to each factor. After the optimal factor solution had been achieved, we calculated factor scores using the regression scoring method, and Cronbach's α coefficient to test internal reliability. Using the factor scores (dependent variable), we developed six (one for each factor score) multivariable linear regression models to assess associations with key physician characteristics ("subgroups") (independent variables, i.e., hospital sector and care level, grouped departments, work experience and medical hierarchy). Given the hierarchical structure of the data (respondents in hospitals) we applied multilevel modelling to control for possible clustering within hospitals. P-values<0.05 were considered statistically significant. All analyses were done with Stata/IC Version 16.1 (StataCorp, College Station, TX, US).

For peer review only

Results

Respondent characteristics

All 1896 antibiotic prescribing physicians at the six hospitals were approached, and 1007 (53.1%) participated in the survey. **Table 1** summarizes the participants' key characteristics. **Table S1 in supplementary file** summarizes the response rates.

Exploratory factor analysis

The KMO was 0.8773 overall and >0.5 for all items, suggesting the data were suitable for EFA. Analysis of the scree plot (**supplementary file Figure S1**) indicated a case for four factors, whereas the parallel analysis (**supplementary file Figure S2**) indicated a case for seven factors. The four-factor solution yielded strong factors but explained only 39.9% of the variance and lacked a theoretical basis for one factor. The seven-factor solution contained one factor with only three items that was difficult to interpret; two of these items (Q9 and Q10) did not load well with any factor in various alternative factor solutions and were removed. Therefore, a six-factor model with a clear theoretical basis based on 38 items was deemed most suitable, explaining 47.4% of the variance, with KMO 0.8802 overall and >0.5 for each item (**Table 2**). The six latent factors are (**Table 3**, see **supplementary file Table S2** for details): 1) Awareness of AMS activities; 2) Awareness of AMS purpose; 3) Views regarding rational antibiotic prescribing; 4) Confidence in antibiotic prescribing decisions; 5) Perception of AMR as a significant problem; and 6) Immediate actions to contain AMR. Internal reliability was excellent for the overall 38-item scale (α=0.85) and factor 1 (α=0.8734) and 2 (α=0.8334), good for factor 3, 4 and 5 (α=0.70 each), and acceptable for factor 6 (α=0.57).

Description of respondent responses

Figure 1 and **Table S3 in supplementary file** summarize the responses to all 40 items.

Scope of the AMR problem and key contributors Most respondents agreed that AMR is an important problem in Indonesia (93.8%; 944/1006) [Q2]; in communities outside of the hospital (83.6%; 838/1003) [Q6]; and at their hospital (80.4%; 808/1005) [Q1], with 30.9% (311/1005) agreeing that patients are likely to develop an infection with a multidrug-resistant infection [Q10]. Most acknowledged as key contributing factors: overuse of antimicrobial drugs (95.1%; 954/1003) [Q3], lack of hand hygiene (71.1%; 715/1005) [Q4], use of broad-spectrum antibiotics (80.5%; 808/1004) [Q5]. Current infection and prevention control (IPC) practices at their hospital were regarded as suboptimal: 64.8% (651/1004) thought that patient rooms are cleaned according to hospital protocol after discharge of a patient with a

multidrug-resistant organism [Q7]; 56.9% (570/1002) thought adherence to hand-hygiene protocols to be excellent [Q8]; and 22.5% (226/1005) felt that their hospital does not provide adequate staff education regarding multidrug-resistant organisms [Q9].

Antibiotic prescribing practices Whereas most respondents (85.7%; 861/1005) agreed that antibiotics are overused in Indonesia (Q11), only 35.5% (357/1005) acknowledged this to be case at their hospital [Q12]. Most agreed that more judicious antimicrobial prescribing practices would decrease AMR (94.8%; 953/1005) [Q17] and that following evidence-based antibiotic guidelines will help optimize treatment outcomes (95.3%; 958/1006) [Q18]. Most gave high priority to rational antibiotic prescribing to their patients (88.8%; 892/1005) [Q19], and considered developing hospital antibiotic guidelines more useful than applying international guidelines (78.4%; 787/1004) [Q20]. Nearly a quarter of respondents indicated to be often unsure if a patient needs an antibiotic or not (23.6%; 237/1006) [Q21] or which antibiotic to prescribe (21.3%; 215/1006) [Q22]. A small but considerable fraction expressed lack of confidence in prescribing decisions, i.e., 12.2% (123/1005) prescribed in patients with just a high fever (≥39°C) [Q24], 36.6% (368/1005) when they felt uncertain about the diagnosis of infection [Q25], 35.0% (352/1006) prescribed more freely because of fear of clinical failure [Q26], and 9.8% (98/1005) frequently prescribed antibiotics because patients or their relatives insist [Q27]. Just more than half of the respondents reported that microbiology laboratory results are efficiently communicated to the treating physician (57.3%; 576/1005) [Q13], considered the hospital antibiogram when empirically prescribing antibiotics (54.5%; 548/1005) [Q14], and would stop antibiotics that others have prescribed in the absence of an appropriate indication (57.0%; 571/1002) [Q23]. Most (72.7%; 731/1006) agreed that, if medically appropriate, intravenous antibiotics should be stepped down to an oral alternative after three days [Q15]. Notably, 33.6% (338/1006) felt that restrictions on antibiotics impaired their ability to provide good patient care [Q16].

Antibiotic stewardship When asked about AMS in general, most respondents were aware that their hospital had an AMS programme (93.1%; 937/1006) [Q28], they reported they understood its purpose (92.1%; 927/1006) [Q29], and they agreed that AMS can improve patient care (88.6%; 891/1006) [Q30], reduce AMR (88.3%; 887/1005) [Q31] and hospital-acquired infections (76.7%; 770/1004) [Q32]. When asked about the usefulness of specific AMS activities, most respondents acknowledged that additional education on antibiotic prescribing was needed (88.4%; 888/1005) [Q33], regular audit and feedback encouraged them to prescribe antibiotics prudently (92.2%; 928/1006) [Q34], rapid and accurate diagnostic tests are useful for diagnosis of infectious diseases and guidance on antibiotic therapy (96.5%; 971/1006) [Q35], implementation of antibiotic restriction (e.g. antibiotic tiers) can reduce antibiotic overuse in hospitals (90.4%; 910/1007) [Q36], regular consultations or

For peer review only

ward rounds with a clinical microbiologist or infectious disease physician can curb AMR (85.4%; 859/1006) [Q37], timely access to microbiological test results is needed to guide antibiotic therapy (92.4%; 930/1006) [Q38], and IPC in the hospital can reduce AMR (95.3%; 959/1006) [Q40].

Physician subgroup analysis

Table 4 summarizes the results of the subgroup analysis.

Hospitals Statistically significant differences were identified between hospitals for awareness of AMS purposes (factor 2), views regarding rational antibiotic prescribing (factor 3), perception of AMR as a significant problem (factor 5) and immediate actions to contain AMR (factor 6), but not for awareness of AMS activities (factor 1) and confidence in antibiotic prescribing decisions (factor 4). None of the factor scores differed between prescribers in public versus private, or secondary versus tertiary hospitals.

Professional hierarchy For awareness of AMS activities (factor 1), consultants, GPs and residents scored higher than interns. For awareness of AMS purposes (factor 2), consultants scored lower than interns and residents. For views regarding rational antibiotic prescribing (factor 3), consultants scored higher than GPs. For confidence in antibiotic prescribing decisions (factor 4), consultants scored lower than residents, whereas for immediate actions to contain AMR (factor 6), consultants scored higher than residents. No differences were identified for perception of AMR as a significant problem (factor 5).

Departments For awareness of AMS activities (factor 1) and purpose (factor 2), physicians in surgery and medicine scored higher than the acute specialties, whereas for awareness of AMS activities (factor 1), medicine scored higher than surgery. For views regarding rational antibiotic prescribing (factor 3), surgery scored higher than acute specialties. For confidence in antibiotic prescribing decisions (factor 4), medicine scored lower than surgery and other specialties. For perception of AMR as a significant problem (factor 5), surgery scored lower than medicine. For immediate actions to contain AMR (factor 6), surgery scored higher than medicine and other specialties, and the acute specialties scored higher than surgery and medicine.

Work experience. Physicians with little (0-5 years) work experience scored lower than more experienced colleagues, for awareness of AMS purpose (factor 2); for perception of AMR as a significant problem (factor 5); and for immediate actions to contain AMR (factor 6). No differences were identified for factors 1, 3 and 4.

For peer review only

Discussion

This survey assessed the perceptions, views and practices regarding AMR, antibiotic prescribing and AMS among over 1000 physicians in Indonesian hospitals. Through an exploratory factor analysis, we identified six distinct constructs in the dataset, i.e., 1) awareness of AMS activities; 2) awareness of AMS purposes; 3) views regarding rational antibiotic prescribing; 4) confidence in antibiotic prescribing decisions; 5) perception of AMR as a significant problem; and 6) immediate actions to contain AMR.

The survey findings outline a series of dynamics around AMR and AMS in the Indonesian context. Spanning issues around *visibility* (diagnostics)[25], *awareness* (education)[26] and *institutional form* (governance)[27], the survey results tease out many of the core issues illustrated in other settings[4,5], but in turn, illustrate that several specific factors play a role, i.e. uncertainty, risk, and lack of sense of responsibility. For instance, only about one-third of physicians recognized that antibiotic overuse was an issue at their own hospital, many physicians were hesitant to stop antibiotics that others had prescribed in the absence of an appropriate indication, and felt that antibiotic restrictions impaired their ability to provide good patient care. Lack of confidence in prescribing decisions and defensive prescribing were common due to diagnostic uncertainty, fear of patient deterioration or complications, or because patients or their relatives insisted. The study findings expand on our recently published paper on the patterns and quality indicators of antibiotic prescribing in the same hospitals, which identified several priority areas for stewardship[15].

The most significant factor in guiding the future agenda in Indonesia around effective AMS implementation, is the perceived "externality of AMR" as a problem[28]. That is, physicians acknowledge its significance but do not take ownership or responsibility, thus reflecting a production of AMR as an externality, e.g., a result of irrational use elsewhere in communities or other hospitals. The lack of systematic surveillance of AMR and antibiotic use and the underutilisation of bacterial cultures, recognised by many of the respondents, also reproduces the perception of AMR as a "problem of elsewhere". This feeds a lack of engaging with AMS, as it is not recognised as a value-add for an already stretched institutional context, and in turn provides the context for continued defensive prescribing "to be on the safe side". Moreover, defensive prescribing practices somewhat offset (in the short term) problems around room cleaning, hand hygiene and staff education. In this way, AMR as an externality and the vulnerabilities of the institution, offer an environment conducive to the ongoing over-use of antimicrobials[29,30]. The higher incidence of hospital-acquired infections in LMIC than in high-income countries could further promote defensive prescribing as a way to compensate for substandard IPC practices[31]. All in all, this supports the notion

For peer review only

that physicians tend to prioritise managing immediate clinical risks, reputation and concordance with peer practice, vis-à-vis the long-term population consequences of AMR[32].

Although the survey identified several differences between physicians across the participating hospitals regarding awareness of AMS purposes, views regarding rational antibiotic prescribing, perception of AMR as a significant problem and immediate actions to contain AMR, these differences could not be attributed or related to the pertinent hospital sector (public versus private) or health care level (secondary versus tertiary).

Work experience and medical hierarchy were found to influence the awareness of AMS purpose, AMR as a significant problem, and immediate actions to contain AMR. Interestingly, compared with junior physicians, specialists/consultants expressed lower confidence to make antibiotic decisions in uncertain situations while showing higher confidence in actions to contain AMR. Possible explanations include that specialists/consultants have a better recognition of the “unknowns” (e.g. lack of data on bacterial susceptibility patterns) and that they bear final patient responsibility, introducing the fear of losing a patient or legal consequences[33], whereas taking actions to curb AMR can be a remedy to compensate their fear. Conversely, residents’ higher confidence in antibiotic prescribing may also relate to their contemporary medical training, which includes AMS, as opposed to late-career physicians[34]. GPs had low scores on views regarding rational antibiotic prescribing compared with consultants which could reflect the GPs’ limited responsibility in the antibiotic decision-making hierarchy, possibly leading to a lack of positive attitude towards guidelines and preference for complying with them[35,36].

The acute specialties (including emergency, ICU, and anaesthesiology) had lower awareness of AMS activities and purposes, compared with surgery or medicine, but scored higher for immediate actions to contain AMR. Compared with surgeons, physicians in medicine had a greater awareness of AMS activities and recognition of AMR as a significant problem, but they had lower confidence in antibiotic prescribing decisions and immediate actions to contain AMR. These observations are in line with a UK study that found that emergency physicians experienced pressure for immediate action out of fear of losing a patient and a lack of ownership of antibiotic decision-making due to the patient transitioning to inpatient care, that medical doctors adopted a more policy-informed, interdisciplinary approach, and that senior surgeons left complex antibiotic decisions to junior staff, resulting in potential defensive and inappropriate antibiotic use[16]. Variations in the social norms, values and behaviours between specialties should inform what is the best approach to antibiotic decision-making.

For peer review only

Our study had several strengths and weaknesses. First, we were able to identify a relevant set of attributes through a factor analysis optimization process, with adequate content, face and construct validity and internal reliability. In the absence of adequately validated instruments regarding AMR and AMS[37], this study adds important value to the field, with particular relevance and applicability for LMIC. Nonetheless, further questionnaire validation steps (such as criterion-related validity) are necessary to achieve a fully valid and reliable instrument. Second, the study had a large, varied respondent sample and high response rate. However, non-participation and the convenient hospital sample could have introduced selection bias, and the data are not necessarily representative for Jakarta or Indonesia at large. The authenticity of the answers was maximised by protecting the respondents' identities, although reliance on self-report has potential for social desirability bias. Third, factor analysis is based on using a "heuristic", which leaves room to more than one interpretation of the same data and cannot identify causality.

Conclusion

AMS implementation in Indonesian hospitals is likely highly dependent on institutional, contextual and diagnostic vulnerabilities. These may result in the problem of AMR being externalised, instead of recognised as a local hospital problem. Current AMS strategies may be insufficiently successful in promoting prudent antibiotic use, due to lack of systematic engagement with and feedback to prescribers, aimed at building confidence in antibiotic decision-making and ownership of the AMR problem. Appropriate recognition of the contextual and social determinants of antibiotic prescribing decision-making, including hospital factors, dynamics in medical hierarchy and experience, among others, will be critical to design context-specific AMS interventions that are adopted by healthcare professionals and successfully influence behaviours[12].

For peer review only

Acknowledgements

The authors are grateful to the management, research/medical committees and clinicians of the participating hospitals for their support to the study.

EXPLAIN study group

Erni J. Nelwan, Ralalicia Limato, Manzilina Mudia, Monik Alamanda, Helio Guterres, Enty Enty, Elfrida R Manurung, Ifael Y Mauleti, Maria Mayasari, Iman Firmansyah, May Hizrani, Roswin Djaafar, Anis Karuniawati, Prof Taralan Tambunan, Prof Amin Soebandrio, Decy Subekti, Iqbal Elyazar, Mutia Rahardjani, Fitria Wulandari, Prof Reinout van Crevel, H Rogier van Doorn, Vu Thi Lan Huong, Nga Do Ti Thuy, Sonia Lewycka, Prof Alex Broom, Raph L Hamers.

Funding

This work was funded by the Wellcome Trust, UK (106680/Z/14/Z). RL is supported by an OUCRU Prize Studentship and a Nuffield Dept of Medicine Tropical Network Fund DPhil Bursary.

Conflicts of interest

The authors declare no competing interests.

Author contributions

EJN and RLH conceived the idea for the study and are the principal investigators. RLH and RL obtained the funding. RL, EJN, HVTL and RLH designed the study protocol and developed the study instrument. MM, ERM, IYM, MM, IF, and RD collected and verified the data, overseen by RL. RL, MM, MA created and curated the database. RLH and MA performed the analysis and had full access to all study data. RL, MA, and RLH drafted the paper, with critical inputs from EJN, HVTL, HRvD and AB. All authors critically revised the manuscript and gave approval for the final version to be published.

Data Availability Statement

De-identified data are available upon reasonable request via the corresponding author, after written permission has been obtained from the lead investigators.

For peer review only

References

1 O'Neill J. Tackling Drug-Resistant Infections Globally: Final Report and Recommendations. 2016.

2 Klein EY, Van Boeckel TP, Martinez EM, *et al.* Global increase and geographic convergence in antibiotic consumption between 2000 and 2015. *Proc Natl Acad Sci U S A* 2018; **115**: E3463–70.

3 Goossens H, Ferech M, Vander Stichele R, Elseviers M. Outpatient antibiotic use in Europe and association with resistance: a cross-national database study. *Lancet* 2005; **365**: 579–87.

4 Chaw PS, Höpner J, Mikolajczyk R. The knowledge, attitude and practice of health practitioners towards antibiotic prescribing and resistance in developing countries—A systematic review. *J Clin Pharm Ther* 2018; **43**: 606–13.

5 Md Rezal RS, Hassali MA, Alrasheedy AA, Saleem F, Md Yusof FA, Godman B. Physicians' knowledge, perceptions and behaviour towards antibiotic prescribing: A systematic review of the literature. *Expert Rev Anti Infect Ther* 2015; **13**: 665–80.

6 Do NTT, Vu HTL, Nguyen CTK, *et al.* Community-based antibiotic access and use in six low-income and middle-income countries: a mixed-method approach. *Lancet Glob Heal* 2021; published online April. DOI:10.1016/S2214-109X(21)00024-3.

7 Schuts EC, Hulscher MEJL, Mouton JW, *et al.* Current evidence on hospital antimicrobial stewardship objectives: A systematic review and meta-analysis. *Lancet Infect Dis* 2016; **16**: 847–56.

8 Honda H, Ohmagari N, Tokuda Y, Mattar C, Warren DK. Antimicrobial stewardship in inpatient settings in the Asia pacific region: A systematic review and meta-analysis. *Clin Infect Dis* 2017; **64**: S119–26.

9 Laundy M, Gilchrist M, Whitney L. Antimicrobial Stewardship. Oxford, UK: Oxford University Press, 2016 DOI:10.1093/med/9780198758792.001.0001.

10 Rolfe R, Kwobah C, Muro F, *et al.* Barriers to implementing antimicrobial stewardship programs in three low- and middle-income country tertiary care settings: findings from a multi-site qualitative study. *Antimicrob Resist Infect Control* 2021; **10**: 60.

11 Teixeira Rodrigues A, Roque F, Falcão A, Figueiras A, Herdeiro MT. Understanding physician antibiotic prescribing behaviour: a systematic review of qualitative studies. *Int J Antimicrob Agents* 2013; **41**: 203–12.

12 Aveling E-L, Martin G, Armstrong N, Banerjee J, Dixon-Woods M. Quality improvement through clinical communities: eight lessons for practice. *J Health Organ Manag* 2012; **26**: 158–74.

13 Parathon H, Kuntaman K, Widiastoety TH, *et al.* Progress towards antimicrobial resistance containment and control in Indonesia. *BMJ* 2017; **358**: 31–5.

For peer review only

14 Herawati F, Ananta SC, Parwitha IAA, *et al.* Interview-based cross-sectional needs assessment to advance the implementation of an effective antibiotic stewardship program in Indonesian hospitals. *Heal Policy OPEN* 2020; **1**: 100002.

15 Limato R, Nelwan EJ, Mudia M, *et al.* A multicentre point prevalence survey of patterns and quality of antibiotic prescribing in Indonesian hospitals. *JAC-Antimicrobial Resist* 2021; **3**. DOI:10.1093/jacamr/dlab047.

16 Charani E, Ahmad R, Rawson TM, Castro-Sanchèz E, Tarrant C, Holmes AH. The Differences in Antibiotic Decision-making Between Acute Surgical and Acute Medical Teams: An Ethnographic Study of Culture and Team Dynamics. *Clin Infect Dis* 2019; **69**: 12–20.

17 Greater New York Association United Hospital Fund. Antimicrobial Stewardship Toolkit: Best practices from the GNYHA/UHF antimicrobial stewardship collaborative. 2011 https://uhfnyc.org/media/filer_public/4a/49/4a497bf9-d0a1-44c0-8849-874af1bb6019/antimicrobial_stewardship.pdf.

18 Chaw PS, Schlinkmann KM, Raupach-Rosin H, *et al.* Knowledge, attitude and practice of Gambian health practitioners towards antibiotic prescribing and microbiological testing: a cross-sectional survey. *Trans R Soc Trop Med Hyg* 2017; **111**: 117–24.

19 Asante KP, Boamah EA, Abdulai MA, *et al.* Knowledge of antibiotic resistance and antibiotic prescription practices among prescribers in the Brong Ahafo Region of Ghana; a cross-sectional study. *BMC Health Serv Res* 2017; **17**: 422.

20 Labi A-K, Obeng-Nkrumah N, Bjerrum S, *et al.* Physicians' knowledge, attitudes, and perceptions concerning antibiotic resistance: a survey in a Ghanaian tertiary care hospital. *BMC Health Serv Res* 2018; **18**: 126.

21 Venugopalan V, Trustman N, Manning N, Hashem N, Berkowitz L, Hidayat L. Administration of a survey to evaluate the attitudes of house staff physicians towards antimicrobial resistance and the antimicrobial stewardship programme at a community teaching hospital. *J Glob Antimicrob Resist* 2016; **4**: 21–7.

22 García C, Llamocca LP, García K, *et al.* Knowledge, attitudes and practice survey about antimicrobial resistance and prescribing among physicians in a hospital setting in Lima, Peru. *BMC Clin Pharmacol* 2011; **11**: 18.

23 Alumran A, Hou X-Y, Hurst C. Validity and reliability of instruments designed to measure factors influencing the overuse of antibiotics. *J Infect Public Health* 2012; **5**: 221–32.

24 Alothman A, Algwizani A, Alsulaiman M, Alalwan A, Binsalih S, Bosaeed M. Knowledge and Attitude of Physicians toward Prescribing Antibiotics and the Risk of Resistance in Two Reference Hospitals. *Infect Dis Res Treat* 2016; **9**: IDRT.S40047.

25 Chandler CI., Hutchinson E, Hutchison C. Addressing Antimicrobial Resistance

For peer review only

Through Social Theory: An Anthropologically Oriented Report. London School of Hygiene & Tropical Medicine. 2016.

26 Charoenboon N, Haenssgen MJ, Warapikuptanun P, Xayavong T, Khine Zaw Y. Translating antimicrobial resistance: a case study of context and consequences of antibiotic-related communication in three northern Thai villages. *Palgrave Commun* 2019; **5**: 23.

27 Broom A, Doron A. Antimicrobial Resistance, Politics, and Practice in India. *Qual Health Res* 2020; **30**: 1684–96.

28 Broom A, Broom J, Kirby E, Gibson A, Davis M. Antibiotic optimisation in 'the bush': Local know-how and core-periphery relations. *Health Place* 2017; **48**: 56–62.

29 Broom A, Kirby E, Gibson AF, Post JJ, Broom J. Myth, Manners, and Medical Ritual: Defensive Medicine and the Fetish of Antibiotics. *Qual Health Res* 2017; **27**: 1994–2005.

30 Will CM. Editorial: Beyond behavior? Institutions, interactions and inequalities in the response to antimicrobial resistance. Sociol. Health Illn. 2018; **40**: E1–9.

31 Chandler CIR. Current accounts of antimicrobial resistance: stabilisation, individualisation and antibiotics as infrastructure. *Palgrave Commun* 2019; **5**: 53.

32 Broom A, Broom J, Kirby E. Cultures of resistance? A Bourdieusian analysis of doctors' antibiotic prescribing. *Soc Sci Med* 2014; **110**: 81–8.

33 Broom A, Broom J, Kirby E, Adams J. The social dynamics of antibiotic use in an Australian hospital. *J Sociol* 2015; **52**: 824–39.

34 Fernandez-Lazaro CI, Brown KA, Langford BJ, Daneman N, Garber G, Schwartz KL. Late-career Physicians Prescribe Longer Courses of Antibiotics. *Clin Infect Dis* 2019; **69**: 1467–75.

35 R Hansen C, Bradley CP, Sahm LJ. Factors Influencing Successful Prescribing by Intern Doctors: A Qualitative Systematic Review. *Pharm (Basel, Switzerland)* 2016; **4**: 24.

36 Roumie CL, Halasa NB, Edwards KM, Zhu Y, Dittus RS, Griffin MR. Differences in antibiotic prescribing among physicians, residents, and nonphysician clinicians. *Am J Med* 2005; **118**: 641–8.

37 DeVon HA, Block ME, Moyle-Wright P, *et al.* A psychometric toolbox for testing validity and reliability. *J Nurs Scholarsh an Off Publ Sigma Theta Tau Int Honor Soc Nurs* 2007; **39**: 155–64.

For peer review only

Appendix: Survey questionnaire

We kindly request you to indicate your agreement with the following 40 statements using a five-point scale:
① Strongly Disagree ② Disagree ③ Neutral or Don't Know ④ Agree ⑤ Strongly Agree

**ANTIMICROBIAL RESISTANCE: SCOPE OF THE PROBLEM AND KEY CONTRIBUTORS**						
**1.**	Antimicrobial resistance is a significant problem in this hospital	①	②	③	④	⑤
**2.**	Antimicrobial resistance is a significant problem in Indonesia	①	②	③	④	⑤
**3.**	A cause of antimicrobial resistance is using too many antimicrobial drugs	①	②	③	④	⑤
**4.**	Lack of hand disinfection by healthcare workers causes spread of antimicrobial resistance	①	②	③	④	⑤
**5.**	Use of broad-spectrum antibiotics can increase antimicrobial resistance when narrower-spectrum antibiotics are available that are equally effective	①	②	③	④	⑤
**6.**	Antibiotic resistance is also a problem outside of the hospital, in communities	①	②	③	④	⑤
**7.**	In this hospital, patient rooms are cleaned according to hospital cleaning protocol once a patient with a multidrug-resistant organism (MDRO) has been discharged	①	②	③	④	⑤
**8.**	Adherence to hand-hygiene protocols is excellent at this hospital	①	②	③	④	⑤
**9.**	This hospital does NOT provide adequate staff education regarding multidrug-resistant organisms	①	②	③	④	⑤
**10.**	A patient is likely to develop an infection with a multidrug-resistant organism during their stay at this hospital	①	②	③	④	⑤
**ANTIBIOTIC PRESCRIBING PRACTICES**						
**11.**	Antibiotics are overused in Indonesia	①	②	③	④	⑤
**12.**	Antibiotics are overused in this hospital	①	②	③	④	⑤
**13.**	Microbiology laboratory results are efficiently communicated to the treating physician	①	②	③	④	⑤
**14.**	I regularly refer to/consider the antibiotic susceptibility patterns at this hospital/institution (i.e. the institutional antibiogram) when empirically prescribing antibiotics	①	②	③	④	⑤
**15.**	If medically appropriate, intravenous antibiotics should be stepped down to an oral alternative after three days	①	②	③	④	⑤
**16.**	Restrictions on antibiotics impair my ability to provide good patient care	①	②	③	④	⑤
**17.**	More judicious use of antibiotics would decrease antimicrobial resistance	①	②	③	④	⑤
**18.**	Following evidence-based antibiotic guidelines will help optimize treatment outcomes	①	②	③	④	⑤
**19.**	In general, rational antibiotic prescribing for my patients is high on my list of priorities	①	②	③	④	⑤
**20.**	Developing hospital antibiotic guidelines is more useful than applying international guidelines	①	②	③	④	⑤

For peer review only

**21.**	I am often unsure if a patient needs an antibiotic or not	①	②	③	④	⑤
**22.**	I am often unsure which antibiotic to prescribe	①	②	③	④	⑤
**23.**	I will stop antibiotics that others have prescribed in the absence of an appropriate indication	①	②	③	④	⑤
**24.**	Patients with high fever (≥39°C) must be treated with antibiotics	①	②	③	④	⑤
**25.**	If I am uncertain about the diagnosis of infection, but think it is possible, I feel safer prescribing an antibiotic	①	②	③	④	⑤
**26.**	Fear of patient deterioration or complications leads me to prescribe antibiotics more freely	①	②	③	④	⑤
**27.**	I frequently prescribe antibiotics because patients or their relatives insist on it	①	②	③	④	⑤
**ANTIMICROBIAL STEWARDSHIP PROGRAM (ASP)** *A formal program formal that monitors and manages the appropiate use of antibiotics*						
**28.**	I am aware that my hospital has an antimicrobial stewardship program (ASP)	①	②	③	④	⑤
**29.**	I understand what the purpose of ASP is	①	②	③	④	⑤
**30.**	ASP improve patient care	①	②	③	④	⑤
**31.**	ASP reduces the problem of antimicrobial resistance	①	②	③	④	⑤
**32.**	ASP reduces this hospital's infection rates	①	②	③	④	⑤
**33.**	Additional staff education on antimicrobial prescribing is needed	①	②	③	④	⑤
**34.**	Regular audit and feedback encourage me to prescribe antibiotics prudently	①	②	③	④	⑤
**35.**	Rapid and accurate diagnostic tests are useful for diagnosis of infectious diseases and guidance on antibiotic therapy	①	②	③	④	⑤
**36.**	To reduce antibiotic overuse in hospitals, implementation of antibiotic restriction (e.g., antibiotic tiers) is a useful measure	①	②	③	④	⑤
**37.**	To curb antimicrobial resistance, regular consultations or ward rounds with a clinical microbiologist or infectious disease physician are useful	①	②	③	④	⑤
**38.**	To curb antimicrobial resistance, doctors need to have timely access to microbiological test results to guide antibiotic therapy	①	②	③	④	⑤
**39.**	Up-to-date information on hospital antimicrobial resistance patterns is important for developing hospital antibiotic guidelines	①	②	③	④	⑤
**40.**	Effective infection prevention and control in the hospital reduces antimicrobial resistance	①	②	③	④	⑤

BACKGROUND INFORMATION

41. What is your primary work unit in this hospital? **Tick ONE**

- ○ Many different unit/not specific
- ○ Emergency department
- ○ Surgery
- ○ Anaesthesiology
- ○ OBGYN (Obstetrics/ Gynaecology)
- ○ Internal Medicine
- ○ Neurology
- ○ Paediatrics
- ○ Eye
- ○ Dermato-venerology
- ○ Pulmonology
- ○ Cardiology
- ○ Orthopaedics
- ○ Rehabilitation
- ○ Pharmacy
- ○ Microbiology
- ○ Laboratory
- ○ Other ____________

For peer review only

	○ Intensive Care Unit (ALL)	○ Psychiatrics<br>○ ENT (Ear Nose Throat)	○ Radiology
**42.**	How long have you worked in this hospital? **Tick ONE**		
	○ Less than 1 year<br>○ 1 – 5 years	○ 6 – 10 years<br>○ 11 – 15 years	○ 16 - 20 years<br>○ More than 20 years
**43.**	What is your position in this hospital? **Tick ONE**		
	○ Internship doctor<br>○ General Practitioner	○ Resident<br>○ Specialist	○ Other<br>___________________
**44.**	How long have you worked in your current specialty or profession? **Tick ONE**		
	○ Less than 1 year<br>○ 1 – 5 years	○ 6 – 10 years<br>○ 11 – 15 years	○ 16 - 20 years<br>○ More than 20 years
**45.**	Which of the following resources do you use to guide your antibiotic prescribing? **Tick ALL that apply**		
	○ Consultation with senior colleague(s)<br>○ Consultation with specialist in microbiology/ infectious disease<br>○ Textbooks	Guideline<br>☐ Internasional<br>☐ Nasional<br>☐ Hospital<br>☐ Departement/ division	○ Medical journal<br>○ Phamaceutical representative<br>______________________________<br>○ Internet _________________________<br>○ Other ___________________________
**46.**	During the past year, how many times have you received training/teaching or attended seminars/courses on antimicrobial prescribing, resistance and/or stewardship? _____ times		
**47.**	What is your sex: ○ Male ○ Female		
**48.**	I would like to take part in the raffle: ○ yes ○ no If yes, my email is:___________________		

Thank you for completing this survey!

For peer review only

Table 1 Characteristics of respondents

**Total**	**1007**
Sex [a]	
Female	477 (47.4)
Male	524 (52.0)
Professional hierarchy	
Intern/Internship doctor	10 (1.0)
General practitioner	113 (11.2)
Resident	500 (49.7)
Specialist/consultant	358 (35.6)
Other	18 (1.8)
Professional experience (years) [b]	
<1	194 (19.3)
1-5	459 (45.6)
6-10	136 (13.5)
11-15	74 (7.4)
16-20	52 (5.2)
>20	81 (8.0)
Grouped departments [c,f]	
Surgery (including subspecialties)	371 (36.8)
Medicine (including subspecialties)	232 (23.0)
Acute specialties	156 (15.5)
Other departments	244 (24.2)
Information sources used to guide prescribing [d]	
Guidelines	
International	619 (61.5)
National	628 (62.4)
Hospital	656 (65.1)
Department/Division	405 (40.2)
Consultation with senior colleague(s)	472 (46.9)
Consultation with microbiologist/infectious disease physician	523 (51.9)
Textbooks	410 (40.7)
Medical journals	389 (38.6)
Pharmaceutical company representative	34 (3.4)
Internet	115 (11.4)
Other	13 (1.3)
Number of AMR/AMS trainings attended in the past year [e]	
0	396 (39.3)
1	342 (34.0)
≥2	168 (16.7)
Median (range)	1 (0, 10)

Abbreviations: AMR, antimicrobial resistance; AMS, antibiotic stewardship;
Data are reported as n (%) unless indicated otherwise.
Data missing for: [a] 6 (0.60%); [b] 11 (1.1%); [c] 4 (0.40%); [d] 2 (0.20%); [e] 101 (10.0%).
[f] Surgery and surgical subspecialties includes obstetrics/gynaecology (146), surgery (122), orthopaedics (57), ENT (32), urology (14); medicine and medical subspecialties includes medicine (128), neurology (63), pulmonology (15), dermatology (14), cardiology (12); acute specialties includes anaesthesiology (72), emergency (57), ICU (27); other departments includes paediatrics (54), ophthalmology (39), multiple units (33), rehabilitation (32), psychiatry (30), dentist (27), other (29) and unspecified (4).

For peer review only

Table 2 Summary of the exploratory factor analysis of the six-factor solution (n=973)

Item #	Original item	Rotated factor loadings						Uniqueness
		Factor 1	Factor 2	Factor 3	Factor 4	Factor 5	Factor 6	
**Q01**	Antimicrobial resistance is a significant problem in this hospital	0.2159	0.04100	0.1389	0.0057	0.5701	0.0416	0.6056
**Q02**	Antimicrobial resistance is a significant problem in Indonesia	0.2433	0.10730	0.3295	0.0588	0.5742	0.0385	0.4861
**Q03**	A cause of antimicrobial resistance is using too many antimicrobial drugs	0.1641	0.20060	0.3993	0.0695	0.5361	-0.0462	0.4790
**Q04**	Lack of hand disinfection by healthcare workers causes spread of antimicrobial resistance	0.0640	0.09020	-0.2533	0.0152	0.5725	0.1179	0.5817
**Q05**	Use of broad-spectrum antibiotics can increase antimicrobial resistance when narrower-spectrum antibiotics are available that are equally effective	0.2150	0.05360	0.1482	0.0005	0.5480	0.1093	0.6167
**Q06**	Antibiotic resistance is also a problem outside of the hospital, in communities	0.2088	0.03180	0.2463	0.0632	0.4766	0.0432	0.6617
**Q07**	In this hospital, patient rooms are cleaned according to hospital cleaning protocol once a patient with a multidrug-resistant organism (MDRO) has been discharged	0.1038	0.07530	0.0868	0.0210	0.0378	0.6058	0.6071
**Q08**	Adherence to hand-hygiene protocols is excellent at this hospital	-0.0098	0.03640	0.0606	0.1099	-0.0830	0.6368	0.5705
**Q11**	Antibiotics are overused in Indonesia	0.1924	0.18310	0.5138	-0.0809	0.2489	-0.2318	0.5432
**Q12**	Antibiotics are overused in this hospital	0.0548	0.04170	0.2094	-0.4550	0.1635	-0.2381	0.6610
**Q13**	Microbiology laboratory results are efficiently communicated to the treating physician	0.1689	0.11740	-0.0915	0.0532	-0.0963	0.5116	0.6754
**Q14**	I regularly refer to/consider the antibiotic susceptibility patterns at this hospital/institution (i.e. the institutional antibiogram) when empirically prescribing antibiotics	0.0288	0.10700	-0.0292	-0.0237	0.2056	0.6115	0.5701
**Q15**	If medically appropriate, intravenous antibiotics should be stepped down to an oral alternative after three days	-0.0873	0.20850	0.1141	-0.0850	0.2311	0.3617	0.7444
**Q16**	Restrictions on antibiotics impair my ability to provide good patient care	0.0460	0.18390	0.0527	0.4031	0.0843	-0.0868	0.7842
**Q17**	More judicious use of antibiotics would decrease antimicrobial resistance	0.3010	0.07470	0.7362	0.0820	0.0892	0.0648	0.3429
**Q18**	Following evidence-based antibiotic guidelines will help optimize treatment outcomes	0.2274	0.19340	0.6565	0.1206	0.1256	0.1851	0.4153

For peer review only

Item #	Original item	Rotated factor loadings						Uniqueness
Q19	In general, rational antibiotic prescribing for my patients is high on my list of priorities	0.1845	0.18040	0.5246	0.1574	0.1184	0.3228	0.5151
Q20	Developing hospital antibiotic guidelines is more useful than applying international guidelines	0.1803	0.00750	0.3499	-0.1335	0.2631	0.0672	0.7534
Q21	I am often unsure if a patient needs an antibiotic or not	0.0489	0.02600	0.0497	0.5640	-0.3364	0.1021	0.5527
Q22	I am often unsure which antibiotic to prescribe	-0.0084	0.04570	0.1256	0.5670	-0.1938	0.0781	0.6170
Q23	I will stop antibiotics that others have prescribed in the absence of an appropriate indication	0.0016	-0.06200	0.1517	-0.1137	0.2090	0.3892	0.7650
Q24	Patients with high fever (≥39°C) must be treated with antibiotics	0.0077	0.13310	0.1695	0.4794	0.2095	-0.2351	0.6245
Q25	If I am uncertain about the diagnosis of infection, but think it is possible, I feel safer prescribing an antibiotic	0.0217	-0.04150	-0.0877	0.6741	0.1927	-0.0086	0.4985
Q26	Fear of patient deterioration or complications leads me to prescribe antibiotics more freely	0.0113	0.00390	-0.1459	0.7092	0.0883	0.0246	0.4672
Q27	I frequently prescribe antibiotics because patients or their relatives insist on it	0.1069	0.04740	0.2869	0.6318	0.0518	-0.0100	0.5021
Q28	I am aware that my hospital has an antimicrobial stewardship program (ASP)	0.2434	0.62240	0.2918	0.0866	0.0418	0.0110	0.4588
Q29	I understand what the purpose of ASP is	0.2217	0.69570	0.2635	0.1188	0.0092	0.0199	0.3828
Q30	ASP improve patient care	0.2364	0.77440	0.0873	0.0932	0.0852	0.1241	0.3055
Q31	ASP reduces the problem of antimicrobial resistance	0.2775	0.75320	0.0949	0.0172	0.0906	0.1753	0.3074
Q32	ASP reduces this hospital's infection rates	0.2045	0.66700	-0.1122	0.0569	0.1283	0.1799	0.4486
Q33	Additional staff education on antimicrobial prescribing is needed	0.5202	0.28160	0.0917	-0.0079	0.2203	-0.0284	0.5923
Q34	Regular audit and feedback encourage me to prescribe antibiotics prudently	0.6151	0.35810	0.0075	0.0064	0.1402	0.1117	0.4612
Q35	Rapid and accurate diagnostic tests are useful for diagnosis of infectious diseases and guidance on antibiotic therapy	0.6714	0.25760	0.1522	-0.0362	0.0386	0.0379	0.4555
Q36	To reduce antibiotic overuse in hospitals, implementation of antibiotic restriction (e.g. antibiotic tiers) is a useful measure	0.6428	0.26700	0.1088	-0.0335	0.2153	0.0013	0.4562
Q37	To curb antimicrobial resistance, regular consultations or ward rounds with a clinical microbiologist or infectious disease physician are useful	0.7046	0.07870	0.0061	0.0190	0.1881	0.0781	0.4555
Q38	To curb antimicrobial resistance, doctors need to have timely access to microbiological test results to guide antibiotic therapy	0.7197	0.08350	0.2786	0.0985	0.0670	0.0820	0.3765

For peer review only

Item #	Original item	Rotated factor loadings						Uniqueness
**Q39**	Up-to-date information on hospital antimicrobial resistance patterns is important for developing hospital antibiotic guidelines	0.7374	0.08540	0.3223	0.1088	0.1739	0.0348	0.3018
**Q40**	Effective infection prevention and control in the hospital reduces antimicrobial resistance	0.7067	0.16900	0.2876	0.0950	0.1798	0.0533	0.3452
	**Eigenvalues**	**4.39**	**3.19**	**2.82**	**2.78**	**2.65**	**2.17**	
	**% of variance explained**	**11.56%**	**8.40%**	**7.43%**	**7.32%**	**6.98%**	**5.72%**	**Overall 47.4%**

Table shows the results of the exploratory factor analysis (principal axis factoring) with orthogonal varimax rotation of the six-factor solution using the factor, pcf command in Stata.
Rotated factor loadings: a measure of how much each item contributes to the factor. Loadings close to -1 or 1 indicate that the factor strongly affects the item and loadings close to 0 indicate that the factor has a weak effect on the item.
Uniqueness: shows the proportion of the item's variance that is not explained by the factors.
Item #9 and 10 were excluded from the analysis as explained in Results.

For peer review only

Table 3 The latent factors of antibiotic prescribing

Factor	Factor label	Number of items	Original item #	Loadings range	Reliability (Cronbach's α)
**1**	Awareness of AMS activities	8	33-40	0.5202, 0.7374	0.8734
**2**	Awareness of AMS purposes	5	28-32	0.6224, 0.7744	0.8334
**3**	Views regarding rational antibiotic prescribing	5	11, 17-20	0.3499, 0.7362	0.6961
**4**	Confidence in antibiotic prescribing decisions	8	12, 16, 21, 22, 24-27	0.4031, 0.7092	0.6997
**5**	Perception of AMR as a significant problem	6	1-6	0.4766, 0.5742	0.6967
**6**	Immediate actions to contain AMR	6	7, 8, 13-15, 23	0.3617, 0.6368	0.5695

Abbreviations: AMR, antimicrobial resistance; AMS, antibiotic stewardship.
Item #9 and 10 were excluded from the analysis, as explained in Results.
The full table is included in Table S2.

For peer review only

Table 4 Physician subgroup analysis

	Factor 1			Factor 2			Factor 3			Factor 4			Factor 5			Factor 6		
	Coeff	95% CI	P	Coeff	95% CI	P	Coeff	95% CI	P	Coeff	95% CI	P	Coeff	95% CI	P	Coeff	95% CI	P
**Hospital**																		
**05**	Ref			Ref			Ref			Ref			Ref			Ref		
**01**	0.194	-0.089, 0.477	0.179	**-0.422**	**-0.701, -0.142**	**0.003**	0.244	-0.039, 0.527	0.091	-0.167	-0.451, 0.117	0.248	**-0.430**	**-0.716, -0.145**	**0.003**	**-0.504**	**-0.778, -0.23**	**0.000**
**02**	0.064	-0.13, 0.258	0.518	**-0.352**	**-0.543, -0.161**	**0.000**	0.134	-0.06, 0.328	0.174	-0.180	-0.375, 0.014	0.069	-0.095	-0.29, 0.101	0.342	-0.087	-0.274, 0.101	0.366
**03**	0.164	-0.116, 0.444	0.252	-0.066	-0.343, 0.211	0.641	**0.387**	**0.107, 0.668**	**0.007**	0.131	-0.151, 0.412	0.363	-0.044	-0.327, 0.238	0.758	0.092	-0.18, 0.364	0.507
**04**	0.117	-0.218, 0.453	0.494	0.116	-0.215, 0.448	0.492	0.096	-0.239, 0.432	0.573	0.249	-0.088, 0.586	0.148	-0.081	-0.419, 0.257	0.638	**0.361**	**0.035, 0.686**	**0.030**
**06**	0.196	-0.153, 0.544	0.271	0.106	-0.239, 0.45	0.548	**0.419**	**0.07, 0.768**	**0.019**	0.013	-0.338, 0.363	0.944	**-0.432**	**-0.783, -0.08**	**0.016**	**0.479**	**0.141, 0.817**	**0.005**
**Departments**																		
**Surgical**	Ref			Ref			Ref			Ref			Ref			Ref		
**Medical**	**0.239**	**0.073, 0.405**	**0.005**	0.150	-0.014, 0.314	0.073	-0.120	-0.286, 0.046	0.156	**0.307**	**0.140, 0.473**	**0.000**	**0.221**	**0.054, 0.389**	**0.009**	**-0.398**	**-0.559, -0.237**	**0.000**
**Acute**	**-0.275**	**-0.488, -0.062**	**0.011**	**-0.214**	**-0.425, -0.004**	**0.046**	**-0.273**	**-0.486, -0.06**	**0.012**	0.169	-0.045, 0.382	0.122	0.124	-0.091, 0.338	0.258	**0.242**	**0.036, 0.449**	**0.021**
**Other**	-0.081	-0.253, 0.092	0.358	-0.027	-0.197, 0.144	0.760	-0.086	-0.259, 0.086	0.326	0.046	-0.127, 0.219	0.603	0.119	-0.055, 0.292	0.181	**-0.302**	**-0.469, -0.135**	**0.000**
**Medical**	Ref			Ref			Ref			Ref			Ref			Ref		
**Acute**	**-0.514**	**-0.745, -0.283**	**0.000**	**-0.364**	**-0.593, -0.136**	**0.002**	-0.153	-0.384, 0.078	0.194	-0.138	-0.370, 0.094	0.243	-0.098	-0.330, 0.135	0.411	**0.640**	**0.417, 0.864**	**0.000**
**Other**	**-0.320**	**-0.507, -0.133**	**0.001**	-0.177	-0.361, 0.0083	0.061	0.034	-0.154, 0.221	0.724	**-0.261**	**-0.449, 0.073**	**0.006**	-0.103	-0.291, 0.086	0.285	0.096	-0.085, 0.277	0.298
**Medical Hierarchy**																		
**Consultant**	Ref			Ref			Ref			Ref			Ref			Ref		
**Intern**	**-0.744**	**-1.374, -0.115**	**0.020**	**0.637**	**0.015, 1.259**	**0.045**	0.443	-0.187, 1.074	0.168	0.067	-0.565, 0.699	0.836	0.495	-0.14, 1.129	0.126	0.003	-0.607, 0.613	0.993
**GP**	0.168	-0.096, 0.432	0.212	0.153	-0.108, 0.413	0.251	**-0.397**	**-0.661, -0.133**	**0.003**	-0.169	-0.434, 0.095	0.210	-0.011	-0.276, 0.255	0.938	-0.024	-0.279, 0.232	0.857
**Resident**	-0.091	-0.287, 0.105	0.363	**0.297**	**0.104, 0.490**	**0.003**	0.004	-0.192, 0.199	0.971	**-0.271**	**-0.468, -0.075**	**0.007**	0.058	-0.139, 0.255	0.563	**-0.202**	**-0.391, -0.012**	**0.037**
**Other**	-0.013	-0.491, 0.466	0.958	0.148	-0.325, 0.620	0.541	0.252	-0.227, 0.731	0.303	-0.172	-0.653, 0.308	0.482	-0.013	-0.495, 0.469	0.959	0.304	-0.159, 0.768	0.198
**Intern**	Ref			Ref			Ref			Ref			Ref			Ref		
**GP**	**0.912**	**0.245, 1.579**	**0.007**	-0.485	-1.144, 0.175	0.150	-0.840	-1.508, -0.172	0.014	-0.236	-0.906, 0.434	0.489	-0.505	-1.18, 0.167	0.141	-0.0263	-0.673, 0.620	0.936
**Resident**	**0.653**	**0.0458, 1.261**	**0.035**	-0.340	-0.941, -0.260	0.267	-0.440	-1.048, 0.169	0.157	-0.338	-0.948, 0.272	0.278	-0.436	-1.049, 0.176	0.162	-0.205	-0.793, 0.384	0.496
**Other**	0.732	-0.457, 1.509	0.065	-0.490	-1.258, 0.279	0.212	-0.191	-0.970, 0.587	0.630	-0.239	-1.020, 0.542	0.548	-0.507	-1.291, 0.276	0.204	0.302	-0.452, 1.055	0.433
**Resident**	Ref			Ref			Ref			Ref			Ref			Ref		
**GP**	0.259	-0.037, 0.555	0.086	-0.145	-0.437, 0.148	0.333	-0.400	-0.696, -0.104	0.008	0.102	-0.196, 0.399	0.503	-0.069	-0.367, 0.229	0.651	0.178	-0.108, 0.465	0.223
**Other**	0.0781	-0.417, 0.574	0.757	-0.150	-0.639, 0.340	0.550	0.248	-0.248, 0.744	0.326	0.099	-0.399, 0.596	0.697	-0.710	-0.570, 0.428	0.780	**0.506**	**-0.163, 1.29**	**0.039**
**Years in current profession**																		
**0-5**	Ref			Ref			Ref			Ref			Ref			Ref		
**6-10**	0.089	-0.122, 0.299	0.410	0.225	**0.017, 0.434**	**0.034**	-0.089	-0.300, 0.122	0.410	0.094	-0.118, 0.305	0.385	**0.290**	**0.077, 0.502**	**0.008**	0.053	-0.151, 0.258	0.608
**11-15**	0.087	-0.185, 0.358	0.532	-0.015	-0.283, 0.254	0.915	-0.066	-0.338, 0.207	0.637	0.271	-0.002, 0.544	0.052	0.156	-0.118, 0.43	0.265	-0.095	-0.358, 0.169	0.481
**>15**	0.117	-0.118, 0.353	0.328	0.194	-0.039, 0.427	0.102	-0.044	-0.279, 0.192	0.715	-0.057	-0.293, 0.179	0.637	**0.333**	**0.096, 0.57**	**0.006**	**0.366**	**0.138, 0.594**	**0.002**
**Public sector***	-0.064	-0.4, 0.273	0.710	0.264	-0.207, 0.736	0.272	-0.238	-0.575, 0.100	0.167	0.256	-0.835, 0.595	0.140	0.192	-0.148, 0.533	0.268	0.362	0.273, 0.996	0.264
**Tertiary level***	-0.093	-0.42, 0.234	0.577	-0.289	-0.767, 0.190	0.237	-0.047	-0.375, 0.281	0.779	-0.317	-0.647, 0.012	0.059	0.052	-0.279, 0.383	0.758	-0.407	-1.065, 0.25	0.225

Abbreviations: GP, general practitioner.
The table summarizes the results of the multivariable mixed-effect linear regression models to assess the associations between independent variables and each of the factor scores (dependent variable), while adjusting for the possible interdependence of observations clustered within hospitals as well as confounders (sex, AMS training).
* This model did not include hospital as an independent variable due to collinearity.

For peer review only

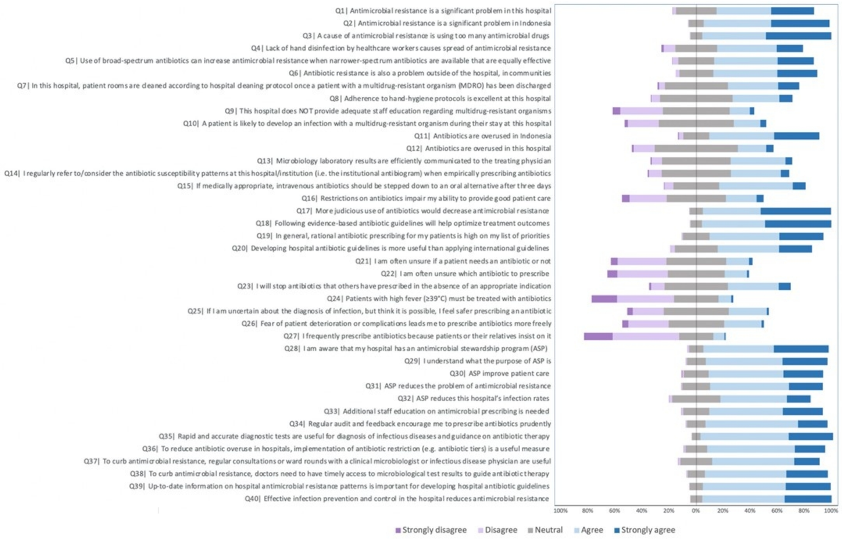

Figure 1. Five-point Likert scale responses for the 40-item questionnaire
Acronyms: ASP, antibiotic stewardship program. Data (n, %) are summarized in supplementary file Table S3

338x190mm (96 x 96 DPI)

Table S1 Survey response rate, overall and by hospital, department and professional hierarchy

	Total physicians invited	Total physicians responded	Response rate (%)	Respondents per physician type				
				Specialist/ Consultant	Resident	General practitioner	Intern/Internship doctor	Other
**Total**	**1896**	**1007**	**53.1%**	**358 (35.6%)**	**500 (49.7%)**	**113 (11.2%)**	**10 (1.0%)**	**18 (1.8%)**
**Hospital** [a]								
01	101 (5.3%)	70 (7.0%)	69.3%	39 (10.9%)	0 (0.0%)	27 (23.9%)	0 (0.0%)	4 (22.2%)
02	255 (13.4%)	155 (15.4%)	60.8%	70 (19.6%)	52 (10.4%)	26 (23.0%)	0 (0.0%)	5 (27.8%)
03	143 (7.5%)	77 (7.7%)	53.9%	41 (11.5%)	0 (0.0%)	35 (31.0%)	0 (0.0%)	1 (5.6%)
04	55 (2.9%)	44 (4.4%)	80.0%	29 (8.1%)	0 (0.0%)	11 (9.7%)	0 (0.0%)	1 (5.6%)
05	1240 (65.4%)	622 (61.8%)	50.2%	152 (42.5%)	448 (89.6%)	4 (3.5%)	10 (100.0%)	5 (27.8%)
06	102 (5.4%)	39 (3.9%)	38.2%	27 (7.5%)	0 (0.0%)	10 (8.9%)	0 (0.0%)	2 (11.1%)
**Sector** [b]								
Private	346	186 (18.5%)	53.8%	107 (29.9%)	0 (0.0%)	0 (0.0%)	72 (63.7%)	7 (38.9%)
Public	1550	821 (81.5%)	53.0%	251 (70.1%)	500 (100.0%)	41 (36.3%)	10 (100.0%)	11 (61.1%)
**Level of care** [c]								
Secondary	1499	777 (77.2%)	51.8%	136 (38.0%)	0 (0.0%)	83 (73.5%)	0 (0.0%)	8 (44.4%)
Tertiary	397	230 (22.8%)	57.9%	222 (62.0%)	500 (100.0%)	30 (26.6%)	10 (100.0%)	10 (55.6%)
**Department** [d]								
Surgery and surgical subspecialties	***628***	***371 (36.8)***	***59.1%***	***91 (24.5%)***	***226 (60.9%)***	***1 (0.27%)***	***4 (1.1%)***	***0 (0.0%)***
Obstetrics/gynaecology	229	146 (14.5%)	63.8%	51 (14.3%)	91 (18.2%)	1 (0.88%)	1 (10.0%)	0 (0.0%)
Surgery	210	122 (12.1%)	58.1%	2 (11.1%)	74 (14.8%)	0 (0.0%)	3 (30.0%)	0 (0.0%)
Orthopaedics	72	57 (5.7%)	44.4%	25 (7.0%)	32 (6.4%)	0 (0.0%)	0 (0.0%)	0 (0.0%)
Ear-nose-throat	74	32 (3.2%)	77.0%	12 (3.4%)	19 (3.8%)	0 (0.0%)	0 (0.0%)	0 (0.0%)
Urology	43	14 (1.4%)	32.6%	1 (1.1%)	10 (2.0%)	0 (0.0%)	0 (0.0%)	0 (0.0%)
Medicine and medical subspecialties	***456***	***232 (23.0%)***	***50.9%***	***115 (49.6%)***	***109 (47.0%)***	***0 (0.0%)***	***3 (1.3%)***	***5 (2.2%)***
Internal medicine	261	128 (12.7%)	49.0%	59 (16.5%)	67 (13.4%)	0 (0.0%)	0 (0.0%)	2 (11.1%)
Neurology	74	63 (6.3%)	85.1%	17 (4.8%)	40 (8.0%)	0 (0.0%)	3 (30.0%)	3 (16.7%)
Pulmonology	21	15 (1.5%)	71.4%	15 (4.2%)	0 (0.0%)	0 (0.0%)	0 (0.0%)	0 (0.0%)
Dermato-venerology	77	14 (1.4%)	18.2%	12 (0.0%)	2 (3.4%)	0 (0.0%)	0 (0.0%)	0 (0.0%)
Cardiology	23	12 (1.2%)	52.2%	12 (3.4%)	0 (0.0%)	0 (0.0%)	0 (0.0%)	0 (0.0%)
Acute specialties [e]	***96***	***156 (15.5)***	***162.5%***	***28 (17.9%)***	***67 (42.9%)***	***68 (43.6%)***	***2 (1.3%)***	***1 (0.64%)***
Anaesthesiology	96	72 (7.2%)	75.0%	4 (1.1%)	66 (13.2%)	0 (0.0%)	2 (20.0%)	0 (0.0%)

For peer review only - http://bmjopen.bmj.com/site/about/guidelines.xhtml

	Total physicians invited	Total physicians responded	Response rate (%)	Respondents per physician type				
				Specialist/ Consultant	Resident	General practitioner	Intern/Internship doctor	Other
Emergency dept [e]	-	57 (5.7%)	-	0 (0.0%)	0 (0.0%)	57 (50.4%)	0 (0.0%)	0 (0.0%)
Intensive Care Unit [e]	-	27 (2.7%)	-	14 (3.9%)	1 (0.2%)	11 (9.7%)	0 (0.0%)	1 (5.6%)
Other departments	***539***	***244 (24.2)***	***45.2%***	***90 (36.9%)***	***98 (40.2%)***	***24 (9.8%)***	***1 (0.41%)***	***10 (4.1%)***
Paediatrics	202	54 (5.4%)	26.7%	28 (7.8%)	25 (5.0%)	0 (0.0%)	0 (0.0%)	0 (0.0%)
Ophthalmology	102	39 (3.9%)	38.2%	22 (6.2%)	17 (3.4%)	0 (0.0%)	0 (0.0%)	0 (0.0%)
Multiple/unspecified units [e]	-	33 (3.3%)	-	5 (1.4%)	5 (1.0%)	21 (18.6%)	1 (10.0%)	0 (0.0%)
Rehabilitation	70	32 (3.2%)	45.7%	4 (1.1%)	28 (5.6%)	0 (0.0%)	0 (0.0%)	0 (0.0%)
Psychiatry	54	30 (3.0%)	55.6%	11 (3.1%)	19 (3.8%)	0 (0.0%)	0 (0.0%)	0 (0.0%)
Dentist	49	27 (2.7%)	55.1%	11 (3.1%)	1 (0.20%)	3 (2.7%)	0 (0.0%)	10 (55.6%)
Other	39	25 (2.5%)	64.1%	4 (1.1%)	1 (0.20%)	20 (17.7%)	0 (0.0%)	0 (0.0%)
Microbiology	4	3 (0.30%)	75.0%	2 (0.56%)	1 (0.20%)	0 (0.0%)	0 (0.0%)	0 (0.0%)
Laboratory	19	1 (0.10%)	5.3%	1 (0.28%)	0 (0.0%)	0 (0.0%)	0 (0.0%)	0 (0.0%)
Missing	-	4 (0.40%)	-	2 (0.56%)	1 (0.20%)	0 (0.0%)	0 (0.0%)	0 (0.0%)

Data are reported as n (%).
Doctor type was missing for 8 respondents, across [a] hospitals (02 (2), 03 (3), and 05 (3)); [b] sector (public 8, private 0); [c] level of care (secondary 3, tertiary 5); and [d] departments (obstetrics/gynaecology 2; ENT 1; paediatrics 1; multiple units 1; dental/oral surgery 2; missing dept 1).
[e] Invitations to participate in the survey were sent out according to primary work units; the discrepancies reported for the acute and other departments result from the fact that n=177 respondents who worked across multiple units (mostly GPs and internship doctors) indicated a different primary department on their questionnaire.

For peer review only

Table S2 The latent factors of antibiotic prescribing (full table)

Factor	Factor label	Survey questions included in the factor loading	Loadings	Reliability (Cronbach's α)
**1**	Awareness of AMS activities	Q33: Additional staff education on antimicrobial prescribing is needed	0.5202	0.8734
		Q34: Regular audit and feedback encourage me to prescribe antibiotics prudently	0.6151	
		Q35: Rapid and accurate diagnostic tests are useful for diagnosis of infectious diseases and guidance on antibiotic therapy	0.6714	
		Q36: To reduce antibiotic overuse in hospitals, implementation of antibiotic restriction (e.g. antibiotic tiers) is a useful measure	0.6428	
		Q37: To curb antimicrobial resistance, regular consultations or ward rounds with a clinical microbiologist or infectious disease physician are useful	0.7046	
		Q38: To curb antimicrobial resistance, doctors need to have timely access to microbiological test results to guide antibiotic therapy	0.7197	
		Q39: Up-to-date information on hospital antimicrobial resistance patterns is important for developing hospital antibiotic guidelines	0.7374	
		Q40: Effective infection prevention and control in the hospital reduces antimicrobial resistance	0.7067	
**2**	Awareness of AMS purposes	Q28: I am aware that my hospital has an antimicrobial stewardship program (ASP)	0.6224	0.8334
		Q29: I understand what the purpose of ASP is	0.6957	
		Q30: ASP improve patient care	0.7744	
		Q31: ASP reduces the problem of antimicrobial resistance	0.7532	
		Q32: ASP reduces this hospital's infection rates	0.6670	
**3**	Views regarding rational antibiotic prescribing	Q11: Antibiotics are overused in Indonesia	0.5138	0.6961
		Q17: More judicious use of antibiotics would decrease antimicrobial resistance	0.7362	
		Q18: Following evidence-based antibiotic guidelines will help optimize treatment outcomes	0.6565	
		Q19: In general, rational antibiotic prescribing for my patients is high on my list of priorities	0.5246	
		Q20: Developing hospital antibiotic guidelines is more useful than applying international guidelines	0.3499	
**4**	Confidence in antibiotic prescribing decisions	Q12: Antibiotics are overused in this hospital	-0.4550	0.6997
		Q16: Restrictions on antibiotics impair my ability to provide good patient care	0.4031	
		Q21: I am often unsure if a patient needs an antibiotic or not	0.5640	
		Q22: I am often unsure which antibiotic to prescribe	0.5670	
		Q24: Patients with high fever (≥39°C) must be treated with antibiotics	0.4794	

For peer review only - http://bmjopen.bmj.com/site/about/guidelines.xhtml

Factor	Factor label	Survey questions included in the factor loading	Loadings	Reliability (Cronbach's α)
		Q25: If I am uncertain about the diagnosis of infection, but think it is possible, I feel safer prescribing an antibiotic	0.6741	
		Q26: Fear of patient deterioration or complications leads me to prescribe antibiotics more freely	0.7092	
		Q27: I frequently prescribe antibiotics because patients or their relatives insist on it	0.6318	
**5**	Perception of AMR as a significant problem	Q1: Antimicrobial resistance is a significant problem in this hospital	0.5701	0.6967
		Q2: Antimicrobial resistance is a significant problem in Indonesia	0.5742	
		Q3: A cause of antimicrobial resistance is using too many antimicrobial drugs	0.5361	
		Q4: Lack of hand disinfection by healthcare workers causes spread of antimicrobial resistance	0.5725	
		Q5: Use of broad-spectrum antibiotics can increase antimicrobial resistance when narrower-spectrum antibiotics are available that are equally effective	0.5480	
		Q6: Antibiotic resistance is also a problem outside of the hospital, in communities	0.4766	
**6**	Immediate actions to contain AMR	Q7: In this hospital, patient rooms are cleaned according to hospital cleaning protocol once a patient with a multidrug-resistant organism (MDRO) has been discharged	0.6058	0.5695
		Q8: Adherence to hand-hygiene protocols is excellent at this hospital	0.6368	
		Q13: Microbiology laboratory results are efficiently communicated to the treating physician	0.5116	
		Q14: I regularly refer to/consider the antibiotic susceptibility patterns at this hospital/institution (i.e. the institutional antibiogram) when empirically prescribing antibiotics	0.6115	
		Q15: If medically appropriate, intravenous antibiotics should be stepped down to an oral alternative after three days	0.3617	
		Q23: I will stop antibiotics that others have prescribed in the absence of an appropriate indication	0.3892	

Abbreviations: AMS, antibiotic stewardship; ASP, antibiotic stewardship programme; IPC, infection prevention and control
Data are summarised in Table 3
Item #9 and 10 were excluded from the analysis, as explained in Results.

For peer review only - http://bmjopen.bmj.com/site/about/guidelines.xhtml

Table S3. Five-point Likert scale responses for the 40-item questionnaire

Item #	No of respondents	Strongly disagree (1)	Disagree (2)	Neutral (3)	Agree (4)	Strongly agree (5)
Q1	1005	3 (0.30)	26 (2.6)	168 (16.7)	452 (45.0)	356 (35.4)
Q2	1006	0 (0.0)	7 (0.70)	55 (5.5)	505 (50.2)	439 (43.6)
Q3	1003	0 (0.0)	6 (0.60)	43 (4.3)	472 (47.1)	482 (48.1)
Q4	1005	19 (1.9)	98 (9.8)	173 (17.2)	497 (49.5)	218 (21.7)
Q5	1004	2 (0.20)	49 (4.9)	145 (14.4)	514 (51.2)	294 (29.3)
Q6	1003	0 (0.0)	29 (2.9)	136 (13.6)	516 (51.5)	322 (32.1)
Q7	1004	12 (1.2)	53 (5.3)	288 (28.7)	460 (45.8)	191 (19.0)
Q8	1002	6 (0.60)	81 (8.1)	345 (34.4)	444 (44.3)	126 (12.6)
Q9	1005	71 (7.1)	397 (39.5)	311 (31.0)	188 (18.7)	38 (3.8)
Q10	1005	32 (3.2)	299 (29.8)	363 (36.1)	256 (25.5)	55 (5.5)
Q11	1005	8 (0.80)	34 (3.4)	102 (10.2)	506 (50.4)	355 (35.3)
Q12	1005	18 (1.8)	213 (21.2)	448 (44.5)	286 (28.5)	71 (7.1)
Q13	1005	11 (1.1)	97 (9.7)	321 (31.9)	515 (51.2)	61 (6.1)
Q14	1005	12 (1.2)	121 (12.0)	324 (32.2)	468 (46.6)	80 (8.0)
Q15	1006	7 (0.70)	73 (7.3)	195 (19.4)	623 (61.9)	108 (10.7)
Q16	1006	67 (6.7)	334 (33.2)	267 (26.5)	276 (27.4)	62 (6.2)
Q17	1005	2 (0.20)	2 (0.20)	48 (4.8)	431 (42.9)	522 (51.9)
Q18	1006	0 (0.0)	6 (0.60)	42 (4.2)	467 (46.4)	491 (48.8)
Q19	1005	1 (0.10)	7 (0.70)	105 (10.5)	545 (54.3)	347 (34.5)
Q20	1004	3 (0.30)	34 (3.4)	180 (17.9)	511 (50.9)	276 (27.5)
Q21	1006	60 (6.0)	440 (43.7)	269 (26.7)	204 (20.3)	33 (3.3)
Q22	1006	89 (8.9)	448 (44.5)	254 (25.3)	196 (19.5)	19 (1.9)
Q23	1002	19 (1.9)	124 (12.4)	288 (28.7)	464 (46.3)	107 (10.7)
Q24	1005	213 (21.2)	480 (47.8)	189 (18.8)	104 (10.4)	19 (1.9)
Q25	1005	51 (5.1)	285 (28.4)	301 (30.0)	348 (34.6)	20 (2.0)
Q26	1006	55 (5.5)	354 (35.2)	245 (24.4)	330 (32.8)	22 (2.2)
Q27	1005	231 (22.3)	538 (53.5)	138 (13.7)	90 (9.0)	8 (0.80)
Q28	1006	0 (0.0)	15 (1.5)	54 (5.4)	526 (52.3)	411 (40.9)
Q29	1006	2 (0.20)	6 (0.60)	71 (7.1)	585 (58.2)	342 (34.0)
Q30	1006	7 (0.70)	11 (1.1)	97 (9.6)	582 (57.9)	309 (30.7)
Q31	1005	2 (0.20)	7 (0.70)	109 (10.89)	620 (61.7)	267 (26.6)

For peer review only - http://bmjopen.bmj.com/site/about/guidelines.xhtml

Item #	No of respondents	Strongly disagree (1)	Disagree (2)	Neutral (3)	Agree (4)	Strongly agree (5)
Q32	1004	3 (0.30)	25 (2.5)	206 (20.5)	568 (56.6)	202 (20.1)
Q33	1005	2 (0.20)	14 (1.4)	101 (10.1)	574 (57.1)	314 (31.2)
Q34	1006	1 (0.10)	7 (0.70)	70 (7.0)	702 (69.8)	226 (22.5)
Q35	1006	0 (0.0)	4 (0.40)	31 (3.1)	646 (64.2)	325 (32.3)
Q36	1007	1 (0.10)	13 (1.3)	83 (8.2)	675 (67.0)	235 (23.3)
Q37	1006	2 (0.20)	17 (1.7)	128 (12.7)	657 (65.3)	202 (20.1)
Q38	1006	0 (0.0)	10 (0.99)	66 (6.6)	616 (61.2)	314 (31.2)
Q39	1006	0 (0.0)	4 (0.40)	49 (4.9)	617 (61.3)	336 (33.4)
Q40	1006	0 (0.0)	4 (0.40)	43 (4.3)	612 (60.8)	347 (34.5)

Data are also shown in Figure 1.

For peer review only

Figure S1 Scree plot showing eigenvalues for the 40 factors

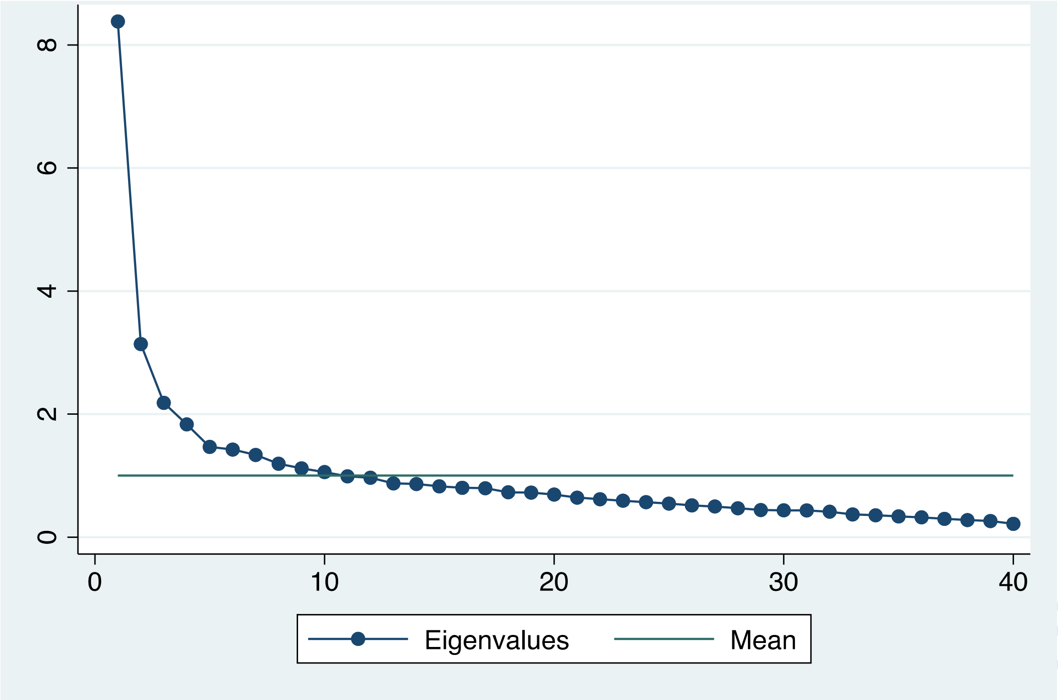

For peer review only - http://bmjopen.bmj.com/site/about/guidelines.xhtml

Figure S2 Parallel analysis showing adjusted eigenvalues for the 40 factors.

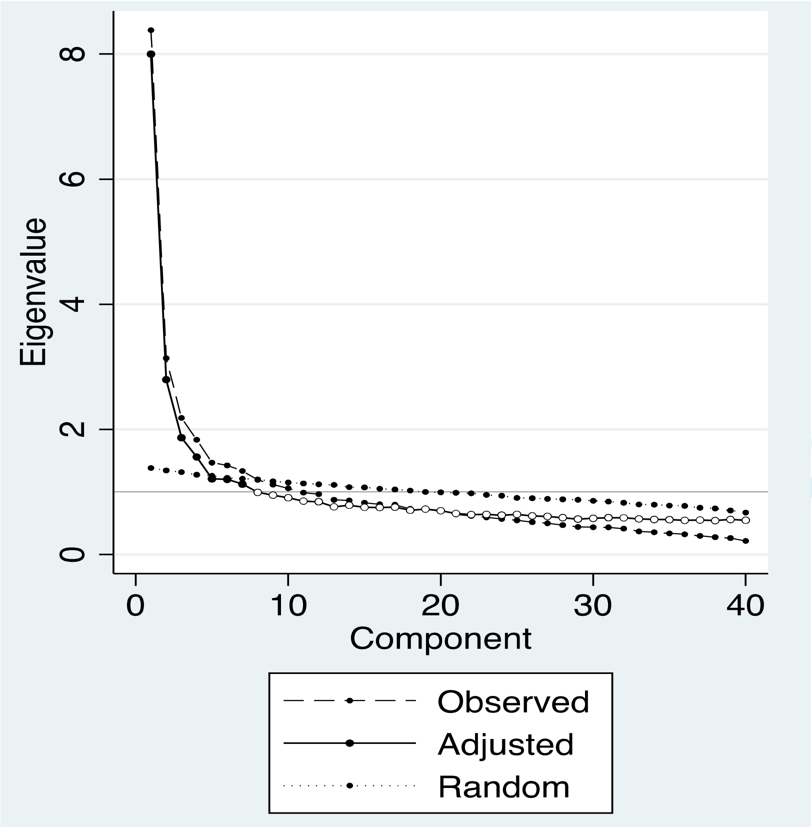

Parallel analysis adjusted the original eigenvalues for sampling error-induced collinearity among the variables to arrive at the adjusted eigenvalues.

For peer review only - http://bmjopen.bmj.com/site/about/guidelines.xhtml

STROBE Statement—
Checklist of items that should be included in reports of ***cross-sectional studies***

	Item No	Recommendation	Page No
**Title and abstract**	1	(*a*) Indicate the study's design with a commonly used term in the title or the abstract	1
		(*b*) Provide in the abstract an informative and balanced summary of what was done and what was found	2
**Introduction**			
Background/rationale	2	Explain the scientific background and rationale for the investigation being reported	4
Objectives	3	State specific objectives, including any prespecified hypotheses	4
**Methods**			
Study design	4	Present key elements of study design early in the paper	5
Setting	5	Describe the setting, locations, and relevant dates, including periods of recruitment, exposure, follow-up, and data collection	5
Participants	6	(*a*) Give the eligibility criteria, and the sources and methods of selection of participants	5
Variables	7	Clearly define all outcomes, exposures, predictors, potential confounders, and effect modifiers. Give diagnostic criteria, if applicable	6
Data sources/ measurement	8*	For each variable of interest, give sources of data and details of methods of assessment (measurement). Describe comparability of assessment methods if there is more than one group	6
Bias	9	Describe any efforts to address potential sources of bias	6
Study size	10	Explain how the study size was arrived at	7
Quantitative variables	11	Explain how quantitative variables were handled in the analyses. If applicable, describe which groupings were chosen and why	7
Statistical methods	12	(*a*) Describe all statistical methods, including those used to control for confounding	7
		(*b*) Describe any methods used to examine subgroups and interactions	7
		(*c*) Explain how missing data were addressed	7
		(*d*) If applicable, describe analytical methods taking account of sampling strategy	7
		(*e*) Describe any sensitivity analyses	NA
**Results**			
Participants	13*	(a) Report numbers of individuals at each stage of study—eg numbers potentially eligible, examined for eligibility, confirmed eligible, included in the study, completing follow-up, and analysed	8
		(b) Give reasons for non-participation at each stage	8
		(c) Consider use of a flow diagram	NA

For peer review only

Descriptive data	14*	(a) Give characteristics of study participants (eg demographic, clinical, social) and information on exposures and potential confounders	8
		(b) Indicate number of participants with missing data for each variable of interest	8-10
Outcome data	15*	Report numbers of outcome events or summary measures	8-11
Main results	16	(*a*) Give unadjusted estimates and, if applicable, confounder-adjusted estimates and their precision (eg, 95% confidence interval). Make clear which confounders were adjusted for and why they were included	8-11
		(*b*) Report category boundaries when continuous variables were categorized	8-11
		(*c*) If relevant, consider translating estimates of relative risk into absolute risk for a meaningful time period	NA
Other analyses	17	Report other analyses done—eg analyses of subgroups and interactions, and sensitivity analyses	10
**Discussion**			
Key results	18	Summarise key results with reference to study objectives	11-12
Limitations	19	Discuss limitations of the study, taking into account sources of potential bias or imprecision. Discuss both direction and magnitude of any potential bias	12
Interpretation	20	Give a cautious overall interpretation of results considering objectives, limitations, multiplicity of analyses, results from similar studies, and other relevant evidence	13
Generalisability	21	Discuss the generalisability (external validity) of the study results	12
**Other information**			
Funding	22	Give the source of funding and the role of the funders for the present study and, if applicable, for the original study on which the present article is based	14

*Give information separately for exposed and unexposed groups.

Note: An Explanation and Elaboration article discusses each checklist item and gives methodological background and published examples of transparent reporting. The STROBE checklist is best used in conjunction with this article (freely available on the Web sites of PLoS Medicine at http://www.plosmedicine.org/, Annals of Internal Medicine at http://www.annals.org/, and Epidemiology at http://www.epidem.com/). Information on the STROBE Initiative is available at www.strobe-statement.org.

For peer review only

BMJ Open

Perceptions, views and practices regarding antibiotic prescribing and stewardship among hospital physicians in Jakarta, Indonesia: a questionnaire-based survey

Journal:	*BMJ Open*
Manuscript ID	bmjopen-2021-054768.R2
Article Type:	Original research
Date Submitted by the Author:	03-Feb-2022

Complete List of Authors:	Limato, Ralalicia; Oxford University, Eijkman-Oxford Clinical Research Unit; Oxford University, Nuffield Department of Medicine, Centre for Tropical Medicine and Global Health<br>Nelwan, Erni Juwita ; RSCM, Department of Internal Medicine, Division of Infectious Diseases; Universitas Indonesia, Faculty of Medicine<br>Mudia, Manzilina; Oxford University, Eijkman-Oxford Clinical Research Unit<br>Alamanda, Monik; Oxford University, Eijkman-Oxford Clinical Research Unit<br>Manurung, Elfrida Rinawaty; Royal Taruma Hospital<br>Mauleti, Ifael Yerosias; Fatmawati General Hospital<br>Mayasari, Maria; St. Carolus Hospital<br>Firmansyah, Iman; Prof. Dr. Sulianti Saroso Infectious Disease Hospital<br>Djaafar , Roswin ; Metropolitan Medical Centre Hospital<br>Vu, Huong; Oxford University Clinical Research Unit, Hanoi Unit<br>van Doorn, H. Rogier; Oxford University Clinical Research Unit; Oxford University, Nuffield Department of Medicine, Centre for Tropical Medicine and Global Health<br>Broom, Alex; The University of Sydney, Sydney Centre for Healthy Societies, School of Social and Political Sciences<br>Hamers, Raph Leonardus; Oxford University, Eijkman-Oxford Clinical Research Unit; Oxford University, Nuffield Department of Medicine, Centre for Tropical Medicine and Global Health

**Primary Subject Heading**:	Public health
Secondary Subject Heading:	Sociology, Public health, Global health
Keywords:	Public health < INFECTIOUS DISEASES, PUBLIC HEALTH, SOCIAL MEDICINE

SCHOLARONE™ Manuscripts

I, the Submitting Author has the right to grant and does grant on behalf of all authors of the Work (as defined in the below author licence), an exclusive licence and/or a non-exclusive licence for contributions from authors who are: i) UK Crown employees; ii) where BMJ has agreed a CC-BY licence shall apply, and/or iii) in accordance with the terms applicable for US Federal Government officers or employees acting as part of their official duties; on a worldwide, perpetual, irrevocable, royalty-free basis to BMJ Publishing Group Ltd ("BMJ") its licensees and where the relevant Journal is co-owned by BMJ to the co-owners of the Journal, to publish the Work in this journal and any other BMJ products and to exploit all rights, as set out in our licence.

The Submitting Author accepts and understands that any supply made under these terms is made by BMJ to the Submitting Author unless you are acting as an employee on behalf of your employer or a postgraduate student of an affiliated institution which is paying any applicable article publishing charge ("APC") for Open Access articles. Where the Submitting Author wishes to make the Work available on an Open Access basis (and intends to pay the relevant APC), the terms of reuse of such Open Access shall be governed by a Creative Commons licence – details of these licences and which Creative Commons licence will apply to this Work are set out in our licence referred to above.

Other than as permitted in any relevant BMJ Author's Self Archiving Policies, I confirm this Work has not been accepted for publication elsewhere, is not being considered for publication elsewhere and does not duplicate material already published. I confirm all authors consent to publication of this Work and authorise the granting of this licence.

For peer review only

Perceptions, views and practices regarding antibiotic prescribing and stewardship among hospital physicians in Jakarta, Indonesia: a questionnaire-based survey

Authors

Ralalicia Limato[1,2], Erni J Nelwan[3,4,5,6], Manzilina Mudia[1], Monik Alamanda[1], Elfrida R Manurung[7], Ifael Y Mauleti[8], Maria Mayasari[9], Iman Firmansyah[10], Roswin Djaafar[6], Huong Vu Thi Lan[11], H Rogier van Doorn[2,11], Alex Broom[12], Raph L Hamers[1,2,4*]

Affiliations

1. Eijkman-Oxford Clinical Research Unit, Jakarta, Indonesia
2. Centre for Tropical Medicine and Global Health, Nuffield Department of Medicine, University of Oxford, Oxford, UK
3. Department of Internal Medicine, Division of Infectious Diseases, Cipto Mangunkusumo National General Hospital, Jakarta, Indonesia
4. Faculty of Medicine, Universitas Indonesia, Jakarta, Indonesia
5. Infectious Disease and Immunology Research Cluster, Indonesian Medical Education and Research Institute, Jakarta, Indonesia
6. Metropolitan Medical Centre Hospital, Jakarta, Indonesia
7. Royal Taruma Hospital, Jakarta, Indonesia
8. Fatmawati General Hospital, Jakarta, Indonesia
9. St. Carolus Hospital, Jakarta, Indonesia
10. Prof. Dr. Sulianti Saroso Infectious Disease Hospital, Jakarta, Indonesia
11. Oxford University Clinical Research Unit, Hanoi, Vietnam
12. Sydney Centre for Healthy Societies, School of Social and Political Sciences, The University of Sydney, Sydney, Australia

***Corresponding author**

Raph L. Hamers MD PhD

Email : raph.hamers@ndm.ox.ac.uk

Phone : +62 21 31900971

Fax : -

Inserts: Tables 4; Figures 1

Appendix: survey questionnaire

Data supplement: Tables 3; Figures 2

For peer review only - http://bmjopen.bmj.com/site/about/guidelines.xhtml

Abstract

Objectives: Antibiotic overuse is one of the main drivers of antimicrobial resistance (AMR), especially in low and middle-income countries. This study aimed to understand the perceptions and views towards antimicrobial resistance (AMR), antibiotic prescribing practice, and antimicrobial stewardship (AMS) among hospital physicians in Jakarta, Indonesia.

Design: Cross-sectional, self-administered questionnaire-based survey, with descriptive statistics, exploratory factor analysis (EFA) to identify distinct underlying constructs in the dataset, and multivariable linear regression of factor scores to analyse physician subgroups.

Setting: Six public and private general hospitals in Jakarta in 2019.

Participants: 1007 of 1896 (53.1% response rate) antibiotic prescribing physicians.

Results: Physicians acknowledged the significance of AMR and contributing factors, rational antibiotic prescribing, and purpose and usefulness of AMS. However, this conflicted with reported suboptimal local hospital practices, such as room cleaning, hand hygiene and staff education, and views regarding antibiotic decision-making. These included insufficiently applying AMS principles and utilising microbiology, lack of confidence in prescribing decisions, and defensive prescribing due to pervasive diagnostic uncertainty, fear of patient deterioration or because patients insisted. EFA identified six latent factors (overall Crohnbach's $\alpha=0.85$): awareness of AMS activities; awareness of AMS purpose; views regarding rational antibiotic prescribing; confidence in antibiotic prescribing decisions; perception of AMR as a significant problem; and immediate actions to contain AMR. Factor scores differed across hospitals, departments, work experience and medical hierarchy.

Conclusions: AMS implementation in Indonesian hospitals is challenged by institutional, contextual and diagnostic vulnerabilities, resulting in externalising AMR instead of recognising it as a local problem. Appropriate recognition of the contextual determinants of antibiotic prescribing decision-making will be critical to change physicians' attitudes and develop context-specific AMS interventions.

Keywords: perceptions; survey; antibiotic resistance; antibiotic prescribing; antibiotic stewardship; Indonesia

For peer review only - http://bmjopen.bmj.com/site/about/guidelines.xhtml

Strengths and limitations of this study

- The self-developed questionnaire in this study identified a relevant set of attributes through a factor analysis optimization process, with adequate content, face and construct validity and internal reliability. This study adds important value in the absence of adequately validated instruments regarding antimicrobial resistance and stewardship, with particular applicability for LMIC.
- This study had a large, varied respondent sample and high response rate among physicians at six public and private hospitals in Jakarta, Indonesia, and identified differences between physicians across hospitals, departments, work experience and medical hierarchy, which can guide priority-setting and tailoring of stewardship interventions.
- However, non-participation and the convenient hospital sample could have introduced selection bias, and the data are not necessarily representative for Jakarta or Indonesia.
- Factor analysis is based on using a "heuristic", which leaves room to more than one interpretation of the same data and cannot identify causality.

For peer review only

Introduction

The global rise in drug-resistant infections is one of the leading threats to public health globally, with increasing rates of morbidity, mortality and escalating healthcare costs[1]. Misuse and overuse of antimicrobial drugs in human health care is one of the main drivers[2,3] and also represents a key solution, i.e. judicious use of remaining antibiotics. Globally, use of antibiotics remains largely unrestrained and poorly governed, with large, unregulated healthcare systems representing an increasingly challenging area for achieving the goal of optimization. Substantial variations in contributing factors to inappropriate antibiotic prescribing exist across contexts, e.g. diagnostic uncertainty, pressure from pharmaceutical industry or patients[4,5], with the structure and funding of health systems inflecting enactment of optimization strategies, including antimicrobial stewardship (AMS)[6].

Patients infected with drug-resistant bacteria causing common bacterial infections in hospitalised populations are more likely to receive inappropriate antibiotic therapy resulting in higher mortality rates and opportunities for spreading to other patients[7,8]. Hospital AMS programs aim to control antimicrobial use, and have been associated with reducing hospital-acquired infections, unnecessary healthcare costs, and potentially drug-resistant infections[9–11]. However, AMS programmes in turn may jar with local constraints and practices and have been shown to have limited traction when attempts to implement occur without adequate understanding of context[12].

The global push to enact effective AMS requires detailed, context-specific data on physicians, given their central role in the complex process of antibiotic prescribing in hospitals, which can inform on how AMR is conceived, how current prescribing is rationalised, and how broad AMS principles may be experienced across contexts and nations[13,14]. Few studies to date have been conducted on this topic in low and middle-income countries (LMIC), with insufficient evaluation of the psychometric properties of their measurement instruments to examine their suitability to the specific context[4,5].

Indonesia, a diverse middle-income country in Southeast Asia with the world's fourth largest population (275 million), is particularly vulnerable for AMR, ranked among the greatest risers (29th of 76 countries) in antibiotic consumption, estimated at 2.5-fold between 2000 and 2015[2]. The implementation of the National Action Plan for AMR (NAP) has been hindered due to, among other factors, a limited evidence base of AMR epidemiology, antibiotic utilization and rational prescribing practices[15]. Weakly enforced antibiotic policies promote inappropriate prescribing and over-the-counter access without a prescription, and AMS is generally in an early stage of implementation[15,16]. Although nationwide representative AMR

For peer review only - http://bmjopen.bmj.com/site/about/guidelines.xhtml

data are not available, anecdotal evidence suggested high rates of AMR among common Gram-negative bacteria.

To identify context-specific opportunities for AMS interventions, we conducted a questionnaire-based survey among antibiotic-prescribing physicians in hospitals in Jakarta, Indonesia, to evaluate their perceptions of AMR, antibiotic prescribing practice, and views on AMS, and explore differences between physician subgroups. The study also aimed to evaluate the construct validity and psychometric properties of the questionnaire, and explore differences between physician subgroups.

Methods

Study design and setting

We conducted a cross-sectional survey between March and August 2019 among all antimicrobial prescribing physicians at six general hospitals in Jakarta, Indonesia, as part of a mixed-method study to identify targets for quality improvement in antibiotic prescribing practices (EXPLAIN study[17]). The participant hospitals were purposively sampled to achieve a representation of the diversity of hospital types in Jakarta, in terms of geographic location, size (small to large), health sector (public and private) and health care level (tertiary and secondary). In 2018, a total of 189 hospitals were operational in Jakarta province, of which 59 were public and 130 private, with a total of 15,444 and 15,425 inpatient beds, respectively[18]. The hospital sample was then pragmatically composed based on existing collaborations, site willingness, and available study resources, and included two tertiary-care government hospitals and four secondary hospitals, three of which were private hospitals, with between 134 and 853 inpatient beds, situated across all five districts of Jakarta. At the time of the survey, all six hospitals had an AMS programme, albeit at various stages of AMS implementation, initiated between 2009 and 2018.

Study respondents

All qualified physicians prescribing antibiotics on a regular basis working were eligible for the survey and were approached to participate. The survey intended to include physicians across all clinical departments, work experience and professional hierarchy, to ensure representation of different antibiotic practices and views, perceptions, and experiences related to AMR and AMS[19]. We included interns/internship doctors *(magang/dokter internsip;* recent graduates working under supervision of a specialist), general practitioners (GPs) (*dokter umum*; physicians who practice general medicine treating common, non-specialized medical conditions), residents (*residen*; physicians who are in specialist training),

For peer review only

For peer review only - http://bmjopen.bmj.com/site/about/guidelines.xhtml

specialist/consultant physicians (*dokter spesialis/konsultan*; specialized in a particular area of medicine), and other physicians.

Patient and public involvement statement

Patients or the public were not involved in the design, conduct, or reporting of the research.

Survey questionnaire

We developed a two-page anonymous, self-administered, paper-based questionnaire, which was easy to complete and based on a conceptual framework that included attributes related to prescribers' perceptions, views and practices. Good practice recommendations for questionnaire design and existing questionnaires in the literature were reviewed and discussed with several experts. The Clinician Pre/Post Perception Survey of the Greater New York Hospital Association United Hospital Fund[20] constituted the initial set of items, supplemented with relevant items from other existing questionnaires[21–27]. From a preliminary pool, we selected 69 items, of which 8 items were worded in the negative to address the acquiescence effect. The instrument was translated from English to Indonesian, and back-translated by an independent translator. The questionnaire was pre-tested by a convenience panel of 18 physicians (2 GPs, 15 residents, and 1 consultant). According to their feedback, we made adjustments to clarify ambiguous items, and remove redundant items. The final version consisted of 40 items and took about 10 minutes to complete.

The final questionnaire included an explanation of study purpose and completion instructions; 40 short statements (items) to which participants were asked to indicate the extent to which each reflected their own opinion on a 5-point Likert scale, divided into 3 sections: scope of the AMR problem and key contributors; antibiotic prescribing practices; AMS; and respondent socio-demographics (**Appendix**).

Respondent recruitment

The hospital management provided the total number of prescribing physicians for each department. The questionnaires were delivered to the head of each unit who then distributed the survey to all eligible staff. The study coordinator kept a record of numbers of physicians approached and participated. Upon survey completion, respondents could enter a raffle to win one of three gift cards in each hospital (US$14 each); there were no other incentives for participation.

Ethical approval

The research ethics committee of the Faculty of Medicine University of Indonesia (1364/UN2.F1/ETIK/2018) and the Oxford Tropical Research Ethics Committee (559-18)

For peer review only - http://bmjopen.bmj.com/site/about/guidelines.xhtml

approved the study, with additional permission from hospital management. As the survey was anonymous, consent was inferred when the participant completed and returned the questionnaire.

Statistical analysis

The percentage of respondents selecting each answer choice was calculated using the total number of responses as the denominator. For an EFA, a common lower bound for sample size is 10 cases per variable, suggesting a minimum sample size of 400; to allow for meaningful subgroup comparisons and minimize selection bias, we targeted a >50% response rate and a sample size of >1000 across the six hospitals.

We performed EFA to identify underlying distinct constructs, using factor, pcf command in Stata with orthogonal (varimax) rotation. For this analysis, the eight items worded in the negative were reverse-coded, and missing data for categorical variables were treated as a separate category. The Kaiser-Meyer-Olkin (KMO) was calculated to ensure EFA requirements were met. Each item was assigned to a certain factor based on the highest absolute factor loading of the rotated solution. To determine the optimal factor solution, we used the Kaiser criterion, scree plot of eigenvalues, and Horn's parallel analysis (*paran* package in Stata). Parallel analysis adjusts the original eigenvalues for sampling error induced collinearity among the variables to arrive at adjusted eigenvalues. Analogous to the Kaiser criterion, only factors with adjusted eigenvalues >1 were retained. The scree plot of eigenvalues and the scree plot of parallel analysis were visually inspected and factors were retained above the inflection. To interpret the factor solutions, we examined the factor loadings, and assigned each item to a certain factor based on the highest absolute factor loading (minimum loading 0.30), and then produced an umbrella label for each factor that best characterized the joint meaning of all the variables associated with it. If a certain item did not fit with the factor structure (e.g., the results were not interpretable or a factor comprised less than 3 items), the analysis was re-run without the pertinent item(s). After the optimal factor solution had been achieved, we used the regression scoring method to compute the factor scores based on the factor loadings, and Cronbach's α coefficient to test internal reliability. Using the factor scores as the dependent variable, we developed six (one for each factor score) multivariable linear regression models to assess associations with key physician characteristics ("subgroups") as the following five independent variables, i.e., hospital sector and care level, grouped departments, work experience and medical hierarchy. Given the hierarchical structure of the data (respondents clustered within hospitals) we applied multilevel (two-level) modelling to control for possible clustering within hospitals. We also adjusted for possible confounders. P-values<0.05 were considered statistically significant. All analyses were done with Stata/IC Version 16.1 (StataCorp, College Station, TX, US).

For peer review only - http://bmjopen.bmj.com/site/about/guidelines.xhtml

Results

Respondent characteristics

All 1896 antibiotic prescribing physicians at the six hospitals were approached, and 1007 (53.1%) participated in the survey. **Table 1** summarizes the participants' key characteristics. **Table S1 in supplementary file** summarizes the response rates.

Description of respondent responses

Figure 1 and **Table S2 in supplementary file** summarize the responses to all 40 items.

Scope of the AMR problem and key contributors Most respondents agreed that AMR is an important problem in Indonesia (93.8%; 944/1006) [Q2]; in communities outside of the hospital (83.6%; 838/1003) [Q6]; and at their hospital (80.4%; 808/1005) [Q1], with 30.9% (311/1005) agreeing that patients are likely to develop an infection with a multidrug-resistant infection [Q10]. Most acknowledged as key contributing factors: overuse of antimicrobial drugs (95.1%; 954/1003) [Q3], lack of hand hygiene (71.1%; 715/1005) [Q4], use of broad-spectrum antibiotics (80.5%; 808/1004) [Q5]. Current infection and prevention control (IPC) practices at their hospital were regarded as suboptimal: 64.8% (651/1004) thought that patient rooms are cleaned according to hospital protocol after discharge of a patient with a multidrug-resistant organism [Q7]; 56.9% (570/1002) thought adherence to hand-hygiene protocols to be excellent [Q8]; and 22.5% (226/1005) felt that their hospital does not provide adequate staff education regarding multidrug-resistant organisms [Q9].

Antibiotic prescribing practices Whereas most respondents (85.7%; 861/1005) agreed that antibiotics are overused in Indonesia (Q11), only 35.5% (357/1005) acknowledged this to be case at their hospital [Q12]. Most agreed that more judicious antimicrobial prescribing practices would decrease AMR (94.8%; 953/1005) [Q17] and that following evidence-based antibiotic guidelines will help optimize treatment outcomes (95.3%; 958/1006) [Q18]. Most gave high priority to rational antibiotic prescribing to their patients (88.8%; 892/1005) [Q19], and considered developing hospital antibiotic guidelines more useful than applying international guidelines (78.4%; 787/1004) [Q20]. Nearly a quarter of respondents indicated to be often unsure if a patient needs an antibiotic or not (23.6%; 237/1006) [Q21] or which antibiotic to prescribe (21.3%; 215/1006) [Q22]. A small but considerable fraction expressed lack of confidence in prescribing decisions, i.e., 12.2% (123/1005) prescribed in patients with just a high fever (≥39°C) [Q24], 36.6% (368/1005) when they felt uncertain about the diagnosis of infection [Q25], 35.0% (352/1006) prescribed more freely because of fear of clinical failure [Q26], and 9.8% (98/1005) frequently prescribed antibiotics because patients or their relatives insist [Q27]. Just more than half of the respondents reported that

For peer review only - http://bmjopen.bmj.com/site/about/guidelines.xhtml

microbiology laboratory results are efficiently communicated to the treating physician (57.3%; 576/1005) [Q13], considered the hospital antibiogram when empirically prescribing antibiotics (54.5%; 548/1005) [Q14], and would stop antibiotics that others have prescribed in the absence of an appropriate indication (57.0%; 571/1002) [Q23]. Most (72.7%; 731/1006) agreed that, if medically appropriate, intravenous antibiotics should be stepped down to an oral alternative after three days [Q15]. Notably, 33.6% (338/1006) felt that restrictions on antibiotics impaired their ability to provide good patient care [Q16].

Antibiotic stewardship When asked about AMS in general, most respondents were aware that their hospital had an AMS programme (93.1%; 937/1006) [Q28], they reported they understood its purpose (92.1%; 927/1006) [Q29], and they agreed that AMS can improve patient care (88.6%; 891/1006) [Q30], reduce AMR (88.3%; 887/1005) [Q31] and hospital-acquired infections (76.7%; 770/1004) [Q32]. When asked about the usefulness of specific AMS activities, most respondents acknowledged that additional education on antibiotic prescribing was needed (88.4%; 888/1005) [Q33], regular audit and feedback encouraged them to prescribe antibiotics prudently (92.2%; 928/1006) [Q34], rapid and accurate diagnostic tests are useful for diagnosis of infectious diseases and guidance on antibiotic therapy (96.5%; 971/1006) [Q35], implementation of antibiotic restriction (e.g. antibiotic tiers) can reduce antibiotic overuse in hospitals (90.4%; 910/1007) [Q36], regular consultations or ward rounds with a clinical microbiologist or infectious disease physician can curb AMR (85.4%; 859/1006) [Q37], timely access to microbiological test results is needed to guide antibiotic therapy (92.4%; 930/1006) [Q38], and IPC in the hospital can reduce AMR (95.3%; 959/1006) [Q40].

Exploratory factor analysis

The KMO was 0.8773 overall and >0.5 for all items, suggesting the data were suitable for EFA. Analysis of the scree plot (**supplementary file Figure S1**) indicated a case for four factors, whereas the parallel analysis (**supplementary file Figure S2**) indicated a case for seven factors. The four-factor solution yielded strong factors but explained only 39.9% of the variance and lacked a theoretical basis for one factor. The seven-factor solution contained one factor with only three items that was difficult to interpret; two of these items (Q9 and Q10) did not load well with any factor in various alternative factor solutions and were removed. Therefore, a six-factor model with a clear theoretical basis based on 38 items was deemed most suitable, explaining 47.4% of the variance, with KMO 0.8802 overall and >0.5 for each item (**Table 2**). The six latent factors are (**Table 3**, see **supplementary file Table S3** for details): 1) Awareness of AMS activities; 2) Awareness of AMS purpose; 3) Views regarding rational antibiotic prescribing; 4) Confidence in antibiotic prescribing decisions; 5) Perception of AMR as a significant problem; and 6) Immediate actions to contain AMR.

For peer review only - http://bmjopen.bmj.com/site/about/guidelines.xhtml

Internal reliability was excellent for the overall 38-item scale (α=0.85) and factor 1 (α=0.8734) and 2 (α=0.8334), good for factor 3, 4 and 5 (α=0.70 each), and acceptable for factor 6 (α=0.57).

Physician subgroup analysis

Table 4 summarizes the results of the subgroup analysis.

Hospitals Statistically significant differences were identified between hospitals for awareness of AMS purposes (factor 2), views regarding rational antibiotic prescribing (factor 3), perception of AMR as a significant problem (factor 5) and immediate actions to contain AMR (factor 6), but not for awareness of AMS activities (factor 1) and confidence in antibiotic prescribing decisions (factor 4). None of the factor scores differed between prescribers in public versus private, or secondary versus tertiary hospitals.

Professional hierarchy For awareness of AMS activities (factor 1), consultants, GPs and residents scored higher than interns. For awareness of AMS purposes (factor 2), consultants scored lower than interns and residents. For views regarding rational antibiotic prescribing (factor 3), consultants scored higher than GPs. For confidence in antibiotic prescribing decisions (factor 4), consultants scored lower than residents, whereas for immediate actions to contain AMR (factor 6), consultants scored higher than residents. No differences were identified for perception of AMR as a significant problem (factor 5).

Departments For awareness of AMS activities (factor 1) and purpose (factor 2), physicians in surgery and medicine scored higher than the acute specialties, whereas for awareness of AMS activities (factor 1), medicine scored higher than surgery. For views regarding rational antibiotic prescribing (factor 3), surgery scored higher than acute specialties. For confidence in antibiotic prescribing decisions (factor 4), medicine scored lower than surgery and other specialties. For perception of AMR as a significant problem (factor 5), surgery scored lower than medicine. For immediate actions to contain AMR (factor 6), surgery scored higher than medicine and other specialties, and the acute specialties scored higher than surgery and medicine.

Work experience. Physicians with little (0-5 years) work experience scored lower than more experienced colleagues, for awareness of AMS purpose (factor 2); for perception of AMR as a significant problem (factor 5); and for immediate actions to contain AMR (factor 6). No differences were identified for factors 1, 3 and 4.

For peer review only - http://bmjopen.bmj.com/site/about/guidelines.xhtml

Discussion

This survey, among over 1000 physicians in Indonesian hospitals, assessed their perceptions and views regarding the AMR problem and its key contributors, antibiotic prescribing practices and AMS. Starting from those three broad themes, we used exploratory factor analysis to identify distinct underlying constructs in the dataset, i.e., 1) awareness of AMS activities; 2) awareness of AMS purposes; 3) views regarding rational antibiotic prescribing; 4) confidence in antibiotic prescribing decisions; 5) perception of AMR as a significant problem; and 6) immediate actions to contain AMR. The survey findings thus outline a series of dynamics around AMR and AMS in the Indonesian context. Spanning issues around *awareness* (education)[28] (factors 1, 2, 5), *visibility* (diagnostics)[29] (factor 3, 4 and 6), and *institutional form* (governance)[30] (factors 3 and 6), the survey results tease out many of the core issues illustrated in other settings[4,5], but in turn, illustrate that several specific factors play a role, i.e. uncertainty, risk, and lack of sense of responsibility.

In this respect, an important finding was that only few physicians recognized that antibiotic overuse was an issue at their own hospital, that many physicians were hesitant to stop antibiotics without an appropriate indication that others had prescribed, and that many felt that antibiotic restrictions impaired their ability to provide good patient care. Lack of confidence in prescribing decisions and defensive prescribing were common due to diagnostic uncertainty, fear of patient deterioration or complications, or because patients or their relatives insisted. These views and perceptions concur with the observed overuse of broad-spectrum antibiotics, the underuse of diagnostic stewardship to guide antibiotic decisions and poor guideline compliance of antibiotic prescribing in the same hospitals, described in our recent paper, which identified several priority areas for stewardship[17].

Based on the survey data, we considered that an important obstacle that hinders effective AMS implementation in this context could be linked to perceived “externality of AMR” as a problem[31]. That is, physicians acknowledge its significance but do not take ownership or responsibility, thus reflecting a production of AMR as an externality, e.g., a result of irrational use elsewhere in communities or other hospitals. The lack of systematic surveillance of AMR and antibiotic use and the underutilisation of bacterial cultures, recognised by many of the respondents, also reinforces the perception of AMR as a “problem of elsewhere”. This feeds a lack of engaging with AMS, as physicians do not recognise it as a value-add for an already stretched institutional context, and in turn provides the context for continued defensive prescribing “to be on the safe side”. Moreover, defensive prescribing practices may somewhat offset (in the short term) problems around room cleaning, hand hygiene and staff education. In this way, AMR as an externality and the vulnerabilities of the institution, offer

For peer review only - http://bmjopen.bmj.com/site/about/guidelines.xhtml

an environment conducive to the ongoing over-use of antimicrobials[32,33]. The higher incidence of hospital-acquired infections in LMIC than in high-income countries could further promote defensive prescribing as a way to compensate for substandard IPC practices[34]. All in all, this supports the notion that physicians tend to prioritise managing immediate clinical risks, reputation and concordance with peer practice, vis-à-vis the long-term population consequences of AMR[35].

The identified differences between hospitals regarding awareness of AMS purposes, views regarding rational antibiotic prescribing, perception of AMR as a significant problem and immediate actions to contain AMR could not be attributed or related to the pertinent hospital sector (public versus private) or health care level (secondary versus tertiary), but they were found to be associated with several differences between the physician subgroups. First of all, work experience and medical hierarchy were found to influence the awareness of AMS purpose, AMR as a significant problem, and immediate actions to contain AMR. Interestingly, compared with junior physicians, specialists/consultants expressed lower confidence to make antibiotic decisions in uncertain situations while showing higher confidence in actions to contain AMR. Possible explanations include that specialists/consultants have a better recognition of the "unknowns" (e.g. lack of data on bacterial susceptibility patterns) and that they bear final patient responsibility, introducing the fear of losing a patient or legal consequences[36], whereas taking actions to curb AMR can be a remedy to compensate their fear. Conversely, residents' higher confidence in antibiotic prescribing may also relate to their contemporary medical training, which promotes the use of evidence-based prescribing guidelines, as opposed to late-career physicians[37]. GPs had low scores on views regarding rational antibiotic prescribing compared with consultants which could reflect the GPs' limited responsibility in the antibiotic decision-making hierarchy, possibly leading to a lack of positive attitude towards guidelines and preference for complying with them[38,39].

Furthermore, the acute specialties (including emergency, ICU, and anaesthesiology) had lower awareness of AMS activities and purposes, compared with surgery or medicine, but scored higher for immediate actions to contain AMR. Compared with surgeons, physicians in medicine had a greater awareness of AMS activities and recognition of AMR as a significant problem, but they had lower confidence in antibiotic prescribing decisions and immediate actions to contain AMR. These observations are in line with a UK study that found that emergency physicians experienced pressure for immediate action out of fear of losing a patient and a lack of ownership of antibiotic decision-making due to the patient transitioning to inpatient care, that medical doctors adopted a more policy-informed, interdisciplinary approach, and that senior surgeons left complex antibiotic decisions to junior staff, resulting

For peer review only - http://bmjopen.bmj.com/site/about/guidelines.xhtml

in potential defensive and inappropriate antibiotic use[19]. Variations in the social norms, values and behaviours between specialties should inform what is the best approach to antibiotic decision-making.

Our study had several strengths and weaknesses. First, we were able to identify a relevant set of attributes through a factor analysis optimization process, with adequate content, face and construct validity and internal reliability. In the absence of adequately validated instruments regarding AMR and AMS[40], this study adds important value to the field, with particular relevance and applicability for LMIC. Nonetheless, further questionnaire validation steps (such as criterion-related validity) are necessary to achieve a fully valid and reliable instrument. Second, the study had a large, varied respondent sample and high response rate. However, non-participation and the purposive and limited hospital sample could have introduced selection bias, and the data are not necessarily representative for Jakarta or Indonesia at large. The authenticity of the answers was maximised by protecting the respondents' identities, although reliance on self-report has potential for social desirability bias. Third, the present study focused on hospital settings. It is, however, acknowledged that the problem of AMR extends beyond hospital settings to the wider community through the primary health care system as well as informal providers, which warrants further study in the Indonesian context[41]. Fourth, factor analysis is based on using a "heuristic", which leaves room to more than one interpretation of the same data and cannot identify causality.

Conclusion

AMS implementation in Indonesian hospitals is likely highly dependent on institutional, contextual and diagnostic vulnerabilities. These may result in the problem of AMR being externalised, instead of recognised as a local hospital problem. Current AMS strategies may be insufficiently successful in promoting prudent antibiotic use, due to lack of systematic engagement with and feedback to prescribers, aimed at building confidence in antibiotic decision-making and ownership of the AMR problem. Appropriate recognition of the contextual and social determinants of antibiotic prescribing decision-making, including hospital factors, dynamics in medical hierarchy and experience, among others, will be critical to design context-specific AMS interventions that are adopted by healthcare professionals and successfully influence behaviours[14].

For peer review only - http://bmjopen.bmj.com/site/about/guidelines.xhtml

Acknowledgements

The authors are grateful to the management, research/medical committees and clinicians of the participating hospitals for their support to the study.

EXPLAIN study group

Erni J. Nelwan, Ralalicia Limato, Manzilina Mudia, Monik Alamanda, Helio Guterres, Enty Enty, Elfrida R Manurung, Ifael Y Mauleti, Maria Mayasari, Iman Firmansyah, May Hizrani, Roswin Djaafar, Anis Karuniawati, Prof Taralan Tambunan, Prof Amin Soebandrio, Decy Subekti, Iqbal Elyazar, Mutia Rahardjani, Fitria Wulandari, Prof Reinout van Crevel, H Rogier van Doorn, Vu Thi Lan Huong, Nga Do Ti Thuy, Sonia Lewycka, Prof Alex Broom, Raph L Hamers.

Funding

This work was funded by the Wellcome Trust, UK (106680/Z/14/Z). RL is supported by an OUCRU Prize Studentship and a Nuffield Dept of Medicine Tropical Network Fund DPhil Bursary. The funders of this study had no role in study design, data collection, data analysis, data interpretation, or writing of the report.

Conflicts of interest

The authors declare no competing interests.

Author contributions

EJN and RLH conceived the idea for the study and are the principal investigators. RLH and RL obtained the funding. RL, EJN, HVTL and RLH designed the study protocol and developed the study instrument. MM, ERM, IYM, MM, IF, and RD collected and verified the data, overseen by RL. RL, MM, MA created and curated the database. RLH and MA performed the analysis and had full access to all study data. RL, MA, and RLH drafted the paper, with critical inputs from EJN, HVTL, HRvD and AB. All authors critically revised the manuscript and gave approval for the final version to be published.

Data Availability Statement

De-identified data are available upon reasonable request via the corresponding author, after written permission has been obtained from the lead investigators.

For peer review only

For peer review only - http://bmjopen.bmj.com/site/about/guidelines.xhtml

References

1 O'Neill J. Tackling Drug-Resistant Infections Globally: Final Report and Recommendations. 2016.

2 Klein EY, Van Boeckel TP, Martinez EM, *et al.* Global increase and geographic convergence in antibiotic consumption between 2000 and 2015. *Proc Natl Acad Sci U S A* 2018; **115**: E3463–70.

3 Goossens H, Ferech M, Vander Stichele R, Elseviers M. Outpatient antibiotic use in Europe and association with resistance: a cross-national database study. *Lancet* 2005; **365**: 579–87.

4 Chaw PS, Höpner J, Mikolajczyk R. The knowledge, attitude and practice of health practitioners towards antibiotic prescribing and resistance in developing countries—A systematic review. *J Clin Pharm Ther* 2018; **43**: 606–13.

5 Md Rezal RS, Hassali MA, Alrasheedy AA, Saleem F, Md Yusof FA, Godman B. Physicians' knowledge, perceptions and behaviour towards antibiotic prescribing: A systematic review of the literature. *Expert Rev Anti Infect Ther* 2015; **13**: 665–80.

6 Do NTT, Vu HTL, Nguyen CTK, *et al.* Community-based antibiotic access and use in six low-income and middle-income countries: a mixed-method approach. *Lancet Glob Heal* 2021; published online April. DOI:10.1016/S2214-109X(21)00024-3.

7 Ayobami O, Brinkwirth S, Eckmanns T, Markwart R. Antibiotic resistance in hospital-acquired ESKAPE-E infections in low- and lower-middle-income countries: a systematic review and meta-analysis. Taylor & Francis, 2022 DOI:10.1080/22221751.2022.2030196.

8 Kim SH, Song JH, Chung DR, *et al.* Changing trends in antimicrobial resistance and serotypes of Streptococcus pneumoniae isolates in Asian countries: An Asian Network for Surveillance of Resistant Pathogens (ANSORP) study. *Antimicrob Agents Chemother* 2012; **56**: 1418–26.

9 Schuts EC, Hulscher MEJL, Mouton JW, *et al.* Current evidence on hospital antimicrobial stewardship objectives: A systematic review and meta-analysis. *Lancet Infect Dis* 2016; **16**: 847–56.

10 Honda H, Ohmagari N, Tokuda Y, Mattar C, Warren DK. Antimicrobial stewardship in inpatient settings in the Asia pacific region: A systematic review and meta-analysis. *Clin Infect Dis* 2017; **64**: S119–26.

11 Laundy M, Gilchrist M, Whitney L. Antimicrobial Stewardship. Oxford, UK: Oxford University Press, 2016 DOI:10.1093/med/9780198758792.001.0001.

12 Rolfe R, Kwobah C, Muro F, *et al.* Barriers to implementing antimicrobial stewardship programs in three low- and middle-income country tertiary care settings: findings from a multi-site qualitative study. *Antimicrob Resist Infect Control* 2021; **10**: 60.

For peer review only - http://bmjopen.bmj.com/site/about/guidelines.xhtml

13 Teixeira Rodrigues A, Roque F, Falcão A, Figueiras A, Herdeiro MT. Understanding physician antibiotic prescribing behaviour: a systematic review of qualitative studies. *Int J Antimicrob Agents* 2013; **41**: 203–12.

14 Aveling E-L, Martin G, Armstrong N, Banerjee J, Dixon-Woods M. Quality improvement through clinical communities: eight lessons for practice. *J Health Organ Manag* 2012; **26**: 158–74.

15 Parathon H, Kuntaman K, Widiastoety TH, *et al.* Progress towards antimicrobial resistance containment and control in Indonesia. *BMJ* 2017; **358**: 31–5.

16 Herawati F, Ananta SC, Parwitha IAA, *et al.* Interview-based cross-sectional needs assessment to advance the implementation of an effective antibiotic stewardship program in Indonesian hospitals. *Heal Policy OPEN* 2020; **1**: 100002.

17 Limato R, Nelwan EJ, Mudia M, *et al.* A multicentre point prevalence survey of patterns and quality of antibiotic prescribing in Indonesian hospitals. *JAC-Antimicrobial Resist* 2021; **3**. DOI:10.1093/jacamr/dlab047.

18 Badan Pusat Statistik Provinsi DKI Jakarta (BPS-Statistics of DKI Jakarta Province). Jumlah Rumah Sakit dan Tempat Tidur yang Tersedia Menurut Kabupaten/Kota Administrasi dan Status Rumah Sakit di Provinsi DKI Jakarta 2018-2020. Badan Pus. Stat. Provinsi DKI Jakarta. 2021. https://jakarta.bps.go.id/indicator/30/539/1/jumlah-rumah-sakit-dan-tempat-tidur-yang-tersedia-menurut-kabupaten-kota-administrasi-dan-status-rumah-sakit-di-provinsi-dki-jakarta.html (accessed Dec 20, 2021).

19 Charani E, Ahmad R, Rawson TM, Castro-Sanchèz E, Tarrant C, Holmes AH. The Differences in Antibiotic Decision-making Between Acute Surgical and Acute Medical Teams: An Ethnographic Study of Culture and Team Dynamics. *Clin Infect Dis* 2019; **69**: 12–20.

20 Greater New York Association United Hospital Fund. Antimicrobial Stewardship Toolkit: Best practices from the GNYHA/UHF antimicrobial stewardship collaborative. 2011 https://uhfnyc.org/media/filer_public/4a/49/4a497bf9-d0a1-44c0-8849-874af1bb6019/antimicrobial_stewardship.pdf.

21 Chaw PS, Schlinkmann KM, Raupach-Rosin H, *et al.* Knowledge, attitude and practice of Gambian health practitioners towards antibiotic prescribing and microbiological testing: a cross-sectional survey. *Trans R Soc Trop Med Hyg* 2017; **111**: 117–24.

22 Asante KP, Boamah EA, Abdulai MA, *et al.* Knowledge of antibiotic resistance and antibiotic prescription practices among prescribers in the Brong Ahafo Region of Ghana; a cross-sectional study. *BMC Health Serv Res* 2017; **17**: 422.

23 Labi A-K, Obeng-Nkrumah N, Bjerrum S, *et al.* Physicians' knowledge, attitudes, and perceptions concerning antibiotic resistance: a survey in a Ghanaian tertiary care hospital. *BMC Health Serv Res* 2018; **18**: 126.

For peer review only - http://bmjopen.bmj.com/site/about/guidelines.xhtml

24 Venugopalan V, Trustman N, Manning N, Hashem N, Berkowitz L, Hidayat L. Administration of a survey to evaluate the attitudes of house staff physicians towards antimicrobial resistance and the antimicrobial stewardship programme at a community teaching hospital. *J Glob Antimicrob Resist* 2016; **4**: 21–7.

25 García C, Llamocca LP, García K, *et al.* Knowledge, attitudes and practice survey about antimicrobial resistance and prescribing among physicians in a hospital setting in Lima, Peru. *BMC Clin Pharmacol* 2011; **11**: 18.

26 Alumran A, Hou X-Y, Hurst C. Validity and reliability of instruments designed to measure factors influencing the overuse of antibiotics. *J Infect Public Health* 2012; **5**: 221–32.

27 Alothman A, Algwizani A, Alsulaiman M, Alalwan A, Binsalih S, Bosaeed M. Knowledge and Attitude of Physicians toward Prescribing Antibiotics and the Risk of Resistance in Two Reference Hospitals. *Infect Dis Res Treat* 2016; **9**: IDRT.S40047.

28 Charoenboon N, Haenssgen MJ, Warapikuptanun P, Xayavong T, Khine Zaw Y. Translating antimicrobial resistance: a case study of context and consequences of antibiotic-related communication in three northern Thai villages. *Palgrave Commun* 2019; **5**: 23.

29 Chandler CI., Hutchinson E, Hutchison C. Addressing Antimicrobial Resistance Through Social Theory: An Anthropologically Oriented Report. London School of Hygiene & Tropical Medicine. 2016.

30 Broom A, Doron A. Antimicrobial Resistance, Politics, and Practice in India. *Qual Health Res* 2020; **30**: 1684–96.

31 Broom A, Broom J, Kirby E, Gibson A, Davis M. Antibiotic optimisation in 'the bush': Local know-how and core-periphery relations. *Health Place* 2017; **48**: 56–62.

32 Broom A, Kirby E, Gibson AF, Post JJ, Broom J. Myth, Manners, and Medical Ritual: Defensive Medicine and the Fetish of Antibiotics. *Qual Health Res* 2017; **27**: 1994–2005.

33 Will CM. Editorial: Beyond behavior? Institutions, interactions and inequalities in the response to antimicrobial resistance. Sociol. Health Illn. 2018; **40**: E1–9.

34 Chandler CIR. Current accounts of antimicrobial resistance: stabilisation, individualisation and antibiotics as infrastructure. *Palgrave Commun* 2019; **5**: 53.

35 Broom A, Broom J, Kirby E. Cultures of resistance? A Bourdieusian analysis of doctors' antibiotic prescribing. *Soc Sci Med* 2014; **110**: 81–8.

36 Broom A, Broom J, Kirby E, Adams J. The social dynamics of antibiotic use in an Australian hospital. *J Sociol* 2015; **52**: 824–39.

37 Fernandez-Lazaro CI, Brown KA, Langford BJ, Daneman N, Garber G, Schwartz KL. Late-career Physicians Prescribe Longer Courses of Antibiotics. *Clin Infect Dis* 2019; **69**: 1467–75.

For peer review only - http://bmjopen.bmj.com/site/about/guidelines.xhtml

38 R Hansen C, Bradley CP, Sahm LJ. Factors Influencing Successful Prescribing by Intern Doctors: A Qualitative Systematic Review. *Pharm (Basel, Switzerland)* 2016; **4**: 24.

39 Roumie CL, Halasa NB, Edwards KM, Zhu Y, Dittus RS, Griffin MR. Differences in antibiotic prescribing among physicians, residents, and nonphysician clinicians. *Am J Med* 2005; **118**: 641–8.

40 DeVon HA, Block ME, Moyle-Wright P, *et al.* A psychometric toolbox for testing validity and reliability. *J Nurs Scholarsh an Off Publ Sigma Theta Tau Int Honor Soc Nurs* 2007; **39**: 155–64.

41 Frost I, Laxminarayan R, McKenna N, Chai S, Joshi J. Antimicrobial resistance and primary health care. *World Heal Organ* 2018; : 3–6.

For peer review only

Table 1 Characteristics of respondents

**Total**	**1007**
Sex [a]	
Female	477 (47.4)
Male	524 (52.0)
Professional hierarchy	
Intern/Internship doctor	10 (1.0)
General practitioner	113 (11.2)
Resident	500 (49.7)
Specialist/consultant	358 (35.6)
Other	18 (1.8)
Professional experience (years) [b]	
<1	194 (19.3)
1-5	459 (45.6)
6-10	136 (13.5)
11-15	74 (7.4)
16-20	52 (5.2)
>20	81 (8.0)
Grouped departments [c,f]	
Surgery (including subspecialties)	371 (36.8)
Medicine (including subspecialties)	232 (23.0)
Acute specialties	156 (15.5)
Other departments	244 (24.2)
Information sources used to guide prescribing [d]	
Guidelines	
International	619 (61.5)
National	628 (62.4)
Hospital	656 (65.1)
Department/Division	405 (40.2)
Consultation with senior colleague(s)	472 (46.9)
Consultation with microbiologist/infectious disease physician	523 (51.9)
Textbooks	410 (40.7)
Medical journals	389 (38.6)
Pharmaceutical company representative	34 (3.4)
Internet	115 (11.4)
Other	13 (1.3)
Number of AMR/AMS trainings attended in the past year [e]	
0	396 (39.3)
1	342 (34.0)
≥2	168 (16.7)
Median (range)	1 (0, 10)

Abbreviations: AMR, antimicrobial resistance; AMS, antibiotic stewardship;
Data are reported as n (%) unless indicated otherwise.
Data missing for: [a] 6 (0.60%); [b] 11 (1.1%); [c] 4 (0.40%); [d] 2 (0.20%); [e] 101 (10.0%).
[f] Surgery and surgical subspecialties includes obstetrics/gynaecology (146), surgery (122), orthopaedics (57), ENT (32), urology (14); medicine and medical subspecialties includes medicine (128), neurology (63), pulmonology (15), dermatology (14), cardiology (12); acute specialties includes anaesthesiology (72), emergency (57), ICU (27); other departments includes paediatrics (54), ophthalmology (39), multiple units (33), rehabilitation (32), psychiatry (30), dentist (27), other (29) and unspecified (4).

For peer review only - http://bmjopen.bmj.com/site/about/guidelines.xhtml

Table 2 Summary of the exploratory factor analysis of the six-factor solution (n=973)

Item #	Original item	Rotated factor loadings						Uniqueness
		Factor 1	Factor 2	Factor 3	Factor 4	Factor 5	Factor 6	
**Q01**	Antimicrobial resistance is a significant problem in this hospital	0.2159	0.0410	0.1389	0.0057	0.5701	0.0416	0.6056
**Q02**	Antimicrobial resistance is a significant problem in Indonesia	0.2433	0.1073	0.3295	0.0588	0.5742	0.0385	0.4861
**Q03**	A cause of antimicrobial resistance is using too many antimicrobial drugs	0.1641	0.2006	0.3993	0.0695	0.5361	-0.0462	0.4790
**Q04**	Lack of hand disinfection by healthcare workers causes spread of antimicrobial resistance	0.0640	0.0902	-0.2533	0.0152	0.5725	0.1179	0.5817
**Q05**	Use of broad-spectrum antibiotics can increase antimicrobial resistance when narrower-spectrum antibiotics are available that are equally effective	0.2150	0.0536	0.1482	0.0005	0.5480	0.1093	0.6167
**Q06**	Antibiotic resistance is also a problem outside of the hospital, in communities	0.2088	0.0318	0.2463	0.0632	0.4766	0.0432	0.6617
**Q07**	In this hospital, patient rooms are cleaned according to hospital cleaning protocol once a patient with a multidrug-resistant organism (MDRO) has been discharged	0.1038	0.0753	0.0868	0.0210	0.0378	0.6058	0.6071
**Q08**	Adherence to hand-hygiene protocols is excellent at this hospital	-0.0098	0.0364	0.0606	0.1099	-0.0830	0.6368	0.5705
**Q11**	Antibiotics are overused in Indonesia	0.1924	0.1831	0.5138	-0.0809	0.2489	-0.2318	0.5432
**Q12**	Antibiotics are overused in this hospital	0.0548	0.0417	0.2094	-0.4550	0.1635	-0.2381	0.6610
**Q13**	Microbiology laboratory results are efficiently communicated to the treating physician	0.1689	0.1174	-0.0915	0.0532	-0.0963	0.5116	0.6754
**Q14**	I regularly refer to/consider the antibiotic susceptibility patterns at this hospital/institution (i.e. the institutional antibiogram) when empirically prescribing antibiotics	0.0288	0.1070	-0.0292	-0.0237	0.2056	0.6115	0.5701
**Q15**	If medically appropriate, intravenous antibiotics should be stepped down to an oral alternative after three days	-0.0873	0.2085	0.1141	-0.0850	0.2311	0.3617	0.7444
**Q16**	Restrictions on antibiotics impair my ability to provide good patient care	0.0460	0.1839	0.0527	0.4031	0.0843	-0.0868	0.7842
**Q17**	More judicious use of antibiotics would decrease antimicrobial resistance	0.3010	0.0747	0.7362	0.0820	0.0892	0.0648	0.3429
**Q18**	Following evidence-based antibiotic guidelines will help optimize treatment outcomes	0.2274	0.1934	0.6565	0.1206	0.1256	0.1851	0.4153

For peer review only - http://bmjopen.bmj.com/site/about/guidelines.xhtml

For peer review only

Item #	Original item	Rotated factor loadings						Uniqueness
Q19	In general, rational antibiotic prescribing for my patients is high on my list of priorities	0.1845	0.1804	0.5246	0.1574	0.1184	0.3228	0.5151
Q20	Developing hospital antibiotic guidelines is more useful than applying international guidelines	0.1803	0.0075	0.3499	-0.1335	0.2631	0.0672	0.7534
Q21	I am often unsure if a patient needs an antibiotic or not	0.0489	0.0260	0.0497	0.5640	-0.3364	0.1021	0.5527
Q22	I am often unsure which antibiotic to prescribe	-0.0084	0.0457	0.1256	0.5670	-0.1938	0.0781	0.6170
Q23	I will stop antibiotics that others have prescribed in the absence of an appropriate indication	0.0016	-0.0620	0.1517	-0.1137	0.2090	0.3892	0.7650
Q24	Patients with high fever (≥39°C) must be treated with antibiotics	0.0077	0.1331	0.1695	0.4794	0.2095	-0.2351	0.6245
Q25	If I am uncertain about the diagnosis of infection, but think it is possible, I feel safer prescribing an antibiotic	0.0217	-0.0415	-0.0877	0.6741	0.1927	-0.0086	0.4985
Q26	Fear of patient deterioration or complications leads me to prescribe antibiotics more freely	0.0113	0.0039	-0.1459	0.7092	0.0883	0.0246	0.4672
Q27	I frequently prescribe antibiotics because patients or their relatives insist on it	0.1069	0.0474	0.2869	0.6318	0.0518	-0.0100	0.5021
Q28	I am aware that my hospital has an antimicrobial stewardship program (ASP)	0.2434	0.6224	0.2918	0.0866	0.0418	0.0110	0.4588
Q29	I understand what the purpose of ASP is	0.2217	0.6957	0.2635	0.1188	0.0092	0.0199	0.3828
Q30	ASP improve patient care	0.2364	0.7744	0.0873	0.0932	0.0852	0.1241	0.3055
Q31	ASP reduces the problem of antimicrobial resistance	0.2775	0.7532	0.0949	0.0172	0.0906	0.1753	0.3074
Q32	ASP reduces this hospital's infection rates	0.2045	0.6670	-0.1122	0.0569	0.1283	0.1799	0.4486
Q33	Additional staff education on antimicrobial prescribing is needed	0.5202	0.2816	0.0917	-0.0079	0.2203	-0.0284	0.5923
Q34	Regular audit and feedback encourage me to prescribe antibiotics prudently	0.6151	0.3581	0.0075	0.0064	0.1402	0.1117	0.4612
Q35	Rapid and accurate diagnostic tests are useful for diagnosis of infectious diseases and guidance on antibiotic therapy	0.6714	0.2576	0.1522	-0.0362	0.0386	0.0379	0.4555
Q36	To reduce antibiotic overuse in hospitals, implementation of antibiotic restriction (e.g. antibiotic tiers) is a useful measure	0.6428	0.2670	0.1088	-0.0335	0.2153	0.0013	0.4562
Q37	To curb antimicrobial resistance, regular consultations or ward rounds with a clinical microbiologist or infectious disease physician are useful	0.7046	0.0787	0.0061	0.0190	0.1881	0.0781	0.4555
Q38	To curb antimicrobial resistance, doctors need to have timely access to microbiological test results to guide antibiotic therapy	0.7197	0.0835	0.2786	0.0985	0.0670	0.0820	0.3765

For peer review only - http://bmjopen.bmj.com/site/about/guidelines.xhtml

Item #	Original item	Rotated factor loadings						Uniqueness
**Q39**	Up-to-date information on hospital antimicrobial resistance patterns is important for developing hospital antibiotic guidelines	0.7374	0.0854	0.3223	0.1088	0.1739	0.0348	0.3018
**Q40**	Effective infection prevention and control in the hospital reduces antimicrobial resistance	0.7067	0.1690	0.2876	0.0950	0.1798	0.0533	0.3452
	**Eigenvalues**	**4.39**	**3.19**	**2.82**	**2.78**	**2.65**	**2.17**	
	**% of variance explained**	**11.56%**	**8.40%**	**7.43%**	**7.32%**	**6.98%**	**5.72%**	**Overall 47.4%**

Table shows the results of the exploratory factor analysis (principal axis factoring) with orthogonal varimax rotation of the six-factor solution using the factor, pcf command in Stata.
Rotated factor loadings: a measure of how much each item contributes to the factor. Loadings close to -1 or 1 indicate that the factor strongly affects the item and loadings close to 0 indicate that the factor has a weak effect on the item.
Uniqueness: shows the proportion of the item's variance that is not explained by the factors.
Item #9 and 10 were excluded from the analysis as explained in Results.

For peer review only

Table 3 The latent factors of antibiotic prescribing

Factor	Factor label	Number of items	Original item #	Loadings range	Reliability (Cronbach's α)
**1**	Awareness of AMS activities	8	33-40	0.5202, 0.7374	0.8734
**2**	Awareness of AMS purposes	5	28-32	0.6224, 0.7744	0.8334
**3**	Views regarding rational antibiotic prescribing	5	11, 17-20	0.3499, 0.7362	0.6961
**4**	Confidence in antibiotic prescribing decisions	8	12, 16, 21, 22, 24-27	0.4031, 0.7092	0.6997
**5**	Perception of AMR as a significant problem	6	1-6	0.4766, 0.5742	0.6967
**6**	Immediate actions to contain AMR	6	7, 8, 13-15, 23	0.3617, 0.6368	0.5695

Abbreviations: AMR, antimicrobial resistance; AMS, antibiotic stewardship.
Item #9 and 10 were excluded from the analysis, as explained in Results.
The full table is included in Table S2.

For peer review only

Table 4 Physician subgroup analysis

	Factor 1			Factor 2			Factor 3			Factor 4			Factor 5			Factor 6		
	Coeff	95% CI	P	Coeff	95% CI	P	Coeff	95% CI	P	Coeff	95% CI	P	Coeff	95% CI	P	Coeff	95% CI	P
**Hospital**																		
**05**	Ref			Ref			Ref			Ref			Ref			Ref		
**01**	0.194	-0.089, 0.477	0.179	**-0.422**	**-0.701, -0.142**	**0.003**	0.244	-0.039, 0.527	0.091	-0.167	-0.451, 0.117	0.248	**-0.430**	**-0.716, -0.145**	**0.003**	**-0.504**	**-0.778, -0.23**	**0.000**
**02**	0.064	-0.13, 0.258	0.518	**-0.352**	**-0.543, -0.161**	**0.000**	0.134	-0.06, 0.328	0.174	-0.180	-0.375, 0.014	0.069	-0.095	-0.29, 0.101	0.342	-0.087	-0.274, 0.101	0.366
**03**	0.164	-0.116, 0.444	0.252	-0.066	-0.343, 0.211	0.641	**0.387**	**0.107, 0.668**	**0.007**	0.131	-0.151, 0.412	0.363	-0.044	-0.327, 0.238	0.758	0.092	-0.18, 0.364	0.507
**04**	0.117	-0.218, 0.453	0.494	0.116	-0.215, 0.448	0.492	0.096	-0.239, 0.432	0.573	0.249	-0.088, 0.586	0.148	-0.081	-0.419, 0.257	0.638	**0.361**	**0.035, 0.686**	**0.030**
**06**	0.196	-0.153, 0.544	0.271	0.106	-0.239, 0.45	0.548	**0.419**	**0.07, 0.768**	**0.019**	0.013	-0.338, 0.363	0.944	**-0.432**	**-0.783, -0.08**	**0.016**	**0.479**	**0.141, 0.817**	**0.005**
**Departments**																		
**Surgical**	Ref			Ref			Ref			Ref			Ref			Ref		
**Medical**	**0.239**	**0.073, 0.405**	**0.005**	0.150	-0.014, 0.314	0.073	-0.120	-0.286, 0.046	0.156	**0.307**	**0.140, 0.473**	**0.000**	**0.221**	**0.054, 0.389**	**0.009**	**-0.398**	**-0.559, -0.237**	**0.000**
**Acute**	**-0.275**	**-0.488, -0.062**	**0.011**	**-0.214**	**-0.425, -0.004**	**0.046**	**-0.273**	**-0.486, -0.06**	**0.012**	0.169	-0.045, 0.382	0.122	0.124	-0.091, 0.338	0.258	**0.242**	**0.036, 0.449**	**0.021**
**Other**	-0.081	-0.253, 0.092	0.358	-0.027	-0.197, 0.144	0.760	-0.086	-0.259, 0.086	0.326	0.046	-0.127, 0.219	0.603	0.119	-0.055, 0.292	0.181	**-0.302**	**-0.469, -0.135**	**0.000**
**Medical**	Ref			Ref			Ref			Ref			Ref			Ref		
**Acute**	**-0.514**	**-0.745, -0.283**	**0.000**	**-0.364**	**-0.593, -0.136**	**0.002**	-0.153	-0.384, 0.078	0.194	-0.138	-0.370, 0.094	0.243	-0.098	-0.330, 0.135	0.411	**0.640**	**0.417, 0.864**	**0.000**
**Other**	**-0.320**	**-0.507, -0.133**	**0.001**	-0.177	-0.361, 0.0083	0.061	0.034	-0.154, 0.221	0.724	**-0.261**	**-0.449, 0.073**	**0.006**	-0.103	-0.291, 0.086	0.285	0.096	-0.085, 0.277	0.298
**Medical Hierarchy**																		
**Consultant**	Ref			Ref			Ref			Ref			Ref			Ref		
**Intern**	**-0.744**	**-1.374, -0.115**	**0.020**	**0.637**	**0.015, 1.259**	**0.045**	0.443	-0.187, 1.074	0.168	0.067	-0.565, 0.699	0.836	0.495	-0.14, 1.129	0.126	0.003	-0.607, 0.613	0.993
**GP**	0.168	-0.096, 0.432	0.212	0.153	-0.108, 0.413	0.251	**-0.397**	**-0.661, -0.133**	**0.003**	-0.169	-0.434, 0.095	0.210	-0.011	-0.276, 0.255	0.938	-0.024	-0.279, 0.232	0.857
**Resident**	-0.091	-0.287, 0.105	0.363	**0.297**	**0.104, 0.490**	**0.003**	0.004	-0.192, 0.199	0.971	**-0.271**	**-0.468, -0.075**	**0.007**	0.058	-0.139, 0.255	0.563	**-0.202**	**-0.391, -0.012**	**0.037**
**Other**	-0.013	-0.491, 0.466	0.958	0.148	-0.325, 0.620	0.541	0.252	-0.227, 0.731	0.303	-0.172	-0.653, 0.308	0.482	-0.013	-0.495, 0.469	0.959	0.304	-0.159, 0.768	0.198
**Intern**	Ref			Ref			Ref			Ref			Ref			Ref		
**GP**	**0.912**	**0.245, 1.579**	**0.007**	-0.485	-1.144, 0.175	0.150	-0.840	-1.508, -0.172	0.014	-0.236	-0.906, 0.434	0.489	-0.505	-1.18, 0.167	0.141	-0.0263	-0.673, 0.620	0.936
**Resident**	**0.653**	**0.0458, 1.261**	**0.035**	-0.340	-0.941, -0.260	0.267	-0.440	-1.048, 0.169	0.157	-0.338	-0.948, 0.272	0.278	-0.436	-1.049, 0.176	0.162	-0.205	-0.793, 0.384	0.496
**Other**	0.732	-0.457, 1.509	0.065	-0.490	-1.258, 0.279	0.212	-0.191	-0.970, 0.587	0.630	-0.239	-1.020, 0.542	0.548	-0.507	-1.291, 0.276	0.204	0.302	-0.452, 1.055	0.433
**Resident**	Ref			Ref			Ref			Ref			Ref			Ref		
**GP**	0.259	-0.037, 0.555	0.086	-0.145	-0.437, 0.148	0.333	-0.400	-0.696, -0.104	0.008	0.102	-0.196, 0.399	0.503	-0.069	-0.367, 0.229	0.651	0.178	-0.108, 0.465	0.223
**Other**	0.0781	-0.417, 0.574	0.757	-0.150	-0.639, 0.340	0.550	0.248	-0.248, 0.744	0.326	0.099	-0.399, 0.596	0.697	-0.710	-0.570, 0.428	0.780	**0.506**	**-0.163, 1.29**	**0.039**
**Years in current profession**																		
**0-5**	Ref			Ref			Ref			Ref			Ref			Ref		
**6-10**	0.089	-0.122, 0.299	0.410	0.225	**0.017, 0.434**	**0.034**	-0.089	-0.300, 0.122	0.410	0.094	-0.118, 0.305	0.385	**0.290**	**0.077, 0.502**	**0.008**	0.053	-0.151, 0.258	0.608
**11-15**	0.087	-0.185, 0.358	0.532	-0.015	-0.283, 0.254	0.915	-0.066	-0.338, 0.207	0.637	0.271	-0.002, 0.544	0.052	0.156	-0.118, 0.43	0.265	-0.095	-0.358, 0.169	0.481
**>15**	0.117	-0.118, 0.353	0.328	0.194	-0.039, 0.427	0.102	-0.044	-0.279, 0.192	0.715	-0.057	-0.293, 0.179	0.637	**0.333**	**0.096, 0.57**	**0.006**	**0.366**	**0.138, 0.594**	**0.002**
**Public sector***	-0.064	-0.4, 0.273	0.710	0.264	-0.207, 0.736	0.272	-0.238	-0.575, 0.100	0.167	0.256	-0.835, 0.595	0.140	0.192	-0.148, 0.533	0.268	0.362	0.273, 0.996	0.264
**Tertiary level***	-0.093	-0.42, 0.234	0.577	-0.289	-0.767, 0.190	0.237	-0.047	-0.375, 0.281	0.779	-0.317	-0.647, 0.012	0.059	0.052	-0.279, 0.383	0.758	-0.407	-1.065, 0.25	0.225

Abbreviations: GP, general practitioner.

For peer review only - http://bmjopen.bmj.com/site/about/guidelines.xhtml

The table summarizes the results of the multivariable mixed-effect linear regression models to assess the associations between hospital (#1-6), department, medical hierarchy, work experience, health sector, health care level as the independent variables of interest and each of the factor scores (#1-6) as the dependent variable. Each model adjusted for the possible interdependence of observations clustered within hospitals, as well as for the confounders sex and AMS training.

* This model did not include hospital as an independent variable due to collinearity.

For peer review only

For peer review only - http://bmjopen.bmj.com/site/about/guidelines.xhtml

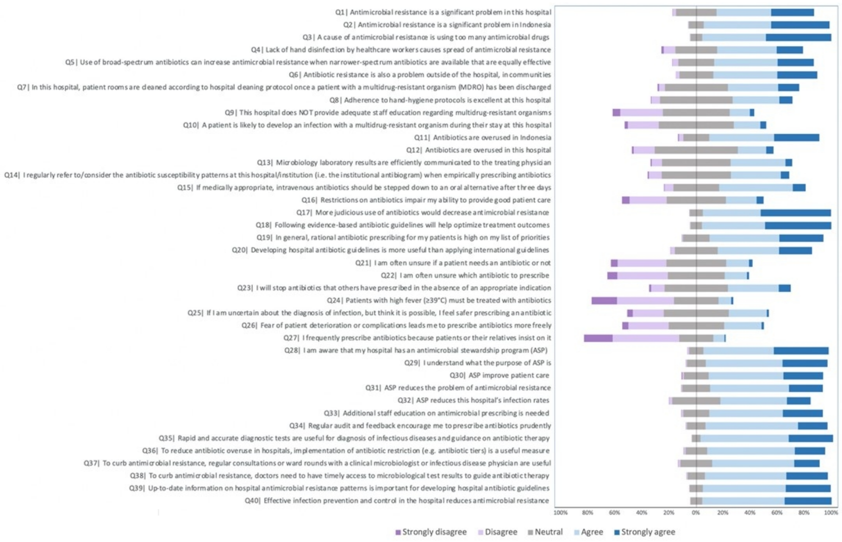

Caption: Figure 1. Five-point Likert scale responses for the 40-item questionnaire.
Legend: Acronyms: ASP, antibiotic stewardship program. Data (n, %) are summarized in supplementary file Table S2

338x190mm (96 x 96 DPI)

Appendix: Survey questionnaire

We kindly request you to indicate your agreement with the following 40 statements using a five-point scale:
① Strongly Disagree ② Disagree ③ Neutral or Don't Know ④ Agree ⑤ Strongly Agree

**ANTIMICROBIAL RESISTANCE: SCOPE OF THE PROBLEM AND KEY CONTRIBUTORS**						
**1.**	Antimicrobial resistance is a significant problem in this hospital	①	②	③	④	⑤
**2.**	Antimicrobial resistance is a significant problem in Indonesia	①	②	③	④	⑤
**3.**	A cause of antimicrobial resistance is using too many antimicrobial drugs	①	②	③	④	⑤
**4.**	Lack of hand disinfection by healthcare workers causes spread of antimicrobial resistance	①	②	③	④	⑤
**5.**	Use of broad-spectrum antibiotics can increase antimicrobial resistance when narrower-spectrum antibiotics are available that are equally effective	①	②	③	④	⑤
**6.**	Antibiotic resistance is also a problem outside of the hospital, in communities	①	②	③	④	⑤
**7.**	In this hospital, patient rooms are cleaned according to hospital cleaning protocol once a patient with a multidrug-resistant organism (MDRO) has been discharged	①	②	③	④	⑤
**8.**	Adherence to hand-hygiene protocols is excellent at this hospital	①	②	③	④	⑤
**9.**	This hospital does NOT provide adequate staff education regarding multidrug-resistant organisms	①	②	③	④	⑤
**10.**	A patient is likely to develop an infection with a multidrug-resistant organism during their stay at this hospital	①	②	③	④	⑤
**ANTIBIOTIC PRESCRIBING PRACTICES**						
**11.**	Antibiotics are overused in Indonesia	①	②	③	④	⑤
**12.**	Antibiotics are overused in this hospital	①	②	③	④	⑤
**13.**	Microbiology laboratory results are efficiently communicated to the treating physician	①	②	③	④	⑤
**14.**	I regularly refer to/consider the antibiotic susceptibility patterns at this hospital/institution (i.e. the institutional antibiogram) when empirically prescribing antibiotics	①	②	③	④	⑤
**15.**	If medically appropriate, intravenous antibiotics should be stepped down to an oral alternative after three days	①	②	③	④	⑤
**16.**	Restrictions on antibiotics impair my ability to provide good patient care	①	②	③	④	⑤
**17.**	More judicious use of antibiotics would decrease antimicrobial resistance	①	②	③	④	⑤
**18.**	Following evidence-based antibiotic guidelines will help optimize treatment outcomes	①	②	③	④	⑤
**19.**	In general, rational antibiotic prescribing for my patients is high on my list of priorities	①	②	③	④	⑤
**20.**	Developing hospital antibiotic guidelines is more useful than applying international guidelines	①	②	③	④	⑤
**21.**	I am often unsure if a patient needs an antibiotic or not	①	②	③	④	⑤

For peer review only

**22.**	I am often unsure which antibiotic to prescribe	①	②	③	④	⑤
**23.**	I will stop antibiotics that others have prescribed in the absence of an appropriate indication	①	②	③	④	⑤
**24.**	Patients with high fever (≥39°C) must be treated with antibiotics	①	②	③	④	⑤
**25.**	If I am uncertain about the diagnosis of infection, but think it is possible, I feel safer prescribing an antibiotic	①	②	③	④	⑤
**26.**	Fear of patient deterioration or complications leads me to prescribe antibiotics more freely	①	②	③	④	⑤
**27.**	I frequently prescribe antibiotics because patients or their relatives insist on it	①	②	③	④	⑤
**ANTIMICROBIAL STEWARDSHIP PROGRAM (ASP)**<br>***A formal program formal that monitors and manages the appropiate use of antibiotics***						
**28.**	I am aware that my hospital has an antimicrobial stewardship program (ASP)	①	②	③	④	⑤
**29.**	I understand what the purpose of ASP is	①	②	③	④	⑤
**30.**	ASP improve patient care	①	②	③	④	⑤
**31.**	ASP reduces the problem of antimicrobial resistance	①	②	③	④	⑤
**32.**	ASP reduces this hospital's infection rates	①	②	③	④	⑤
**33.**	Additional staff education on antimicrobial prescribing is needed	①	②	③	④	⑤
**34.**	Regular audit and feedback encourage me to prescribe antibiotics prudently	①	②	③	④	⑤
**35.**	Rapid and accurate diagnostic tests are useful for diagnosis of infectious diseases and guidance on antibiotic therapy	①	②	③	④	⑤
**36.**	To reduce antibiotic overuse in hospitals, implementation of antibiotic restriction (e.g., antibiotic tiers) is a useful measure	①	②	③	④	⑤
**37.**	To curb antimicrobial resistance, regular consultations or ward rounds with a clinical microbiologist or infectious disease physician are useful	①	②	③	④	⑤
**38.**	To curb antimicrobial resistance, doctors need to have timely access to microbiological test results to guide antibiotic therapy	①	②	③	④	⑤
**39.**	Up-to-date information on hospital antimicrobial resistance patterns is important for developing hospital antibiotic guidelines	①	②	③	④	⑤
**40.**	Effective infection prevention and control in the hospital reduces antimicrobial resistance	①	②	③	④	⑤

BACKGROUND INFORMATION

41. What is your primary work unit in this hospital? **Tick ONE**

○ Many different unit/not specific<br>○ Emergency department<br>○ Surgery<br>○ Anaesthesiology<br>○ Intensive Care Unit (ALL)	○ OBGYN (Obstetrics/ Gynaecology)<br>○ Internal Medicine<br>○ Neurology<br>○ Paediatrics<br>○ Psychiatrics<br>○ ENT (Ear Nose Throat)	○ Eye<br>○ Dermato-venerology<br>○ Pulmonology<br>○ Cardiology<br>○ Orthopaedics<br>○ Radiology	○ Rehabilitation<br>○ Pharmacy<br>○ Microbiology<br>○ Laboratory<br>○ Other ______________

For peer review only

**42.**	How long have you worked in this hospital? **Tick ONE**
	○ Less than 1 year<br>○ 1 – 5 years<br>○ 6 – 10 years<br>○ 11 – 15 years<br>○ 16 - 20 years<br>○ More than 20 years
**43.**	What is your position in this hospital? **Tick ONE**
	○ Internship doctor<br>○ General Practitioner<br>○ Resident<br>○ Specialist<br>○ Other ____________________
**44.**	How long have you worked in your current specialty or profession? **Tick ONE**
	○ Less than 1 year<br>○ 1 – 5 years<br>○ 6 – 10 years<br>○ 11 – 15 years<br>○ 16 - 20 years<br>○ More than 20 years
**45.**	Which of the following resources do you use to guide your antibiotic prescribing? **Tick ALL that apply**
	○ Consultation with senior colleague(s)<br>○ Consultation with specialist in microbiology/ infectious disease<br>○ Textbooks<br>Guideline<br>☐ Internasional<br>☐ Nasional<br>☐ Hospital<br>☐ Departement/ division<br>○ Medical journal<br>○ Phamaceutical representative ______________________________<br>○ Internet ________________________<br>○ Other __________________________
**46.**	During the past year, how many times have you received training/teaching or attended seminars/courses on antimicrobial prescribing, resistance and/or stewardship? _____ times
**47.**	What is your sex: ○ Male ○ Female
**48.**	I would like to take part in the raffle: ○ yes ○ no If yes, my email is:___________________

Thank you for completing this survey!

For peer review only

Table S1 Survey response rate, overall and by hospital, department and professional hierarchy

	Total physicians invited	Total physicians responded	Response rate (%)	Respondents per physician type				
				Specialist/ Consultant	Resident	General practitioner	Intern/Internship doctor	Other
**Total**	**1896**	**1007**	**53.1%**	**358 (35.6%)**	**500 (49.7%)**	**113 (11.2%)**	**10 (1.0%)**	**18 (1.8%)**
**Hospital** [a]								
01	101 (5.3%)	70 (7.0%)	69.3%	39 (10.9%)	0 (0.0%)	27 (23.9%)	0 (0.0%)	4 (22.2%)
02	255 (13.4%)	155 (15.4%)	60.8%	70 (19.6%)	52 (10.4%)	26 (23.0%)	0 (0.0%)	5 (27.8%)
03	143 (7.5%)	77 (7.7%)	53.9%	41 (11.5%)	0 (0.0%)	35 (31.0%)	0 (0.0%)	1 (5.6%)
04	55 (2.9%)	44 (4.4%)	80.0%	29 (8.1%)	0 (0.0%)	11 (9.7%)	0 (0.0%)	1 (5.6%)
05	1240 (65.4%)	622 (61.8%)	50.2%	152 (42.5%)	448 (89.6%)	4 (3.5%)	10 (100.0%)	5 (27.8%)
06	102 (5.4%)	39 (3.9%)	38.2%	27 (7.5%)	0 (0.0%)	10 (8.9%)	0 (0.0%)	2 (11.1%)
**Sector** [b]								
Private	346	186 (18.5%)	53.8%	107 (29.9%)	0 (0.0%)	0 (0.0%)	72 (63.7%)	7 (38.9%)
Public	1550	821 (81.5%)	53.0%	251 (70.1%)	500 (100.0%)	41 (36.3%)	10 (100.0%)	11 (61.1%)
**Level of care** [c]								
Secondary	1499	777 (77.2%)	51.8%	136 (38.0%)	0 (0.0%)	83 (73.5%)	0 (0.0%)	8 (44.4%)
Tertiary	397	230 (22.8%)	57.9%	222 (62.0%)	500 (100.0%)	30 (26.6%)	10 (100.0%)	10 (55.6%)
**Department** [d]								
Surgery and surgical subspecialties	***628***	***371 (36.8)***	***59.1%***	***91 (24.5%)***	***226 (60.9%)***	***1 (0.27%)***	***4 (1.1%)***	***0 (0.0%)***
Obstetrics/gynaecology	229	146 (14.5%)	63.8%	51 (14.3%)	91 (18.2%)	1 (0.88%)	1 (10.0%)	0 (0.0%)
Surgery	210	122 (12.1%)	58.1%	2 (11.1%)	74 (14.8%)	0 (0.0%)	3 (30.0%)	0 (0.0%)
Orthopaedics	72	57 (5.7%)	44.4%	25 (7.0%)	32 (6.4%)	0 (0.0%)	0 (0.0%)	0 (0.0%)
Ear-nose-throat	74	32 (3.2%)	77.0%	12 (3.4%)	19 (3.8%)	0 (0.0%)	0 (0.0%)	0 (0.0%)
Urology	43	14 (1.4%)	32.6%	1 (1.1%)	10 (2.0%)	0 (0.0%)	0 (0.0%)	0 (0.0%)
Medicine and medical subspecialties	***456***	***232 (23.0%)***	***50.9%***	***115 (49.6%)***	***109 (47.0%)***	***0 (0.0%)***	***3 (1.3%)***	***5 (2.2%)***
Internal medicine	261	128 (12.7%)	49.0%	59 (16.5%)	67 (13.4%)	0 (0.0%)	0 (0.0%)	2 (11.1%)
Neurology	74	63 (6.3%)	85.1%	17 (4.8%)	40 (8.0%)	0 (0.0%)	3 (30.0%)	3 (16.7%)
Pulmonology	21	15 (1.5%)	71.4%	15 (4.2%)	0 (0.0%)	0 (0.0%)	0 (0.0%)	0 (0.0%)
Dermato-venerology	77	14 (1.4%)	18.2%	12 (0.0%)	2 (3.4%)	0 (0.0%)	0 (0.0%)	0 (0.0%)
Cardiology	23	12 (1.2%)	52.2%	12 (3.4%)	0 (0.0%)	0 (0.0%)	0 (0.0%)	0 (0.0%)
Acute specialties [e]	***96***	***156 (15.5)***	***162.5%***	***28 (17.9%)***	***67 (42.9%)***	***68 (43.6%)***	***2 (1.3%)***	***1 (0.64%)***
Anaesthesiology	96	72 (7.2%)	75.0%	4 (1.1%)	66 (13.2%)	0 (0.0%)	2 (20.0%)	0 (0.0%)

For peer review only - http://bmjopen.bmj.com/site/about/guidelines.xhtml

	Total physicians invited	Total physicians responded	Response rate (%)	Respondents per physician type				
				Specialist/ Consultant	Resident	General practitioner	Intern/Internship doctor	Other
Emergency dept [e]	-	57 (5.7%)	-	0 (0.0%)	0 (0.0%)	57 (50.4%)	0 (0.0%)	0 (0.0%)
Intensive Care Unit [e]	-	27 (2.7%)	-	14 (3.9%)	1 (0.2%)	11 (9.7%)	0 (0.0%)	1 (5.6%)
Other departments	***539***	***244 (24.2)***	***45.2%***	***90 (36.9%)***	***98 (40.2%)***	***24 (9.8%)***	***1 (0.41%)***	***10 (4.1%)***
Paediatrics	202	54 (5.4%)	26.7%	28 (7.8%)	25 (5.0%)	0 (0.0%)	0 (0.0%)	0 (0.0%)
Ophthalmology	102	39 (3.9%)	38.2%	22 (6.2%)	17 (3.4%)	0 (0.0%)	0 (0.0%)	0 (0.0%)
Multiple/unspecified units [e]	-	33 (3.3%)	-	5 (1.4%)	5 (1.0%)	21 (18.6%)	1 (10.0%)	0 (0.0%)
Rehabilitation	70	32 (3.2%)	45.7%	4 (1.1%)	28 (5.6%)	0 (0.0%)	0 (0.0%)	0 (0.0%)
Psychiatry	54	30 (3.0%)	55.6%	11 (3.1%)	19 (3.8%)	0 (0.0%)	0 (0.0%)	0 (0.0%)
Dentist	49	27 (2.7%)	55.1%	11 (3.1%)	1 (0.20%)	3 (2.7%)	0 (0.0%)	10 (55.6%)
Other	39	25 (2.5%)	64.1%	4 (1.1%)	1 (0.20%)	20 (17.7%)	0 (0.0%)	0 (0.0%)
Microbiology	4	3 (0.30%)	75.0%	2 (0.56%)	1 (0.20%)	0 (0.0%)	0 (0.0%)	0 (0.0%)
Laboratory	19	1 (0.10%)	5.3%	1 (0.28%)	0 (0.0%)	0 (0.0%)	0 (0.0%)	0 (0.0%)
Missing	-	4 (0.40%)	-	2 (0.56%)	1 (0.20%)	0 (0.0%)	0 (0.0%)	0 (0.0%)

Data are reported as n (%).
Doctor type was missing for 8 respondents, across [a] hospitals (02 (2), 03 (3), and 05 (3)); [b] sector (public 8, private 0); [c] level of care (secondary 3, tertiary 5); and [d] departments (obstetrics/gynaecology 2; ENT 1; paediatrics 1; multiple units 1; dental/oral surgery 2; missing dept 1).
[e] Invitations to participate in the survey were sent out according to primary work units; the discrepancies reported for the acute and other departments result from the fact that n=177 respondents who worked across multiple units (mostly GPs and internship doctors) indicated a different primary department on their questionnaire.

For peer review only

For peer review only - http://bmjopen.bmj.com/site/about/guidelines.xhtml

Table S2. Five-point Likert scale responses for the 40-item questionnaire

Item #	No of respondents	Strongly disagree (1)	Disagree (2)	Neutral (3)	Agree (4)	Strongly agree (5)
Q1	1005	3 (0.30)	26 (2.6)	168 (16.7)	452 (45.0)	356 (35.4)
Q2	1006	0 (0.0)	7 (0.70)	55 (5.5)	505 (50.2)	439 (43.6)
Q3	1003	0 (0.0)	6 (0.60)	43 (4.3)	472 (47.1)	482 (48.1)
Q4	1005	19 (1.9)	98 (9.8)	173 (17.2)	497 (49.5)	218 (21.7)
Q5	1004	2 (0.20)	49 (4.9)	145 (14.4)	514 (51.2)	294 (29.3)
Q6	1003	0 (0.0)	29 (2.9)	136 (13.6)	516 (51.5)	322 (32.1)
Q7	1004	12 (1.2)	53 (5.3)	288 (28.7)	460 (45.8)	191 (19.0)
Q8	1002	6 (0.60)	81 (8.1)	345 (34.4)	444 (44.3)	126 (12.6)
Q9	1005	71 (7.1)	397 (39.5)	311 (31.0)	188 (18.7)	38 (3.8)
Q10	1005	32 (3.2)	299 (29.8)	363 (36.1)	256 (25.5)	55 (5.5)
Q11	1005	8 (0.80)	34 (3.4)	102 (10.2)	506 (50.4)	355 (35.3)
Q12	1005	18 (1.8)	213 (21.2)	448 (44.5)	286 (28.5)	71 (7.1)
Q13	1005	11 (1.1)	97 (9.7)	321 (31.9)	515 (51.2)	61 (6.1)
Q14	1005	12 (1.2)	121 (12.0)	324 (32.2)	468 (46.6)	80 (8.0)
Q15	1006	7 (0.70)	73 (7.3)	195 (19.4)	623 (61.9)	108 (10.7)
Q16	1006	67 (6.7)	334 (33.2)	267 (26.5)	276 (27.4)	62 (6.2)
Q17	1005	2 (0.20)	2 (0.20)	48 (4.8)	431 (42.9)	522 (51.9)
Q18	1006	0 (0.0)	6 (0.60)	42 (4.2)	467 (46.4)	491 (48.8)
Q19	1005	1 (0.10)	7 (0.70)	105 (10.5)	545 (54.3)	347 (34.5)
Q20	1004	3 (0.30)	34 (3.4)	180 (17.9)	511 (50.9)	276 (27.5)
Q21	1006	60 (6.0)	440 (43.7)	269 (26.7)	204 (20.3)	33 (3.3)
Q22	1006	89 (8.9)	448 (44.5)	254 (25.3)	196 (19.5)	19 (1.9)
Q23	1002	19 (1.9)	124 (12.4)	288 (28.7)	464 (46.3)	107 (10.7)
Q24	1005	213 (21.2)	480 (47.8)	189 (18.8)	104 (10.4)	19 (1.9)
Q25	1005	51 (5.1)	285 (28.4)	301 (30.0)	348 (34.6)	20 (2.0)
Q26	1006	55 (5.5)	354 (35.2)	245 (24.4)	330 (32.8)	22 (2.2)
Q27	1005	231 (22.3)	538 (53.5)	138 (13.7)	90 (9.0)	8 (0.80)
Q28	1006	0 (0.0)	15 (1.5)	54 (5.4)	526 (52.3)	411 (40.9)
Q29	1006	2 (0.20)	6 (0.60)	71 (7.1)	585 (58.2)	342 (34.0)
Q30	1006	7 (0.70)	11 (1.1)	97 (9.6)	582 (57.9)	309 (30.7)
Q31	1005	2 (0.20)	7 (0.70)	109 (10.89)	620 (61.7)	267 (26.6)

For peer review only - http://bmjopen.bmj.com/site/about/guidelines.xhtml

Item #	No of respondents	Strongly disagree (1)	Disagree (2)	Neutral (3)	Agree (4)	Strongly agree (5)
Q32	1004	3 (0.30)	25 (2.5)	206 (20.5)	568 (56.6)	202 (20.1)
Q33	1005	2 (0.20)	14 (1.4)	101 (10.1)	574 (57.1)	314 (31.2)
Q34	1006	1 (0.10)	7 (0.70)	70 (7.0)	702 (69.8)	226 (22.5)
Q35	1006	0 (0.0)	4 (0.40)	31 (3.1)	646 (64.2)	325 (32.3)
Q36	1007	1 (0.10)	13 (1.3)	83 (8.2)	675 (67.0)	235 (23.3)
Q37	1006	2 (0.20)	17 (1.7)	128 (12.7)	657 (65.3)	202 (20.1)
Q38	1006	0 (0.0)	10 (0.99)	66 (6.6)	616 (61.2)	314 (31.2)
Q39	1006	0 (0.0)	4 (0.40)	49 (4.9)	617 (61.3)	336 (33.4)
Q40	1006	0 (0.0)	4 (0.40)	43 (4.3)	612 (60.8)	347 (34.5)

Data are also shown in Figure 1.

For peer review only

Table S3 The latent factors of antibiotic prescribing (full table)

Factor	Factor label	Survey questions included in the factor loading	Loadings	Reliability (Cronbach's α)
**1**	Awareness of AMS activities	Q33: Additional staff education on antimicrobial prescribing is needed	0.5202	0.8734
		Q34: Regular audit and feedback encourage me to prescribe antibiotics prudently	0.6151	
		Q35: Rapid and accurate diagnostic tests are useful for diagnosis of infectious diseases and guidance on antibiotic therapy	0.6714	
		Q36: To reduce antibiotic overuse in hospitals, implementation of antibiotic restriction (e.g. antibiotic tiers) is a useful measure	0.6428	
		Q37: To curb antimicrobial resistance, regular consultations or ward rounds with a clinical microbiologist or infectious disease physician are useful	0.7046	
		Q38: To curb antimicrobial resistance, doctors need to have timely access to microbiological test results to guide antibiotic therapy	0.7197	
		Q39: Up-to-date information on hospital antimicrobial resistance patterns is important for developing hospital antibiotic guidelines	0.7374	
		Q40: Effective infection prevention and control in the hospital reduces antimicrobial resistance	0.7067	
**2**	Awareness of AMS purposes	Q28: I am aware that my hospital has an antimicrobial stewardship program (ASP)	0.6224	0.8334
		Q29: I understand what the purpose of ASP is	0.6957	
		Q30: ASP improve patient care	0.7744	
		Q31: ASP reduces the problem of antimicrobial resistance	0.7532	
		Q32: ASP reduces this hospital's infection rates	0.6670	
**3**	Views regarding rational antibiotic prescribing	Q11: Antibiotics are overused in Indonesia	0.5138	0.6961
		Q17: More judicious use of antibiotics would decrease antimicrobial resistance	0.7362	
		Q18: Following evidence-based antibiotic guidelines will help optimize treatment outcomes	0.6565	
		Q19: In general, rational antibiotic prescribing for my patients is high on my list of priorities	0.5246	
		Q20: Developing hospital antibiotic guidelines is more useful than applying international guidelines	0.3499	
**4**	Confidence in antibiotic prescribing decisions	Q12: Antibiotics are overused in this hospital	-0.4550	0.6997
		Q16: Restrictions on antibiotics impair my ability to provide good patient care	0.4031	
		Q21: I am often unsure if a patient needs an antibiotic or not	0.5640	
		Q22: I am often unsure which antibiotic to prescribe	0.5670	
		Q24: Patients with high fever (≥39°C) must be treated with antibiotics	0.4794	

For peer review only - http://bmjopen.bmj.com/site/about/guidelines.xhtml

Factor	Factor label	Survey questions included in the factor loading	Loadings	Reliability (Cronbach's α)
		Q25: If I am uncertain about the diagnosis of infection, but think it is possible, I feel safer prescribing an antibiotic	0.6741	
		Q26: Fear of patient deterioration or complications leads me to prescribe antibiotics more freely	0.7092	
		Q27: I frequently prescribe antibiotics because patients or their relatives insist on it	0.6318	
**5**	Perception of AMR as a significant problem	Q1: Antimicrobial resistance is a significant problem in this hospital	0.5701	0.6967
		Q2: Antimicrobial resistance is a significant problem in Indonesia	0.5742	
		Q3: A cause of antimicrobial resistance is using too many antimicrobial drugs	0.5361	
		Q4: Lack of hand disinfection by healthcare workers causes spread of antimicrobial resistance	0.5725	
		Q5: Use of broad-spectrum antibiotics can increase antimicrobial resistance when narrower-spectrum antibiotics are available that are equally effective	0.5480	
		Q6: Antibiotic resistance is also a problem outside of the hospital, in communities	0.4766	
**6**	Immediate actions to contain AMR	Q7: In this hospital, patient rooms are cleaned according to hospital cleaning protocol once a patient with a multidrug-resistant organism (MDRO) has been discharged	0.6058	0.5695
		Q8: Adherence to hand-hygiene protocols is excellent at this hospital	0.6368	
		Q13: Microbiology laboratory results are efficiently communicated to the treating physician	0.5116	
		Q14: I regularly refer to/consider the antibiotic susceptibility patterns at this hospital/institution (i.e. the institutional antibiogram) when empirically prescribing antibiotics	0.6115	
		Q15: If medically appropriate, intravenous antibiotics should be stepped down to an oral alternative after three days	0.3617	
		Q23: I will stop antibiotics that others have prescribed in the absence of an appropriate indication	0.3892	

Abbreviations: AMS, antibiotic stewardship; ASP, antibiotic stewardship programme; IPC, infection prevention and control
Data are summarised in Table 3
Item #9 and 10 were excluded from the analysis, as explained in Results.

For peer review only - http://bmjopen.bmj.com/site/about/guidelines.xhtml

Figure S1 Scree plot showing eigenvalues for the 40 factors

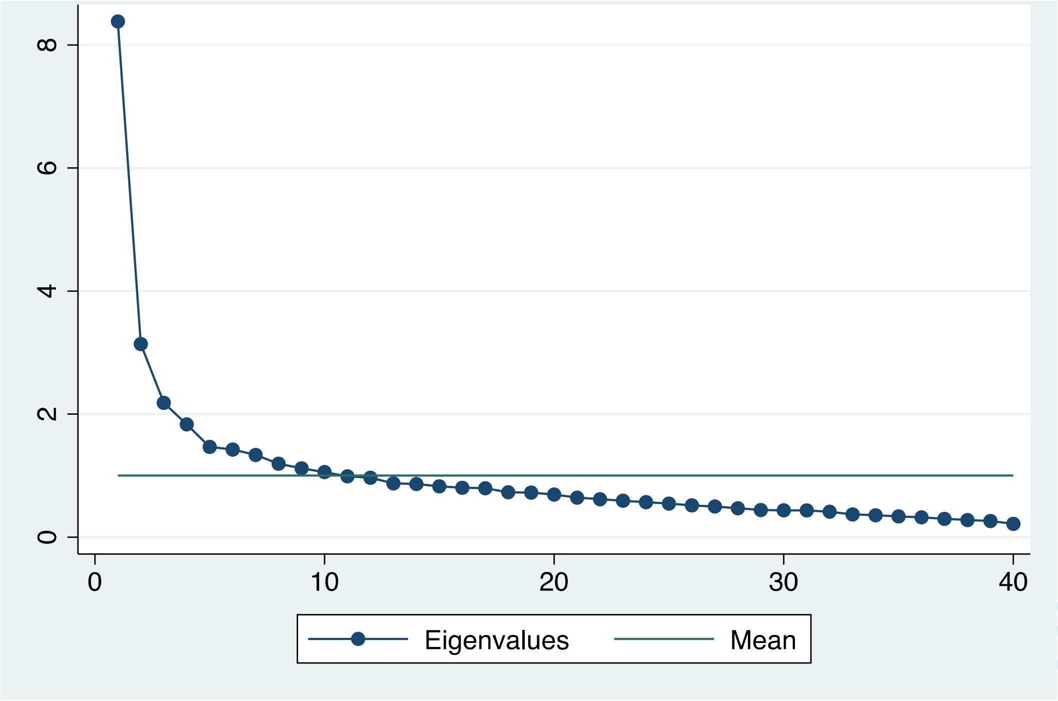

ew only

For peer review only - http://bmjopen.bmj.com/site/about/guidelines.xhtml

Figure S2 Parallel analysis showing adjusted eigenvalues for the 40 factors.

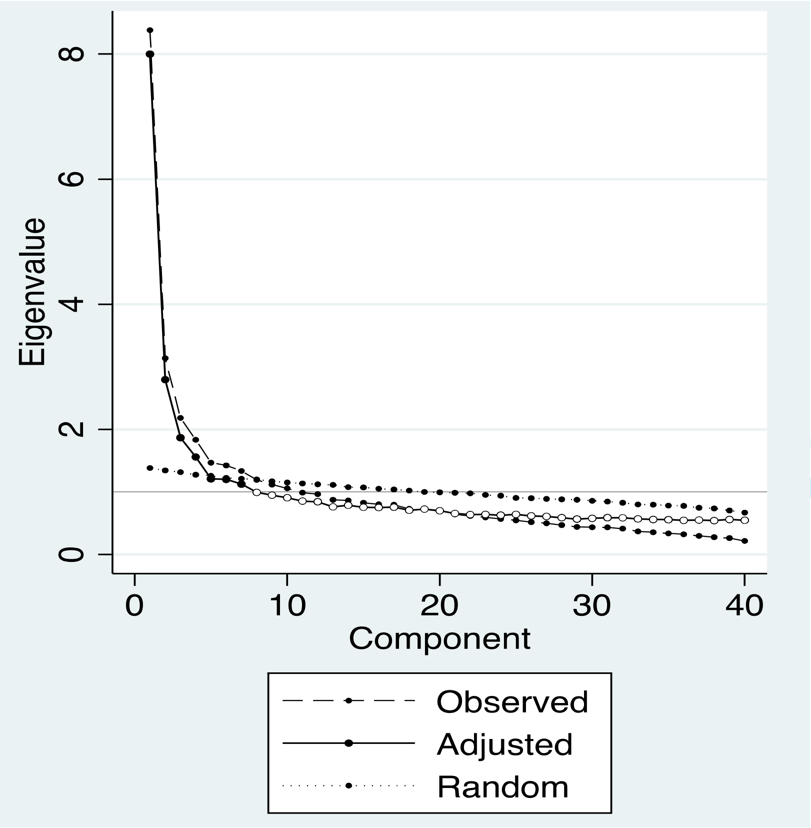

Parallel analysis adjusted the original eigenvalues for sampling error-induced collinearity among the variables to arrive at the adjusted eigenvalues.

STROBE Statement—

Checklist of items that should be included in reports of ***cross-sectional studies***

	Item No	Recommendation	Page No
**Title and abstract**	1	(*a*) Indicate the study's design with a commonly used term in the title or the abstract	1
		(*b*) Provide in the abstract an informative and balanced summary of what was done and what was found	2
**Introduction**			
Background/rationale	2	Explain the scientific background and rationale for the investigation being reported	4
Objectives	3	State specific objectives, including any prespecified hypotheses	5
**Methods**			
Study design	4	Present key elements of study design early in the paper	5
Setting	5	Describe the setting, locations, and relevant dates, including periods of recruitment, exposure, follow-up, and data collection	5-6
Participants	6	(*a*) Give the eligibility criteria, and the sources and methods of selection of participants	5-6
Variables	7	Clearly define all outcomes, exposures, predictors, potential confounders, and effect modifiers. Give diagnostic criteria, if applicable	6
Data sources/ measurement	8*	For each variable of interest, give sources of data and details of methods of assessment (measurement). Describe comparability of assessment methods if there is more than one group	6
Bias	9	Describe any efforts to address potential sources of bias	6
Study size	10	Explain how the study size was arrived at	7
Quantitative variables	11	Explain how quantitative variables were handled in the analyses. If applicable, describe which groupings were chosen and why	7
Statistical methods	12	(*a*) Describe all statistical methods, including those used to control for confounding	7
		(*b*) Describe any methods used to examine subgroups and interactions	7
		(*c*) Explain how missing data were addressed	7
		(*d*) If applicable, describe analytical methods taking account of sampling strategy	7
		(*e*) Describe any sensitivity analyses	NA
**Results**			
Participants	13*	(a) Report numbers of individuals at each stage of study—eg numbers potentially eligible, examined for eligibility, confirmed eligible, included in the study, completing follow-up, and analysed	8
		(b) Give reasons for non-participation at each stage	8
		(c) Consider use of a flow diagram	NA

For peer review only

Descriptive data	14*	(a) Give characteristics of study participants (eg demographic, clinical, social) and information on exposures and potential confounders	8
		(b) Indicate number of participants with missing data for each variable of interest	8-9
Outcome data	15*	Report numbers of outcome events or summary measures	8-10
Main results	16	(*a*) Give unadjusted estimates and, if applicable, confounder-adjusted estimates and their precision (eg, 95% confidence interval). Make clear which confounders were adjusted for and why they were included	8-10
		(*b*) Report category boundaries when continuous variables were categorized	8-10
		(*c*) If relevant, consider translating estimates of relative risk into absolute risk for a meaningful time period	NA
Other analyses	17	Report other analyses done—eg analyses of subgroups and interactions, and sensitivity analyses	10
**Discussion**			
Key results	18	Summarise key results with reference to study objectives	11
Limitations	19	Discuss limitations of the study, taking into account sources of potential bias or imprecision. Discuss both direction and magnitude of any potential bias	13
Interpretation	20	Give a cautious overall interpretation of results considering objectives, limitations, multiplicity of analyses, results from similar studies, and other relevant evidence	11-12
Generalisability	21	Discuss the generalisability (external validity) of the study results	11-12
**Other information**			
Funding	22	Give the source of funding and the role of the funders for the present study and, if applicable, for the original study on which the present article is based	14

*Give information separately for exposed and unexposed groups.

Note: An Explanation and Elaboration article discusses each checklist item and gives methodological background and published examples of transparent reporting. The STROBE checklist is best used in conjunction with this article (freely available on the Web sites of PLoS Medicine at http://www.plosmedicine.org/, Annals of Internal Medicine at http://www.annals.org/, and Epidemiology at http://www.epidem.com/). Information on the STROBE Initiative is available at www.strobe-statement.org.

For peer review only
