# Supplementary material for: Perceptions, views and practices regarding antibiotic prescribing and stewardship among hospital physicians in Jakarta, Indonesia: a questionnaire-based survey
Source: BMJ Open. 2022 May 18;12(5):e054768. doi: 10.1136/bmjopen-2021-054768 (PMC9121411; doi:10.1136/bmjopen-2021-054768)
Supplement: Reviewer comments [file bmjopen-2021-054768.reviewer_comments.pdf]

PEER REVIEW HISTORY

BMJ Open publishes all reviews undertaken for accepted manuscripts. Reviewers are asked to complete a checklist review form (**http://bmjopen.bmj.com/site/about/resources/checklist.pdf**) and are provided with free text boxes to elaborate on their assessment. These free text comments are reproduced below.

ARTICLE DETAILS

**TITLE (PROVISIONAL)**	Perceptions, views and practices regarding antibiotic prescribing and stewardship among hospital physicians in Jakarta, Indonesia: a questionnaire-based survey
**AUTHORS**	Limato, Ralalicia; Nelwan, Erni Juwita; Mudia, Manzilina; Alamanda, Monik; Manurung, Elfrida Rinawaty; Mauleti, Ifael Yerosias; Mayasari, Maria; Firmansyah, Iman; Djaafar, Roswin; Vu, Huong; van Doorn, H. Rogier; Broom, Alex; Hamers, Raph Leonardus

VERSION 1 – REVIEW

**REVIEWER**	Liu, Chenxi<br>Huazhong University of Science and Technology Tongji Medical College, School of medicine and health management
**REVIEW RETURNED**	25-Sep-2021

**GENERAL COMMENTS**	Page 3 Line 6. The study aims to explore the perception and views of antibiotic prescribing behavior and AMS, actually no practice concerning AMR was explored in this study.<br><br>Introduction Section<br>The introduction part is concise and clearly defined.<br>Page 5 Line 45-47 Maybe more detailed statistical information of threats of AMR and AMS in Indonesia is provided, such as how many AMR programs launched? In hospital or in communities?<br>Page 5 Line 57 What is the meaning of accounts of antibiotic prescribing?<br>Page5-6 Line59 Line 1~Line2 The last sentence in the Introduction part is suggested to move into the Methods Section.<br><br>Methods section<br>Page 6 Line 10 What is the sampling method? In addition, maybe more detailed information of the participating facilities, for example, size or location of the participating facilities? And what is the main difference of private and public sector, especially the key indicator related with this study, the departments and professional hierarchy?<br>Page 6 Line 26~Line 28 Please provide more information about the clinical departments, the residents or internship was professional titles for specialists? GPs and specialists for departments?<br><br>Page 6 Line 32~Line 34, Page 7 Line 34~Line 37 The ethics part was duplicates in this part.<br>Page 6 Line 51 What was the conceptual framework was based to develop the questionnaire?<br>Page 7 Line 7 Adjustments were made in accordance to the feedback? What kind of adjustments?

	Page 7 Line 21~31 How can the researchers guarantee the quality of the questionnaire, for example, if it is possible that physicians helped each other to fill the questionnaire? If there is any way to guarantee participating physicians completed the survey independently<br>Line 35 Immediate actions to contain AMR? Or control AMR?<br><br>Results section<br>Page 9 Line 21 What did you mean “lack a theoretical basis for one factor”? How did this research define theoretical basis?<br>Page 11 Line 9 Subgroup analysis How was the mixed effects linear regression (in Methods statistical analysis) was conducted?<br><br>Discussion<br>Page 12 Line 8-Line 12. It was part of the Results section. Please remove it from Discussion part.<br>Page 12 Line 19 “In turn, illustrate a specific mix of variables at play” This sentence was hard to understand.<br>The discussion of potential reasons of the differences of the hospitals should be added.<br><br>Herawati, Fauna<br>University of Surabaya, Department of Clinical and Community Pharmacy, Faculty of Pharmacy<br><br>03-Dec-2021<br><br>This is an extensive and important study. There are some considerations to improve the manuscript.<br><br>Methods: What is the difference between perspective and view in this study? With the questionnaire, the researchers measure the self-perception for their practice, not their action or behavior practice. How author differentiate item questions for every domain (antimicrobial resistance, antibiotic prescribing practices, antimicrobial stewardship program)?<br><br>Statistical analysis<br>Antibiotic prescribing practices in public and private hospitals are different because of their governance (Mahendradhata, 2017). If we look at Table S1, there is a non-respondent bias. The response rate is <10% except for hospital 5. Moreover, the percentage of respondent consultants, residents, GP, and intern/internship doctors in public and private sectors differ. This characteristic will affect results, particularly confidence in prescribing decisions, uncertain about the diagnosis of infection. The researcher should consider this aspect when analyzing the data. In Table 4 Physician subgroup analysis, the researcher only performs statistical analysis for the public sector and tertiary care.<br><br>Note: Intern/internship doctor is graduated from medical education who is currently studying to become a doctor. Resident is a doctor who is undergoing education to become a specialist. Private sector consist of consultant and intern/internship doctor only.<br><br><br>There is discrepancy between Table 1 and S1. Tabel S1: Consultant: 358, resident: 500, GP: 41, intern/internship doctor: 82. Table 1: Consultant: 358, resident: 500, GP: 113, intern/internship doctor: 10.

	Table 1<br><br>Reference:<br>Mahendradhata Y, Trisnantoro L, Listyadewi S, Soewondo P, Marthias T. et al. (2017). The Republic of Indonesia Health System Review, Health Systems in Transition, Vol-7 No.1. WHO Regional Office for South-East Asia.<br>https://apps.who.int/iris/handle/10665/254716.<br><br>Discussion<br>On page 7, lines 50-56. The authors explain questionnaire development.<br>Behavioural theories, good practice recommendations for questionnaire design and existing questionnaires in the literature were reviewed and discussed with several experts<br><br>This behavioral theory should use to explain the phenomena of the finding.<br>Whereas most respondents (85.7%; 861/1005) agreed that antibiotics are overused in Indonesia (Q11), only 35.5% (357/1005) acknowledged this to be case at their hospital [Q12]. Most agreed that more judicious antimicrobial prescribing practices would decrease AMR (94.8%; 953/1005) [Q17] and that following evidence-based antibiotic guidelines will help optimize treatment outcomes (95.3%; 958/1006) [Q18]. Most gave high priority to rational antibiotic prescribing to their patients (88.8%; 892/1005) [Q19], and considered developing hospital antibiotic guidelines more useful than applying international guidelines (78.4%; 787/1004) [Q20].<br>The respondent is the perceived “externality of AMR” as a problem29. That is, physicians acknowledge its significance but do not take ownership or responsibility, thus reflecting a production of AMR as an externality, e.g. a result of irrational use elsewhere in communities or other hospitals.<br><br>On page 13, lines 30-33. The authors hadn’t given clear priority recommendations for what sectors or medical hierarchy.

**REVIEWER**	Herawati, Fauna<br>University of Surabaya, Department of Clinical and Community Pharmacy, Faculty of Pharmacy
**REVIEW RETURNED**	03-Dec-2021

**GENERAL COMMENTS**	This is an extensive and important study. There are some considerations to improve the manuscript.<br><br>Methods: What is the difference between perspective and view in this study? With the questionnaire, the researchers measure the self-perception for their practice, not their action or behavior practice. How author differentiate item questions for every domain (antimicrobial resistance, antibiotic prescribing practices, antimicrobial stewardship program)?<br><br>Statistical analysis<br>Antibiotic prescribing practices in public and private hospitals are different because of their governance (Mahendradhata, 2017). If we look at Table S1, there is a non-respondent bias. The response rate is <10% except for hospital 5. Moreover, the percentage of

	respondent consultants, residents, GP, and intern/internship doctors in public and private sectors differ. This characteristic will affect results, particularly confidence in prescribing decisions, uncertain about the diagnosis of infection. The researcher should consider this aspect when analyzing the data. In Table 4 Physician subgroup analysis, the researcher only performs statistical analysis for the public sector and tertiary care.<br><br>Note: Intern/internship doctor is graduated from medical education who is currently studying to become a doctor. Resident is a doctor who is undergoing education to become a specialist. Private sector consist of consultant and intern/internship doctor only.<br><br>There is discrepancy between Table 1 and S1. Tabel S1: Consultant: 358, resident: 500, GP: 41, intern/internship doctor: 82. Table 1: Consultant: 358, resident: 500, GP: 113, intern/internship doctor: 10.<br>Table 1<br><br>Reference:<br>Mahendradhata Y, Trisnantoro L, Listyadewi S, Soewondo P, Marthias T. et al. (2017). The Republic of Indonesia Health System Review, Health Systems in Transition, Vol-7 No.1. WHO Regional Office for South-East Asia.<br>https://apps.who.int/iris/handle/10665/254716.<br><br>Discussion<br>On page 7, lines 50-56. The authors explain questionnaire development.<br>Behavioural theories, good practice recommendations for questionnaire design and existing questionnaires in the literature were reviewed and discussed with several experts<br><br>This behavioral theory should use to explain the phenomena of the finding.<br>Whereas most respondents (85.7%; 861/1005) agreed that antibiotics are overused in Indonesia (Q11), only 35.5% (357/1005) acknowledged this to be case at their hospital [Q12]. Most agreed that more judicious antimicrobial prescribing practices would decrease AMR (94.8%; 953/1005) [Q17] and that following evidence-based antibiotic guidelines will help optimize treatment outcomes (95.3%; 958/1006) [Q18]. Most gave high priority to rational antibiotic prescribing to their patients (88.8%; 892/1005) [Q19], and considered developing hospital antibiotic guidelines more useful than applying international guidelines (78.4%; 787/1004) [Q20].<br>The respondent is the perceived “externality of AMR” as a problem29. That is, physicians acknowledge its significance but do not take ownership or responsibility, thus reflecting a production of AMR as an externality, e.g. a result of irrational use elsewhere in communities or other hospitals.<br><br>On page 13, lines 30-33. The authors hadn’t given clear priority recommendations for what sectors or medical hierarchy.

VERSION 1 – AUTHOR RESPONSE

Reviewer #1 (Dr. Chenxi Liu):

Abstract

Reviewer comment #1:
Page 3 Line 6. The study aims to explore the perception and views of antibiotic prescribing behavior and AMS, actually no practice concerning AMR was explored in this study.
Author response #1:
We revised the sentence.

Introduction

Reviewer comment #2
Page 5 Line 45-47 Maybe more detailed statistical information of threats of AMR and AMS in Indonesia is provided, such as how many AMR programs launched? In hospital or in communities?
Author response #2:
We provided further details on the national response to AMR in Indonesia.

Reviewer comment #3
Page 5 Line 57 What is the meaning of accounts of antibiotic prescribing?
Author response #3:
We clarified the statement.

Reviewer comment #4:
Page5-6 Line59 Line 1~Line2 The last sentence in the Introduction part is suggested to move into the Methods Section.
Author response #4:
We revised this sentence to only describe the study's objectives.

Methods

Reviewer comment #5:
Page 6 Line 10 What is the sampling method? In addition, maybe more detailed information of the participating facilities, for example, size or location of the participating facilities? And what is the main difference of private and public sector, especially the key indicator related with this study, the departments and professional hierarchy?
Author response #5:
We have provided additional information related to the hospital characteristics and sampling strategies for hospitals and respondents. An analysis of differences between private and public hospitals is included in the analysis.

Reviewer comment #6:
Page 6 Line 26~Line 28 Please provide more information about the clinical departments, the residents or internship was professional titles for specialists? GPs and specialists for departments?
Author response #6:
We added additional details in the methods section (study respondents):

Reviewer comment #7:
Page 6 Line 32~Line 34, Page 7 Line 34~Line 37 The ethics part was duplicates in this part.
Author response #7:
We have removed the duplicate.

Reviewer comment #8:
Page 6 Line 51 What was the conceptual framework was based to develop the questionnaire?
Author response #8:
We pragmatically worked with a conceptual framework that included attributes related to prescribers' perceptions, views and practices, based on existing questionnaires in the literature and specific input from several experts with clinical experience in Indonesia and the region. We did not use any specific or named behavioural theory as our guide, and have removed this to avoid any confusion.

Reviewer comment #9:
Page 7 Line 7 Adjustments were made in accordance to the feedback? What kind of adjustments
Author response #9:
We have clarified the statement.

Reviewer comment #10:
Page 7 Line 21~31 How can the researchers guarantee the quality of the questionnaire, for example, if it is possible that physicians helped each other to fill the questionnaire? If there is any way to guarantee participating physicians completed the survey independently?
Author response #10:
The reviewer raises a good point. We acknowledge that we cannot 100% guarantee the complete independence of all respondents. However, we are quite certain that any exchange of information or collaboration did not occur, or if so, only at a very limited scale. Mainly, we restricted the possibility of respondents helping each other by, where ever possible, distributing the questionnaires and having them completed in real-time during department meetings, under the supervision of the study coordinator and department supervisors. Further, during data analysis we did not identify any unusual data patterns suggestive of shared questionnaire completion.

Reviewer comment #11:
Line 35 Immediate actions to contain AMR? Or control AMR?
Author response #11:
To contain AMR.

Results

Reviewer comment #12:
Page 9 Line 21 What did you mean "lack a theoretical basis for one factor"? How did this research define theoretical basis?
Author response #12:
We did not use a strict definition for theoretical basis. In this case, the four-factor solution brought together items in one factor mixing "Perception of AMR as a significant problem" (Q1-6) with "Views

regarding rational antibiotic prescribing" (Q11, 17-20), which were considered two separate constructs and were appropriately categorised as such in the preferred 6-factor solution.

Reviewer comment #13:
Page 11 Line 9 Subgroup analysis How was the mixed effects linear regression (in Methods statistical analysis) was conducted?
Author response #13:
Using the factor scores from the EFA as the dependent variables, we developed 6 regression models (one for each factor score). Physician characteristics ("subgroups") were the independent variables i.e., hospital sector and care level, grouped departments, work experience and medical hierarchy. We then used multivariable linear regression to analyze the associations between each of the factor scores and the physician characteristics. Independent variables were selected for multivariable model based on p<0.10 using a step-forward process. Given the hierarchical structure of the data (respondents in hospitals) we applied multilevel modelling to control for possible clustering within hospitals. P-values<0.05 were considered statistically significant. All analyses were done with Stata/IC Version 16.1 (StataCorp, College Station, TX, US). We added some additional details on the regression analysis in the Methods section.

Discussion

Reviewer comment #14:
Page 12 Line 8-Line 12. It was part of the Results section. Please remove it from Discussion part
Author response #14:
At the start of the Discussion, we have included a summary of key findings to set the stage for further discussion and interpretation of the study findings.

Reviewer comment #15:
Page 12 Line 19 "In turn, illustrate a specific mix of variables at play" This sentence was hard to understand.
Author response #15:
We reworded the sentence.

Reviewer comment #16
The discussion of potential reasons of the differences of the hospitals should be added.
Author response #16:
We included a brief description in the Discussion to highlight that no differences were observed between public/private or secondary/tertiary hospitals.

Reviewer #2 (Dr. Fauna Herawati):

Methods

Reviewer comment #1:
What is the difference between perspective and view in this study? With the questionnaire, the researchers measure the self-perception for their practice, not their action or behavior practice.
Author response #1:
The difference between views and perception in this study refers to the different meaning between these two terms.
Views refers to the way to regard something, usually based on the individual's attitude. For example: [Q17] More judicious use of antibiotics would decrease antimicrobial resistance.

Perception refers to interpretation/understanding about certain situation/people based on sensory experience, including past experience. For example, [Q6] Antibiotic resistance is also a problem outside of the hospital, in communities.
Related to antibiotic prescribing practice, the questionnaire assessed prescribers' reported practice/actions related to antibiotic use. For example, [Q14] I regularly refer to/consider the antibiotic susceptibility patterns at this hospital/institution (i.e., the institutional antibiogram) when empirically prescribing antibiotics.

Reviewer comment #2:
How author differentiate item questions for every domain (antimicrobial resistance, antibiotic prescribing practices, antimicrobial stewardship program)?
Author response #2:
As described in the Methods section (under survey questionnaire), we developed the questionnaire and the initial list of questions under each respective heading, based on the questionnaire from The Clinician Pre/Post Perception Survey of the Greater New York Hospital Association United Hospital Fund (reference no.19), and we then supplemented those questions with relevant questionnaire items identified from peer-reviewed publications (reference no. 20-26).

Results

Reviewer comment #3:
Antibiotic prescribing practices in public and private hospitals are different because of their governance (Mahendradhata, 2017). If we look at Table S1, there is a non-respondent bias. The response rate is <10% except for hospital 5. Moreover, the percentage of respondent consultants, residents, GP, and intern/internship doctors in public and private sectors differ. This characteristic will affect results, particularly confidence in prescribing decisions, uncertain about the diagnosis of infection. The researcher should consider this aspect when analysing the data.
Reference:
Mahendradhata Y, Trisnantoro L, Listyadewi S, Soewondo P, Marthias T. et al. (2017). The Republic of Indonesia Health System Review, Health Systems in Transition, Vol-7 No.1. WHO Regional Office for South-East Asia. https://apps.who.int/iris/handle/10665/254716.
Author response #3:
We thank the Reviewer for their thoughtful comments.

Table S1 column "Response rate (%)" provides the response rates for each hospital (and other subgroups), showing that the response rate was 53.1% overall and >50% for each hospital, with the exception of hospital 6 (38.2%). The Reviewer is correct in noting the differences in numbers and distributions of physician types across the hospitals. Those variations are a reflection of the clinical reality in those hospitals, that is, each hospital has a specific staff profile based on a range of factors, including the case-mix of the communities they serve and the organisational structure. In the study limitations we have acknowledged that non-participation and the convenient hospital sample could have introduced selection bias, and the data are not necessarily representative for Jakarta or Indonesia at large.

We agree with the Reviewer, that antibiotic prescribing differ between public and private hospitals in Indonesia. However, based on this study, we found no statistically significant differences between hospital sector (public versus private) or health care level (secondary versus tertiary) hospitals with regards to perceptions and views on AMR and AMS.
We did identify differences between physician subgroups based on work experience, professional hierarchy and department as presented in the Results section.

Reviewer comment #4:

In Table 4 Physician subgroup analysis, the researcher only performs statistical analysis for the public sector and tertiary care.
Author response #4:
As shown in Table 4, the regression model includes the following independent variables: hospitals, departments, professional hierarchy, years in current profession, health care sector and health care level. The findings are further described in the Results section, under Physician subgroup analysis.

Reviewer comment #5:
Note: Intern/internship doctor is graduated from medical education who is currently studying to become a doctor. Resident is a doctor who is undergoing education to become a specialist. Private sector consist of consultant and intern/internship doctor only.
Author response #5:
We added definitions of the doctor types to the methods section (study respondents)
Of note, residents only practiced in two public hospitals (hospital 2 and 5), and there were 10 interns in one public hospital (hospital 5) (Table S1).

Reviewer comment #6:
There is discrepancy between Table 1 and S1. Tabel S1: Consultant: 358, resident: 500, GP: 41, intern/internship doctor: 82. Table 1: Consultant: 358, resident: 500, GP: 113, intern/internship doctor: 10.
Author response #6:
We have double-checked both tables and we found no discrepancy between Table 1 and Table S1. The total participated GPs were 113 respondents, and 41 of them were from public hospitals (Table S1).

Discussion

Reviewer comment #7:
On page 7, lines 50-56. The authors explain questionnaire development.
Behavioural theories, good practice recommendations for questionnaire design and existing questionnaires in the literature were reviewed and discussed with several experts. This behavioral theory should use to explain the phenomena of the finding.
Whereas most respondents (85.7%; 861/1005) agreed that antibiotics are overused in Indonesia (Q11), only 35.5% (357/1005) acknowledged this to be case at their hospital [Q12]. Most agreed that more judicious antimicrobial prescribing practices would decrease AMR (94.8%; 953/1005) [Q17] and that following evidence-based antibiotic guidelines will help optimize treatment outcomes (95.3%; 958/1006) [Q18]. Most gave high priority to rational antibiotic prescribing to their patients (88.8%; 892/1005) [Q19], and considered developing hospital antibiotic guidelines more useful than applying international guidelines (78.4%; 787/1004) [Q20].
The respondent is the perceived “externality of AMR” as a problem29. That is, physicians acknowledge its significance but do not take ownership or responsibility, thus reflecting a production of AMR as an externality, e.g. a result of irrational use elsewhere in communities or other hospitals.
Author response #7:
Regarding the conceptual framework and behaviour theory, please refer to Reviewer #1 Author response #8. If there additional questions, we are happy to address those after clarification by the Reviewer.

Reviewer comment #8:
On page 13, lines 30-33. The authors hadn’t given clear priority recommendations for what sectors or medical hierarchy.
Author response #8:

The first paragraph of page 13 interprets findings related to professional hierarchy and the second paragraph discusses differences between departments/specialties. This study improves our understanding of the issues related to context-specific perceptions regarding antibiotic prescribing practice and AMS implementation among physicians in Jakarta, and hence helps identify opportunities of interventions. However, the study findings, at this stage, cannot be directly translated into firm recommendations for practice. Further contextual research is needed to design and test locally tailored interventions that work.

Point by point response to editorial requests

Editorial request #1:
Please include the study design in the Title.
Author response #1:
We revised the title as requested.

Formatting Amendments

Editorial request #2:
Required Supplementary format: Please re-upload your Supplementary files in PDF format
Author response #2:
We re-up-loaded the Supplementary files in PDF format as requested.

Editorial request #3:
Corresponding author's institution is not validated by Ringgold.
We have noticed that the corresponding author's institution is not validated by Ringgold, we suggest the below steps:
· Try a few alternate spellings of their institution.
· Try the full name of the institution.
· Is there a Sub-name within the overall name? (For instance, some universities will have named departments like University of Virginia’s Darden School of Business)
· Still not able to find your institution? Contact Ringgold directly to double-check the institution is included in the Ringgold Database at https://support.ringgold.com/.
Author response #3:
We followed your suggestions, and now the corresponding author’s institutions are validated by Ringgold.

Editorial request #4:
Author's name mismatch. The author “Djafar, Roswin” in your main document is registered as “Djaafar, Roswin” in ScholarOne. Please ensure that the author has the same registered name.
Author response #4:
We have corrected the author’s name. The correct name is Djaafar, Roswin as written in ScholarOne.

VERSION 2 – REVIEW

**REVIEWER**	Liu, Chenxi<br>Huazhong University of Science and Technology Tongji Medical College, School of medicine and health management
**REVIEW RETURNED**	17-Jan-2022

GENERAL COMMENTS	Thanks very much for the opportunity to review this manuscript. The study aimed to understand the perceptions of antimicrobial resistance (AMR), antibiotic prescribing practice, and views on antimicrobial stewardship (AMS) among hospital physicians in Jakarta, Indonesia. A cross-sectional survey, with exploratory factor analysis (EFA) and multivariable linear regression, was adopted. The result is interesting and, to an extent, can help inform the interventions of AMS in Indonesia. However, there are some main concerns raised as follows:<br>1. Background:<br>The study was conducted in hospitals, while the overuse of antibiotics was mainly occurred in primary care according to existing studies. I recommended that authors may need to add a brief summary of the role of hospitals in the overall AMR and overuse of antibiotics in Indonesia, why the current study focused on hospitals. This information may help readers understand the significance of the current study and contextualize the results into the overall context of Indonesia.<br>2. Methods-Study design and setting<br>Authors may need to present the inclusion and exclusion criteria of selected hospitals in the current study. How many hospitals there in Jakarta and how these 6 hospitals were selected? Why the six hospitals were selected and to what extent that the 6 hospitals were able to represent the status quo in Jakarta (for example, the 6 hospitals provide 80% outpatients/inpatients services in this area)?<br>3. Methods- Study respondents<br>The authors stated a purposive sampling in the current study. However, it seemed that the process of sampling described in respondent recruitment was a full sample within each hospital (all eligible doctors were approached)? Thus, I'm confused about the sampling process. Did the authors adopt a two-stage sampling method with purposive sampling on department-level and a full sample within each department? If so, authors may need to present how the purposive sampling was able to cover "all departments, work experience, and professional hierarchy, to ensure representation of different antibiotic practice and views, perceptions, experiences related to AMR and AMS". To my knowledge, it seemed that random sampling methods were more likely to generate such a sample. Was there any reason considered within the sampling process for purposive sampling? Pls, add such information.<br>4. Methods- Study respondents<br>What are the others? Did they represent the other departments in table S1? Are they doctors and able to prescribe antibiotics in Indonesia? (For example, Laboratory, Microbiology) If not, they seemed are not able to be included in the current study?<br>5. Methods- Statistical analysis<br>5.1 Authors conducted the screen plot and parallel analysis to determine the numbers of eigenvalues. But they were missed in the statistical analysis. Pls, add a description of these methods and processes.<br>5.2 Authors may need to add information regarding how they address insufficient loading (criteria for item removal, for example, Q9 & Q10 in the current study) and cross-loading (criteria and process, potential cross-loading items based on table 2, Q6, Q12, Q15 & Q20) during factor analysis.<br>5.3 It seemed that authors calculate the factor scores based on the regression scoring method, but the details were missing,

	especially how the coefficients were determined. Did the authors use the results of factor loading to generate the formula for regression scoring? If so, pls add this information. In addition, the results of the overall score of 6 factors were missing in the main content.<br>5.4 Authors stated that they adopted multilevel modeling. However, the number of levels was not indicated, namely, three-level or two-level. In addition, what are the different levels, for example, hospital-level, department-level, and individual-level? I strongly recommended authors to present the formula for multi-level modeling, which can be clear to present the different levels and within each level, what factors were considered.<br>6. Discussion-2nd and 3rd paragraph<br>I'm confused that how the authors concluded "visibility (diagnostics), awareness (education) and institutional form (governance)" were the three main issues regarding AMR and AMS in Indonesia based on the results of the current study. The main results of the current study were the six underlying latent constructs identified by factor analysis, namely, awareness of AMS activities; awareness of AMS purpose; views regarding rational antibiotic prescribing; confidence in antibiotic prescribing decisions; perception of AMR as a significant problem; and immediate actions to contain AMR. How authors inferred the results of these six latent constructs into the three aspects was missing.<br>On the other hand, why do authors infer that the "externality of AMR" was the most significant factor? To my knowledge, doctors' rational use of antibiotics and perception of AMR were complex. According to existing studies, the attitudes to comply with patients, fear of adverse events, lack of incentives, ignorance of overuse of antibiotics and AMR, limited diagnostic supporting, limited ability to rational use antibiotics, limited ability to communicate with patients to address pressure, insufficient knowledge, availability of antibiotics and restriction of policies, all these factors contributed to the prevalent overuse of antibiotics and to an extent resulted in the AMR. Among them, the doctor's responsibility avoidance (similar to the externality of AMR) was one of these factors. Authors may need to provide evidence to justify this statement that why the externality of AMR was the most significant factor under the context in hospitals in Indonesia.<br>7. Discussion-4th paragraph<br>In the paragraph, the authors stated the results that different hospitals contributed to the differences of some factors in the current study. However, the reasons underlying the differences were not fully discussed. Authors may need to present possible reasons why the different participating hospitals contributed to the differences of awareness of AMS purposes, views regarding rational antibiotic prescribing, perception of AMR as a significant problem, and immediate actions to contain AMR.<br>8. Discussion- 5th paragraph<br>Despite the effect of the unknown of the residents' unknown, another plausible reason for their high confidence in antibiotic prescribing may be the expectation effect of survey, namely respondents responded to the survey as they were expected to be.<br>9. Table 2-Column of Factor 2<br>The number of kept decimal points was different compared with other columns.

**REVIEWER**	Herawati, Fauna

	University of Surabaya, Department of Clinical and Community Pharmacy, Faculty of Pharmacy
**REVIEW RETURNED**	30-Dec-2021

**GENERAL COMMENTS**	Thank you for clarifying the importance of some variables.

VERSION 2 – AUTHOR RESPONSE

Reviewer #2: Dr. Fauna Herawati, University of Surabaya

Reviewer comment: Thank you for clarifying the importance of some variables.

Author response: We thank the Reviewer for the positive feedback.

Reviewer #1: Dr. Chenxi Liu, Huazhong University of Science and Technology Tongji Medical College

Reviewer comment #1. Background:

The study was conducted in hospitals, while the overuse of antibiotics was mainly occurred in primary care according to existing studies. I recommended that authors may need to add a brief summary of the role of hospitals in the overall AMR and overuse of antibiotics in Indonesia, why the current study focused on hospitals. This information may help readers understand the significance of the current study and contextualize the results into the overall context of Indonesia.

Author response #1:

We agree with the reviewer and acknowledge the important contribution of antibiotic use in the community as a driver of AMR in human health. Antibiotic self-medication and non-prescription dispensing are often cited as critical drivers of AMR.

Nonetheless, given the overall dearth of data from Indonesia, our current study's focus on hospital settings seems well justified for the following reasons:

• Hospitals are facing significant AMR challenges, especially driven by the over-prescribing of broad-spectrum antibiotics. Patients infected with drug-resistant bacteria causing common infections in hospitalised populations are more likely to receive inappropriate antibiotic therapy resulting in higher mortality rates and opportunities for spreading to other patients [1–3].

• Our group is close to completing a systematic review on antibiotic use in Indonesia, spanning the past 20 years (protocol registered in the PROSPERO database CRD42019134641). This Review has identified critical gaps in antibiotic practices both in hospitals and communities, highlighting that enhanced stewardship interventions are urgently needed across both settings (Limato et al, unpublished).

• Hospital settings are included as one of the focus areas for policy and research according to the WHO Global Action Plan [4].

We have added a clarifying sentence to the Background section, as well as a study limitation (Discussion) that the current study focuses on hospital settings, while acknowledging the importance of community AMR.

Reviewer comment #2. Methods-Study design and setting

Authors may need to present the inclusion and exclusion criteria of selected hospitals in the current study. How many hospitals there in Jakarta and how these 6 hospitals were selected? Why the six hospitals were selected and to what extent that the 6 hospitals were able to represent the status quo in Jakarta (for example, the 6 hospitals provide 80% outpatients/inpatients services in this area)?

Author response #2

This study sought to include a purposive sample of hospitals, representing the diversity of hospital types in Jakarta (refer to Table 1) in terms of location (5 districts of Jakarta Province), size, health sector (public and private) and health care level (tertiary and secondary). Based on 2018 data, a total of 189 hospitals were operational in Jakarta province, of which 59 were public and 130 private, with a total of 15,444 and 15,425 inpatient beds, respectively [5]. We then pragmatically selected a varied sample, based on existing collaborations or affiliation with the Faculty of Medicine Universitas Indonesia and/or Eijkman-Oxford Clinical Research Unit, their willingness to participate in the study, and within the resources available to us to conduct the study. We clearly stated in the study limitations that the study sample was not representative for Jakarta or the country. We included further details in the Methods section under “Study setting”.

We would also like to note that the physician KAP survey, presented in this paper, was part of a larger study that adopted a range of methodologies to explore targets for stewardship interventions in the six participant hospitals. The overall findings of the study will be reported in a DPhil thesis and a series of forthcoming quantitative and qualitative papers (first paper can be found here: https://doi.org/10.1093/jacamr/dlab047).

Reviewer comment #3. Methods- Study respondents

The authors stated a purposive sampling in the current study. However, it seemed that the process of sampling described in respondent recruitment was a full sample within each hospital (all eligible doctors were approached)? Thus, I’m confused about the sampling process. Did the authors adopt a two-stage sampling method with purposive sampling on department-level and a full sample within each department? If so, authors may need to present how the purposive sampling was able to cover “all departments, work experience, and professional hierarchy, to ensure representation of different antibiotic practice and views, perceptions, experiences related to AMR and AMS”. To my knowledge, it seemed that random sampling methods were more likely to generate such a sample. Was there any reason considered within the sampling process for purposive sampling? Pls, add such information.

Author response #3

The Reviewer is correct: the hospitals were purposely selected, as described in author response #2. Within each participant hospital, we approached and invited all antibiotic prescribing physicians

across all clinical departments for participation in the survey. We added a further clarification to the Methods section under study respondents.

Reviewer comment #4. Methods- Study respondents

What are the others? Did they represent the other departments in table S1? Are they doctors and able to prescribe antibiotics in Indonesia? (For example, Laboratory, Microbiology) If not, they seemed are not able to be included in the current study?

Author response #4

All the doctors included in the survey were qualified to prescribe antibiotics and were therefore included in the survey according to the survey selection criteria. The “Others” represent a small and heterogenous subgroup (N=18) that could not be classified in any of the other four categories (such as dentists) and these are described in Table S1. No firm inference can be derived about this subgroup.

Reviewer comment #5. Methods- Statistical analysis

5.1 Authors conducted the screen plot and parallel analysis to determine the numbers of eigenvalues. But they were missed in the statistical analysis. Pls, add a description of these methods and processes.

Author response #5.1

We included further details under the “Statistical analysis” section of how we determined the optimal factor solution, using a combination of approaches (Kaiser criterion, scree plot and Horn’s parallel analysis).

5.2. Authors may need to add information regarding how they address insufficient loading (criteria for item removal, for example, Q9 & Q10 in the current study) and cross-loading (criteria and process, potential cross-loading items based on table 2, Q6, Q12, Q15 & Q20) during factor analysis.

Author response #5.2

To interpret the factor solutions, we examined the factor loadings, and assigned each item to a certain factor based on the highest absolute factor loading (minimum loading 0.30), and then produced an umbrella term for each factor that best characterizes the joint meaning of all the variables associated with it. If a certain item did not fit with the factor structure (e.g. the results were not interpretable, or a factor included <3 items), the analysis was re-run without the pertinent item(s). This was deemed justified as this analysis was exploratory and we wanted the results to be interpretable and actionable. We added above details the “Statistical analysis” section. The process of achieving a suitable factor solution is described in the Results section.

5.3. It seemed that authors calculate the factor scores based on the regression scoring method, but the details were missing, especially how the coefficients were determined. Did the authors use the results of factor loading to generate the formula for regression scoring? If so, pls add this information. In addition, the results of the overall score of 6 factors were missing in the main content.

Author response #5.3:

The Reviewer is correct; we used the regression scoring method to compute the factor scores based on the factor loadings. We added this to the “Statistical analysis” section. The factor analysis is presented in Tables 2 and 3 and S2.

5.4. Authors stated that they adopted multilevel modelling. However, the number of levels was not indicated, namely, three-level or two-level. In addition, what are the different levels, for example, hospital-level, department-level, and individual-level? I strongly recommended authors to present the formula for multi-level modelling, which can be clear to present the different levels and within each level, what factors were considered.

Author response #5.4:

The results of the regression analysis are presented in Table 4. We used two levels to adjust for inter-observation clustering between hospitals. The Stata command we used for the model was: xtmixed factor[x] [independent variables] || hospital_id

The multivariable mixed-effect linear regression models included five independent variables of interest: hospital (#1-6), department, medical hierarchy, work experience, health sector, health care level, and each of the factor scores (#1-6) as the dependent variable. Each model adjusted for the possible interdependence of observations clustered within hospitals, as well as for the confounders sex and AMS training. We added some clarifications to the “Statistical analysis” section and the Table 4 footnote.

Reviewer comment #6. Discussion-2nd and 3rd paragraph

I’m confused that how the authors concluded “visibility (diagnostics), awareness (education) and institutional form (governance)” were the three main issues regarding AMR and AMS in Indonesia based on the results of the current study. The main results of the current study were the six underlying latent constructs identified by factor analysis, namely, awareness of AMS activities; awareness of AMS purpose; views regarding rational antibiotic prescribing; confidence in antibiotic prescribing decisions; perception of AMR as a significant problem; and immediate actions to contain AMR. How authors inferred the results of these six latent constructs into the three aspects was missing.

On the other hand, why do authors infer that the “externality of AMR” was the most significant factor? To my knowledge, doctors’ rational use of antibiotics and perception of AMR were complex. According to existing studies, the attitudes to comply with patients, fear of adverse events, lack of incentives, ignorance of overuse of antibiotics and AMR, limited diagnostic supporting, limited ability to rational use antibiotics, limited ability to communicate with patients to address pressure, insufficient knowledge, availability of antibiotics and restriction of policies, all these factors contributed to the prevalent overuse of antibiotics and to an extent resulted in the AMR. Among them, the doctor’s responsibility avoidance (similar to the externality of AMR) was one of these factors. Authors may need to provide evidence to justify this statement that why the externality of AMR was the most significant factor under the context in hospitals in Indonesia.

Author response #6:

We appreciate the Reviewer's comment, and we have attempted to address their concern by making several revisions to the Results and Discussion sections, aimed at improving the logical flow of thought, as follows:

1. We changed the sequence of the Results sections: we first present the descriptive results of the questionnaire (based on the 3 questionnaire themes), followed by the EFA analysis (based on the 6 underlying constructs) and, lastly, we further explored patterns in the physician subgroup analysis;

2. In the Discussion, we attempted to elevate the discussion by connecting the six underlying constructs to three higher-level overarching key issues identified as awareness, diagnostics and governance, while making sure the factors were connected to each of those.

3. We have opted to dedicate the next sections of the Discussion to highlight several specific themes that seem to be most relevant to the local context, rather than elaborating on the well-known, complex factors associated with irrational prescribing (as referenced by the Reviewer as well as in the paper's introduction). These context-specific key issues are related to risk, uncertainty and sense of responsibility. Several of the constructs come together here and we introduce the concept of "externality of AMR as a problem" as a common theme. We have now toned down "externality of AMR" describing it as an obstacle that hinders effective AMS implementation in this context, instead of calling it the "most significant factor".

We hope these revisions will satisfy the Reviewer.

Reviewer comment #7. Discussion-4th paragraph

In the paragraph, the authors stated the results that different hospitals contributed to the differences of some factors in the current study. However, the reasons underlying the differences were not fully discussed. Authors may need to present possible reasons why the different participating hospitals contributed to the differences of awareness of AMS purposes, views regarding rational antibiotic prescribing, perception of AMR as a significant problem, and immediate actions to contain AMR.

Author response #7:

We appreciate the Reviewer's comment. The identified differences in factor scores between the 6 hospitals could not be attributed or related to their hospital sector (public versus private) or health care level (secondary versus tertiary), in the multivariable models. We did not assess any other hospital-level characteristics.

However, the multivariable analysis identified several differences between physician subgroups across the participating hospitals regarding awareness of AMS purposes, views regarding rational antibiotic prescribing, perception of AMR as a significant problem and immediate actions to contain AMR (Table 4). Citing relevant literature, the Discussion reflects on the influences of the physicians' work experience, medical hierarchy, department on the pertinent factors.

Reviewer comment #8. Discussion- 5th paragraph

Despite the effect of the unknown of the residents' unknown, another plausible reason for their high confidence in antibiotic prescribing may be the expectation effect of survey, namely respondents responded to the survey as they were expected to be.

Author response #8:

We acknowledge that desirability bias may have influenced survey results, and we have included this in the study limitations. Based on data and observations from the larger study (including stakeholder interviews, ethnographic observations), we have no indication that residents would be more inclined than the other physician subgroups to exhibit socially desirable responses to this anonymous survey. We added to the Discussion that residents may be more inclined to follow prescribing guidelines compared to previous generations, because of their contemporary medical training.

Reviewer comment #9. Table 2-Column of Factor 2

The number of kept decimal points was different compared with other columns.

Author response #9:

We made the correction.
